# Enantioselective Photochemical Generation of a Short‐Lived, Twisted Cycloheptenone Isomer: Catalytic Formation, Detection, and Consecutive Chemistry

**DOI:** 10.1002/anie.202501433

**Published:** 2025-04-21

**Authors:** Max Stierle, Constantin Jaschke, Daniel J. Grenda, Martin T. Peschel, Thomas Pickl, Niklas Gessner, Patrick Nuernberger, Benjamin P. Fingerhut, Christian Ochsenfeld, Regina de Vivie‐Riedle, Thorsten Bach

**Affiliations:** ^1^ Department Chemie and Catalysis Research Center (CRC) School of Natural Sciences Technische Universität München D‐85747 Garching Germany; ^2^ Department of Chemistry Ludwig‐Maximilians‐Universität München D‐81377 München Germany; ^3^ Institut für Physikalische und Theoretische Chemie Universität Regensburg D‐93053 Regensburg Germany

**Keywords:** Computational chemistry, Cycloaddition, Enantioselectivity, Isomerization, Photochemistry

## Abstract

Cyclohept‐2‐enone‐3‐carboxylic acid undergoes a photochemical isomerization from its *cis*‐ to its *trans*‐form either upon direct irradiation (*λ* = 366 nm) or in the presence of a triplet sensitizer (*λ* = 459 nm). The intermediate chiral *trans*‐isomer was detected by step‐scan FTIR, displaying a lifetime of 130 µs (r.t., CH_2_Cl_2_). Ensuing Diels–Alder reactions of the *trans*‐isomer occurred smoothly and produced chiral *trans*‐fused cycloaddition products (14 examples, 24%–98% yield). Benzylation led to esters, which were separated by chiral HPLC and which were employed to evaluate a possible enantioselective reaction course. It was discovered that a chiral phosphoric acid with a pendant sensitizing group induces a notable enantioselectivity in the photoisomerization step. The planar chirality of the *trans*‐cycloheptene translates into point chirality in the Diels–Alder reaction (seven examples, up to 38% *ee*). Computational studies suggest that the chiral conformation of the *cis*‐isomer adopted within the assembly to the chiral phosphoric acid induces the enantioselectivity in a one‐bond flip (OBF) toward the *trans*‐isomer. Trajectory surface hopping (TSH) simulations showed exemplarily how a chiral *trans*‐cyclohept‐2‐enone is formed from a chiral *cis*‐conformer. For the Diels–Alder reaction, a weak ground state selectivity was found to attenuate the enantioselectivity achieved in the photochemical step.

## Graphical abstract

In a proof‐of‐concept study, it was for the first time shown that planar chiral *trans*‐cycloalk‐2‐enones, such as **2**, can be formed enantioselectively from their *cis*‐isomers by triplet energy transfer from a chiral catalyst. Computational studies performed with cyclohept‐2‐enone and enone **1** provided invaluable insights into the mode of action of the sensitizing thioxanthone catalyst.

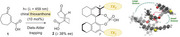

## Introduction

**Scheme 1:**
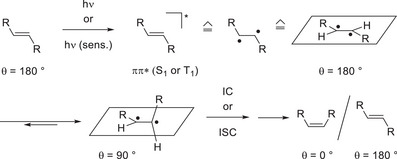
Photochemical isomerization of olefins either by direct excitation to S_1_ or sensitization to T_1_ (IC = internal conversion; ISC = intersystem crossing).

Olefins can reach their first excited singlet state (S_1_) by direct excitation or their first excited triplet state (T_1_) by sensitization. Since either state is typically ππ* in character, the olefin loses its configurational stability, and an out‐of‐plane rotation of its substituents becomes feasible. While the dihedral angle *θ* of two *trans*‐positioned, vicinal substituents *R* is typically approx. 180° in the ground state, the substituents move away from each other in the excited state and adopt a conformation in which the dihedral angle approaches 90°. Once the olefin returns to the ground state, the planar arrangement is restored, and the two substituents can be either *cis*‐ (*θ* = 0°) or *trans*‐positioned (*θ* = 180°). For acyclic olefins, it has been shown that a counter‐thermodynamic^[^ ^1^, ^2^, ^3^ ^]^ isomerization from a *trans*‐ to a *cis*‐olefin is feasible either by choosing an irradiation wavelength at which the *trans*‐isomer is excited but the *cis*‐isomer is not or by selecting a sensitizer (sens.) with a triplet energy which is between the triplet energy of the *trans*‐ and the *cis*‐isomer (Scheme 1).^[^ ^4^, ^5^, ^6^, ^7^ ^]^

**Scheme 2:**
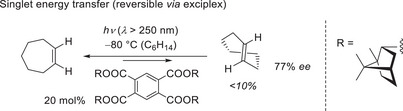
Singlet energy transfer as a vehicle to achieve an enantioselective photoisomerization:^[^ ^15^ ^]^ Low yield of enantioenriched *trans*‐cycloheptene.

For cyclic olefins with a ring size *n* = 4–10, the *cis*‐isomer is typically more stable than the *trans*‐isomer^[^ ^8^ ^]^ and the ring restricts free rotation even in an excited ππ* state. Starting from cyclohexenes (*n* = 6), the *trans*‐isomer is sufficiently stable to be detected and to be trapped in consecutive reactions (vide infra).^[^ ^9^, ^10^, ^11^, ^12^, ^13^ ^]^ An intriguing fact pertinent to *trans*‐cycloalkenes is their chirality, with the plane of chirality defined by the carbon atoms and the substituents of the olefin. It should, thus, be possible to create *trans*‐cycloalkenes enantioselectively and to perform consecutive reactions which ideally occur with a high degree of chirality transfer. Pioneering work by Inoue and co‐workers^[^ ^14^, ^15^, ^16^, ^17^ ^]^ has established the enantioselective formation of *trans*‐cycloheptene by singlet energy transfer from chiral menthyl‐ and bornyl‐derived catalysts (20 mol%). Although the enantioselectivity of the process reached up to 77% *ee* (enantiomeric excess), it suffered from the fact that the *trans*‐isomer remained the minor diastereoisomer formed in a ratio of *trans*/*cis* = 10/90 at best (Scheme 2). A yield of 6% was recorded in a preparative run in which racemic *trans*‐cycloheptene was trapped by 1,3‐diphenylisobenzofuran in a Diels–Alder reaction.^[^ ^15^ ^]^

While the enantioselectivities achieved by singlet energy transfer catalysis are notable, the intrinsic issue that the catalyst also promotes the reverse reaction to the *cis*‐cycloalkene has not been overcome. In situ trapping of the *trans*‐isomer is difficult due to the low reaction temperature and the short wavelength employed in the transformation. Triplet energy transfer could offer a solution to this challenge since it occurs under more suitable conditions and is strongly distance dependent. Geometric and thermodynamic constraints might be favorably employed to steer the directionality of the isomerization. However, there has been no conceptual proof that triplet energy transfer can be successfully employed for the enantioselective photoisomerization of *cis*‐cycloalkenes.

**Scheme 3:**
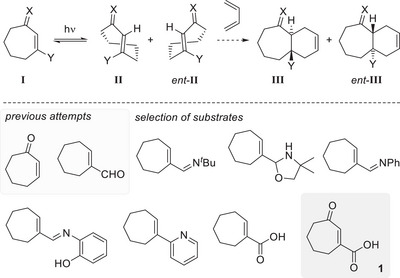
Top: The isomerization of cycloheptenes **I** leads to chiral twisted *trans*‐isomers **II** and *ent*‐**II,** which should be competent to undergo a subsequent Diels–Alder reaction leading to **III** (from **II**) and to *ent*‐**III** (from *ent*‐**II**). Bottom: Previously studied cycloheptenes and a selection of alternative substrates from which cyclohept‐2‐enone‐3‐carboxylic acid (**1**) evolved as a suitable isomerization precursor.

Our group has for some time studied the photoisomerization of substituted cycloheptenes **I** and their subsequent Diels–Alder reactions (Scheme 3).^[^ ^18^, ^19^ ^]^ The key idea was to enforce the enantioselective formation of the strained *trans*‐isomer **II** from the *cis*‐isomer **I** by employing either a chiral Lewis acid^[^ ^20^ ^]^ or a chiral triplet sensitizer.^[^ ^21^ ^]^ A Diels–Alder reaction, schematically shown for 1,3‐butadiene, would deliver with high chirality transfer *trans*‐fused product **III**. Accordingly, enantiomer *ent*‐**II** would deliver the mirror image *ent*‐**III**. Although we could show that the reaction of the *trans*‐isomers with various dienes proceeds in very good yields both for cyclohept‐2‐enones^[^ ^18^ ^]^ and for cyclohept‐1‐ene‐1‐carbaldehydes,^[^ ^19^ ^]^ attempts to render the reactions enantioselective remained futile. In the present study, we have attempted to identify cycloheptene substrates that would display a binding motif suitable for association with a chiral phosphoric acid. If the latter compound displayed a sensitizing substituent, an energy transfer would be possible, which in turn would populate one of the enantiomeric *trans*‐isomers selectively. Several cycloheptenes were evaluated in racemic reactions from which cyclohept‐2‐enone‐3‐carboxylic acid (**1**) evolved as a suitable candidate.

It was found that the in situ generated *trans*‐isomer reacts with a variety of dienes, forming intriguing tricyclic lactones. A comprehensive computational study suggests that a conformational bias prior to the photoisomerization was responsible for the formation of either enantiomeric *trans*‐isomer. The experiments culminated in the first enantioselective cascade of a sensitized *cis*/*trans* isomerization and a Diels–Alder reaction. A full account of the details of our study is presented in this research article.

## Results and Discussion

**Figure 1:**
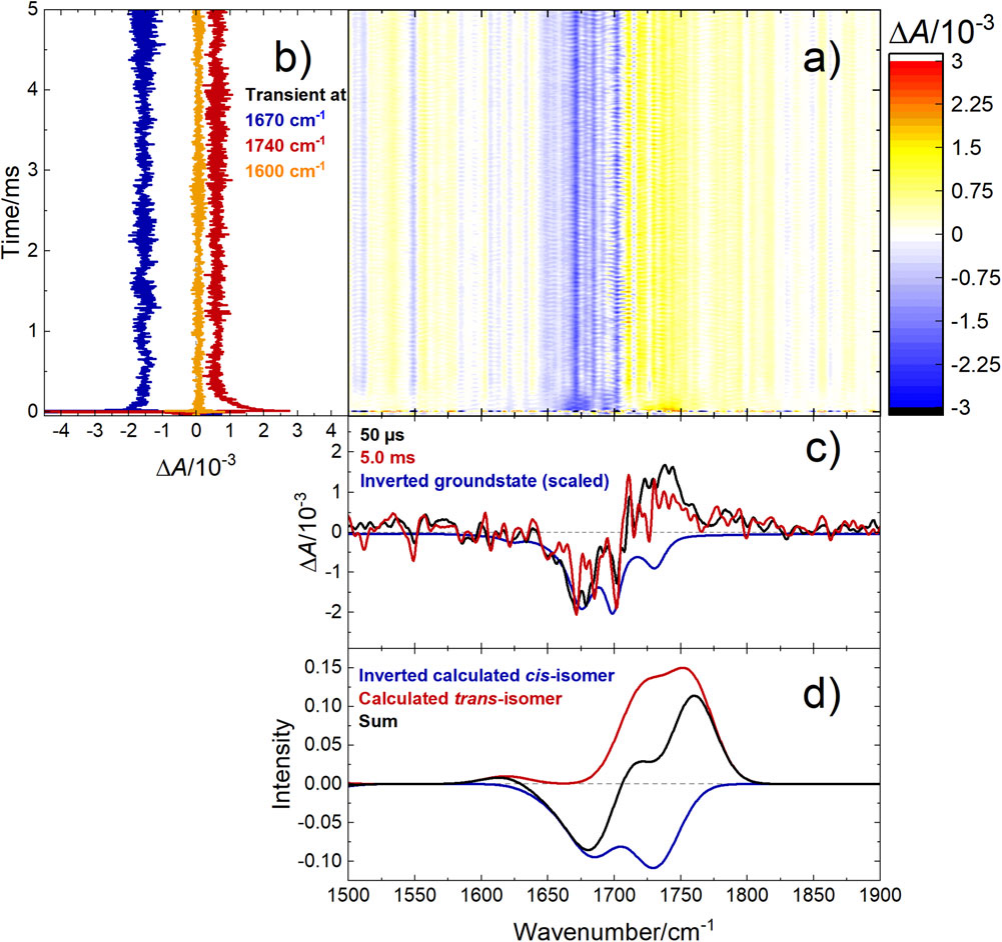
a) Step‐scan FTIR data for **1** in CH_2_Cl_2_ (*c* = 71.8 mM) at room temperature. b) Evolution of bleached *cis*‐isomer **1** at 1670 cm^−1^ and transiently formed *trans*‐isomer (*rac*‐**2**) at 1740 cm^−1^, as well as the baseline at 1600 cm^−1^. c) Spectra directly after excitation (black), toward the end of the measurement (red), and the steady‐state spectrum of **1** (blue, inverted and scaled). d) Calculated spectra {[CPCM(CH_2_Cl_2_)]‐R^2^SCAN‐3C/def2‐mTZVPP, see the Supporting Information for details} for **1** (blue, inverted), *rac*‐**2** (red), and their difference mimicking the difference spectra after excitation (black). Note that oscillatory signals of electronic origin had to be removed from the raw data (see Supporting Information for details).

*Nature of the excited state and racemic reactions*. There are several techniques available to determine the lifetime of twisted *trans*‐cycloheptenes. Early studies with UV illumination at temperatures of 113 K or lower showed the formation of *trans*‐cyclohepten‐2‐one, which was stable for more than an hour.^[^ ^22^, ^23^ ^]^ Transient studies by Bonneau and co‐workers revealed a lifetime of hours for *trans*‐1‐phenylcycloheptene at low temperatures as well and could even demonstrate light‐induced back‐isomerization; at ambient temperature, a lifetime of 250 s was reported in cyclohexane.^[^ ^24^ ^]^ For *trans*‐cyclohept‐2‐enone (*c* ≈ 100 µM), room‐temperature studies yielded lifetimes of 45 s in cyclohexane, but much lower values in polar protic solvents, e.g., 33 ms in methanol.^[^ ^25^ ^]^ Inoue and his group determined the lifetime of *trans*‐cycloheptene by trapping experiments with methanol at various temperatures (*c* = 0.01 m), which was extrapolated to 45 s at ambient temperature.^[^ ^26^, ^27^ ^]^ A lifetime of 7.1 min at −78 °C was determined for *cis*,*trans*‐cycloheptadiene by Inoue and co‐workers from transient UV–vis data (*c* = 0.12 mm) in isopentane‐methylcyclohexane (3:1).^[^ ^28^ ^]^ We investigated cycloheptene **1** in CH_2_Cl_2_ at room temperature by step‐scan FTIR spectroscopy with a ns pump laser.^[^ ^29^, ^30^, ^31^ ^]^ The transient IR data showed the ground‐state bleach of *cis*‐configured **1** together with a newly formed absorption associated with *trans*‐isomer *rac*‐**2** (Figure 1). While the former remained constant, the latter decayed with a lifetime of about 130 µs toward yet another carbonyl signal, which may be attributed to the [2+2] photocycloaddition product (see the Supporting Information for details). The relatively short lifetime appears to be testimony to the higher strain induced by the additional substitution, but it also suggests that the transient intermediate was sufficiently long‐lived to participate in trapping studies.

**Scheme 4:**
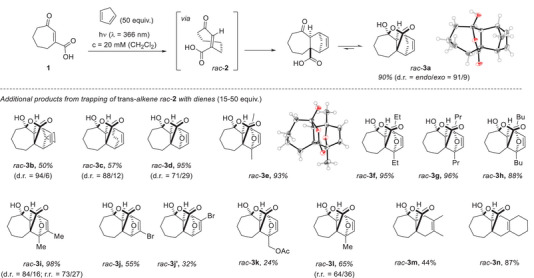
Scope and limitations of the photochemical isomerization of cyclohept‐2‐enone‐3‐carboxylic acid (**1**) and subsequent trapping of the racemic *trans*‐isomer *rac*‐**2** by various dienes. In most cases, a lactonization/hemiacetal formation occurs, and this structure is depicted for the racemic products *rac*‐**3**.

Preliminary synthetic experiments were performed with various cycloheptenes and 1,3‐cyclopentadiene as the diene component. It was found that some imine‐derived cycloheptenes (see Scheme 3) suffered from stability issues and that pyridyl‐ and hydroxylcarbonyl‐substituted derivatives were not sufficiently reactive. In notable contrast, irradiation of cyclohept‐2‐enone‐3‐carboxylic acid (**1**) at *λ* = 366 nm resulted in clean product formation, supporting the notion that the lifetime of the transient isomer *rac*‐**2** allows for a successful bimolecular reaction. Optimization revealed dichloromethane as the superior solvent and a 1 m concentration of the diene (50 equiv.) as optimal (Scheme 4). The Diels–Alder product^[^ ^32^, ^33^, ^34^, ^35^, ^36^, ^37^ ^]^ *rac*‐**3a** displayed the expected *trans*‐configuration at the ring conjunction, and the *endo*‐isomer was clearly preferred, i.e., the approach of the diene double bond had occurred *endo* to the carbonyl carbon atom. The diastereomeric ratio (d.r. = *endo*/*exo*) was 91/9 in favor of the *endo*‐product, with the total yield being 90%. Due to the *trans* ring fusion, the carboxylic acid group gets into the proximity of the carbonyl group, enabling the formation of a lactone‐type hemiacetal, which was, for compound *rac*‐**3a**, the only detectable species. Single‐crystal structure analysis confirmed the constitution and relative configuration of the compound.^[^ ^38^ ^]^

**Scheme 5:**
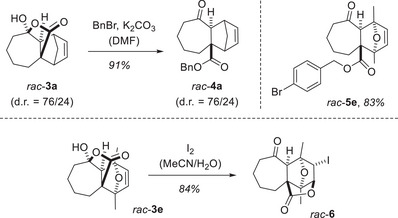
Consecutive reactions of the Diels–Alder products *rac*‐**3**. Top: Benzylation leads to products *rac*‐**4a** and *rac*‐**5e**, which can be detected and are separable by chiral HPLC. Bottom: Iodolactonization of compound *rac*‐**3e** provides iodide *rac*‐**6** in diastereomerically pure form.

The novel structural feature of the products from the isomerization/Diels–Alder reaction/lactonization cascade warranted a more extensive exploration of the reaction scope. Several other dienes were employed in the sequence, and the respective products *rac*‐**3** fully characterized. Six‐membered dienes reacted well and displayed—like 1,3‐cyclopentadiene—a preference for the *endo* product (products *rac*‐**3b** and *rac*‐**3c**). Furans were found to be suitable dienes, and the parent compound produced a mixture of two diastereoisomers, with the *endo*‐product *rac*‐**3e** prevailing. 2,5‐Disubstituted furans gave in full analogy to the parent compound, the cascade products *rac*‐**3e**‐*rac*‐**3** **h** in high yields as single isomers. For representative product *rac*‐**3e**, it was shown by single‐crystal X‐ray crystallography that the Diels–Alder reaction proceeded in an *endo*‐fashion.^[^ ^39^ ^]^ 2,3‐Dimethylfuran resulted in an inseparable product mixture with the shown isomer *rac*‐**3i** being predominant (r.r. = regioisomeric ratio). The two regioisomeric products *rac*‐**3j** and *rac*‐**3j**’ stemming from the reaction of 3‐bromofuran could be separated and were free from any diastereoisomers. 2‐Substituted furans also underwent the cascade reaction, but the regioselectivity was less pronounced. For 2‐acetoxymethylfuran, only a single product isomer, *rac*‐**3k** was isolated, but the low yield indicated that the other regioisomer was likely lost during work‐up or was too unstable to be isolated. 2‐Methylfuran delivered both conceivable regioisomers as an inseparable mixture with a preference for the shown isomer *rac*‐**3l**. Acyclic dienes (2,3‐dimethylbuta‐1,3‐diene, 1,2‐bismethylene‐cyclohexane) were competent reaction partners, but the respective products *rac*‐**3** **m** and *rac*‐**3n** delivered poorly resolved NMR spectra. It appeared as if there was an equilibrium between the lactone and the free carboxylic acid form, which led to line broadening of the NMR signals. To facilitate the isolation of single compounds from Diels–Alder products *rac*‐**3** **m** and *rac*‐**3n,** a consecutive benzylation of the carboxylic acid was pursued. In addition, it was desirable to convert the weakly UV‐active compounds *rac*‐**3** into better detectable products for HPLC analysis. In fact, racemic samples were required for unequivocal *ee* determination in the projected enantioselective reactions. Taking Diels–Alder product *rac*‐**3a** (d.r. = 76/24) as a test case, we found treatment with benzyl bromide and potassium carbonate in DMF to be an operationally simple benzylation protocol (Scheme 5).

The desired ester *rac*‐**4a** was obtained in 91% yield (d.r. = 76/24). The reaction **3** → **4** is also applicable to the other lactones, and the relative configuration of product *rac*‐**4e** was confirmed by single‐crystal X‐ray crystallography (see the Supporting Information for details).^[^ ^40^ ^]^ The *para*‐bromobenzyl ester *rac*‐**5e** was obtained from Diels–Alder product *rac*‐**3e** by benzylation with *para*‐bromobenzyl bromide. The heavy atom was considered useful in the enantioenriched series (vide infra) for determining the absolute configuration. The same starting material, *rac*‐**3e**, delivered upon treatment with iodine^[^ ^41^ ^]^ the halolactonization product *rac*‐**6**. The reaction confirmed the *endo* position of the double bond in product *rac*‐**3e** since an *exo*‐positioned double bond would not be available for halolactonization. The tetracyclic skeleton so generated has not been previously reported.

**Figure 2:**
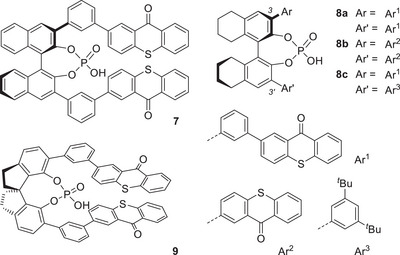
Structure of chiral phosphoric acids **7**–**9** used for triplet energy transfer to cyclohept‐2‐enone‐3‐carboxylic acid (**1**) in an attempted photochemical isomerization to the twisted isomer **2**.

*Enantioselective reactions employing a sensitizing phosphoric acid*. Our group^[^ ^42^, ^43^ ^]^ and others^[^ ^44^, ^45^, ^46^, ^47^, ^48^, ^49^, ^50^, ^51^, ^52^, ^53^, ^54^ ^]^ have for some time studied chiral phosphoric acids with pendant sensitizing groups as chiral catalysts for enantioselective photochemical reactions. So far, the focus has been on the discrimination of enantiotopic faces upon substrate binding to the phosphoric acid. To the best of our knowledge, there have not been any studies devoted to enantioselective photochemical isomerization reactions.^[^ ^55^, ^56^, ^57^, ^58^ ^]^ In this regard, there was no clear rationale as to the structure of a potential catalyst nor to its mode of action. Since the known *C*_2_‐symmetric acid **7** had shown promising results in [2+2] photocycloaddition chemistry,^[^ ^42^, ^43^ ^]^ it was taken as a blueprint for catalyst design (Figure 2).

The thioxanthone chromophore was considered suitable for energy transfer to cyclohept‐2‐enone‐3‐carboxylic acid (**1**). Starting from (*R*)‐(+)‐5,5′,6,6′,7,7′,8,8′‐octahydro‐1,1′‐bi‐2‐naphthol (octahydrobinol) and from (*R*)‐2,2′,3,3′‐tetrahydro‐1,1′‐spirobis[indene]‐7,7′‐diol (spinol), the analogous *C*_2_‐symmetric catalysts **8a** and **9** were prepared (see the Supporting Information for details). Another *C*_2_‐symmetric phosphoric acid **8b**, lacking the phenyl spacer, was prepared from octahydrobinol. In addition, a fifth catalyst, **8c**, was added to the selection, which lacked *C*_2_‐symmetry but rather displayed a lipophilic aryl group at position C3’. Spectral features for catalysts **8a**, **8b**, and **9** were similar in that all three of them showed an absorption maximum around *λ* = 400 nm in their UV–vis spectrum with a tail extending in all cases far into the visible range (*λ* ≤ 470 nm). The triplet energies (*E*_T_) determined from their phosphorescence spectra (77 K, CH_2_Cl_2_) were found to be almost identical, with *E*_T_ varying between 252 and 253 kJ mol^−1^ (±2 kJ mol^−1^). The values are higher than the previously determined triplet energy of catalyst **7** (*E*_T_ = 235 ± 2 kJ mol^−1^).^[^ ^42^ ^]^ The red‐shifted emission is in line with a lower singlet state (S_1_) energy of the latter compound as compared to thioxanthenes **8a**, **8b**, and **9**. The energy for the 0–0 transition determined from the crossing point of absorption and fluorescence emission was *E*(S_1_) = 286 kJ mol^−1^ for **7**, *E*(S_1_) = 288 kJ mol^−1^ for **8a**, *E*(S_1_) = 292 kJ mol^−1^ for **8b**, and *E*(S_1_) = 290 kJ mol^−1^ for **9**.

Studies toward an enantioselective photochemical isomerization followed by a Diels–Alder reaction were performed with 2,5‐dimethylfuran as the diene component. Although enone **1** displays no distinct absorption at a wavelength *λ* > 400 nm, there was some notable background reaction upon irradiation with light‐emitting diodes (LEDs) emitting at *λ*_em_ ≤ 437 nm. Thus, we selected an irradiation source with an emission maximum (*λ*_em_ = 459 nm) that barely touched the long‐wavelength absorption of the catalyst. Not surprisingly, the reaction **1** → **3e**/*ent*‐**3e** was significantly slower than in the racemic case. The primary cascade products were directly taken into the benzylation reaction, and the ratio of benzyl esters **4e** and *ent*‐**4e** was quantified by chiral HPLC after isolation. We were delighted to see that already at ambient temperature, there was a notable preference for one enantiomer over the other when employing 10 mol% of catalyst **7** (Table 1, entry 1). The enantioselectivity improved when decreasing the temperature to −20 °C (entry 2), but no improvement was recorded when further lowering the reaction temperature (entry 3) or when increasing the catalyst loading (entry 4). Although the product yield increased slightly when using other solvents (DCE = 1,2‐dichloroethane) but dichloromethane (entries 5 and 6), the loss in selectivity made a solvent switch unattractive. Among the octahydrobinol‐ and spinol‐derived catalysts (entries 7–10), catalyst **8a** clearly outperformed the others in terms of yield while the enantioselectivity remained similar throughout the series. The switch in the preference of *ent*‐**4e** over **4e** for spinol‐derived catalyst **9** was no surprise given the fact that its axis of chirality has an opposite direction than the stereogenic axis of catalysts **7** and **8**. The absolute configuration of the major enantiomer was deduced from the reaction **1** → **3e**/*ent*‐**3e** in the presence of catalyst **8a**. Instead of derivatizing the primary product with benzyl bromide as done in entry 7, the reaction mixture was treated with *para*‐bromobenzyl bromide, delivering a mixture of products **5e**/*ent*‐**5e** (vide infra). Separation of the enantiomeric bromo compounds by chiral HPLC delivered enantiomerically pure samples of both enantiomers. The minor enantiomer *ent*‐**5e** gave crystals that were suitable to determine its absolute configuration by anomalous X‐ray diffraction (see the Supporting Information for further details).^[^ ^59^ ^]^

**Table 1:** Optimization of reaction conditions for a sequence of enantioselective *E*/*Z*‐isomerization, Diels–Alder reaction, and benzylation to generate enantioenriched product **4e** from cyclohept‐2‐enone‐3‐carboxylic acid (**1**).

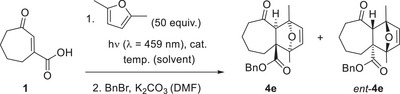						
Entry^a)^	Cat.	Temp. [°C]	Solvent	Time [h]	Yield^b)^ [%]	**4e** */ent‐* **4e** ^c)^
1	**7**	r.t.	CH_2_Cl_2_	21	33	55/45
2	**7**	−20	CH_2_Cl_2_	19	40	61/39
3	**7**	−40	CH_2_Cl_2_	23	33	58/42
4^d)^	**7**	−20	CH_2_Cl_2_	19	33	58/42
5	**7**	−20	DCE	21	53	56/44
6	**7**	−20	PhCl	19	42	55/45
7	**8a**	−20	CH_2_Cl_2_	19	83	60/40
8	**8b**	−20	CH_2_Cl_2_	17	19	53/47
9	**8c**	−20	CH_2_Cl_2_	17	34	60/40
10	**9**	−20	CH_2_Cl_2_	16	45	41/59

^a)^ Optimization experiments were conducted on a 100 µmol scale under the indicated conditions with 10 mol% catalyst in a previously described set‐up.^[^ ^60^ ^]^ Irradiation was performed with an LED displaying an emission maximum at *λ* = 459 nm. (See Supporting Information for additional information).

^b)^ Yield of isolated product after chromatography.

^c)^ The ratio **4e** */ent‐* **4e** was determined by HPLC on a chiral stationary phase.

^d)^ The reaction was conducted with 20 mol% of the catalyst.

**Scheme 6:**
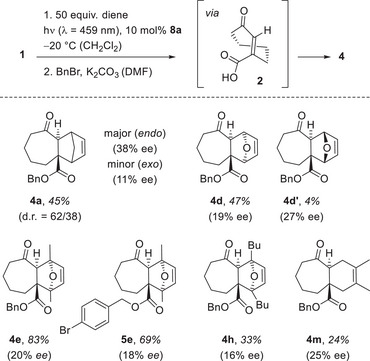
Enantioselective *cis*‐*trans* isomerization of cyclohept‐2‐enone‐3‐carboxylic acid (**1**) mediated by chiral catalyst **8a** and subsequent trapping of enantioenriched *trans*‐isomer **2** by various dienes. The enantiomeric excess (*ee*) was determined after derivatization to the respective benzyl esters **4** by chiral HPLC analysis.

After extensive futile experiments with other cycloheptenes (see Scheme 3) in an enantioselective sensitized photochemical isomerization, the reaction **1** → **3e** mediated by catalyst **8a** (entry 7) was a major breakthrough. It validated the so far unproven hypothesis that triplet energy transfer is a suitable vehicle to drive the reactions toward a single enantiomer. It allowed for a quantitative photochemical isomerization as opposed to previous work employing singlet energy transfer. Although the enantiomeric excess was low (20% *ee*), it seemed warranted to verify with other dienes the generality of the approach (Scheme 6). The experimental approach included the cascade reaction to compound **3** followed by benzylation of the crude mixtures. Yields and enantioselectivities were determined for benzyl esters **4**, with the former depending to some extent on the success of the benzylation reaction. 1,3‐Cyclopentadiene gave product **4a** as an inseparable *endo*/*exo* mixture with a comparably high enantioselectivity for the *endo* product *endo*‐**4a** (38% *ee*). The enantioselectivity of the furan cycloaddition reactions (products **4d**, **4d’**, **4e**, **5e**, **4h**) varied around the 20% *ee* previously recorded for product **4e**. The final example with 2,3‐dimethylbuta‐1,3‐diene proceeded smoothly (product **4** **m**, 25% *ee*) but suffered from lower yields in the benzylation reaction.

A few additional experiments were performed to investigate the nature of the catalysis (see the Supporting Information for details). With the benzyl ester of cyclohept‐2‐enone‐3‐carboxylic acid and 2,5‐dimethylfuran as the substrate, product *rac*‐**4e** was obtained upon direct irradiation at *λ* = 366 nm (91% yield). However, a reaction of the same substrate under the optimized conditions of the sensitized irradiation (10 mol% **8a**) did not lead to any product, indicating the importance of the binding interaction exerted by the carboxylic acid but not by its ester. In the same vein, the reaction of cyclohept‐2‐enone‐3‐carboxylic acid (**1**) and 2,5‐dimethylfuran proceeded cleanly upon direct irradiation in the presence of chiral phosphoric acid 3,3′‐bis(2,4,6‐triisopropylphenyl)‐1,1′‐binaphthyl‐2,2′‐diylhydrogenphosphat (TRIP, 50 mol%). However, upon benzylation, only the racemic Diels–Alder product *rac*‐**4e** was isolated (83% yield), emphasizing the role of the pendant thioxanthone in catalyst **8a**.

While these experiments clearly supported the importance of both coordination and sensitization for a successful reaction, we felt that a deeper understanding of the photochemical isomerization could only be obtained by having a closer look at its progress by computational chemistry.

*Computational results: General considerations and cyclohept‐2‐enones as model substrates*. In order to maximize precision and to minimize computation time, we initially performed a more elaborate study with cyclohept‐2‐enone (CHp) as a model compound before turning toward cyclohept‐2‐enone‐3‐carboxylic acid (**1**) in the chiral setting of phosphoric acid **8a**. The goal of the computational study was to develop a comprehensive model of the reaction cascade. We focused on the high‐yielding, *endo*‐selective reaction **1** → **3e** mediated by catalyst **8a** to rationalize the experimentally observed enantioselectivity. While the photoinduced *cis*/*trans* isomerization of alkenes is well understood,^[^ ^61^ ^]^ ring strain and the presence of catalyst **8a** increase the complexity of the reaction. We pursued a reductionist approach:

- Building on the experimental evidence (Scheme 4), the total reaction coordinate for the transformation of cycloheptene **1** to product **3e** was decomposed into an excited state coordinate (**1** to **2**/*ent*‐**2**) and a ground state (S_0_) coordinate (**2**/*ent*‐**2** to **3e**/*ent*‐**3e**), both of which were treated separately.
- Comparing the bond topologies of **1**, **2**/*ent*‐**2**, and **3e**/*ent*‐**3e**, we were able to partition the net enantioselectivity into three consecutive contributions: (i) The excited state dynamics, where two chiral depletion channels (one‐bond flip [OBF]) compete directly. (ii) Non‐equilibrium ground state dynamics after excited state depletion: Population, in separate enantiomeric channels, can branch between productive conversion into the *trans*‐configured photoproduct **2**/*ent‐* **2** or return non‐productively to **1** with a *cis*‐configuration. (iii) Equilibrium ground state dynamics (Diels–Alder reaction), describing the productive conversion into product **3e** or back‐isomerization into **1**. Once formed, *trans*‐configured species are non‐interconverting, such that the relative effect of the factor (i) is expected to be larger than that of (ii) and (iii). These factors can be addressed separately, and results are presented along the order of elementary steps from **1** to **3e**.
- To keep the computational cost tractable, we commenced with a high‐accuracy, unconstrained investigation of a non‐catalyzed subsystem reaction, revealing dominant reaction pathways and identifying critical parameters modulating enantioselectivity. Subsequently, we used this knowledge to increase the system size while narrowing the full system's degrees of freedom to the essential stages only.

**Scheme 7:**
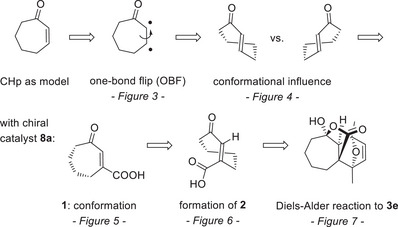
Workflow of the quantum chemical studies: Cyclohept‐2‐enone (CHp) served as a model system to visualize the motion of substrate **1** in the complex with chiral catalyst **8a**.

**Figure 3:**
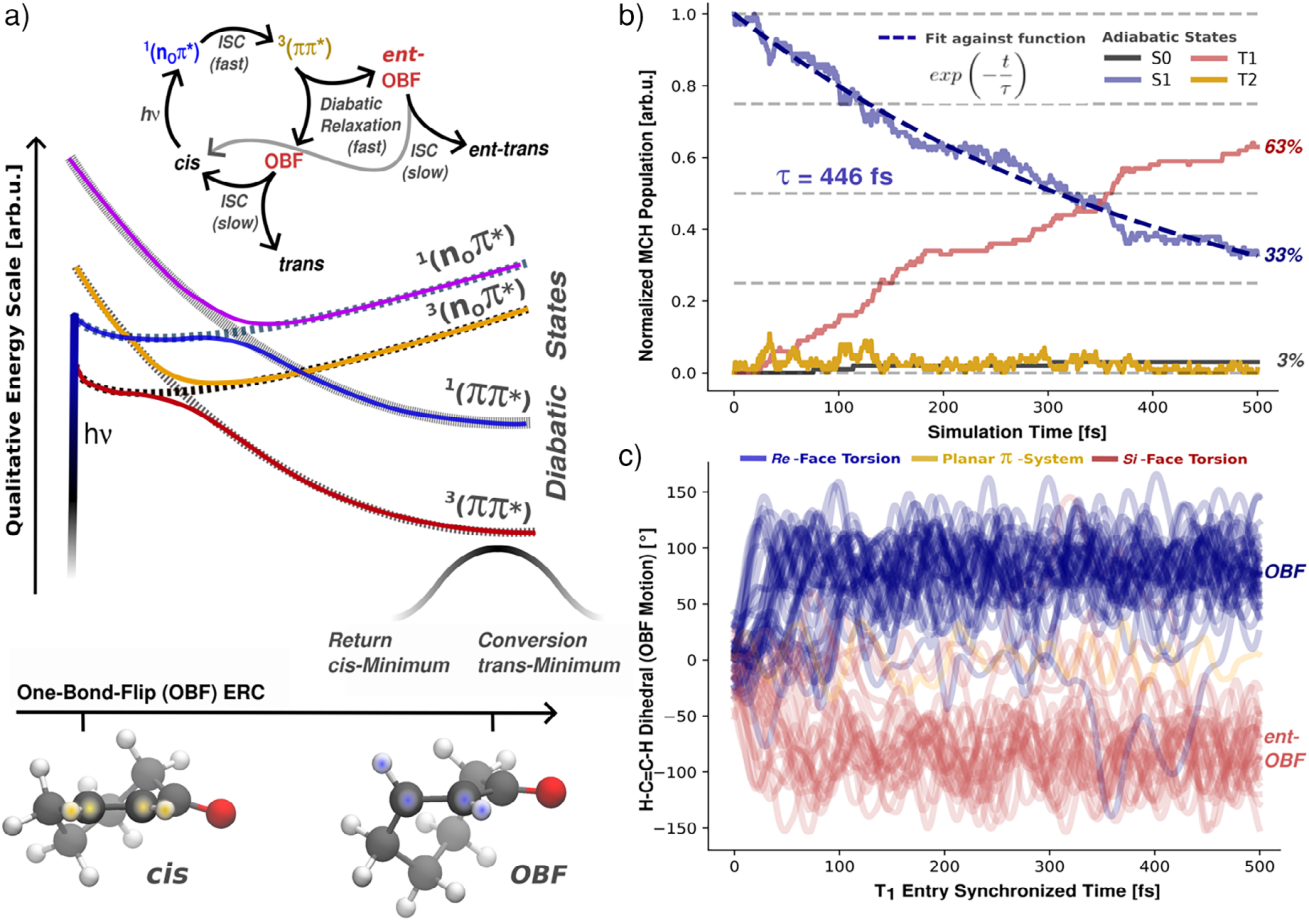
Results of the TSH simulations of the model system CHp. a) Sketch of the excited states T_1_, S_1_, T_2_, and S_2_ (solid‐colored lines) and respective diabatic state character (dashed lines) along the ERC of OBF motion toward the dominant enantiomer in OBF‐configuration. The scheme (top) illustrates the overall photoreaction cycle, which produces either the *trans* photoproduct or its enantiomer (*ent‐trans*). Colored dots in the molecular structure highlight the H‐C=C‐H dihedral angle used to characterize the OBF motion. b) Population dynamics of TSH simulations from the initially populated S_1_ state together with a global exponential fit of the dynamics. The timescale τ characterizes the ISC process toward a long‐lived triplet species on T_1_ via a transient, short‐lived T_2_ population. Population dynamics was averaged over an ensemble of 100 trajectories; all trajectories that violated various energy conservation criteria have been discarded in the analysis (for technical details, see Supporting Information). c) Evolution of the OBF motion, characterized via the H‐C=C‐H dihedral angle as a geometric parameter. Shown are the 66 trajectories populating the T_1_ state within 500 fs. The time evolution of OBF motion is synchronized with respect to entry into the T_1_ state. Trajectories were extended for an additional 500 fs after population of the T_1_. The color scheme characterizes each individual trajectory according to the sign of the dihedral at the end of the simulation time. Interconversion between the faces was a rare event (two instances) and short‐lived.

**Figure 4:**
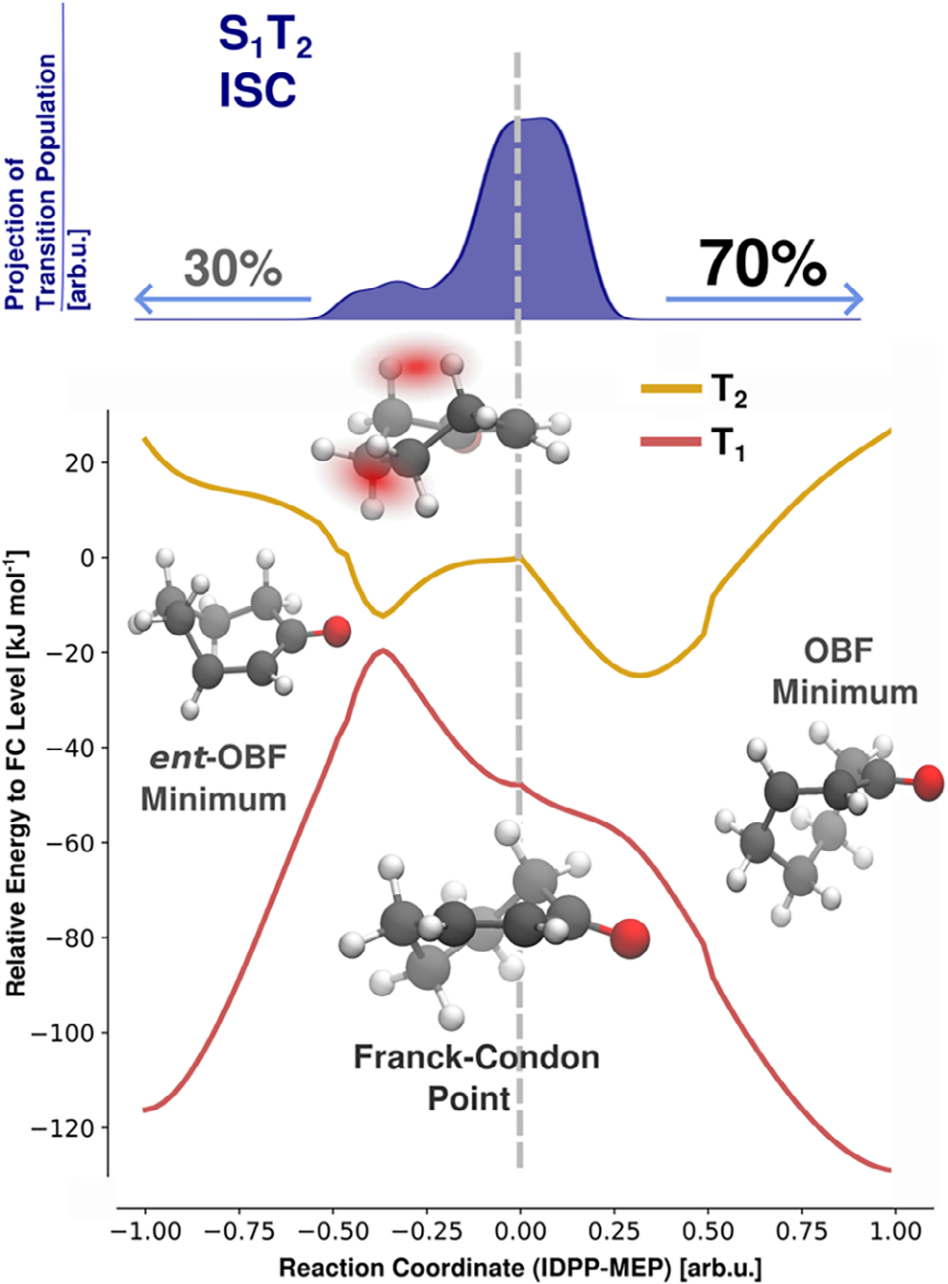
Fate of the chiral conformer CHp‐2 formed by ISC during the surface hopping trajectories dynamics of cyclohept‐2‐enone (CHp). Top: Gaussian‐broadened distribution of geometries at the time point of the S_1_‐T_2_ surface hop, projected onto a vector capturing the essential motions along the *ent*‐OBF/OBF reaction coordinate (see Supporting Information). The two maxima correspond to the position of the S_1_T_2_ crossing seam on either side. Bottom: Excited state energy landscape along an IDPP‐MEP reaction coordinate from the FC geometry to either the **OBF** or *ent*‐**OBF** T_1_ minima. Adiabatic surfaces T_1_ (red) and T_2_ (yellow) are evaluated at the XMS‐CASPT2 level of theory. The red shading of methylene groups (molecular inlay) highlights intramolecular repulsion between the reorganizing aliphatic methylene groups.

**Figure 5:**
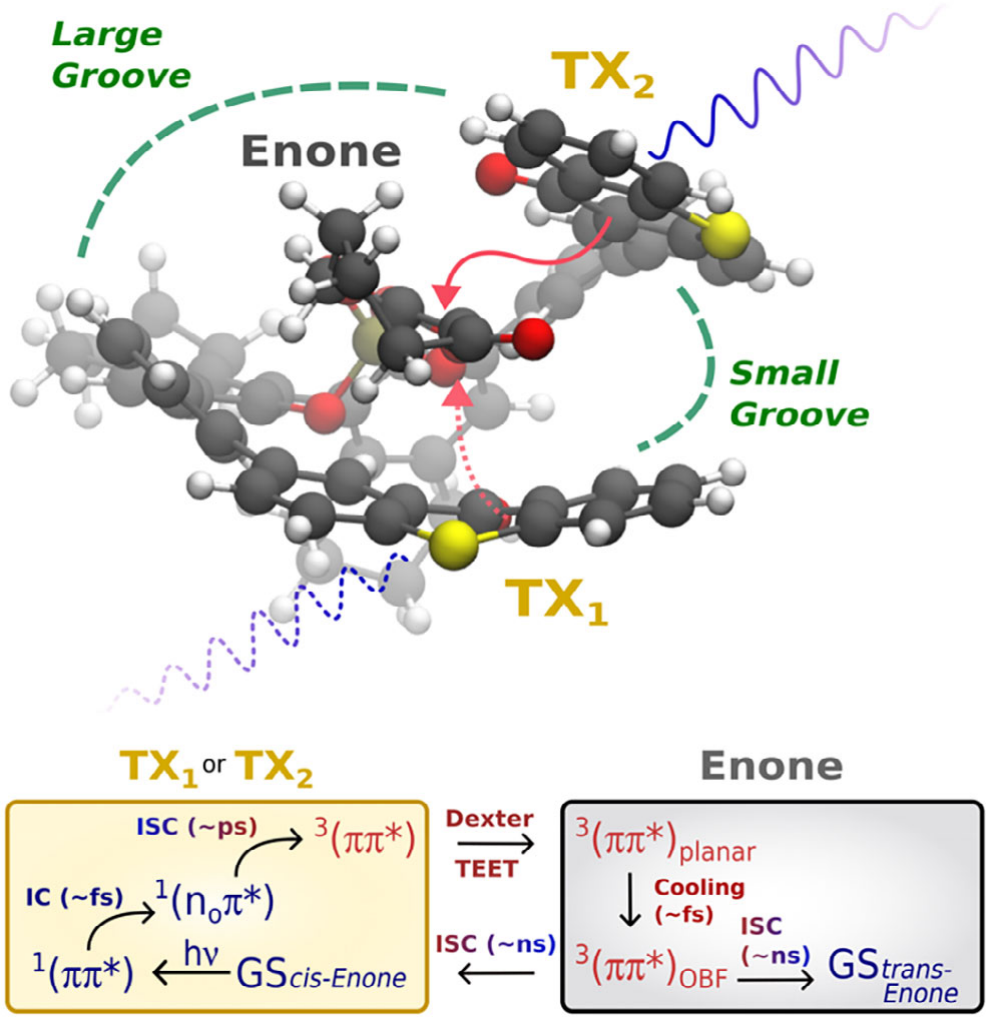
Model for excitation energy relaxation within the complex of cyclohept‐2‐enone‐3‐carboxylic acid (**1**) and catalyst **8a**. Top: Lowest free energy conformation of the substrate‐catalyst complex, depicted at a perspective looking into the binding pocket of the sandwich motif. The asymmetric confinement forces compound **1** to adopt a chiral conformation, which predetermines the direction of the OBF. Bottom: Proposed relaxation model, starting from an initially excited state (indicated by the blue wave) on either the TX_1_ or TX_2_ sides of catalyst **8a** and subsequent triplet excitation energy transfer ([TEET], indicated by the red arrows) into the reactive triplet state of the enone fragment.

**Figure 6:**
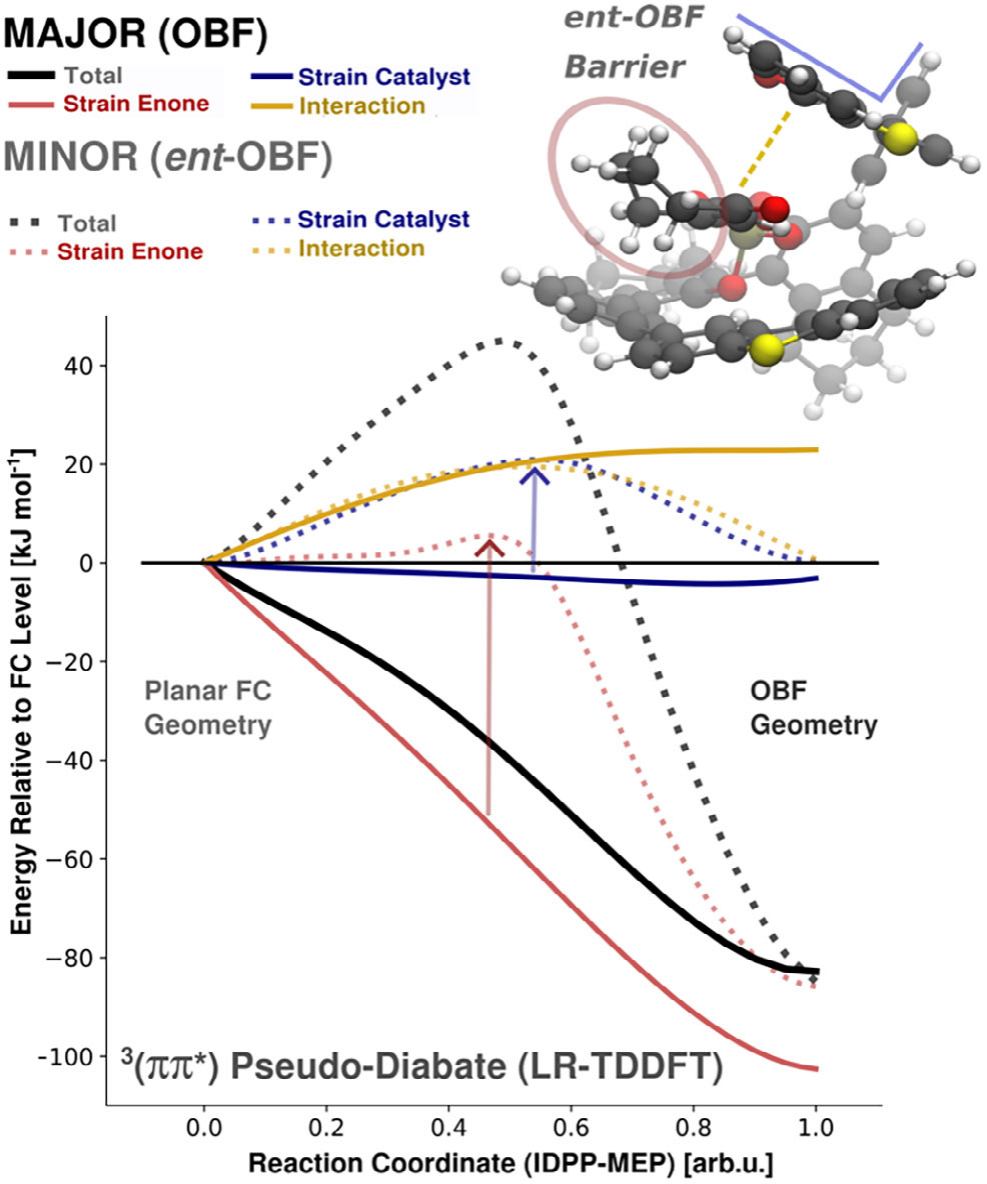
Excited state distortion‐interaction energy decomposition analysis of the dominant chiral conformer of the **1**·**8a** complex along the IDPP‐MEP reaction coordinate linking the FC geometry (planar enone configuration) with the related enone‐**OBF** and enone‐*ent*‐**OBF** configurations. In the top right corner, the *ent*‐**OBF** geometry with the highest barrier at the center of the pathway is shown. Total (black) denotes the total energy, strain enone (red) denotes repulsive contributions due to Pauli repulsion, strain catalyst (blue) denotes destabilization due to deconjugation of the π‐system, and interaction (yellow) denotes van der Waals interactions in the complex.

**Figure 7:**
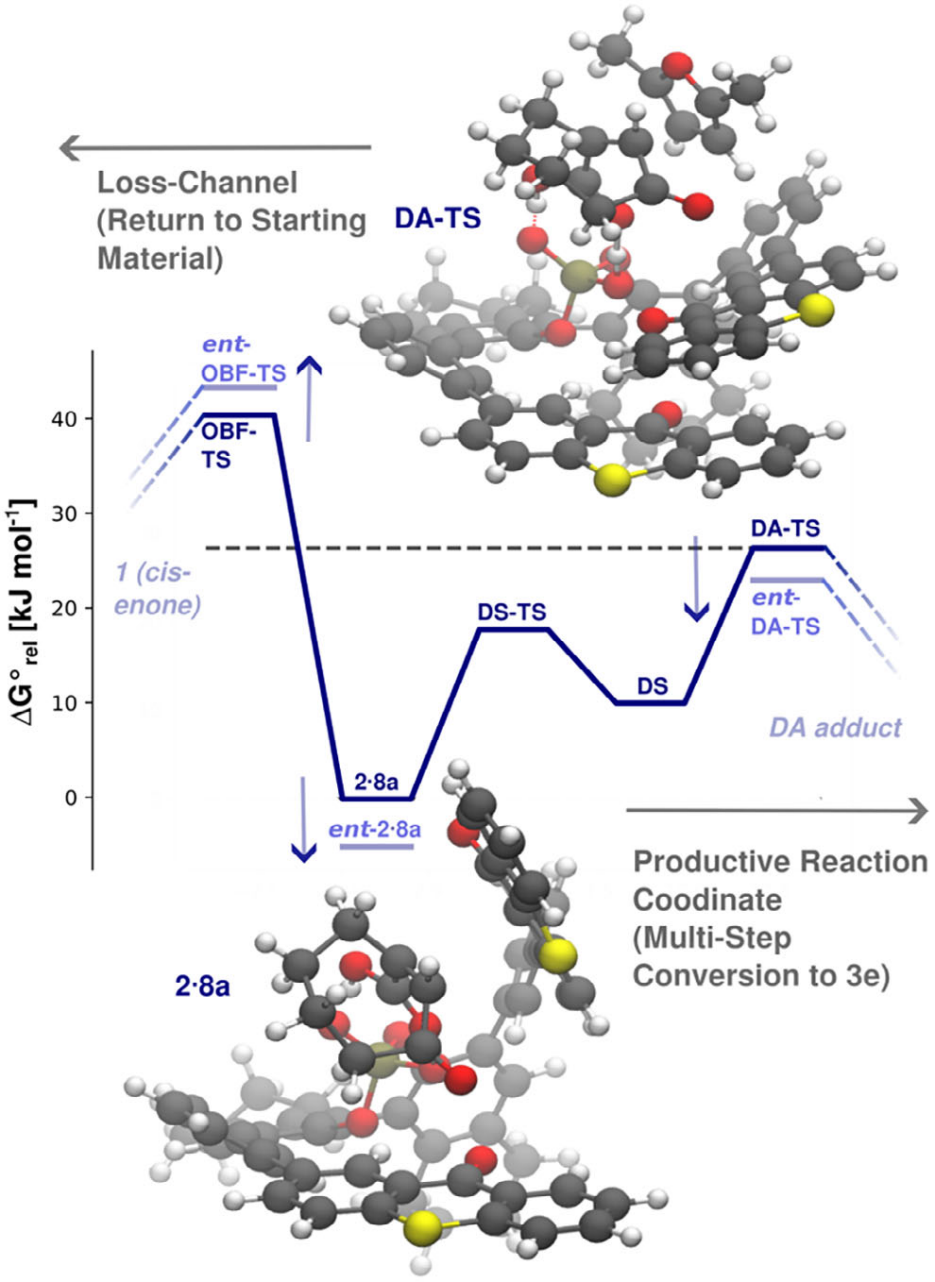
Computed ground state reaction pathways relative to the photochemically generated *trans*‐isomers **2**/*ent*‐**2** with 2,3‐dimethylfuran as the reaction partner. The productive reaction coordinate describes the Diels–Alder (DA) reaction to product **3e**, whilst the loss channel describes the back‐isomerization to **1**. The energy diagram presents the critical section along the reaction coordinates for the catalyzed reaction. The catalyst **8a** adds complexity due to the interconversion between a shielded conformation **2·8a** and a de‐shielded (DS) conformation as a local minimum; the main barrier is the **DA‐TS**. Comparing the major (dark blue) with the minor enantiomers (faint blue), a weak ground state selectivity is suggested that opposes the photochemical selectivity.

The presentation of computational results (Scheme 7) starts by presenting explicit dynamic simulations on the photoisomerization of the CHp model compound. The dynamic simulations reveal the interdependence of the dynamics along an OBF reaction coordinate and the alkyl backbone (Figures 3 and 4). This is followed by an investigation of the photoreaction of compound **1** in the presence of catalyst **8a**, rationalizing the influence exerted by hydrogen bonding (Figures 5 and 6). Finally, the catalyzed Diels–Alder ground state reaction leading to **3e** is discussed (Figure 7) and compared to the uncatalyzed reaction.

*Photochemical reaction dynamics of cyclohept‐2‐enone (CHp)*. In the following, we aim to construct a model which explains the selectivity (i) from a static perspective. As we will demonstrate, this objective is met by a conformer‐specific projection of the photo‐reactive ^3^(ππ*) state along an essential motion twisting the C═C double bond (OBF reaction coordinate, cf. Scheme 7). We start by introducing the electronic structure of cycloalk‐2‐enones. CHp was chosen as the model system for **1** due to the smaller π‐system allowing for a significant reduction of computational cost. As detailed in the Supporting Information, CHp is able to recover the relevant aspects of the electronic structure and conformational distribution of **1**. The latter is relevant as we will show that interactions between the chromophore and the alkyl fragment induce *conformer‐specific* asymmetry.

Cycloalk‐2‐enones display a complex photoreactivity, which is strongly influenced by ring size and substituent effects.^[^ ^62^, ^63^, ^64^, ^65^, ^66^ ^]^ Yet, the electronic structure in the Franck–Condon (FC) region of the *cis*‐configuration remains largely unaltered (Figure 3a).^[^ ^67^, ^68^, ^69^ ^]^ Highly accurate, wave function‐based XMS‐CASPT2/cc‐pVTZ calculations showed that the lowest singlet excited state (S_1_) is always spectroscopically dark and has ^1^(n_O_π*) character, followed by a bright ^1^(ππ*) state (S_2_) (see Supporting Information for the choices of active spaces).^[^ ^70^, ^71^ ^]^ Higher‐lying states are of ^1^(ππ*) character with significant contributions from double excitations. The energetically lower‐lying triplet excited states (T*_n_*) show the same ordering [^3^(n_O_π*) followed by ^3^(ππ*)]. For the following discussion, the nuclear motion in the excited states is expressed through *essential reaction coordinates* ([ERCs]; see the Supporting Information for a detailed description). While a Norrish type I cleavage pathway^[^ ^69^ ^]^ is not observed for CHp, torsion about the C═C bond leads toward two chiral one‐bond flip (**OBF**/*ent*‐**OBF**) channels (OBF‐ERC). They are responsible for *cis‐trans*‐isomerization reactions in flexible cycloalk‐2‐enones and were investigated in closer detail for both CHp and **1**:

Figure 3a illustrates the excited‐state reaction scheme along the OBF coordinate. Starting in the *cis‐*configuration of CHp (left), the bright state S_2_ has ^1^(ππ*) character, and the S_1_ state has ^1^(n_O_π*) character. Two triplet states, T_1_ and T_2_ (^3^(n_O_π*) and ^3^(ππ*) character, respectively), are found close to S_1_. Elongation of the C═O bond leads to local minima of the S_1_ and T_1_ states of ^1^(n_O_π*) and ^3^(n_O_π*) character. Close by, interaction among S_1_ and T_2_ states forms an energetically accessible intersection, suggesting the importance of intersystem crossing (ISC). According to El‐Sayed's rules, transitions between S_1_^1^(n_O_π*) and T_2_^3^(ππ*) are allowed, which can subsequently lead to relaxation dynamics within the triplet manifold. In the OBF‐configuration (right), minima of ^1^(ππ*) and ^3^(ππ*) states are found at low energies.

The role of vibrational modes in guiding the excited state relaxation cascade along these paths is essential, necessitating explicit dynamical treatment. Trajectory surface hopping (TSH) simulations allow to follow the nonadiabatic excited state dynamics in full coordinate space while retaining some quantum aspects, like the branching of population between electronic states. We performed TSH dynamics within the SHARC program suite on the XMS‐CASPT2 level of theory using a cc‐pVDZ basis set. Validation of the basis was performed via comparison of the energetics of stationary points to the superior cc‐pVTZ basis set and via a systematic basis set expansion in static simulations to the aug‐cc‐pV5Z basis (a detailed description of the workflow and a validation of the basis set is provided in the Supporting Information).^[^ ^72^ ^]^ The simulations treated the full conformer space of CHp within a *chiral* standard orientation chosen to match the chiral frame within the constraints of catalyst **8a**. The dynamics simulations were expected to allow insight into two questions: First, we needed to track electronic relaxation to discern whether the singlet or triplet manifold is the decisive relaxation channel (see Supporting Information). Second, we sought to quantify the competition of excited state depletion to S_0_ and **OBF**/*ent*‐**OBF** channel interconversion. With this, we discerned if either the initial branching of population or the Boltzmann distribution of the OBF‐conformers is relevant for estimating the enantioselectivity from the excited state dynamics [cf. factor (i)]. An ensemble of 106 independent trajectories was simulated of which 100 trajectories fulfilled all energy conservation criteria during the propagation time of 500 fs and were further employed for data analysis. Initial conditions were obtained from sampling a Wigner distribution using a restricted excitation window (*λ* ≥ 300 nm) that reflects direct excitation conditions (cf. Scheme 4). Under these conditions, a probabilistic assignment of the initial electronic state via transition dipole moments yielded the exclusive population of the S_1_ state with ^1^(n_O_π*) character (see the Supporting Information for technical details). Transferred to the catalytic experimental conditions (cf. Scheme 6), where the reaction dynamics are presumably initiated by triplet energy transfer from the catalyst (see below), we consider initiating the dynamics in S_1_ more realistic than initiating the dynamics in the S_2_ state, which would involve substantial vibrational excess energy. Electronic relaxation commences immediately after initiation, as is evident from the evolution of state populations (Figure 3b). The S_1_ state shows, after population, an exponential decay on the few hundred femtosecond timescale. The decay of S_1_ is associated with the population of the T_2_ state, which almost immediately relaxes into the lowest triplet T_1_ state. The lifetime of the S_1_ state was determined to be 446 fs (exponential fit), which is slightly faster than the previously reported value of 746 fs for cyclohexen‐2‐enone.^[^ ^68^ ^]^ OBF‐mediated internal conversion (IC) into S_0_ is observed as a minor pathway (3%). Instead, ISC within the planar configuration space accounts for the majority of the observed S_1_ population decay (IC:ISC = 5:95). The acceptor state in the triplet manifold was almost exclusively T_2_, which quickly decayed into a long‐lived T_1_ as the end point of the excited state relaxation. Accordingly, we focused the attention on the fate of the triplet species. The simulation time for the 66 trajectories undergoing ISC was extended until a stable population of the T_1_ was observed for 500 fs. This set of longer trajectories revealed that the OBF motion was the only active ERC, agreeing with the absence of Norrish type I side products (vide supra). To understand a possible preference toward one enantiomer of *trans*‐CHp, we tracked several well‐defined geometric parameters. In particular, the chiral OBF‐ERCs were monitored through the H─C═C─H dihedral evolution (Figure 3c; see Supporting Information for further details). Upon entry into T_1_, the ensemble underwent rapid and irreversible differentiation into the **OBF** and *ent*‐**OBF** channels (timescale 10 fs). This branching was observed to be asymmetric, favoring the **OBF** channel over the *ent*‐**OBF** channel. In addition, the aliphatic reorganization was monitored through inversions of its dihedrals. While the conformer distribution of the S_0_ was maintained throughout S_1_ occupancy, it decayed toward the Boltzmann distribution of the OBF‐space upon population of T_1_ with an 80 fs delay on a timescale of 100 fs (see Supporting Information). The moderate timescale separation suggests an interaction between the chromophore and aliphatic fragments, investigated in the following.

Combining the observations of a very short‐lived T_2_ and immediate activation of the OBF‐ERCs after population of T_1_, the system dynamics follow the diabatic character of the ^3^(ππ*) hypersurface (Figure 3a). As such, we suggest that the *early* branching on the ^3^(ππ*) surface captures the excited state enantioselectivity. For the vibrationally hot **OBF** and *ent*‐**OBF** channels, cross‐channel interconversion was rare and the initially formed enantiomer ratio remarkably stable (Figure 3c). We thus assume that the initial T_1_‐branching ratio is upheld on the longer timescales of excited state depletion.^[^ ^73^ ^]^ Intriguingly, the delay between the chromophore and response of the aliphatic skeleton indicates that the excited state dynamics may differ for each conformer. Indeed, we found large alterations for their intrinsic enantiomeric ratios (see Supporting Information).

A closer investigation was conducted for the most selective chiral conformer [CHp‐2, **OBF**/*ent*‐**OBF** = 70:30, corresponding to 40% *ee*], which is also dominant upon binding to the catalyst (vide infra). The critical pathway, interpolating from the S_0_‐FC to both T_1_‐**OBF** and T_1_‐***ent*‐OBF** minima, was investigated along a reaction coordinate described as an image‐dependent pair potential minimum energy path (IDPP‐MEP)^[^ ^74^ ^]^ (Figure 4; see Supporting Information for details).

Along the reaction coordinate, the surfaces of the T_1_, T_2_, and S_1_ states (S_1_ not shown) were evaluated at the XMS‐CASPT2 level of theory (see Supporting Information for a detailed description).^[^ ^75^, ^76^, ^77^ ^]^ We found that the T_1_ and T_2_ potential energy surfaces exhibit an asymmetry toward the **OBF** channel, which induces a biased branching of population via two mechanisms: First, a weak asymmetry on the S_1_ surface shifts the population upon S_1_‐T_2_ transition toward the ISC seam of the **OBF**‐direction (Figure 4, top panel). Nevertheless, such biased preparation does not fully account for the observed ratio, as the majority of transitions occur fairly centered. Second, propagation toward the **OBF** minimum is favored via a steep gradient. The fraction of the population that moves toward this direction is rapidly steered away from the *cis*‐configuration of the FC point and trapped in the **OBF** minimum (Figure 4, lower panel). In contrast, a barrier induces a slower evolution toward the *ent‐* **OBF**‐ERC, suggesting that the **OBF**/*ent*‐**OBF** asymmetry is imposed by conformational constraints.

Overall, we deciphered the necessary conditions to explain the photochemical enantioselectivity in CHp: A time scale separation between the chromophore and alkyl reorganization is just close enough to prevent equilibration between the enantiomeric channels, such that the initial branching becomes decisive. It is, thus, sufficient to study the ground state conformer distribution and the properties of the ^3^(ππ*) state toward the early‐stage OBF‐ERCs to explain the excited state selectivity. The model CHp and compound **1** vary by the COOH substitution of the enone fragment (cf. Scheme 7). Electronic structure simulations of excited states (see Supporting Information) allow to quantify the influence of the COOH group on the topology of the ERC and thus assess the influence on the initial reaction dynamics. We find that along the initial OBF reaction coordinate, the minima are preserved, but the OBF minima of **1** are slightly more stabilized. Also, the energy gap between the ^1^(ππ*) minimum with OBF‐configuration and associated S_0_/S_1_ crossing seams is widened, which suggests that nonradiative relaxation to the ground state is reduced. Both findings support the notion that cross‐channel interconversion of the competing chiral OBF channels, found to be rare for the CHp model system, should be even more reduced in compound **1**. The model system CHp thus provides valuable insight into the early steps of the catalyzed photoreaction to be applied to a more complex substrate.

*Computational results: Isomerization of cyclohept‐2‐enone‐3‐carboxylic acid (* ***1*** *) in the chiral pocket*. In this section, we analyze the influence of the catalyst on the relaxation cascade toward an asymmetric photoproduct distribution, considering substrate **1**. The results on the model system CHp suggest the following important conditions for a high enantiomeric ratio of the **2**/*ent‐* **2** intermediate: First, it is necessary to generate an asymmetric conformer distribution in the S_0_. Yet, this is not sufficient for a satisfactory performance of the overall photoreaction, as a vibrationally hot ensemble could, in principle, undergo a random walk on the photoreactive surface. Thus, either vibrational cooling is required preceding the population transfer onto the enone or enhanced asymmetry along the enone's photoreactive state. To describe the complex interactions between **1** and catalyst **8a**, we follow an efficient yet accurate protocol to narrow the large conformer space. A previous study by Yepes et al. validated a combination of metadynamics sampling, ensemble optimization, and Boltzmann weight refinement for a *C*_2_‐symmetric, chiral catalyst with similarities to **8a**.^[^ ^78^, ^79^, ^80^ ^]^ Extending beyond the work of Yepes et al., we conceived an iterative, dynamically constrained refinement of the initially obtained Boltzmann sum that succeeds in removing intruder conformers from the prescreening on the lower level of theory (see Supporting Information for a detailed description). In a similar manner to Bickelhaupt and Houk, we extended the IDPP‐MEP reaction coordinate approach by a distortion‐interaction energy decomposition analysis of the excited state that provides an intuitive explanation of the influence of the catalyst.^[^ ^81^ ^]^ Comparing both the ground and excited state perspectives, our results suggest a crucial role of the flexibility of the linker segments in **8a**.

Calculations on the **1**·**8a** complex showed the clear dominance of a specific binding motif (termed the sandwich motif, top panel in Figure 5), which accounts for about 96% of the Boltzmann sum. In this motif, the substrate is bound to the phosphoric acid moiety via two hydrogen bonds. An asymmetric catalyst interaction is achieved if at least three binding anchors between the catalyst and the substrate are provided, and the hydrogen bonding interactions account for two. The third anchor is introduced by a key feature of the catalyst: The *C*_2_‐symmetry of **8a** is broken by an inward tilt of the TX_1_‐phenyl‐linker, which allows for the thioxanthone groups (TX_1_ and TX_2_) to tightly fold around the binding site. Both thioxanthone groups adopt a relative perpendicular orientation, in turn generating a small and a large groove. Upon capture of **1**, the TX groups simultaneously engage in stabilizing π‐π interactions with the enone moiety within the small groove (enone‐TX_n_‐distance: ∼0.35 nm), while forcing the alkyl fragment to bend into the large groove. This not only induces a chiral conformer distribution but also narrows it. Only for the CHp‐2‐like conformation of **1**, predicted to show an intrinsic selectivity, steric repulsion with the TX arms is minimized, and its relative Boltzmann weight increases substantially (from 56% in **1** to 94% in **1**·**8a**). Electronic excitation is spatially confined to either of the TX sites (see Supporting Information for natural transition orbital analysis). At the double‐hybrid density functional level of theory (SCS‐RI‐ωPBEPP86), we found two nearly degenerate transitions to S_1_ (359 nm, TX_1_ site) and S_2_ (357 nm, TX_2_ site), both of which are spectroscopically accessible. Their local orbital symmetry resembles the optically allowed excited state of thioxanthone (TX). Previous studies on TX outlined an initial relaxation cascade (Figure 5, bottom right): Following excitation, a fast two‐step process yields the lowest triplet state, likely via initial IC toward ^1^(n_O_π*) (fs timescale) followed by ISC (ps timescale).^[^ ^82^ ^]^ Similar timescales are expected for the complex **1**·**8a**. Accordingly, we assume that the excitation energy is transferred to the enone chromophore via short‐range triplet energy transfer.^[^ ^83^, ^84^ ^]^ Such a mechanism is consistent with the loss of conversion upon *O*‐benzylation of **1** (vide supra) and highlights the catalytic efficacy of the sandwich motif by bringing the donor and acceptor sites in close proximity. Since triplet energy transfer is an exchange‐mediated process depending on orbital overlap, a transition is most likely to occur into the ^3^(ππ*) state, preparing the substrate chromophore for photoisomerization. The potential surface of the full system (Figure 6) exhibits strong asymmetry between the enantiomeric channels, even more pronounced than observed for the CHp model (cf. Figure 4).

The favored enantiomer shows barrier‐less relaxation toward the OBF channel, in agreement with the experimental outcome. Such selectivity is found to be a strain‐mediated effect: Along the early OBF‐ERCs (∼ 0.4 Reaction Coordinate), the TX_n_ groups undergo a synchronous torsional motion about the C_linker_─C_TX_ bond, maintaining π─π interactions. Hence, the interaction energies vary synchronously. The decomposition analysis suggests that the strongest contribution to the *ent*‐**OBF** barrier stems from the enone fragment, and it is caused by the reorganization of the alkyl fragment. A minor contribution to the barrier further stems from a rearrangement of the catalyst. Rotation onto the **OBF** face allows the alkyl backbone of **1** to escape confinement by further bending into the large groove. In contrast, rotation onto the *ent*‐**OBF** face induces a complete de‐conjugation of the phenyl and TX_2_ π‐systems, as the latter evades the approaching, bulky alkyl fragment. These findings allow to rationalize the experimentally observed outcome: The origin of the systems' excited‐state selectivity is the formation of the sandwich binding motif, which forces an asymmetric conformer space.

*Computational results: Thermal Diels–Alder reaction inside the chiral pocket*. What remained to be determined were selectivity effects of the equilibrium ground state dynamics [cf. factor (iii)]. In a first step, we ruled out that lactonization to **3e** occurs before the Diels–Alder reaction (see the Supporting Information for details). Rather, the lactonization is an ensuing step without any influence on the stereoselectivity. To identify critical reaction barriers for the cycloaddition, we performed a high‐level assay on the achiral ground state reaction network of *trans*‐isomers **2**/*ent‐* **2** (see Supporting Information). The results identify productive reaction coordinates, which afford products **3e**/*ent*‐**3e**, and nonproductive loss channels associated with the back‐isomerization to **1**. It was found that the competition of the **OBF‐TS** (TS = transition state) against the **DA‐TS** is decisive at this stage of the reaction. The cycloaddition proceeds over a thermally accessible barrier and affords **3e**/*ent* **‐3e** in a net exergonic cascade. In the reaction catalyzed by **8a** (Figure 7), the reorganization dynamics of both TX groups increase the complexity of the productive reaction coordinate compared to the uncatalyzed reaction. We identify decisive barriers (**DA‐TS/** *ent*‐**DA‐TS**, **OBF‐TS**/*ent*‐**OBT‐TS**) for both enantiomeric channels, for which the minor *ent*‐**2**·**8a** network appears to be stabilized along the productive coordinate and destabilized along the loss channel.

While the calculated free energy differences of the barriers should be considered with some care, the findings suggest that the equilibrium ground state selectivity [cf. (iii)] is opposed to the photochemical selectivity of the reaction [cf. (i)]. This could explain the erratic trend observed for varying diene reaction partners (Scheme 4) and the relatively low overall enantioselectivity. The final product, **3e**/*ent*‐**3e**, is sterically bulky and thus interacts poorly with the catalyst, such that it is readily exchanged for **1**. The event closes the catalytic cycle and guarantees a reasonable turnover. The prevalence of **3e** over *ent*‐**3e** is a result of the excited state process.

## Conclusion

In summary, we have shown for the first time that a *cis*‐cycloalk‐2‐enone can be enantioselectively converted into its chiral *trans*‐isomer. In a fundamental study employing *cis*‐cyclohept‐2‐enone‐3‐carboxylic acid (**1**) as the substrate, the *trans*‐isomer was identified by transient IR spectroscopy and trapped in a stereospecific Diels–Alder reaction. Proof of principle was obtained that a chiral thioxanthone with an appropriate binding cavity can induce a notable enantioselectivity. A suite of computational studies revealed the enantioselective reaction course to be linked to a chiral recognition within the constraints of the catalyst‐cycloalkenone assembly. The *cis*‐isomer **1** is bound in a chiral conformation, which then translates upon energy transfer via an OBF to the chiral *trans*‐isomer **2**. The computational results clearly associate the rotation around the former double bond with the first excited ^3^(ππ*) triplet state of the enone and provide a clear picture of how the *trans*‐isomer is formed. Since the reorganization of the aliphatic backbone is slower than the bond flip, the chirality transfer is high. The findings allowed access to the enantioselectivity of the process from the binding properties of various conformers of substrate **1** to the chiral phosphoric acid **8a** with two pendant thioxanthone groups. Triplet energy transfer was shown to be possible for a preferred conformation and triggers the consecutive reactions. The model so obtained (cf. Figure 6) can serve as guidance for the design of future photoisomerization mediated by chiral phosphoric acids. When studying the further course of the *trans*‐isomers **2** and *ent*‐**2** in the Diels–Alder reaction, it became evident that the chosen catalyst **8a** favors a preferred cycloaddition to the minor enantiomer *ent*‐**2**. In other words, the enantioselectivity achieved in the photoisomerization erodes in the Diels–Alder reaction depending on which diene traps the chiral *trans*‐isomer and which approach the diene takes (*endo* vs *exo*). This hypothesis is supported by the fact that the enantioselectivity varied between 16% and 38% *ee* for different dienes. *Vice versa*, it is safe to assume that the enantioselectivity obtained in the photoisomerization is at least 38% *ee*, which in turn provides an excellent reference point for further work in the area.

## Conflict of Interests

The authors declare no conflict of interest.

## Supporting information



Supporting Information

Supporting Information

Supporting Information

Supporting Information

Supporting Information

## Data Availability

The data that supports the findings of this study are available in the supplementary material of this article. Primary research data are openly available in the repository RADAR4Chem at https://doi.org/10.22000/2pbrutk9aq6s1t0y.^[^ ^85^ ^]^
