# Supplementary material for: Enantioselective Photochemical Generation of a Short‐Lived, Twisted Cycloheptenone Isomer: Catalytic Formation, Detection, and Consecutive Chemistry
Source: Angew Chem Int Ed Engl. 2025 Apr 21;64(23):e202501433. doi: 10.1002/anie.202501433 (PMC12124355; doi:10.1002/anie.202501433)
Supplement: Supplementary file 1 — Supporting Information [file ANIE-64-e202501433-s001.pdf]

Supporting Information for:

Enantioselective Photochemical Generation of a Short-Lived, Twisted Cycloheptenone Isomer: Catalytic Formation, Detection, and Consecutive Chemistry

Max Stierle,[a] Constantin Jaschke,[b] Daniel J. Grenda,[c] Martin Peschel,[b] Thomas Pickl,[a] Niklas Gessner,[c] Patrick Nuernberger,[c] Benjamin P. Fingerhut,[b*] Christian Ochsenfeld,[b] Regina de Vivie-Riedle[†b] and Thorsten Bach[a*]

[a]Department Chemie and Catalysis Research Center (CRC), School of Natural Sciences
Technische Universität München
D-85747 Garching, Germany

[b]Department of Chemistry
Ludwig-Maximilians-Universität München
D-81377 München, Germany
[†] Deceased on June 20, 2024.

[c]Institut für Physikalische und Theoretische Chemie,
Universität Regensburg
D-93053 Regensburg, Germany

Table of Contents

1. General Information	3
2. Analytical Methods	4
3. Transient Spectroscopy	6
4. Computational Studies	16
5. Substrate Synthesis	43
6. Synthesis of Phosphoric Acid Catalysts	47
7. Racemic Photoinduced *Diels-Alder Reaction*	60
8. Benzylation of Primary Photoproducts and Catalytic Photoinduced *Diels-Alder* Reaction	71
9. Iodolactonization of Compound *rac*-**3e**	80
10. Determination of the Absolute Configuration	81
11. Additional Experiments	86
12. Additional Crystal Structures	87
13. Emission Spectra and Triplet Energy Measurements	94
14. Chiral HPLC Traces	96
15. NMR Spectra of New Compounds	105
15.1 NMR Spectra of Substrates	105
15.2 Synthesis of Phosphoric Acid Catalysts	108
15.3 Primary Photoproducts	118
15.4 Derivatized Photoproducts	131
16. Data Sheets of Light Sources	140
17. References	142

1. General Information

All reactions sensitive to air or moisture were carried out in flame-dried glassware under positive argon pressure using standard *Schlenk* techniques.

Commercially available chemicals were used without further purification unless stated differently.

Dichloromethane (CH_2Cl_2) and diethyl ether (Et_2O) were dried using a MBSP 800 MBraun purification system with the following columns:

Dichloromethane: 2 × MB-KOL-A type 2 (aluminium oxide)

Diethyl ether: 1 × MB-KOL-A type 2 (aluminium oxide), 1 × MB-KOL-M type 2 (3 Å molecular sieves)

The following dry solvents are commercially available and were used without further purification: *N,N*-dimethylformamide (*Acros Organics*, 99.9% extra dry, over molecular sieves), dioxane (*Acros Organics*, 99.5% extra dry, over molecular sieves), pyridine (*Acros Organics*, 99.5% extra dry, over molecular sieves), methanol (*Acros Organics*, 99.8% extra dry, over molecular sieves), chlorobenzene (*Acros Organics*, 99.5% extra dry, over molecular sieves), 1,2-dichloroethane (*Acros Organics*, 99.5% extra dry, over molecular sieves). 1,2-dimethoxyethane (DME) was purchased from *TCI Europe* (>99%). Toluene was used after simple distillation of the technical solvent.

Deuterated solvents were purchased from *Deutero*.

Technical solvents were distilled prior to use for column chromatography [hexane (Hex), pentane (p), dichloromethane, methanol (MeOH), ethyl acetate (EtOAc), diethyl ether (Et_2O), acetone (Ac)]. Flash column chromatography was performed on silica 60 (*Merck*, 230-400 mesh) with the indicated solvent mixtures (v/v). All solvents used in photochemical reactions (CH_2Cl_2, DCE, PhCl) were degassed by four freeze-pump-thaw cycles and stored over 4 Å molecular sieves.

Photochemical reactions under direct irradiation conditions (λ = 366 nm) were performed in flame dried *Duran* tubes (diameter = 1 cm or 4 cm) in a positive geometry setup (cylindrical array of 16 UV-A lamps, 8 W nominal power) with the sample placed in the center of the illumination chamber. [1]

Catalytic reactions were performed using an LED (λ = 459 nm) in a Schlenk tube (diameter = 1 cm) with a polished quartz rod as an optical fiber, which was roughened by sandblasting at one end.[2] The reactions were cooled by using a Huber TC100E immersion cooler with ethanol as coolant. In other cases, a mixture of ice/NaCl was used (−21 °C) to perform reactions at low temperature.

The commercially available dienes spiro[2.4]hepta-4,6-diene, cyclohexadiene, furan, 2,5-dimethylfuran, 2,3-dimethylfuran, 2-methylfuran and 2,3-dimethylbutadiene were freshly distilled under an argon atmosphere prior to use. The commercially available dienes 3-bromofuran and furfurylacetate were used without further purification. Cyclopentadiene was freshly cracked under an argon atmosphere prior to use. The dienes 2,5-diethylfuran, 2,5-dipropylfuran and 2,5-dibutyllfuran were prepared according to a literature procedure[3] and distilled under an argon atmosphere prior to use. 1,2-Dimethylenecyclohexane was prepared according to a literature procedure[4] and distilled under an argon atmosphere prior to use.

The catalyst **7**[5], catalyst precursors (*R*)-3,3'-diiodo-5,5',6,6',7,7',8,8'-octahydro-[1,1'-binaphthalene]-2,2'-diol[6] and (*R*)-6,6'-diiodo-7,7'-bis(methoxymethoxy)-2,2',3,3'-tetrahydro-1,1'-spirobi[indene][7], as well as the brominated thioxanthone derivatives 2-bromo-9*H*-thioxanthen-9-one[5] and 2-(3-bromophenyl)-9*H*-thioxanthen-9-one[5] were prepared according to literature procedures.

2. Analytical Methods

Melting points (M.p.) were determined using a *Kofler* heating bar and are uncorrected.

Thin layer chromatography (TLC) was performed on silica coated glass plates (*Merck*, silica 60 F254) using detection by UV-light (λ = 254 nm) and/or by staining with a potassium permanganate solution [$KMnO_4$] followed by heating.

$KMnO_4$-staining solution: potassium permanganate (3.00 g), potassium carbonate (20.0 g), and 5% aqueous sodium hydroxide solution (5.00 mL) in water (300 mL).

Infrared spectra (IR) were recorded on a Perkin Elmer Frontier IR-FTR spectrometer by ATR technique. The signal intensity is stated using the following abbreviations: s (strong), m (medium), w (weak).

Nuclear Magnetic Resonance Spectra (NMR) were recorded at room temperature either on a Bruker AVHD-300, AVHD-400, AVHD-500, or an AV-III-500 (equipped with a QNP cryo sample head). ^{1}H NMR spectra were calibrated to the residual proton signal of chloroform-d_1 (δ = 7.26 ppm), dimethylsulfoxide-d_6 (δ = 2.50 ppm), or methylene chloride-d_2 (δ = 5.32 ppm). ^{13}C NMR spectra were referenced to the 13C triplet of chloroform-d_1 (δ = 77.16 ppm), to the ^{13}C septet of dimethylsulfoxide-d_6 (δ = 39.5 ppm), or the ^{13}C quintet of methylene chloride-d_2 (δ = 54.0 ppm). ^{31}P NMR spectra were measured without reference. The following abbreviations were used to indicate the multiplicities of a signal: br – broad, s – singlet, d – doublet, t – triplet, q – quartet, m – multiplet.

Assignments of ^{1}H and ^{13}C NMR signals were based on two-dimensional NMR experiments (COSY, HSQC, HMBC, NOESY).

High Resolution Mass Spectra (HRMS) were measured on a *Thermo Scientific* LTQ-FT Ultra (ESI) or a Synapt XS High Resolution MS from *Waters* (ESI).

UV-Vis Spectroscopy was performed on a *Perkin Elmer* Lambda 35 UV-Vis spectrometer using a *Hellma* precision cell (quartz SUPRASIL®) with a pathway of 1 mm.

High Performance Liquid Chromatography (HPLC) was performed using a *Thermo Fisher* Ultimate 3000 device equipped with a chiral stationary phase [ChiralPak AD-H (250 × 4.6 mm) or Chiralcel OJ-RH (150 × 4.6 mm), *Daicel Chemical Industries*] with LPG 3400SD Pump, WPS3000SL Autosampler and a DAD 3000 photodiode array (detection at λ = 215 nm or λ = 210).

Preparative High Performance Liquid Chromatography (HPLC) was performed (*Thermo Fisher,* Ultimate 3000 series, HPG 3200BX Pump, MWD 3000-RS detector [λ = 215 and 254 nm]) using a chiral stationary phase (ChiralPak AD-H).

Specific Rotation was determined using a *Bellingham+Stanley* ADP440+ polarimeter and is reported as follows: $[\alpha]_D^T$ (c in g per 100 mL solvent).

Luminescence Measurements were performed on a Horiba Scientific FluoroMax-4P instrument equipped with a continuous Xe source for steady state measurements and a Xe flashlight source for the observation of phosphorescence spectra. All measurements were performed in quartz tubes (inner diameter = 4 mm) under nitrogen-atmosphere. If necessary, the samples were cooled in a small quartz Dewar vessel with liquid nitrogen (77 K).

3. Transient Spectroscopy

Spectroscopic Materials and Methods

Stationary UV/Vis spectra were recorded in dichloromethane (Fisher Scientific, >= 99.8 %) using a Cary60 spectrometer (Agilent and quartz cuvettes (Starna or Hellma) with an optical path length *l* of 10 mm.

IR spectra, both stationary and time resolved, were recorded using a Vertex 80 FTIR spectrometer (Bruker). Omnicells (Specac) with CaF_2 windows were used as cuvettes and a teflon spacer set the optical pathlength to 200 µm or 500 µm.

Stationary Spectroscopy

The stationary spectrum of compound **1** in dichloromethane is shown in Figure S3.1 for different spectral ranges. The peak around 340 nm allows for excitation using near-UV light. Most relevant for the step-scan measurements (*vide infra*) is the strong carbonyl signal in the IR spectrum around 1700 cm^{-1}.

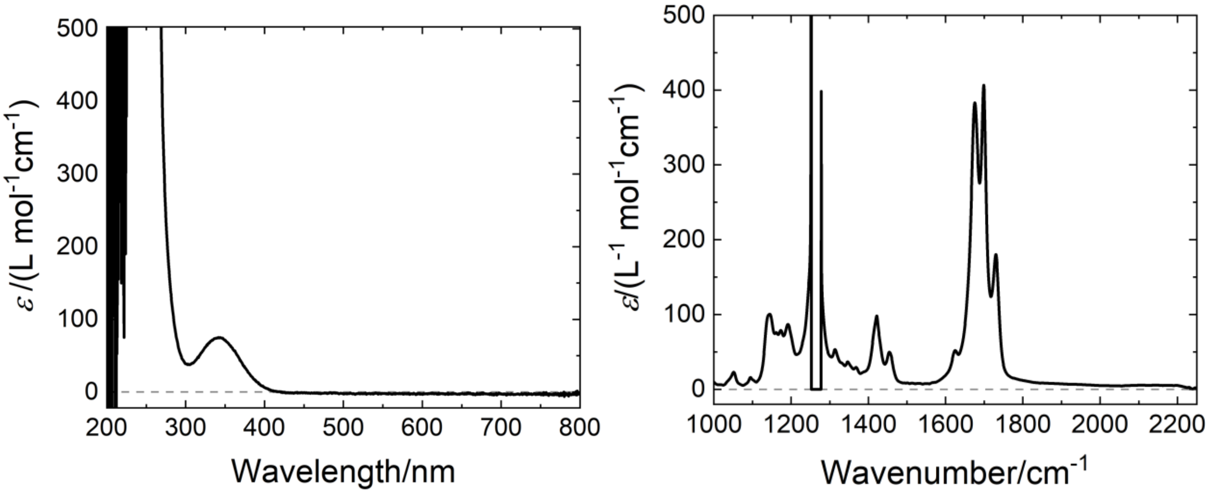

Figure S3.1. The absorption spectrum of **1** (left, c = 1.58 mM) shows absorption up to 410 nm. The IR spectrum of **1** (right, c = 63.1 mM, l = 200 µm) shows strong carbonyl signals around 1700 cm^{-1}. The negative signal around 1260 cm^{-1} is due to saturated solvent absorption.

Stepscan-FTIR Spectroscopy Setup

Stepscan measurements were performed using a Bruker VERTEX 80 FTIR spectrometer which was equipped with a fast MCT detector (D-317/BF) cooled by liquid nitrogen. The front of the lid of the sample chamber was modified to integrate a window (Edmund Optics; Fused Silica, AR 250 – 700 nm) at the height of the IR beam. It enabled the light of a ns-laser (Ekspla; NT-342C; 4 - 6 ns, 10 Hz, either 355 nm or adjustable from 210 – 2600 nm using the internal OPO) to enter the sample chamber so it could repeatedly excite the sample. For this purpose, the laser light was routed using

right angle prisms (Eksma Optics; 330-1202) into a home-made periscope utilizing the same type of prisms to match the height of the IR beam. The light was finally routed into the sample chamber and into the sample by two aluminium mirrors (Thorlabs; PF10-03-F01). The last mirror was placed inside the sample chamber on a home-made plate that was screwed to the sample holder. A spherical lens (Eksma Optics; pl/cv, f = 1000 mm, AR 210 - 400 nm), was placed after the periscope to reduce the diameter of the laser beam to roughly 2 mm. The focal diameter of the IR light was set to 1.7 mm. To avoid any laser light reaching the MCT detector or the interferometer, the sample chamber was equipped with two germanium windows (Quantum Design; BBAR 2 – 14 µm). The pulse energy was adjusted via the timing of the Q switch to a value close to 2 mJ. To reduce the duration of the measurement, a long-pass filter (Edmund Optics; 4.5 µm) was placed inside the path of the IR beam. The Synch Out connector of the laser driver was connected to the VERTEX 80 to trigger the stepscan measurement. The timing was adjusted to start the recording of a time series at each step of the interferogram before the laser pulse arrived at the Omnicell containing the sample.

Stepscan FTIR Measurement of 1 and Correction of Artifacts

To obtain dynamics of *rac*-**2**, the stepscan functionality of the Vertex 80 was utilized. The sample of **1** in dichloromethane (*c* = 71.8 mM) was directly excited using pulses generated from the OPO of the Ekspla (1.88 mJ, 340 nm). The transients of the interferogram points were measured with a temporal resolution of 6 µs over 10 coadditions. The spectral resolution was set to 4 cm^{-1}. This type of measurement was conducted five times for later averaging.

The different interferograms show slight offsets, which were corrected by fitting 10th order polynomials to the baselines of the interferograms and subtracting these polynomials (Figure S3.2).

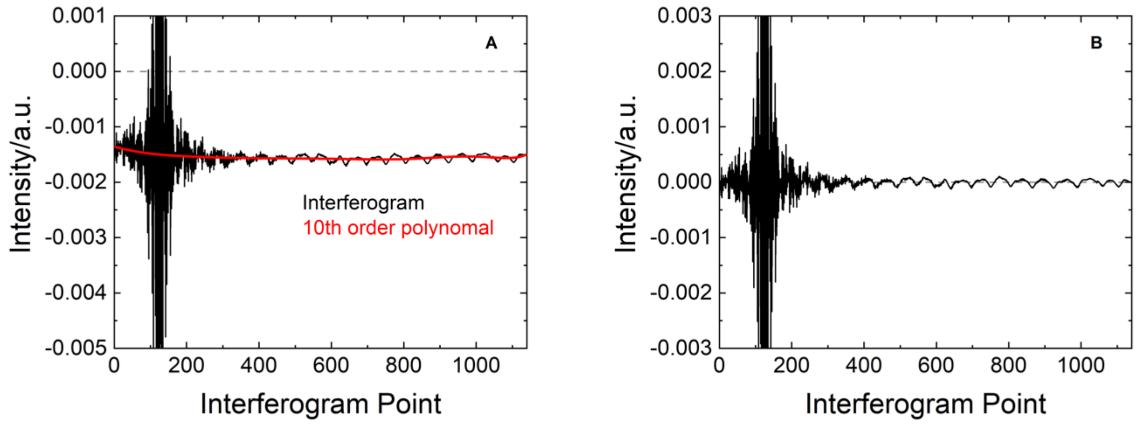

Figure S3.2. A Correction of the Interferogram Offsets using a 10th order polynomial, shown exemplary for one of the interferograms. **B** The resulting corrected interferograms lay on the baseline.

Unfortunately, we encountered artifacts in the form of oscillations along the time axis plaguing the stepscan data. These oscillations appear to have a frequency of just under 1000 Hz and occur mostly around the center burst of the interferograms, as exemplary shown for one interferogram point in Figure S3.3 (**A**). While the origin of the oscillations is unknown, they must be corrected for. We found the most convenient way to do so was to apply Fourier filtering. The interferogram transients were Fourier transformed along the time axis, which revealed peaks around 150-1000 Hz in both the real and imaginary parts (Figure S3.3, **B**), corresponding to the oscillations. The correction was performed by simply setting the values at these frequencies to zero (Figure S3.3, **D**). After inverse fourier transformation, it could be seen that the oscillations are mostly removed (Figure S3.3, **C**).

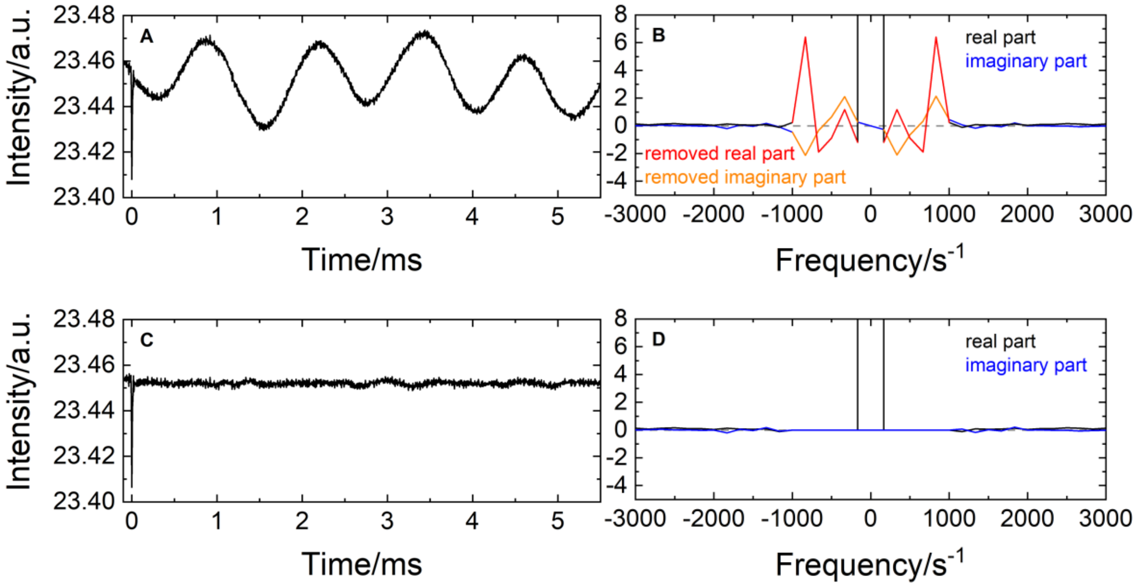

Figure S3.3. **A** Oscillations of the interferogram transients, shown exemplary for the central burst. The spike at $t = 0$ ms is caused by the laser pulse. **B** Fourier transformation along the temporal axis reveals peaks between 150 and 1000 Hz belonging to this oscillation. These peaks are present in both the real (black) and imaginary (blue) parts. They are removed by setting the values at these frequencies (labeled red and orange respectively) to zero. **C** Resulting interferogram transient after the correction. The oscillations have mostly been removed. **D** The areas of the peaks in frequency space have been set to zero.

The same process was performed for all interferogram points in all measurements, whereby within a single measurement the same frequencies were removed for all points.

For different measurements the correction was adjusted in that the removed frequencies were altered as needed, however in all cases as few frequencies as possible were removed in order not to compromise the data. Afterwards, the Fourier transformation from spatial to wavenumber domain could be performed following the described instructions.[8]

Difference spectra were obtained by averaging over the first 20 spectra (-168 µs to -54 µs) and subtracting the resulting spectrum from the entire dataset. Averaging over all five datasets yields the following results (Figure S3.4):

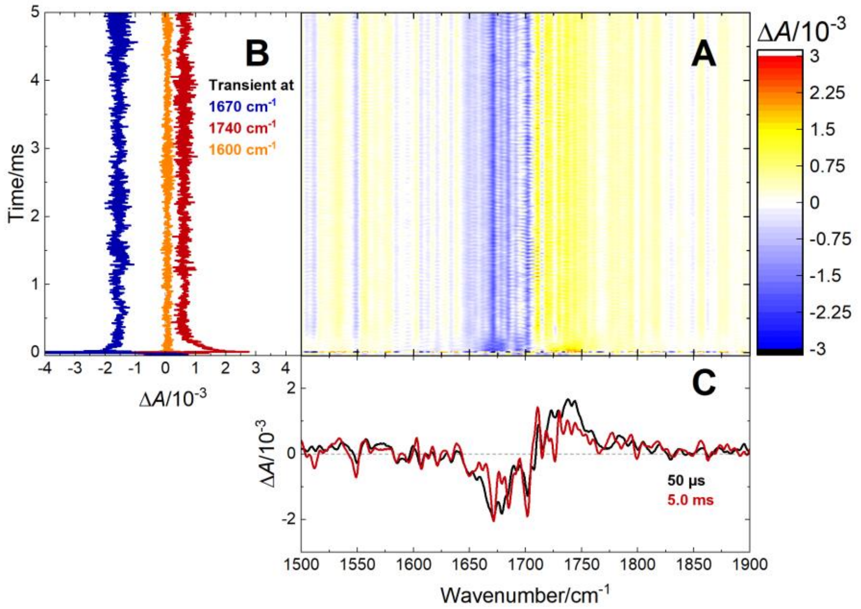

Figure S3.4. A Transient data from the stepscan FTIR measurement of **1**. **B** Transients at relevant wavenumbers. The groundstate bleach around 1670 cm^{-1} lasts for the entirety of the measurement time. Positive signals at 1740 cm^{-1} show a fast decay within the first hundreds of µs towards a plateau, which lasts longer than the measurement time. **C** Spectra taken directly after excitation and towards the end of the measurement time show a slight shift of the absorption maximum towards higher wavenumbers.

While the data is unfortunately rather noisy, one can still clearly see a positive as well as a negative signal in the carbonyl regions. The negative signal matches the inverted groundstate spectrum (Figure S3.5) and persists over the complete measurement time. The positive signal on the other hand, shows a rapid decay within the first hundreds of µs, during which the spectrum shifts slightly towards higher wavenumbers. This new signal then lasts for the entirety of the measurement time. Stationary spectra taken before and after each stepscan measurement show degradation of **1** as well as a new signal just under 1800 cm^{-1} matching the long-living signals from the stepscan data.

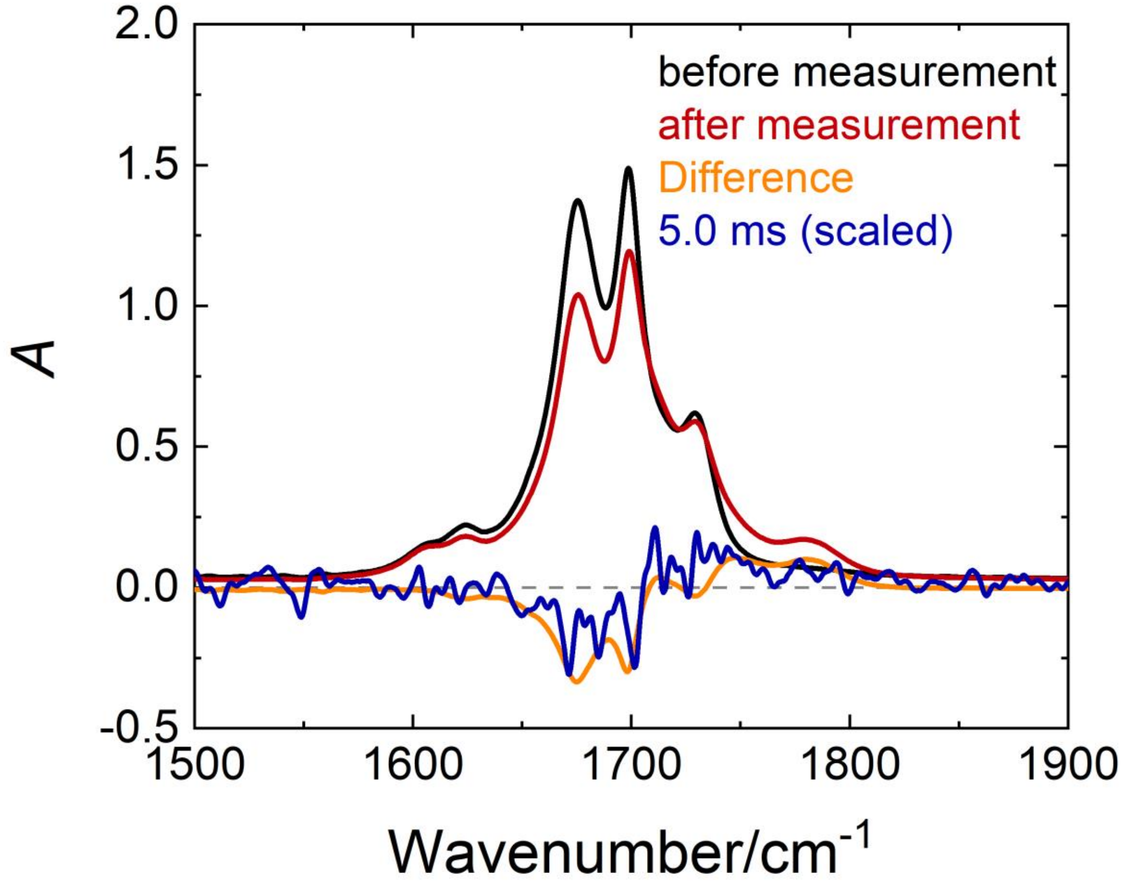

Figure S3.5. Stationary FTIR spectra before (black) and after (red) a stepscan measurement show bleaching of **1** as well as a new signal just under 1800 cm^{-1}. Their difference (orange) matches the spectra towards the end of the stepscan datasets (blue), indicating that the new signal belongs to a stable photoproduct.

The most plausible interpretation of the spectroscopic data is formation of the isomerized cycloheptenone carboxylic acid *rac*-**2**, which then undergoes some kind of reaction, leading to a decay of the signal with a lifetime in the 100 µs regime (Scheme S3.1). Possible reaction pathways include inter- or intramolecular protonation of the carbonyl group or the double bond or [2+2] cycloadditions with either **1** or other *rac*-**2** molecules. Both types of reaction are known for cycloheptenone.[9] Back-isomerization towards **1** may also play a role but cannot be the only pathway due to bleaching of the sample. If *rac*-**2** deactivated in this way, one would expect a decrease of the groundstate bleach. While the transient at 1670 cm^{-1} does indeed show this, the overall weakness of the signal makes it difficult to ascertain if this effect is real, just an artifact of the remaining oscillations or comes into being due to spectral overlap of **1** with the photoproduct **PP**.

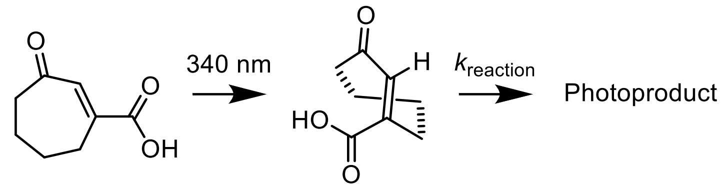

1 *rac*-**2** **PP**

Scheme S3.1. *rac*-**2**, which is formed from excitation of **1** appears to form a photoproduct **PP** leading to a stable signal just under 1800 cm^{-1} as well as bleaching of **1**. Possible reaction pathways for this include protonation of the carbonyl group as well as [2+2] cycloadditions. Back-isomerization to **1** can neither be confirmed nor excluded with this dataset.

Global Fitting

Global fitting was performed using the software KiMoPack[10], in which the fitting model is given as a set of differential equations. The results are both spectra and kinetic rate constants of the different species defined within the model. Since the raw data (Figure S3.4) indicates that first *rac*-**2** is formed, after which it undergoes a reaction to an unknown species, which can be detected *via* stationary spectroscopy even minutes later, the model shown in Scheme S3.1 was used.

Translated into a set of differential equations, the model looks the following (Equation S3.1 - S3.3):

$$\mathrm{d}c(\mathbf{1}) = -c(\mathrm{exc}) \cdot \frac{1}{\sigma\sqrt{2\pi}} \mathrm{e}^{-\frac{1}{2}(\frac{t-t_0}{\sigma})^2} \cdot \mathrm{d}t \quad (S3.1)$$

$$\mathrm{d}c(\boldsymbol{rac}-\mathbf{2}) = c(\mathrm{exc}) \cdot \frac{1}{\sigma\sqrt{2\pi}} \mathrm{e}^{-\frac{1}{2}(\frac{t-t_0}{\sigma})^2} \cdot \mathrm{d}t - k_{\mathrm{reaction}} \cdot c(\boldsymbol{rac}-\mathbf{2}) \cdot \mathrm{d}t \quad (S3.2)$$

$$\mathrm{d}c(\mathbf{PP}) = k_{\mathrm{reaction}} \cdot c(\boldsymbol{rac}-\mathbf{2}) \cdot \mathrm{d}t \quad (S3.3)$$

In this simple model the instrument response function is described by a gaussian, including the time of excitation t_0 as well as the temporal resolution σ. Since the excitation pulses of the laser are only 10 ns long, σ is only determined by the resolution of the measurement, which is 6 µs. Hence, σ is fixed to this value for the fit. t_0 is a fitting parameter, however times close to t_0 suffer from laser artifacts in the form of spikes in the transients (see Figure S3.3). In order to remove them, the starting value for t_0 is set to the time of the strongest laser artifact and times between 6 µs before and after this (so overall 3 points of each transient) are omitted from the fit.

The gaussian function is scaled by the concentration of molecules that are excited during a laser pulse *c*(exc). Since the groundstate bleach remains constant during the measurement, its inversion can be used to calculate the fraction of excited molecules by simply applying the Beer-Lambert law (Equation S3.4).

$$c(\mathrm{exc}) = A(1670\ \mathrm{cm}^{-1})/(\varepsilon(\mathbf{1}, 1670\ \mathrm{cm}^{-1}) \cdot l) \quad (S3.4)$$

Using the absorbance at 1670 cm^{-1} as well as the known extinction coefficient (Figure S3.1) and the optical thickness *l*, *c*(exc) was calculated to be about 0.1 mM. This corresponds to about 0.14 % of the sample being excited and not returning to the initial ground state. One should note that this calculation assumes that no other species than **1** absorbs at 1670 cm^{-1}. If e.g. **PP** also absorbs there, the amount of *c*(exc) is underestimated. However, the aim of these measurements is only to investigate if the *rac*-**2** can react in a diffusion-controlled manner, hence small discrepancies in the SAS resulting from the fit are not too great a concern.

The 340 nm laser pulses used had an average pulse energy of 1.88 mJ. Using the extinction coefficient from Figure S3.1 and knowing concentration and optical thickness of the sample, the amount of absorbed photons can be approximated to be about $1.49 \cdot 10^{15}$ per pulse. Estimating a diameter of 1.5 mm for the pump beam within the sample, and assuming that the beam within the sample is a perfect cylinder, this corresponds to 3.9 % of excited molecules. Taking these values the quantum yield of the isomerization would be around 3.6 %, however one should keep in mind that there are rather big uncertainties concerning the exact pump energy, the size of the pump beam's focus and the exact amount of groundstate bleach.

To minimize artifacts of both the laser and the remaining oscillations, delay times between -6 µs and 6 µs as well as above 2 ms were omitted from the fit. The optimized parameters after the global fit are shown in Table S3.1.

Table S3.1. Optimized Parameters from the global lifetime analysis. The values in red were fixed and not optimized.

Parameter	Value
t_0	20.1 µs
σ	6 µs
c(exc)	0.1 mM
$k_{reaction}$	$7.71 \cdot 10^3$ s^{-1}

The optimized species associated difference spectra (SADS) of this global analysis were converted to species associated spectra (SAS) by adding the spectrum of **1** to each of them. The results are shown in Figure S3.6.

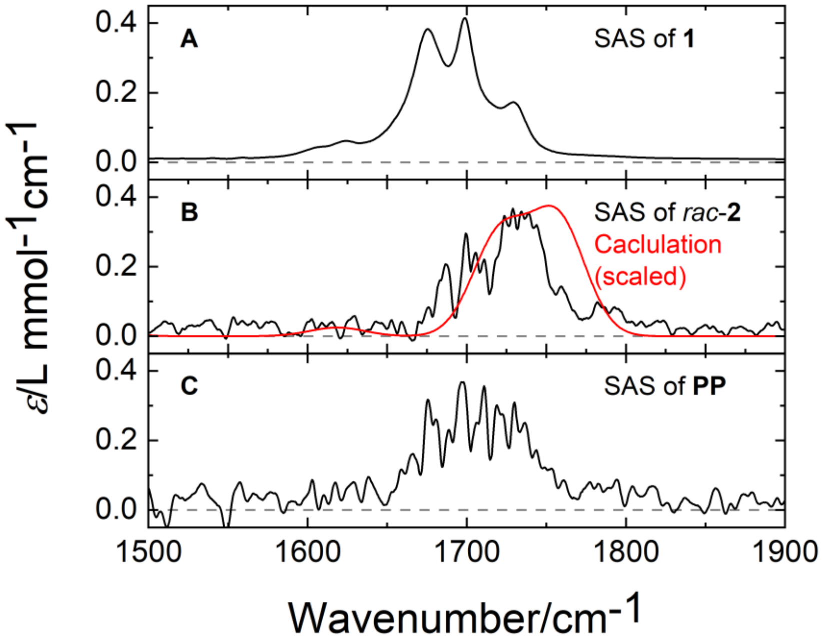

Figure S3.6. SAS resulting from the global analysis of the stepscan data. **A** The spectrum assigned to **1** matches the spectrum from stationary measurements. **B** The spectrum assigned to *rac*-**2** shows strong absorption at about 1740 cm^{-1}, the decay of which can be traced. Although there is a small spectral shift, it is in reasonable agreement with the calculated IR spectrum of *rac*-**2**. **C** The spectrum assigned to the photoproduct resembles the signal lasting longer than the timescale.

All of these SAS are very similar, as they are all assigned to carbonyl signals of very closely related molecules. The fitted value of the rate constant results in a lifetime for *rac*-**2** of 130 µs, assuming a first order reaction, placing it well in the timescale of taking part in diffusion-controlled reactions. If the reaction is in fact not first but second order, it becomes dependent on the concentration. However, this is not too big of a concern, since in this study synthetic concentrations are actually lower than the spectroscopic ones, meaning the lifetime in synthesis would be even higher.

To evaluate the quality of the fit the residues should be examined (Figure S3.7). While there are still spectral components unaccounted for, these are in the same order of magnitude as the remaining oscillations. Therefore, the origin of the residues could be due to a lack of fitting quality or remaining artifacts. However, the data appears to mostly be described by the model.

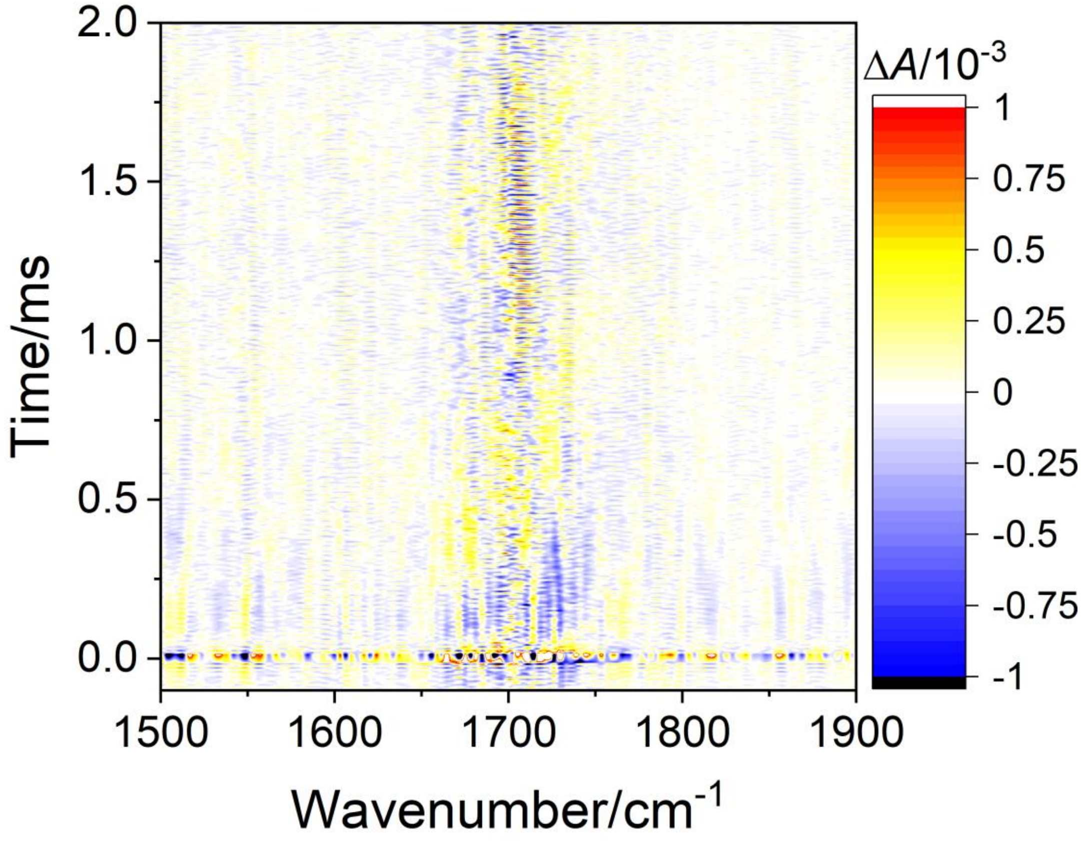

Figure S3.7. The residues of the fit still show small spectral components. However, they are in the same order of magnitude as the remaining artifacts from the oscillations (see Figure S3.8), therefore it is plausible to assume that the fit describes the data sufficiently well.

As an additional criterion for the descriptive power of the global analysis, the resulting fitted transients and spectra can be compared to the measured ones to see if the most relevant features are described. For this purpose, the transients at 1670 cm^{-1}, describing the groundstate bleach, 1725 cm^{-1}, mostly describing *rac*-**2**, and 1740 cm^{-1}, composed of both *rac*-**2** and **PP**, are compared to transients predicted by the fit (Figure S3.8, A). The spectra during the time of the decay are also mostly reproduced by the fit (Figure S3.8, B).

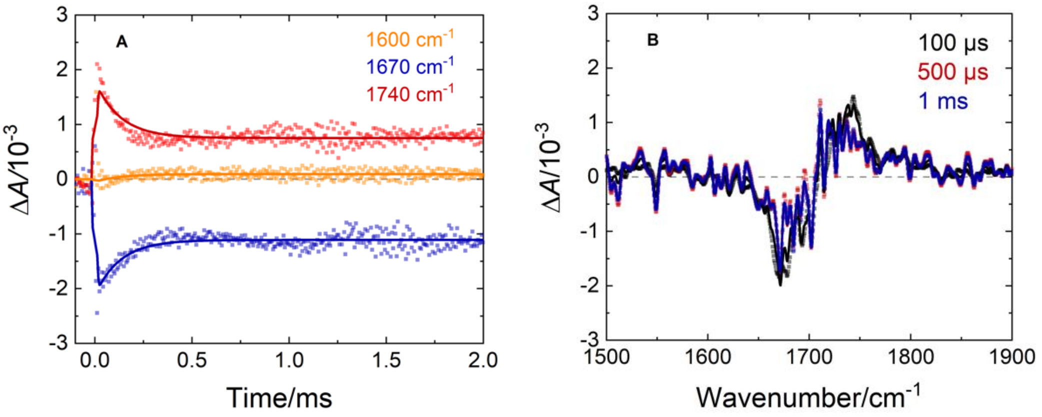

Figure S3.8. Comparison of the measured data with the fit, whereby points indicate measured values and lines represent the results of the fit. **A** The transients at relevant wavenumbers show that the dynamics are mostly well described. It can also be seen that the deviations in Figure S3.7 stem mostly from the remaining oscillations. **B** The spectra at relevant times are also described well by the fit.

Therefore, we conclude that the fit describes the data reasonably well, lending credit to the model used (Scheme S3.1), which in turn corroborates the interpretation of the stepscan data, while the fitted lifetime of *rac*-**2** would enable it to easily participate in diffusion-controlled reactions.

4. Computational Studies

Unless stated otherwise calculations applied the default settings recommended for the respective quantum chemistry programs. Density functional theory (DFT) methods used the highest order integration grid available to each program package. Nuclear gradients were evaluated in an analytical manner.

General Methods

Electronic Structure Methods: Excited States

Calculations on the SA(n)-XMS-CASPT2(e/o) level of theory were conducted with BAGEL 2019.[11] Unless stated otherwise, triple-zeta cc-pVTZ basis set was employed, along with the cc-pVTZ/JKFit auxiliary basis set for density fitting.[12] The wavefunction for the multi-state perturbation theory treatment was obtained from a state-averaged CASSCF calculation of n electronic states with an active space over e electrons and o orbitals, typically 10 electrons in 8 orbitals, comprising the 6 π-orbitals and carbonyl oxygen lone pairs and state averaging over 9 states was used (see detailed information below). For the multi-state part of the perturbation theory calculation, the single-state single-reference (SS-SR) internal contraction scheme was used. An imaginary shift of 0.2 eV was applied and no IPEA shift, the latter being justified by previous results on the effect of an IPEA shift for organic chromophores.[13]

Multi-reference perturbation theories are established as quasi-'gold standard' for the characterization of excited states.[14] To correctly describe the electronic structure at (near-) degeneracies, quasi-degenerate perturbation theory is required. A previous study found a superior performance of XMS-CASPT2 for smooth potential energy surfaces (PES) compared to multi-state (MS), extended dynamically weighed multi-state (XDW) and rotated multi-state (RMS) CASPT2 at or near conical intersections (CoIns).[15] Continuous PES are important for the numerical stability of surface hopping dynamics simulations. A further advantage of XMS-CASPT2 is the availability of analytical gradients.

Calculations on the time-dependent DFT (TD-DFT) ωB97X-D3 level of theory were carried out with ORCA v5.0.3.[16] The Def2-SVP basis set was used along with the Def2/J auxiliary basis set for the resolution-of-the-identity approximation.[17] Very tight SCF convergence thresholds were applied. This method was chosen for excited state optimizations when XMS-CASPT2 was computationally intractable. ωB97X-D3 is widely recognized as a reliable DFT functional for which analytical excited state gradients are available.

Calculations on the RI-SCS-ωPBEPP86 level of theory were carried out in ORCA v5.0.3.[18] The Def2-TZVP basis set was used along with the Def2/JK and Def2-TZVP/C auxiliary basis sets for the RIJCOSX resolution-of-the-identity approximation.[19] The SMD implicit solvation method was applied (solvent: dichloromethane).[20] Very tight SCF convergence thresholds were applied and the Tamm-Dancoff approximation (TDA) and relaxed difference densities were used. The ωPBEPP86 functional was chosen since a previous study had shown a superior description of charge transfer states compared to other functionals.[21] Moreover, we performed a limited benchmark on catalyst **8a**, probing the LC-PBE0, PBE0, ωB97X-D3, BHandHLYP and CAM-B3LYP functionals, all yielded a qualitatively worse performance for excited state excitation energies and state orderings at an optimized geometry on the R^2SCAN-3C level of theory. The functionals LC-PBE0, PBE0, ωB97X-D3, BHandHLYP and CAM-B3LYP underestimated the energy of charge transfer states and overestimated (n_O π^*) states.

Trajectory Surface Hopping Dynamics

The gas phase UV-vis spectrum of cyclohept-2-enone (CHp) in the *Z*-configuration was generated from three conformers (denoted as CHp-Z1, CHp-Z2 and CHp-Z3) using a harmonic Wigner distribution at 298.15 K, as detailed in a previous publication.[4,22] Spectral sampling was extended to 2000 single point calculations over the full conformer space, according to the respective DLPNO-CCSD(T)//ωB97X-D2 Boltzmann weights. Surface hopping dynamics simulations were performed using an in-house modification of the SHARC v2.1.1 program suite, which includes an interface that combines gradient information obtained from BAGEL 2019 with spin-orbit couplings and wavefunction overlaps obtained from OpenMolcas v22.10.[23] To ensure synchronicity of both calculations and enhance performance, the orbital coefficients of BAGEL were translated into the OpenMolcas formatting, read as starting guess orbitals and wavefunction construction handled via the keyword CIONLY.

Initial conditions were retrieved from the calculation of the gas phase spectra (*vide supra*): A probabilistic selection (random number seeds: CHp-Z1: 768657645, CHp-Z2 and CHp-Z3: 637628376, SHARC format intitial condition files provided in the electronic repository) was simulated via the instantaneous transition dipole moments relative to the ensemble maximum, within an energy range of 0 eV to 4.15 eV (299 nm). This corresponds to the experimental conditions of a previous publication on the photochemistry of CHp[4] and led to exclusive population of the S_1. A total of 106 independent trajectories were simulated (37 for conformer CHp-Z1, 48 for conformer CHp-Z2, 21 for conformer CHp-Z3); Note that these ratios do not reflect the Boltzmann weights due to differences in transition dipole moments between the conformers, favoring CHp-Z2.

The surface hopping trajectories were propagated in the spin-diagonal representation with a default time step of 0.5 fs. 25 sub-steps for the propagation of the electronic wavefunction were performed. Non-adiabatic effects were included using local diabatization.[24] As decoherence correction, the A-FFSH scheme was employed.[25] After a successful hop, the velocity was re-scaled along the gradient difference vector. For frustrated hops, the velocity was reflected in direction of the gradient difference vector.[26] To save computational time, trajectories were terminated after 100 fs of continuous population in the electronic ground state (MCH representation). Gradients and couplings used in the spin-diabatic to spin-adiabatic transformation were calculated only for states whose energy difference to the classically occupied state was smaller than 0.5 eV. Spin-orbit couplings were calculated using the RASSI module of OpenMolcas 22.10. The trajectories were propagated at the SA5-XMS-CASPT2(8/7) level of theory (vide infra) using the cc-pVDZ basis set and auxiliary basis set cc-pVDZ/JK, as larger basis sets were prohibitively expensive in dynamics simulations.[27] The cc-pVDZ basis set used in surface hopping dynamics simulations was validated across multiple metrics against the very large aug-cc-pV5Z basis set (*vide infra*, cf. Table **S4.6** and **Figure S4.7**).

Electronic Structure Methods: Ground State

Calculations on the GFN2-XTB level of theory were carried out in CREST v3.0.[28]

Calculations on the ωB97X-D2 DFT level of theory were carried out in Gaussian16[29] with the aug-cc-pVTZ basis set, along with the polarizable continuum model (PCM) implicit solvation model (solvent: dichloromethane) and tight convergence thresholds.[30] The range separated hybrid functional ωB97X is well-known to produce accurate ground state geometries and frequencies, and has been used in a previous publication on CHp.[4] It was chosen for the treatment of CHp to obtain the best possible Boltzmann weights for the initialization of the computationally highly demanding TSH dynamics simulations.

Calculations on the R^2SCAN-3C level of theory were carried out with either CREST or ORCA v5.0.3.[31] The Def2-mTZVPP basis set was employed along with either the SMD (CREST) or CPCM (ORCA) implicit solvation model (solvent: dichloromethane).[32] The R^2SCAN-3C level of theory was chosen for the treatment of all systems in their ground state, except for CHp, a decision that was motivated by the low computational cost, allowing to optimize hundreds of large substrate-catalyst adduct conformers with up to 144 atoms and 618 electrons. At the same time, equal-to-superior accuracy was reported in literature for ground state barriers of activation and Boltzmann weights compared to double hybrid functionals.[33] For conformers of the adduct between **1** and **8a**, we found a significantly better agreement in Boltzmann weights between the R^2SCAN-3C functional and DLPNO-CCSD(T) than ωB97X-D2.

Calculations on the DLPNO-CCSD(T) level of theory were carried out with ORCA v5.0.3.[34] The SMD implicit solvation model was employed (solvent: dichloromethane), scalar-relativistic corrections were included via the Douglas-Kroll-Hess Hamiltonian and the resolution-of-the-identity approximation (RIJK) was employed. PNO thresholds and SCF convergence thresholds were set to very tight. Due to the slow convergence of the correlation energy with respect to basis set size, the final energies were evaluated in the complete basis set limit (CBS) by two-point extrapolation.[35] For the small compounds 1, 2, ent-2, DA-TS, LAC-TS, DA, ent-DA, LAC, ent-LAC, LAC'-TS, 3e, ent-3e and CHp, the cc-pVTZ and cc-pVQZ basis sets were used, whereas for all adducts with the catalyst 8a, the basis sets cc-pVDZ and cc-pVTZ were employed. CCSD(T) is a generally accepted 'gold-standard' for the treatment of ground state species with closed-shell configuration and the DLPNO approximation has been validated for the investigation of large systems.[36]

Conformer Sampling and Geometry Optimization

Unless stated otherwise, all species discussed in this article were subjected to a thorough conformer screening procedure in full conformer space. We pursued an iterative approach that is comprised of macro- and micro-iterations: In each macro-iteration, the starting guess geometry was first optimized on the GFN2-XTB level of theory (level 1) and then subject to a meta-dynamics sampling on the same level of theory in the gas phase.[37] Samples retrieved from these simulations were re-optimized on the GFN2-XTB level of theory and the 6 conformers lowest in energy re-subjected to meta-dynamics simulations (micro-iterations). This procedure was repeated until no new lowest energy conformers were found after a given cycle. The finally obtained conformational samples were used in high-level R^2SCAN-3C ensemble optimization (level 2a). The resulting geometries were sorted with respect to their Gibbs free energy, and those within 99% of the Boltzmann sum subjected to a refined high-level DFT optimization with tightened convergence thresholds (level 2b). For these final geometries, ab-initio single point calculations (level 3) were performed and the final Gibbs free energy of each conformer calculated as the sum of the level 3 absolute energy and level 2b thermochemical corrections and zero-point energies (at standard conditions). The three lowest Gibbs free energy conformers entered the next macro-iteration (level 1 meta-dynamics, level 2 optimization, level 3 energy refinement), until the composition of 99% of the Boltzmann sum was stable at level 3.

For the adduct of **1** and catalyst **8a**, we started with multiple independent meta-dynamics simulations from geometrically different initial guesses. Due to the underestimation of Van-der-Waals interactions between the enone and catalyst fragments on the level 1 method, the results were qualitatively mismatched, i.e., we find different level 3-refined binding motives. We circumvented

this effect by manually identifying geometric features for which the rank between the level 1 and level 3 ensemble changed considerably, and restrained the subsequent meta-dynamics simulations to favor low energy and disfavor high energy (intruder) geometry features. With each macro-iteration, these restraints were tightened. In detail, meta-dynamics simulations in the first macro-iteration were unrestrained and comprised three meta-dynamics groups from independent initial guesses. In the second cycle, the distance between the phosphoric acid and carboxylic acid moieties was restrained. In the third, two groups were simulated with additional restraints for the distance to either thioxanthone moieties. In the fourth and final cycle, the entire molecule was constrained, except for the alkyl moieties of the catalysts backbone. The Gibbs free energies discussed in the main text are the Boltzmann-weighed sum of the final level 3 ensemble. We note that the automatization of this procedure is fairly straightforward. Meta-dynamics simulations were performed on the GFN2-XTB level of theory as level 1 in gas phase using the CREST program, treating a group of six independent trajectories. For the sampling of adducts between multiple species, a repulsive potential wall was used to confine the components, the parameters of which were automatically determined via the NCI command. A sampling frequency of 100 fs was used, with the length of the trajectories automatically determined. Spearman-rank ensemble optimization was performed within the CENSO v1.2.0 program, with the R^2SCAN-3C method for level 2 and implicit solvation handled as described above.[38] A pre-screening on the GFN2-XTB geometries was performed to sort out high-lying conformers, applying a sorting threshold of 3.5 kcal mol^{-1} against the geometry lowest in energy. The ensemble optimization applied a sorting threshold of 2.5 $kcal^{-1}$ and 8 optimization cycles per sorting event. Refined optimization on level 2 was done within ORCA 5.0.3. and very tight convergence thresholds required for both the SCF and geometry optimization cycles. An exception to this was the treatment of the CHp conformers, which were optimized on the ωB97X-D2 level of theory as level 2b. Unless stated otherwise, DLPNO-CCSD(T)/CBS was used as the level 3 method.

For the screening of transition states, the initial guess was pre-optimized on the R^2SCAN-3C level of theory, using the nudged elastic band (NEB) algorithm with an image-dependent pair potential (IDPP) initial guess for the minimum energy path (MEP), and all meta-dynamics sampling conducted with a hard constraint for the bonds breaking/forming during the reaction. The screening for the ground state OBF transition states involved pseudo-excited state meta-dynamics simulations optimizing the S0-T1 gap on the GFN2-XTB level of theory, followed by unrestricted R^2SCAN-3C (UR^2SCAN-3C) level 2a ensemble optimization and spin-flipp R^2SCAN-3C level 2b refined transition state optimization. The screening for triplet state minima was analogous, but with the refined optimization performed in the triplet manifold on the UR^2SCAN-3C level of theory. OBF-configured conical intersections with the ground state were optimized from the 3 lowest level 2b

energy conformers in the triplet manifold (UR²SCAN-3C). Optimizations of excited state stationary points (minima, transition states and conical intersections) on the XMS-CASPT2 level of theory were performed in gas phase via the SHARC v2.1.1 interface between BAGEL 2019, providing the energies and energy derivatives, and ORCA v5.0.3, handling the geometry optimization. For all optimized minimum energy geometries, the absence of imaginary frequencies was ensured, and for all optimized transition state geometries, the presence of one imaginary frequency was ensured.

Detailed Descriptions

Definition of Essential Reaction Coordinates

For the discussion of the excited state relaxation cascades, a decomposition of the nuclear motion of the trajectory ensemble in reaction coordinates is convenient. We discern three levels:

First, fundamental reaction coordinates (FRCs) are used which represent a single internal degree of freedom, comprising meaningfully defined bond lengths, angles and dihedrals.

Second, essential reaction coordinates (ERCs) mediate between the start or end of the excited state dynamics and well-characterized intermediates. As such they are (a) process-specific, (non-)linear combinations of a minimum number of FRCs necessary to induce a meaningful electronic or geometric change, while (b) excluding stochastic motions. For example, the transition from the Franck-Condon (FC) region of the $^1(n_O\pi^*)$ state to the $^1(\pi\pi^*)$ minimum requires at least four FRCs to be activated: in-sync orthogonalization of the H1-C2-C3-H2 dihedral and C1-C2-C3-C4 dihedral, as well as contraction of the C1-C2 bond and elongation of the C2-C3 bond. In contrast, stochastic C-H bond alterations of the aliphatic fragment do not enter the ERC. As such, ERC can be constructed from combinations of lower-order ERCs acting on different fragments of the molecule, provided their simultaneous activation is not subject to random fluctuation: Within the main text, the triplet manifold transition from FC to the ent-OBF face of a CHp conformer (CHp-Z-2) set to a standard orientation is discussed, which forced a specific alkyl fragment reorganization to strictly coincide with OBF-relaxation of the chromophore fragment, due to strain. As such, the ERC characterizing this process is a combination of two ERCs describing the fragment-specific reorganization subprocesses. In the case of the OBF-face transition, the same subprocesses are active, but in a sequential fashion, such that the OBF-ERC alone suffices to describe the $(\pi\pi^*)$ relaxation. In this work, ERCs were defined via manual inspection of automated principal component analysis, performed within in-house software.

Third, the global reaction coordinate (GRC) is defined as a sequence of ERCs which maps the relaxation from the initial conditions to the ground state like a classical particle, i.e., the mean transition-synchronized trajectory toward a specific photo-product.

As intermediates, we selected the (ent-)OBF minima in the singlet and triplet manifold. In surface hopping simulations they are sufficiently long-lived to be, in principle, experimentally observable. Similarly, we divide the relaxation cascade into an ultra-fast, quasi-ballistic period and a slower, quasi-stochastic period. This justifies the definition of the GRC. Supporting material on the singlet manifold late-stage ERCs is provided in the next subsection.

Comparison of CHp and 1

The active space and model space for XMS-CASPT2 calculations of compound **1** were determined by systematic variation. A choice of 10 electrons in 8 orbitals, comprising the 6 π-orbitals and carbonyl oxygen lone pairs, along with state averaging over a model space of 9 states, gave a stable active space along the entire OBF reaction coordinate (Figure S4.1).

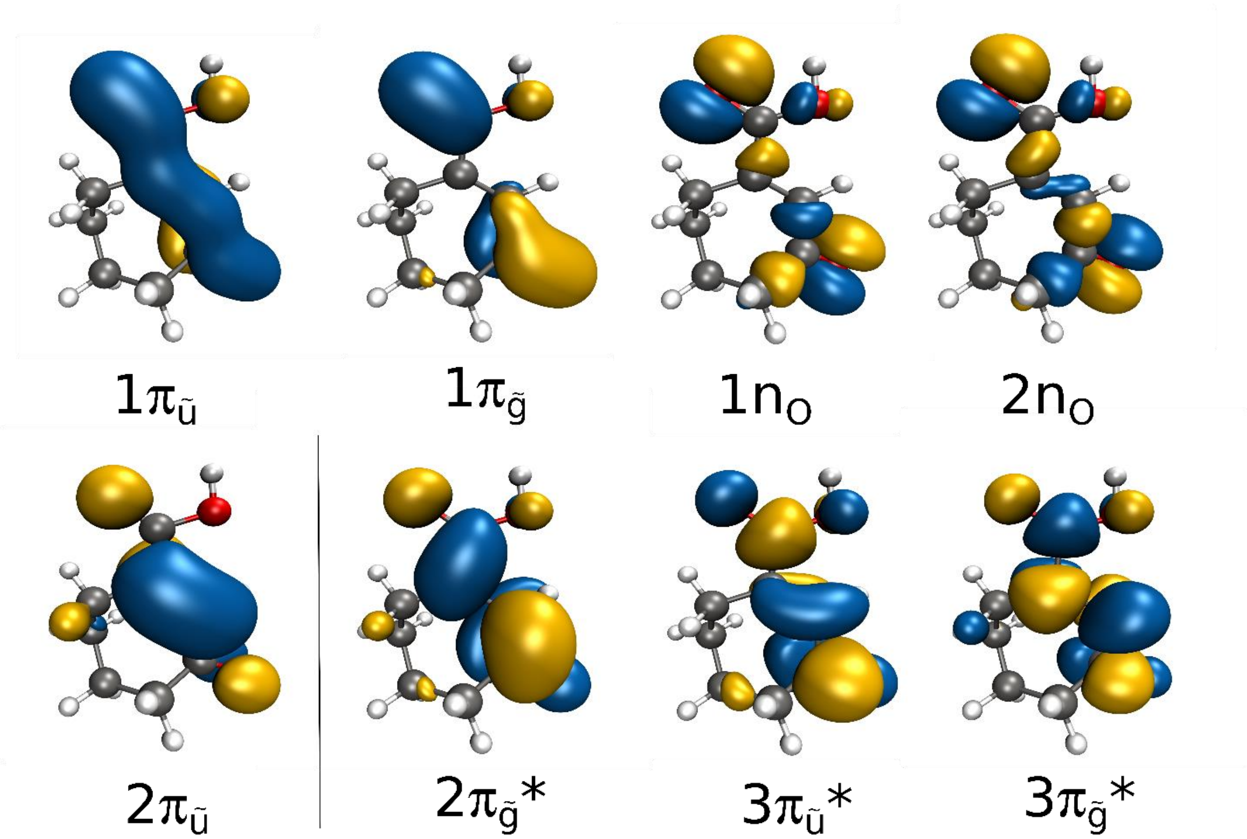

Figure S4.1. Choice for the CASSCF/XMS-CASPT2 active space of **1**, comprising all π orbitals and carbonyl oxygen lone pairs, thus forming a quasi-natural active space. Extension by a hydroxyl-oxygen lone pair did not improve the description of the excited states (*vide infra*). Since the chromophore is derived from butadiene, the π molecular orbitals are labeled with subscript indices denoting their pseudo-parity. Orbitals are shown with an isovalue of 0.05.

The (10/8) active space was used for the calculation of vertical excitation energies in the gas phase for a ground state geometry optimized on R²SCAN-3C level of theory. Excitation energies are in good agreement with RI-SCS-ωPBEPP86/Def2-TZVP excitation energies (SMD implicit solvation, dichloromethane). The excitation characters, quantified via natural transition orbitals (NTOs) generated with THEODORE, agreed well with the experimental data in dichloromethane solution (Table S4.1).[39]

Table S4.1. Comparison of vertical excitation energies (in eV) calculated on XMS-CASPT2/cc-pVTZ level of theory for different combinations of active space and model space, as well as on RI-SCS-ωPBEPP86/Def2-TZVP level of theory. Simulations are compared to the experimental maxima of distinct absorption bands, along with an assignment of the state character. CO denotes a lone pair on the carbonyl, CO2H a lone pair on the carboxyl moieties.

**Excited State Method**	**S_1 ($n_{CO}\pi^*$) [eV]**	**S_2 ($n_{CO2H}\pi^*$) [eV]**	**S_3 ($\pi\pi^*$) [eV]**
SA5-XMS-CASPT2(8/6)	3.40	4.38	6.25
SA9-XMS-CASPT2(10/8)	3.73	5.04	5.51
SA5-XMS-CASPT2(12/9)	3.52	4.76	6.28
RI-SCS-ωPBEPP86	3.47	4.77	5.23
Experiment	3.64		5.21

The simulations assign the band at 3.6 eV in the experimental spectrum as a formally forbidden excitation from the carbonyl oxygen into the LUMO π*-orbital. The second, forbidden ($n_O\pi^*$) absorption is overlaid by the bright HOMO-LUMO (ππ*) transition with single excitation character, which accounts for the band observed around 5.21 eV. The simulations on RI-SCS-ωPBEPP86/Def2-TZVP level of theory further demonstrate that the effect of a non-polar solvent environment on the low lying excited states of enones is minor, in agreement with previous publications.[4,40] This justifies neglecting the effect of the solvent environment in XMS-CASPT2 calculations.

Excited state stationary points and minimum energy crossing points (MECPs) were optimized in gas phase at the SA9-XMS-CASPT2(10/8)/cc-pVTZ level of theory. Starting geometries were the lowest energy conformers obtained from the meta-dynamics sampling as described above, using singlet manifold meta-dynamics for stationary points in the FC region and triplet manifold meta-dynamics for stationary points in the OBF region. Compared to their counterparts at the SA5-XMS-CASPT2(6/5)/cc-pVTZ level of theory for CHp, the dominant conformer of the aliphatic moiety was

the same. Comparing the energies relative to the FC level (Figure S4.2, Table S4.2, Table S4.3), deviations along the early OBF-ERC were negligible and reached 8 kJ/mol at most.

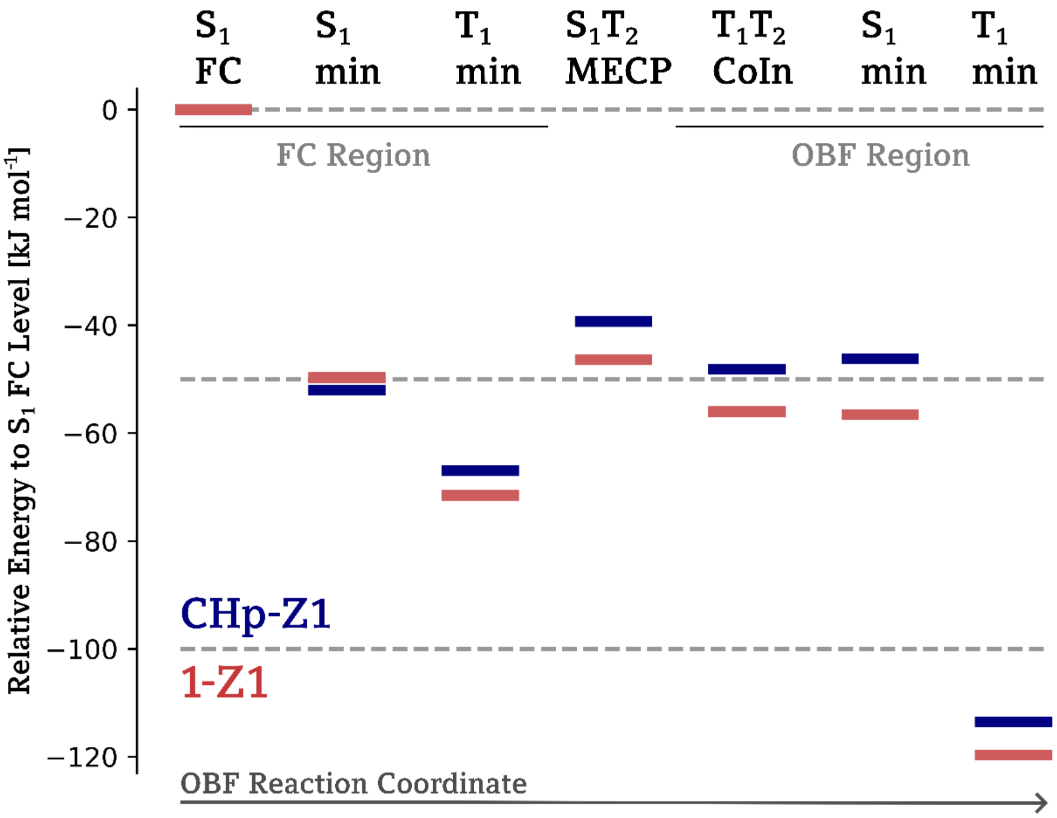

Figure S4.2. Comparison of energies of optimized excited state stationary points and minimum energy crossing points between **1** (blue) and CHp (red) at the XMS-CASPT2/cc-pVTZ level of theory, relative to the energy of the S_1 at the FC geometry.

Table S4.2. Absolute energies (singlet manifold) of stationary points of **1**, optimized at the SA9-XMS-CASPT2(10/8)/cc-pVTZ level of theory. Cartesian atomic coordinates are available from the electronic data repository.

**Geometry**	**Absolute Energy (S_0) [Hartree]**	**Absolute Energy (S_1) [Hartree]**
FC minimum	-535.5624138	-535.4340798
S_1 (planar minimum)	-	-535.45300058
S_1T_2 (MECP)	-	-535.4517524
S_1 (OBF minimum)	-	-535.455636

Table S4.3. Absolute energies (triplet manifold) of stationary points of **1**, optimized at the SA9-XMS-CASPT2(10/8)/cc-pVTZ level of theory. Cartesian atomic coordinates are available from the electronic data repository.

**Geometry**	**Absolute Energy (T_1) [Hartree]**	**Absolute Energy (T_2) [Hartree]**
T_1 (planar minimum)	-535.46132200	-
S_1T_2 (MECP)	-535.45188440	-
T_1T_2 (CoIn)	-535.45541814	-535.45540948
T_1 (OBF)	-535.47966924	-

Compared to CHp, the OBF minima of **1** appear slightly more stabilized, which supports the notion that if CHp shows no interconversion between the chiral OBF channels, neither should **1**. However, for the singlet manifold, we identified four accessible CoIns with the S_0 along late-stage ERCs (Figure S4.3, Table S4.4).

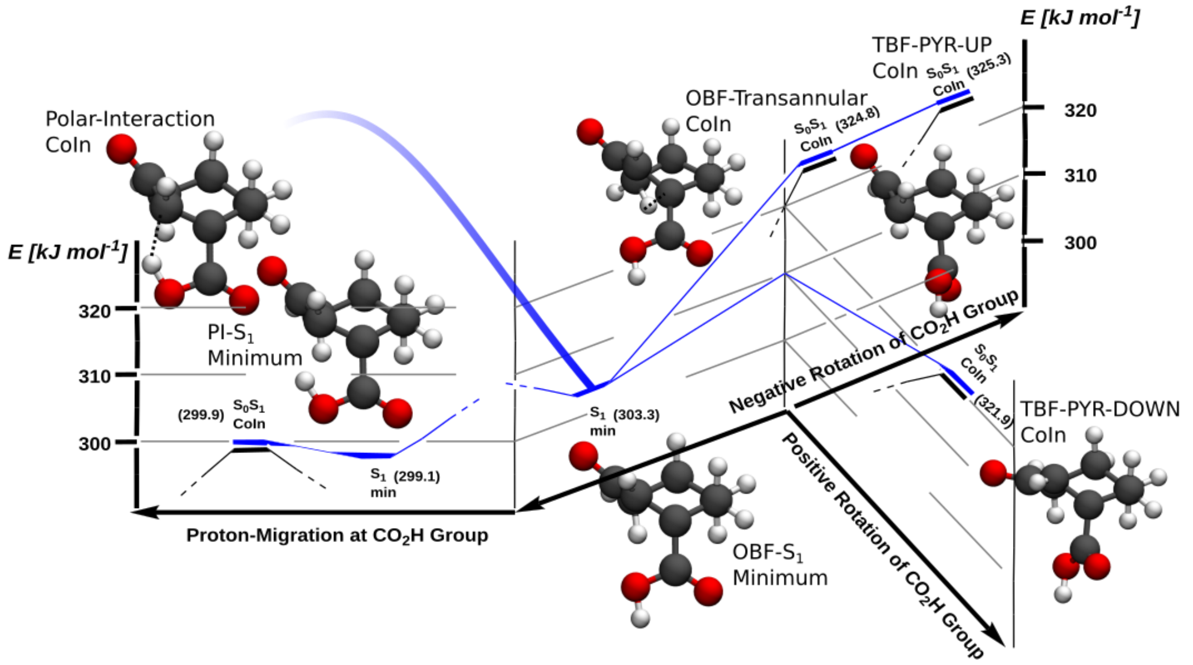

Figure S4.3. Summary of singlet manifold late stage ERCs of **1**, showing the coupling of the immediate S_1 minimum (center) to multiple accessible conical intersections. The thick faded line symbolises entry into the OBF region from the FC region. From there, four ERCs are accessible, comprising an approach of a transannular hydrogen toward the C3-carbon center, two two-bond-flip

(TBF) motions coupled with opposing (up/down) pyramidalization about the C2-carbon center, and torsion of the OH-group introducing a polar interaction between the acidic hydrogen and C2-carbon center. Energies relative to the FC point ground state are reported in brackets. The level of theory was SA9-XMS-CASPT2(10/8)/cc-pVDZ.

Table S4.4. Absolute energies of stationary points of **1** in the OBF and TBF configuration spaces, optimized at the SA9-XMS-CASPT2(10/8)/cc-pVDZ level of theory. Cartesian atomic coordinates are available from the electronic data repository.

**Geometry**	**Absolute Energy (S_0) [Hartree]**	**Absolute Energy (S_1) [Hartree]**
S1 (OBF minimum)	-	-534.921563
S0S1 (OBF-Transannular-CoIn)	-534.913393	-534.913380
S0S1 (TBF-PYR-DOWN CoIn)	-534.913220	-534.913197
S0S1 (TBF-PYR-UP CoIn)	-534.914484	-534.914478
S1 (PI-minimum)	-	-534.923177
S0S1 (PI-CoIn)	-534.922898	-534.922882

In the vicinity of the OBF S_1 minimum, we found a CoIn exhibiting a transannular interaction with a methylenic hydrogen. Two C2-pyramidalized CoIns were found energetically almost equal to the transannular motif and required a two-bond-flip motion (TBF), rotating the acidic OH-group either into or away from the heptacycle. The TBF motion is explicable within an extended 2-electron-2-orbital (x2e2o) model, which requires a maximization of site energy difference for OBF-configured degeneracies and since both the carboxy and carbonyl moieties are electron-withdrawing, the sterically less confined carboxy moiety rotates to minimze overlap.[41] While these geometric motifs also occur on the CoIn seams of CHp, their energetic splitting is much larger. Moreover, a second, low-energy OBF-configured singlet minimum was identified, where via an unknown transition state the acidic proton rotates by 180°. Upon partial dissociation of the O-H bond, the C2-carbon site experiences a strong electron-withdrawal, the C3-carbon site electron-donation. As such, the condition for a S_0S_1 degeneracy is fulfilled with minimal geometric distortion, and accordingly, a fourth CoIn is accessible practically barrierless. This crossing seam topology is a unique effect of the C3-carboxy moiety. As such, the late-stage singlet excited state dynamics between **1** and CHp are expected to differ considerably. Thus, CHp is only a good model if the OBF motion is activated in

the triplet manifold. Due to good access toward the S_1T_2 crossing seam along the OBF-ERC and, to a minor extend, heavy-atom effect of the oxygens, we expect **1** to perform ISC at or below the timescales of CHp. Hence, the choice for the CHp model is sensible.

TSH Dynamics Simulations

Trial simulations on conformer CHp-Z1, utilising the natural active space of 6 electrons and 5 orbitals (all four π-orbitals and one oxygen lone pair), showed spurious photo-reactivity, i.e., frequent Norrish Type-I bond breaking of the σ-C-C bond between the carboxy carbon and aliphatic moiety. This finding is in contradiction to experimental observations, where no Norrish Type I degradation products were identified. Extension of the active space to 8 electrons and 7 orbitals (Figure S4.4), by including the σ orbitals describing the bond breaking, deactivated the Norrish reaction coordinate during the dynamics, in agreement with prior findings.[4]

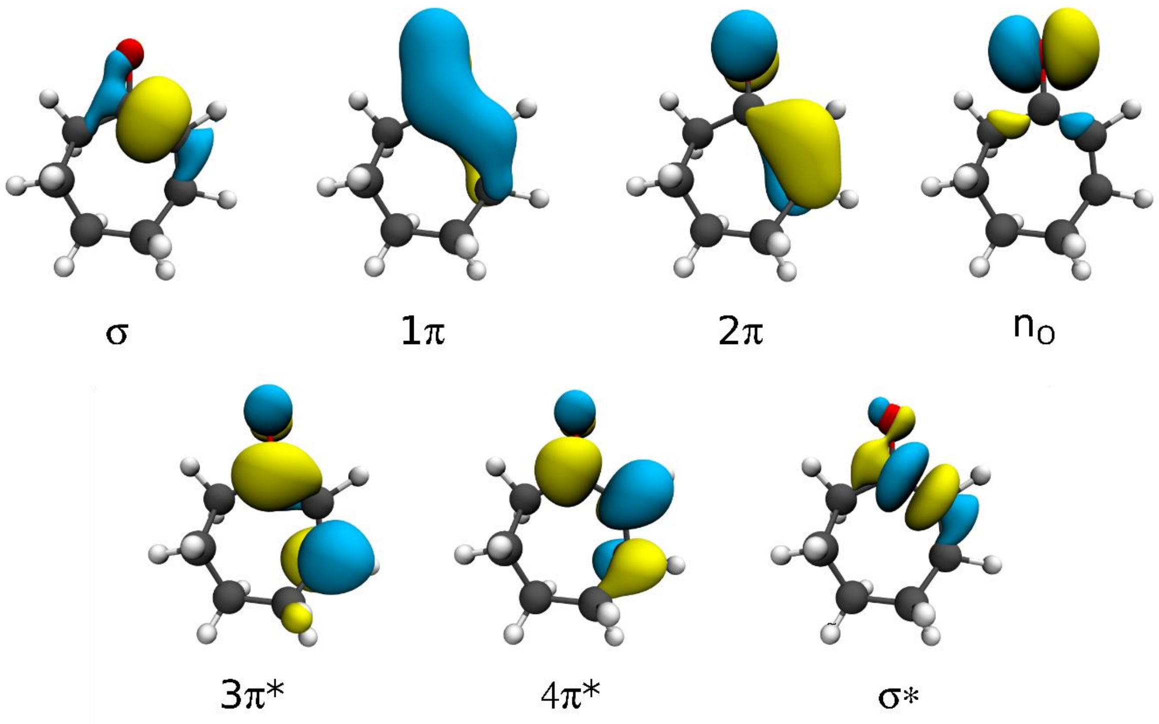

Figure S4.4. Extended active space (8 electrons, 7 orbitals) used for the TSH dynamics simulations (isovalue 0.05), including a additional σ/σ* orbitals.

The extended active space was further used for all TSH dynamics simulations. Additional simulations that tested the size of the model space showed ′numerical instability of the simulations below five electronic states. The resulting high computational cost of a large model space and active space limited the simulation time to 500 fs, which was, however, sufficient to deplete the S_1 to an MCH population below 1/e. In addition to the data presented in the manuscript, three measures were

collected: (1) total simulation time, (2) the fraction of trajectories aborting with an error termination and (3) the valid simulation time. For the latter, the upper limit is either a crash, normal termination or violation of criteria addressing energy conservation: A window within which fluctuations of the total energy are allowed of 0.25 eV, a maximum change in total energy per step of 0.15 eV, a maximum change in kinetic energy per step of 0.15 eV, a maximum change in potential energy per step of 0.15 eV and a maximum change in total energy per transition event of 1.2 eV. Premature normal termination after 100 fs in the ground state was counted as completion of the full simulation time. The full simulation duration was reached by 96% of trajectories, with a valid fraction of 90% and only 3 error terminations (Figure S4.5), attesting to the high quality of the results obtained.

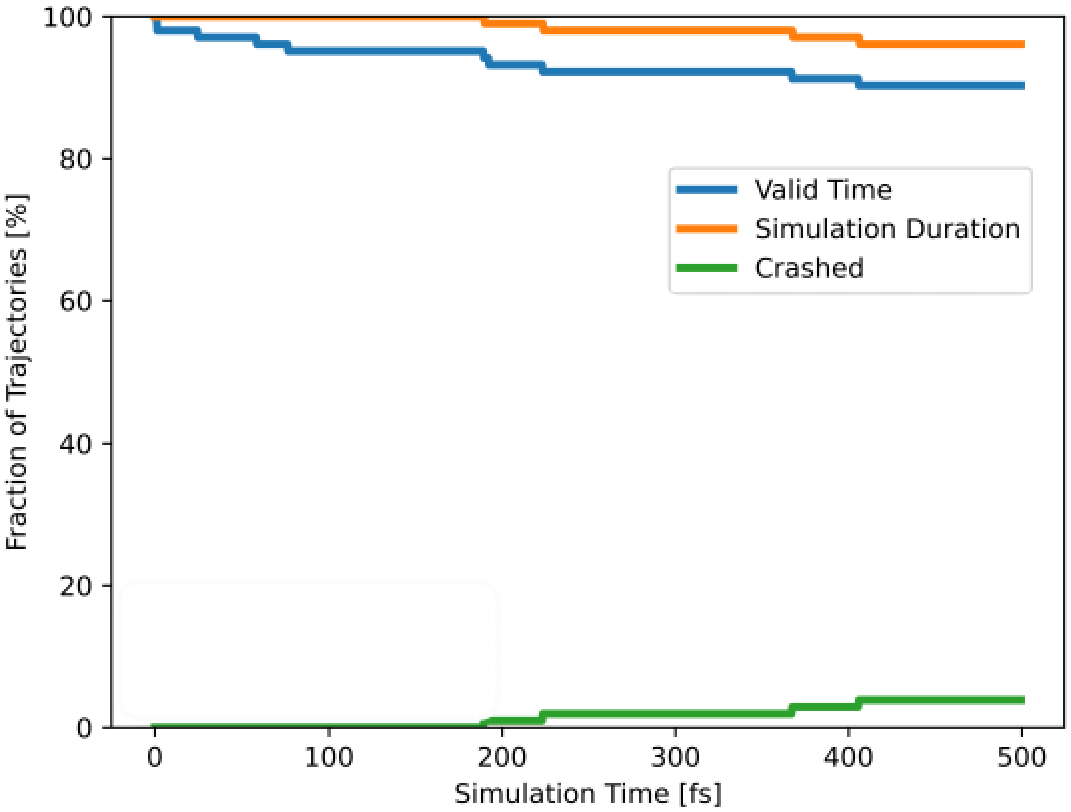

Figure S4.5. Decomposition of the simulation time for CHp with respect to valid time, simulated time and crashed trajectories as fractions of the ensemble.

All trajectories which underwent ISC into either the T_1 or T_2 during 500 fs (N = 66) received an extension of their simulation time until a continuous population of the T_1 was observed for 500 fs, i.e. the total simulation time in this subset varied from 501 to 1000 fs. All trajectories were analyzed with respect to two well-defined geometric measures. (1) The H-C=C-H dihedral of the olefinic moiety, measuring the activity of the OBF-ERC (2) Aliphatic reorganisation (True/False), which is true if at least one of the four C-C-C-C dihedrals of the aliphatic moiety (with IUPAC priorities: C3-

C4-C5-C6, C4-C5-C6-C7, C5-C6-C7-C1, C6-C7-C8-C9) changes by more than 60° over more than 20 fs. The results are summarized in Figure S4.6.

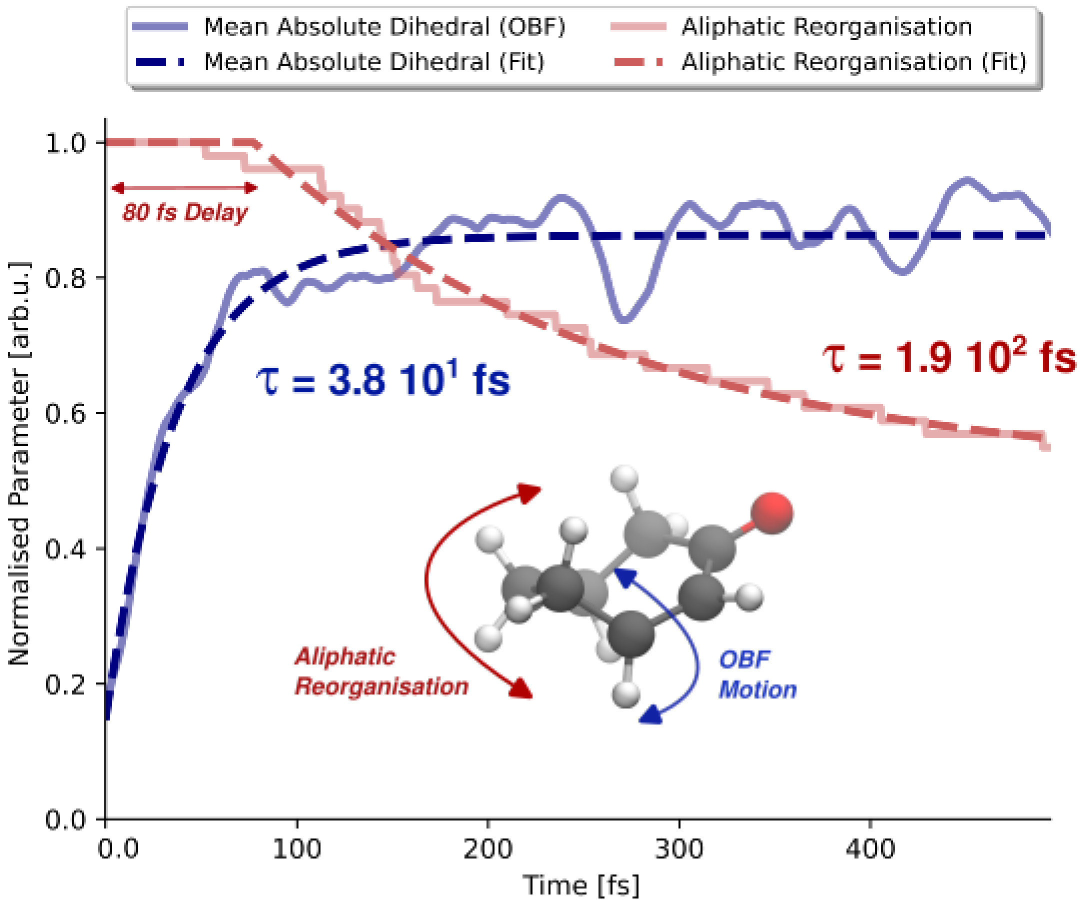

Figure S4.6. Summary of tracked geometric features, characterizing the two essential reorganization processes in CHp-Z2 upon photo-excitation: Aliphatic reorganization as an OBF motion, and aliphatic reorganisation by conformer redistribution. A marked timescale separation is evident.

Prior to ISC, no reorganization of the aliphatic moiety is observed. With a delay of about 80 fs, reorganization within the triplet manifold commences, which later is well-described as an exponential decay. We interpret this as the result of energy dissipation that deactivates the OBF motion and thus, prevents return to the planar configuration (see Figure 3 in the main text). The fitted reorganization rate of the aliphatic moiety is one order of magnitude lower than the mean change of the H-C=C-H dihedral (OBF motion). As such, the OBF motion out-competes the aliphatic reorganization. This implies that the direction of the OBF can be conformer-specific. As shown in Table S4.5, that is found to be true.

Table S4.5: Summary of enantiomer distribution between the OBF-configured and planar triplet minima of CHp after 500 fs stable population of the T1, per conformer.

Conformer	Ent-OBF	Planar	OBF
CHp-Z1	15	0	6
CHp-Z2	9	0	21
CHp-Z3	2	1	10

Since the simulations were performed in a standard orientation, these ratios approximate the intrinsic enantioselectivity induced by the side chain, i.e. the effect of a non-interacting chiral environment. The standard orientations were chosen to align with the chiral frame of the catalyst, such that we can infer a favoring of the OBF enantiomer already from the fixed aliphatic group within the catalyts' confinement.

Validation of the CC-pVDZ Basis Set for TSH Simulations

Due to the high cost, TSH dynamics simulations used the cc-pVDZ basis set. To validate the quality of the cc-pVDZ basis, we performed extensive benchmark calculations across multiple metrics. First, basis set convergence was investigated via the optimization of stationary points, restricted to the critical, early section of the OBF-ERC. A comparison of optimized geometries roughly judges the accuracy of gradients. Both **CHp** and **1** were treated. We restricted this comparison to the cc-pVTZ basis set, as larger basis sets were prohibitively expensive for compound **1**. The results are shown in Table S4.6, along with the relative energies at the same level of theory as the respective optimization.

Table S4.6: Accuracy of the cc-pVDZ basis set against the superior cc-pVTZ basis set with respect to optimized geometries (S0 minimum, $^1(n\pi^*)$ minimum, $^3(n\pi^*)$ minimum, $^3(n\pi^*)/^3(\pi\pi^*)$ minimum energy conical intersection and $^3(n\pi^*)/^1(\pi\pi^*)$ minimum energy intersystem crossing point) at the critical, inital section of the OBF coordinate for the dominant conformer of each model enone **CHp** and enone **1**. Relative energies are calculated against the ground state minimum. Both the root mean square deviation (RMSD) and maximum absolute deviation (MAD) are provided.

Metric	CHp		1	
	RMSD	MAD	RMSD	MAD
RMSD (Geometries) [pm]	5.9	12.6	2.3	2.9
Rel. Energies [meV] ([kJ/mol])	29 (2.8)	42 (4.0)	4 (0.4)	10 (1.0)

The RMSD errors for geometries and relative energies indicate close agreement between the cc-pVDZ and the superior cc-pVTZ basis set. Further, we systematically expanded the basis set size at the SAn-XMS-CASPT2(e/o)/cc-pVTZ optimized geometries of both **CHp** and **1**, using 10^{-9} Eh accurate Cholesky decomposition, a 10^{-9} Hartree energy converge threshold during the SA-CASSCF optimization and a 10^{-9} Hartree energy converge threshold during the second-order optimization (Figure S4.7 and Figure S4.8).

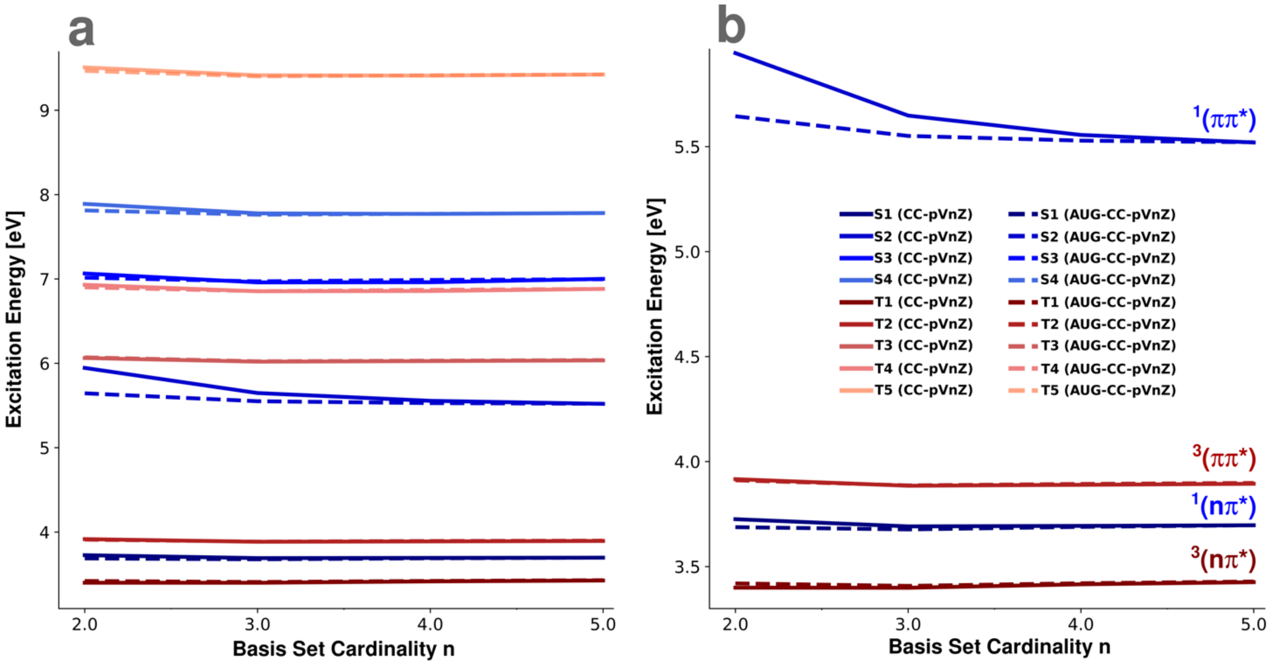

Figure S4.7. Systematic basis set expansion from cc-pVDZ (cardinality of 2) through to cc-pV5Z (cardinality of 5) for model compound **CHp**. Additionally, the corresponding basis sets with diffuse augmentation (aug-...) are shown as dashed lines. XMS-CASPT2(6/5) excitation energies for both the full model space of 5 states per manifold (a) and a zoom-in on the states discussed in the main text (b) are shown. Excellent convergence behavior is found in particular for low lying excited states.

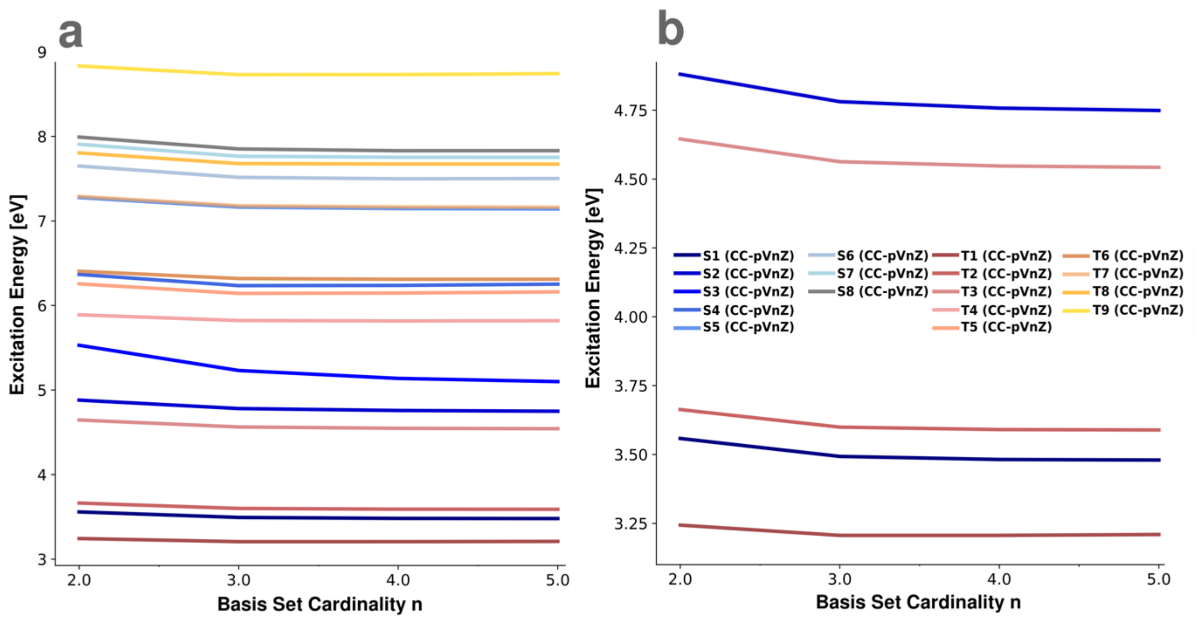

Figure S4.8. Systematic basis set expansion from cc-pVDZ (cardinality of 2) through to cc-pV5Z (cardinality of 5) on model compound **CHp**. XMS-CASPT2(10/8) excitation energies for both the full model space of 9 states per manifold (a) and a zoom-in on the states discussed in the main text (b) are shown. Excellent convergence behavior is found in particular for low lying excited states.

For both systems, the characters of the excited states at the FC point remain unaltered across the entire model space, i.e. no *root-flipping* is observed. Moreover, almost all states show rapid convergence behavior. The largest basis sets show meV-accuracy against the corresponding (n-1) cardinalities, such that we closely approach the complete basis set limit. Thus, we are able to estimate the basis set incompleteness error (BSIE) of cc-pVDZ to be in the low ceV order of magnitude (*vide infra*). A notable exception are singlet excited states with high contribution of pseudo-B_u-like configuration state functions. However, even for the largest basis sets, their energetic separation to the active excited states in TSH simulations is well above 1 eV, such that we can safely neglect a contribution of the latter. Finally, we studied the error along the IDPP-MEP approximation to the OBF-ERCs between the cc-pVDZ and the aug-cc-pV5Z basis set, which informs about the BSIE evolution upon excited state relaxation. The results are summarized in Figure S4.9 and statistical analysis is provided in Table S4.7.

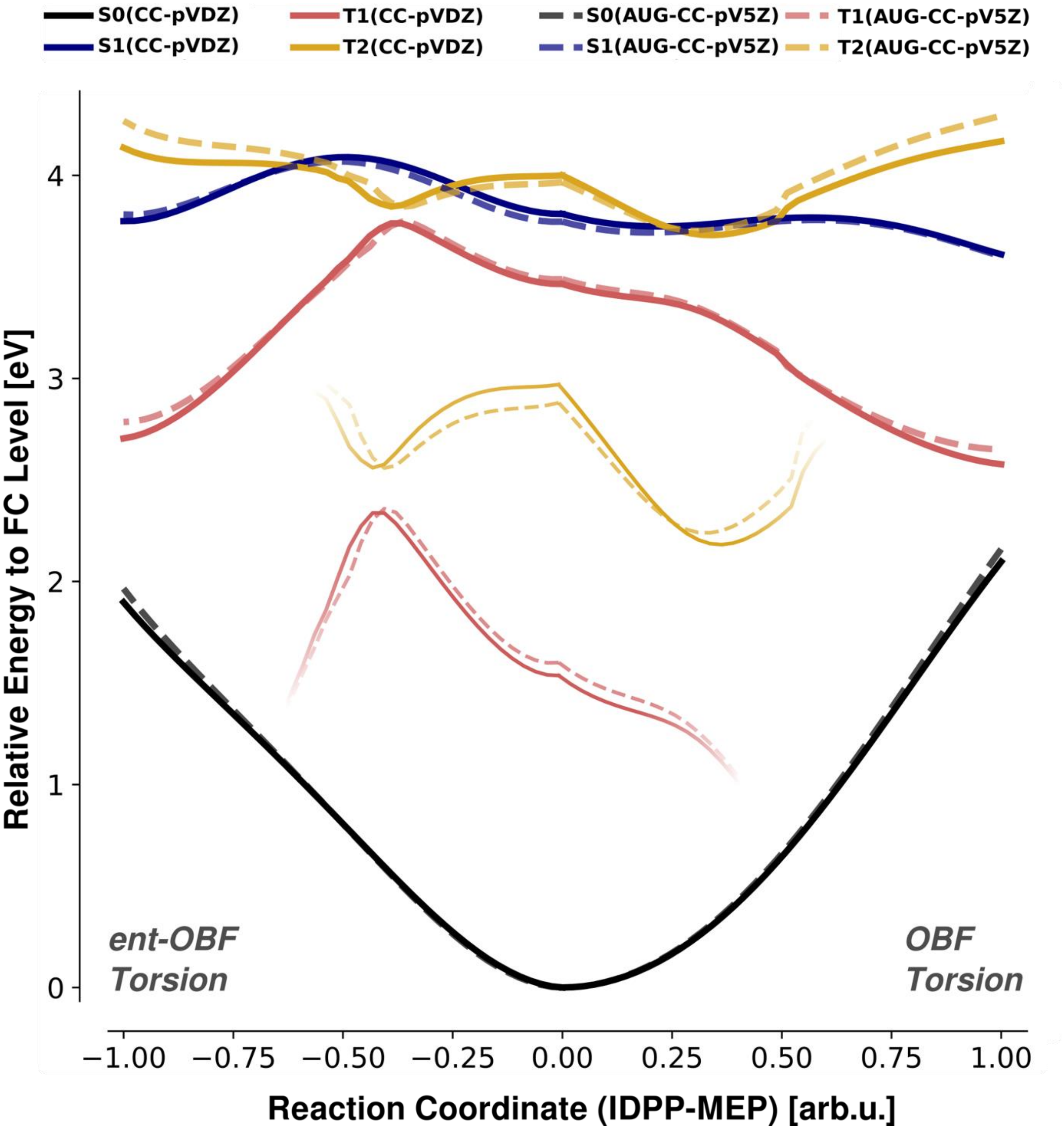

Figure S4.9. Comparison of SA5-XMS-CASPT2 energies relative to the FC point of the important singlet and triplet states between the cc-pVDZ (bold lines) and the very large aug-cc-pV5Z (faint dashed lines) basis sets along the IDPP-MEP approximation to the OBF-ERC, for both faces. The shown data is the genuine, un-shifted result without any applied scaling. Small deviations to Figure S4.7 are due to an accelerated basis set expansion protocol (cc-pVDZ to cc-pVQZ to aug-cc-pV5Z), highlighting that the ~10^{-2} eV errors observed are close to the fundamental accuracy of CASSCF-based *ab-initio* methods. The inlay (center) presents a zoom-in on the critical, central section of the triplet manifold surfaces. The S_1 surface appears asymmetric at this projection since the C-O bond elongation relevant for the ISC dynamics is neglected.

Table S4.7: Accuracy of the cc-pVDZ basis set against the superior aug-cc-pV5Z basis set along the IDPP-MEP approximation to the OBF-ERCs in both major (OBF) and minor (*ent*-OBF) torsional direction. Besides the total reaction coordinate, the central portion (coordinate –0.5 to +0.5) is separated out, where the decisive facial branching occurs. Chemical accuracy for relative energies (below 1 kcal/mol (4.2 kJ/mol)) is found in terms of basis set incompleteness.

**State**	**RMSD Energy (Total IDPP)**		**RMSD Energy (Central 50%)**	
	**[meV]**	**[kJ mol^{-1}]**	**[meV]**	**[kJ mol^{-1}]**
S_0	25	2.4	7	0.7
S_1	25	2.5	33	3.1
T_1	32	3.1	21	2.1
T_2	68	6.5	34	3.3
Total	41	4.0	26	2.5

The cc-pVDZ calculations recover the qualitative shape of the potential calculated with the larger basis. Further, near quantitative agreement with an RMSD of 2-3 kJ/mol during the decisive section of the ERC is found. As such, we attest *chemical accuracy* in terms of the BSIE. Finally, we checked the effect of scalar relativistic corrections by means of the Douglass-Kroll-Hess Hamiltonian [M. Reiher, A. Wolf, *J. Chem. Phys.*, **121** (2004) 10945–10956] and respective cc-pVnZ-DK (n = D,T,Q,5) basis sets, which were between 1-2 meV (0.1-0.2 kJ mol^{-1}), i.e. negligible. As such, we conclude that TSH dynamics simulations at the cc-pVDZ basis set size are adequate for the purposes of this work.

Excitation of the Adduct of 1 and 8a

The character of the initial excitation of the adduct between **1** and **8a** was characterized via the transition densities for the S_1 and S_2 of an RI-SCS-ωPBEPP86/Def2-TZVP (5 singlet states) singlepoint calculation. The analysis was performed with the THEODORE program. As depicted in Figure S4.10, they are confined to either the TX_1 or TX_2 site and resemble the HOMO/LUMO excitation of thioxanthone.

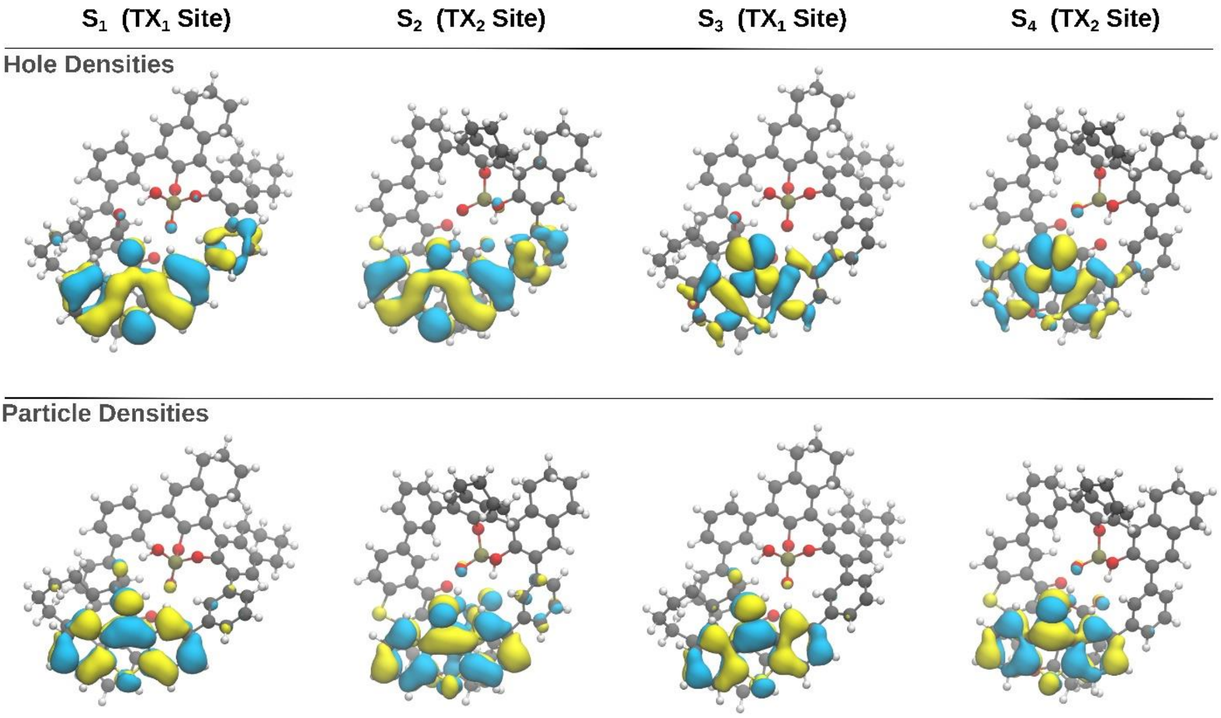

Figure S4.10. NTOs (isosurface 0.01) corresponding to hole and particle densities for the first four singlet excited states of the adduct between **8a** and **1**, which are well-localized on the respective thioxanthone groups TX_1 and TX_2.

Since previous publications on thioxanthone characterized its S_1 state as a single electron excitation, the use of TD-DFT is appropriate. Additional calculations including 20 singlet states at the Def2-SVP level of theory did not change the ordering of the lowest 4 excited states, which we thus take to be converged (Table S4.8).

Table S4.8. Characterization of the four lowest singlet excited states in the minimum free energy conformer of the adduct between substrate **1** and catalyst **8a** at the [SMD(dichloromethane)]-RI-SCS-wPBEPP86/Def2-TZVP//[CPCM(Dichloromethane)]-R^2SCAN-3C/Def2-mTZVPP level of theory. In addition, the ($n_O\pi^*$) state on the enone site is reported, which is blue shifted compared to the free enone on the same level of theory (*vide supra*).

State	Excitation Energy [eV]	Character	Site
S_1	3.458	$(\pi\ \pi^*)$ / $2A_1$	TX_1
S_2	3.474	$(\pi\ \pi^*)$ / $2A_1$	TX_2
S_3	3.675	$(n_{C0}\ \pi^*)$ / $1A_2$	TX_1
S_4	3.687	$(n_{CO}\ \pi^*)$ /$1A_2$	TX_2
S_n	4.380	$(n_O\ \pi^*)$	Enone

IDPP-MEP Interpolation

The excited state reaction pathways were studied along an IDPP approximation to the minimum energy path (MEP) as implemented in ORCA v5.0.3. This approach was chosen due to its very low computational cost and ability to handle torsional motions, which characterize the OBF-ERC.[42] Despite being an approximate technique, we consider it suitable for the qualitative description of excited state reaction coordinates. For each system, CHp and the adduct between **1** and **8a**, two interpolations were performed. Starting from the S_0 minimum optimized on R²SCAN-3c/Def2-mTZVPP level of theory towards the immediately-related OBF and ent-OBF triplet minima optimized on UR²SCAN-3C/Def2-mTZVPP level of theory. All calculations were conducted in gas phase. Starting geometries for the immediately-related OBF triplet minima were obtained from relaxed scans of the H-C=C-H dihedral torsion (90°, 20 steps) for the lowest energy S_0 conformer, using the first excited state at the TD-DFT ωB97X-D3/Def2-SVP level of theory. The end points were subjected to optimization at the UKS-R^{2}SCAN-3C level of theory. The latter was necessary due to convergence problems of UR²SCAN-3C near the T_1T_2-CoIn.

For each face, 38 interpolation points were used. For CHp, energies along the IDPP-MEP were obtained from singlepoint calculations on three levels of theory: SA5-XMS-CASPT2(8/7)/cc-pVDZ, matching the level of theory used in the TSH dynamics. In addition, two DFT-based approximations were screened (Figure S4.11):

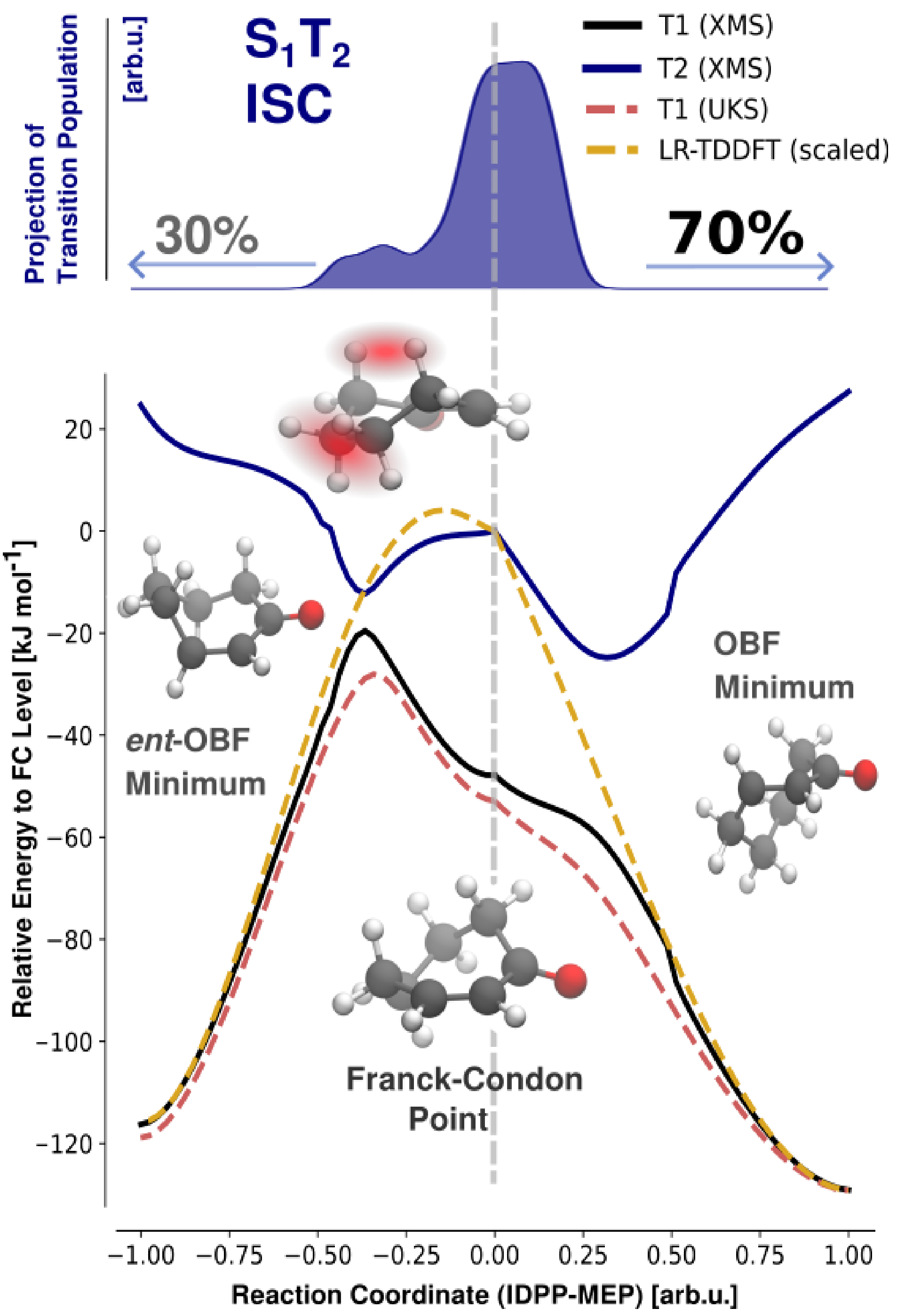

Figure S4.11. Fate of the chiral conformer CHp-2 formed by ISC during the surface hopping trajectories dynamics of cyclohept-2-enone (CHp). Top: Gaussian-broadened distribution of geometries at the time point of the S_1 - T_2 surface hop, projected onto a vector capturing the essential motions along the *ent*-OBF/OBF reaction coordinate. The two maxima roughly correspond to the position of the S_1T_2 crossing seam on either side. Bottom: Excited state energy landscape along an IDPP-MEP reaction coordinate from the FC geometry to either the **OBF** or *ent*-**OBF** T_1 minima. Adiabatic surfaces T_1 and T_2 (black, blue) are evaluated at the XMS-CASPT2/cc-pVDZ level of theory. Also displayed are the UKS-R^{2}SCAN-3c/def2-mTZVPP (UKS, red) and RI-SCS-

ωPBEPP86/de2-TZVP (TDDFT, golden) approximations to the T_1 adiabate and $^3(\pi\pi^*)$ diabate, respectively. The observed splitting favoring the **OBF** minimum becomes plausible from an early barrier along the *ent*-**OBF** reaction pathway (center) on all surfaces, the red shading of methylene groups (molecular inlay) highlights intramolecular repulsion between the reorganizing aliphatic methylene groups.

UR²SCAN-3C/Def2-mTZVPP appeared as a reasonable approximation to the PES of the T_1 adiabatic state. In addition, we tested a TD-DFT description with MP2 ground state energy correction and perturbative doubles excited state energy correction, at the RI-SCS-ωPBEPP86/Def2-TZVP level of theory. It was found that the latter method produces a spurious ordering of the excited states during the TD-DFT part of the calculation, such that the $^3(\pi\pi^*)$ state localized on the enone is always lowest in energy, behaving rather like a diabatic state. We exploited this behavior to approximate the critical $^3(\pi\pi^*)$ diabatic surface of the large adduct between enone **1** and catalyst **8a** (Figure 6 of the main text), for which a higher-order level of theory was not feasible. The triplet excited states were obtained from a TD-DFT calculation on a closed-shell singlet reference, which provides a better description of the $^3(\pi\pi^*)$ in the FC region than a triplet reference state. However, this does not hold near the OBF geometry due to strong multi-reference character of the proper wavefunction. As such, this Ansatz gradually degrades along the reaction coordinate, but we are primarily interested in the early part of the reaction coordinate.

For the representation of the S_1T_2 transition population, a principal component analysis was performed over the joint set of OBF and ent-OBF IDPP pathways, using in-house-developed software. This afforded a vector of 89% explained variances, onto which the S1T2 transition geometries from the TSH dynamics simulations were projected and scaled to match the projection of interpolated points. Both forward and backward transitions within the MCH representation were included, and Gaussian-broadened (FWHM = 0.1 arb.u.). For the adduct between **1** and **8a**, it is assumed that the results on the CHp model system hold, such that the XMS-CASPT2 evaluations were omitted. All excited state surfaces were shifted to match the respective XMS-CASPT2 energies at the FC point. The RI-SCS-ωPBEPP86 energies were scaled to match the XMS-CASPT2 energy at the OBF minimum. The energy decomposition was performed as described in the original publication.[43] Excited states were treated by evaluating the full system and fragment where the excitation is localized at the denoted excited state method, while the catalyst fragment was always treated on the corresponding ground state method, i.e. UKS-R²SCAN-3C/Def2-mTZVPP(enone, full system) + R²SCAN-3C/mTZVPP(catalyst) or TDA-TDDFT RI-SCS-ωPBEPP86/Def2-SVP (enone, full system) + RI-SCS-ωPBEPP86/Def2-SVP (catalyst). The excitation along the OBF coordinate is

well-localized on the enone fragment, such that the fragment decomposition does not cut through the exciton.

Ground State Reactions – Supporting Analysis

After a detailed treatment of the excited state selectivity factor, it remained to determine the selectivity effects of the equilibrium ground state dynamics [cf. factor (iii)]. We reduced the computational effort by performing a high-level assay on the achiral ground state reaction network of compounds **2**/*ent*-**2** (detailed technical description below). Identifying the critical reaction barriers, we then narrowed the treatment of the dimeric adduct between **2**/*ent*-**2** and **8a** to those decisive stages only. We differentiate between productive reaction coordinates (PC), which afford **3e**/*ent*-**3e**, and loss channels (LC), along which the back-isomerization to **1** occurs. The productive coordinate comprises two scenarios (Figure S4.12a):

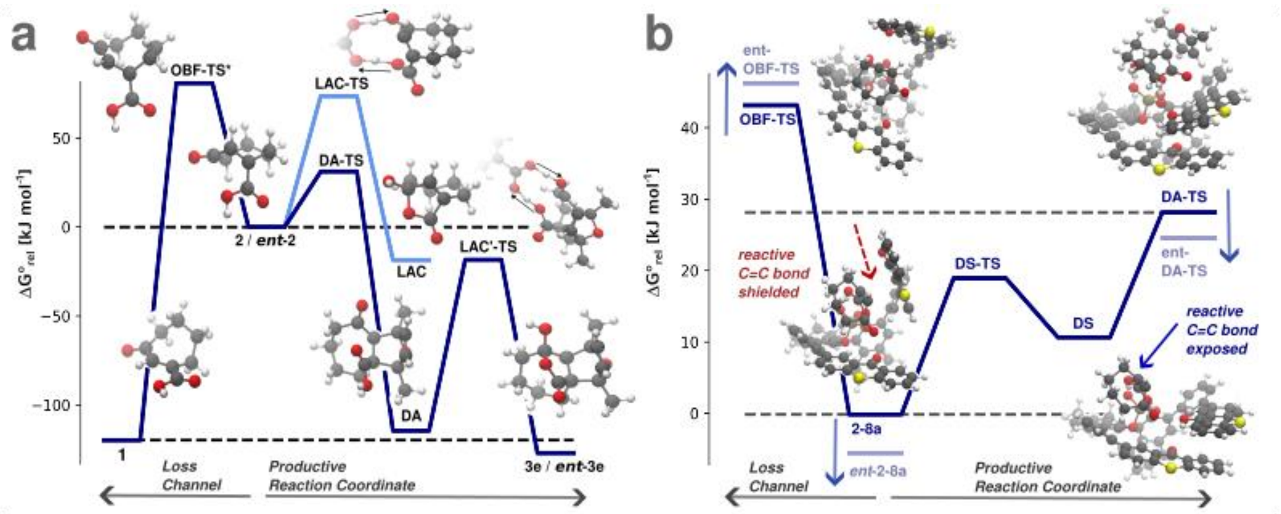

Figure S4.12. Computed ground state reaction pathways relative to the photochemically generated **2**/*ent*-**2** species with 2,3-dimethylfuran as the reaction partner. The productive reaction coordinate describes the conversion to species **3e**, whilst the loss channel describes the back-isomerisation to **1**. a racemic reaction in the absence of the catalyst. The mechanism involving an early, irreversible Diels-Alder reaction emerges as favored, identifying the relative barrier height between the OBF- and Diels-Alder-transition states as critical to the ground state selectivity. b Investigation of the critical section along the reaction coordinates for the catalyzed reaction. The catalyst adds complexity due to the interconversion between a stable, shielded and unstable, de-shielded configuration. Comparing the major (dark blue) with the minor (faint blue) enantiomers, a weak ground state selectivity opposing the photochemical selectivity is suggested.

(PC1) Initial intramolecular cyclization to an intermediate lactone **LAC**/*ent*-**LAC**, followed by Diels-Alder reaction to **3**/*ent*-**3** and (PC2), the reverse reaction sequence of Diels-Alder reaction followed by intramolecular cyclisation. The loss channel (LC) is a unimolecular isomerization via the S_0-OBF transition state (**OBF-TS**). The reaction network obtained shows that the intramolecular cyclization toward **LAC**/*ent*-**LAC** and **3e**/*ent*-**3e** (Figure S4.12a) proceed via proton transfer catalysis within a dimer of two carboxylic acids, that are associated with high barriers. In contrast, the Diels-Alder reaction of PC2 is nearly barrier-less and out-competes LC and PC1. The **DA**/*ent*-**DA** intermediates thus formed are highly exergonic species and interception of **2**/*ent*-**2** is irreversible. The results thus suggest that the decisive stage of the reaction is the competition of the **OBF-TS** against the **DA-TS**. Final cyclisation over a thermally accessible barrier affords **3e**/*ent*-**3e** in a net exergonic cascade. In the reaction catalyzed by **8a** (Figure S4.12b), the reorganization dynamics of both TX-groups increase the complexity of the productive reaction coordinate. In the immediate photoproduct complex, the reactive C=C bond is shielded by one TX arm, and a shallow barrier needs to be overcome to expose it to the diene reaction partner in a high-energy configuration (**DS**/*ent*-**DS**). This extends the lifetime of the *trans*-configured intermediate prior to irreversible DA interception. Therefore, we assume that PC2 and LC become competitive. In contrast, the LC pathway is assumed to be insensitive to the catalytic environment, due to its low sterical demand. Under this assumption, the decisive barriers (**DA-TS**/*ent*-**DA-TS**, **OBF-TS**/*ent*-**OBT-TS**) for both enantiomeric channels can be compared. We find that the minor *ent*-**2**-**8a** network appears to be stabilized along the productive coordinate and de-stabilized along the loss channel. While the calculated free energy differences of the barriers should be considered with some care, the findings suggest that the equilibrium ground state selectivity (cf. (iii)) is opposed to the photochemical selectivity of the reaction (cf. (i)). This could explain the erratic trend observed for varying diene reaction partners (Scheme 4) and the relatively low overall enantioselectivity. The final product **3e**/*ent*-**3e** is sterically bulky and thus, interacts poorly with the catalyst, such that it is readily exchanged for **1**. The event closes the catalytic cycle and guarantees a reasonable turnover. In the overall view, we conclude that the prevalence of **3e** over *ent*-**3e** is a result of the excited state process.

Ground State Reactions – Computational Details

All barriers documented in the main text, as well as below, are between Boltzmann-weighed ground state conformer ensembles and the lowest transition state along a given reaction coordinate. All minima and transition states along the ground state reaction pathways were subjected to meta-dynamics conformer sorting and ensemble optimization (vide supra) and the energies reported in the main text refer to Boltzmann-weighed sums on the DLPNO-CCSD(T)/CBS//R^2SCAN-3C/mTZVPP

level of theory. Exceptions are the following transition states: For the non-catalyzed reaction (Figure S4.12a), the transition states LAC-TS and LAC'-TS were optimized from the dimer of **2** and DA with the minimum energy conformer of **1**, restrained to a configuration where its carboxy group bridges the carboxy-oxygen and carbonyl-oxygen positions of the substrate, via two hydrogen bonds. All other acidic species could also act as catalysts, but their effect on the reaction barrier is deemed negligible. For the S_0-OBF-TS (Figure S4.12a), the final single point energy evaluation was performed on the SA6-XMS-CASPT2(10/8)/cc-pVTZ level of theory to account for the multi-reference character of the wavefunction. For alignment, the conformer ensemble of **2** was re-evaluated at this level of theory and the S_0 transition state shifted to match the XMS-CASPT2//R^2SCAN-3C barrier between them. Likewise, the species DA-TS and DA were aligned with the Boltzmann sum of **2** by equating the latter to the Boltzmann sum of the lowest 20 conformers of the Van-der-Waals adduct between the diene and **2**. The same alignment was performed for the species LAC-TS and LAC, using the 20 lowest energy conformers of the Van-der-Waals adduct between **1** and **2**. The same alignment was performed for the species LAC'-TS and **3e**, using the 20 lowest energy conformers of the Van-der-Waals adduct between **1** and DA. The OBF-TS within the catalyst **8a** adduct (Figure S4.12b and Figure 7) was not refined and instead, the shifting performed at the R^2SCAN-3C level of theory. The reported barrier height is thus a lower boundary, as DFT methods are well-known to underestimate bond-breaking barriers.

For the transition state of the de-shielding reorganization (Figure S4.12b and Figure 7)), the full meta-dynamics-based sampling was omitted due to its computational complexity. Instead, a single NEB transition state optimization was performed, between the lowest energy conformers found via the proper conformer screening, for the adduct placing the enone within (**2**-**8a**) and outside (DS) of the sandwich pocket. It stands to reason that this should correspond to the lowest de-shielding transition state, via the Hammond postulate. Except for the DA-TS species, all adducts in Figure 7b were treated without the diene. To align the free energies, a single XTB meta-dynamics simulation was performed for the Van-der-Waals adduct between the diene and **2**-**8a** as well as *ent*-**2**-**8a** adducts, and the lowest energy conformer at the XTB level of theory subjected to level 2b optimization and level 3 refinement, which energy was set equal to the Boltzmann sum of sandwich-configured sub-ensemble **2**-**8a** and *ent*-**2**-**8a** species. This approximation was necessary since this system has the highest degrees of freedom and weakest intramolecular interaction of all species treated in this work. Due to the latter, all conformers of this adduct will have very similar Boltzmann weights, such that this decision does not affect the accuracy of the results. This rationalization was confirmed previously for a very similar catalyst, placing the gross free energy error below 1 kJ/mol (unpublished).

IR spectra reported in Figure 1 of the main text were calculated from harmonic analytical frequency analyses on the Boltzmann sum of **1** and **2** at the R^2SCAN-3C level of theory. The spectrum was obtained as the sum over the individual, Gaussian-broadened (FWHM = 40 1/cm) Franck-Condon points.

5. Substrate Synthesis

Methyl cyclohept-1-ene-1-carboxylate (S1)

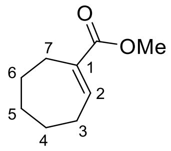

$C_9H_{14}O_2$
154.21 g/mol

According to a modified literature procedure:[44,45] A solution of $NaHSO_3$ (21.4 g, 206 mmol, 2.06 eq.) in water (50 mL) was added dropwise at −21 °C to a suspension of NaCN (10.1 g, 206 mmol, 2.06 eq.) and cycloheptanone (11.2 g, 11.8 mL, 100 mmol, 1.00 eq.) in water (42 mL). The resulting white suspension was stirred for 4 h at room temperature. Et_2O (50 mL) was added, the phases were separated, and the aqueous phase was extracted with Et_2O (3 × 50 mL). The combined organic extracts were dried over Na_2SO_4, filtered and the solvent was removed under reduced pressure.

The crude cyanohydrine was dissolved in PhMe (22 mL) and pyridine (48 mL), and $POCl_3$ (41.4 g, 25.2 mL, 270 mmol, 2.70 eq.) was added dropwise at 0 °C. After complete addition, the reaction mixture was heated at 95 °C for 1 h and subsequently poured into ice (500 mL). The phases were separated, and the aqueous phase was extracted with Et_2O (3 × 100 mL). The organic phases were washed with 1 N HCl_{aq} (1 × 100 mL) and water (1 × 100 mL), dried over Na_2SO_4 and filtered. The solvent was removed under reduced pressure and the obtained crude nitrile was used without further purification in the next step.

The crude nitrile was dissolved in MeOH (100 mL) and H_2SO_4 (20 mL) was slowly added at 0 °C. The resulting mixture was heated at 120 °C for 72 h and the reaction progress was monitored by TLC. Upon complete conversion of the nitrile, the phases were separated, and the aqueous phase was extracted with CH_2Cl_2 (3 × 100 mL). The combined organic phases were washed with 5% $NaHCO_{3aq}$ (1 × 100 mL), dried over Na_2SO_4, filtered and the solvent was removed under reduced pressure. Fractionated distillation under reduced pressure (2 mbar, 87 °C) yielded Ester **S1** as a colorless liquid (12.1 g, 78.5 mmol, 79 %).

B.p.: 87 °C (2 mbar).

^{1}H NMR (500 MHz, $CDCl_3$, 300 K): δ [ppm] = 7.16 (t, ^3J = 6.7 Hz, 1H, C2-H), 3.71 (s, 3H, OCH_3), 2.49 – 2.54 (m, 2H, C7-H), 2.24 – 2.32 (m, 2H, C3-H), 1.74 – 1.80 (m, 2H, C5-H), 1.48 – 1.56 (m, 4H, C4-H, C6-H).

^{13}C NMR (101 MHz, $CDCl_3$, 300 K): δ [ppm] = 168.9 (s, COO), 144.7 (d, C2), 136.6 (s, C1), 51.9 (q, OCH_3), 32.2 (t, C5), 29.0 (t, C3), 27.6 (t, C7), 26.4 (t, C4), 25.9 (t, C6).

Analytical data matched those previously reported in the literature.[45]

Methyl 3-oxocyclohept-1-ene-1-carboxylate (S2)

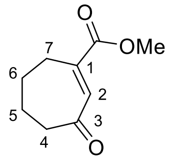

$C_9H_{12}O_3$
168.19 g/mol

According to a modified literature procedure:[46] **S1** (2.00 g, 13.0 mmol, 1.00 eq.), 20% $Pd(OH)_2/C$ (344 mg, 0.648 mmol Pd, 5 mol%), and K_2CO_3 (448 mg, 3.24 mmol, 0.250 eq.) were suspended in CH_2Cl_2 (40 mL) and the reaction flask was sealed with a rubber septum and a ballon under air (during the reaction oxygen evolution was observed). A 5.5 M solution of TBHP in Decane (5.84 g, 11.8 mL, 64.9 mmol, 5.00 eq.) was added dropwise at 0 °C and the resulting suspension was stirred for 19 h at room temperature. The suspension was filtered over *Celite*® and the solvent was removed under reduced pressure. Column chromatography (silica, Hex/EtOAc = 9/1) yielded the oxidized ester **S2** (412 mg, 2.45 mmol, 19%) as a colorless liquid.

TLC: R_f = 0.44 (Hex/EtOAc = 8/2) [UV/$KMnO_4$].

^{1}H NMR (500 MHz, $CDCl_3$, 300 K): δ [ppm] = 6.89 (s, 1H, C2-H), 3.80 (s, 3H, OCH_3), 2.69 – 2.76 (m, 2H, C7-H), 2.62 – 2.68 (m, 2H, C4-H), 1.79 – 1.89 (m, 4H, C5-H, C6-H).

^{13}C NMR (126 MHz, $CDCl_3$, 300 K): δ [ppm] = 204.6 (s, C3), 168.2 (s, COO), 144.8 (s, C1), 137.4 (d, C2), 52.9 (q, OCH_3), 42.8 (t, C4), 27.7 (t, C7), 25.1 (t, C6), 21.6 (t, C5).

IR (ATR): ṽ (cm^{-1}) = 2950 (m), 2869 (m), 1717 (s, C=O), 1671 (s), 1453 (m), 1177 (s), 916 (m).

HRMS (ESI): calc. for [M + H^+]: 169.0865; found: 169.0864.

3-Oxocyclohept-1-ene-1-carboxylic acid (1)

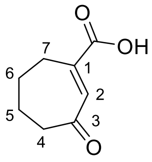

$C_8H_{10}O_3$
154.16 g/mol

According to a modified literature procedure:[5] Oxidized ester **S2** (856 mg, 5.09 mmol, 1.00 eq.) was suspended in water (6.2 mL) and Na_2CO_3 (647 mg, 6.11 mmol, 1.20 eq.) was added portion-wise. After stirring at room temperature for 24 h, 1 N HCl_{aq} was added until pH = 1 was reached and the reaction mixture was extracted with CH_2Cl_2 (3 × 10 mL). The combined extracts were dried over Na_2SO_4, filtered and the solvent was removed under reduced pressure. Column chromatography (silica, Hex/EtOAc = 8/2) yielded the carboxylic acid **1** (424 mg, 2.75 mmol, 54%) as a colorless solid.

TLC: R_f = 0.20 (Hex/EtOAc = 92/8) [UV/$KMnO_4$].

M.p.: 55 °C

^{1}H NMR (500 MHz, $CDCl_3$, 300 K): δ [ppm] = 7.02 (s, 1H, C2-H), 2.73 (t, ^3J = 6.2 Hz, 2H, C7-H), 2.68 (t, ^3J = 6.5 Hz, 2H, C4-H), 1.82 – 1.90 (m, 4H, C5-H, C6-H).

^{13}C NMR (126 MHz, $CDCl_3$, 300 K): δ [ppm] = 204.6 (s, C3), 172.6 (s, COOH), 143.7 (s, C1), 139.0 (d, C2), 42.8 (t, C4), 27.4 (t, C7), 25.0 (t, C6), 21.7 (t, C5).

IR (ATR): ṽ (cm^{-1}) = 2941 (m), 2894 (m), 1713 (s, C=O), 1634 (s), 1181 (s), 1154 (s), 761 (s).

HRMS (ESI): calc. for [M + H$^+$]: 155.0708; found: 155.0709.

Benzyl 3-oxocyclohept-1-ene-1-carboxylate (S3)

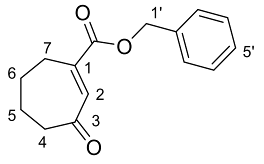

$C_{15}H_{16}O_3$
244.29 g/mol

According to a modified literature procedure: Carboxylic acid **1** (50.0 mg, 324 µmol, 1.00 eq.) was dissolved in DMF (13 mL), K_2CO_3 (67.2 mg, 486 µmol, 1.50 eq.) and BnBr (111 mg, 77.2 µL, 649 µmol, 2.00 eq.) were added, and the obtained suspension was stirred for 1.5 h. Water (15 mL) was added, the phases were separated, and the aqueous phase was extracted with Et_2O (3 × 10 mL). The combined organic extracts were washed with $NaCl_{aq}$ (1 × 10 mL), dried over Na_2SO_4, filtered, and the solvent was removed under reduced pressure. Column chromatography (silica, Hex/EtOAc = 9/1) yielded the benzylated product **S3** (61.1 mg, 250 µmol, 77%) as a colorless oil.

TLC: R_f = 0.17 (Hex/EtOAc = 90/10) [UV/$KMnO_4$].

^{1}H NMR (300 MHz, $CDCl_3$, 300 K): δ [ppm] = 7.32 – 7.46 (m, 5H, C3'-H, C4'-H, C5'-H), 6.76 – 7.02 (m, 1H, C2-H), 5.23 (s, 2H, C1'-H), 2.69 – 2.81 (m, 2H, C7-H), 2.59 – 2.68 (m, 2H, C4-H), 1.78 – 1.91 (m, 4H, C5-H, C6-H).

^{13}C NMR (101 MHz, $CDCl_3$, 300 K): δ [ppm] = 204.4 (s, C3), 167.6 (s, COO), 144.8 (s, C1), 137.5 (d, C2), 135.5 (s, C2'), 128.8 (d, C4'), 128.6 (d, C5'), 128.4 (d, C3'), 67.6 (t, C1'), 42.9 (t, C4), 27.9 (t, C7), 25.2 (t, C6), 21.7 (t, C5).

IR (ATR): ṽ (cm^{-1}) = 3035 (w), 1713 (s, C=O), 1670 (s), 1455 (m), 1234 (s), 1172 (s), 696 (s).

HRMS (ESI): calc. for [M + H^+]: 245.1178; found: 245.1172.

6. Synthesis of Phosphoric Acid Catalysts

Symmetric H8-BINOL Based Catalysts

2-(4,4,5,5-Tetramethyl-1,3,2-dioxaborolan-2-yl)-9*H*-thioxanthen-9-one (S4)

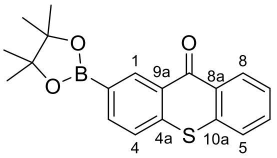

$C_{19}H_{19}BO_3S$
338.23 g/mol

According to a literature procedure:[47] 2-Bromo-9*H*-thioxanthen-9-one (1.00 g, 3.43 mmol, 1.00 eq.), B_2Pin_2 (1.12 g, 4.40 mmol, 1.28 eq.) and KOAc (2.16 g, 22.0 mmol, 6.40 eq.) were suspended in 1,4-dioxane (34 mL) and $Pd(PPh)_3Cl_2$ (121 mg, 172 µmol, 5 mol%) was added. After heating at 100 °C for 22 h, the brown reaction mixture was filtered over *Celite*® and the solvent was removed under reduced pressure. The crude product was washed with hexane (5 × 10 mL) and dried under high vacuum to afford borylated compound **S4** (925 mg, 2.74 mmol, 80%) as a yellow solid.

TLC: R_f = 0.54 (Hex/EtOAc = 70/30) [UV].

^{1}H NMR (500 MHz, $CDCl_3$, 300 K): δ [ppm] = 9.06 (d, ^4J = 1.4 Hz, 1H, C1-H), 8.63 (dd, ^3J = 8.2 Hz, ^4J = 1.5 Hz, 1H, C8-H), 7.98 (dd, ^3J = 8.1 Hz, ^4J = 1.4 Hz, 1H, C3-H), 7.62 (ddd, ^3J = 8.3 Hz, ^3J = 6.9 Hz, ^4J = 1.5 Hz, 1H, C6-H), 7.55 – 7.59 (m, 2H, C4-H, C5-H), 7.49 (ddd, ^3J = 8.2 Hz, ^3J = 6.9 Hz, ^4J = 1.4 Hz, 1H, C7-H), 1.37 (s, 12H, CH_3).

^{13}C NMR (101 MHz, $CDCl_3$, 300 K): δ [ppm] = 180.0 (s, C=O), 140.3 (s, C4a), 137.7 (d, C3), 137.1 (s, C10a), 137.0 (d, C1), 132.4 (d, C6), 130.1 (d, C8), 129.8 (s, C8a), 128.6 (s, C9a), 126.5 (d, C7), 126.1 (d, C5), 125.4 (d, C4), 84.3 (s, O*C*[CH_3]$_2$), 25.0 (q, OC[*C*H_3]$_2$).§

§The signal of C2 was not detectable by ^{13}C NMR.

Analytical data matched those previously reported in the literature.[47]

2-(3-(4,4,5,5-Tetramethyl-1,3,2-dioxaborolan-2-yl)phenyl)-9*H*-thioxanthen-9-one (S5)

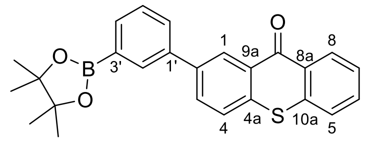

$C_{25}H_{23}BO_3S$
414.33 g/mol

According to a literature procedure:[47] 2-(3-Bromophenyl)-9*H*-thioxanthen-9-one (1.00 g, 2.72 mmol, 1.00 eq.), B_2Pin_2 (885 mg, 3.49 mmol, 1.28 eq.) and KOAc (1.71 g, 17.4 mmol, 6.40 eq.) were suspended in 1,4-dioxane (27 mL) and $Pd(PPh)_3Cl_2$ (95.6 mg, 136 µmol, 5 mol%) was added. After heating at 95 °C for 16 h, the brown reaction mixture was filtered over *Celite*® and the solvent was removed under reduced pressure. The crude product was washed with hexane (5 × 10 mL) and dried under high vacuum to afford borylated compound **S5** (957 mg, 2.30 mmol, 85%) as a yellow solid.

TLC: R_f = 0.58 (Hex/EtOAc = 70/30) [UV].

M.p.: > 230 °C

^{1}H NMR (400 MHz, $CDCl_3$, 300 K): δ [ppm] = 8.90 (d, ^4J = 2.1 Hz, 1H, C1-H), 8.65 (dd, ^3J = 8.0 Hz, ^4J = 1.5 Hz, 1H, C8-H), 8.11 – 8.22 (m, 1H, C2'-H), 7.89 – 7.94 (m, 1H, C3-H), 7.84 (dt, ^3J = 7.3 Hz, ^4J = 1.3 Hz, 1H, C4'-H), 7.80 (ddd, ^3J = 7.8 Hz, ^4J = 2.1 Hz, ^4J = 1.3 Hz, 1H, C6'-H), 7.56 – 7.66 (m, 3H, C4-H, C5-H, C6-H), 7.45 – 7.53 (m, 2H, C5'-H, C7-H), 1.38 (s, 12H, CH_3).

^{13}C NMR (101 MHz, $CDCl_3$, 300 K): δ [ppm] = 180.0 (s, C=O), 139.3 (s, C1'), 138.8 (s, C2), 137.2 (s, C10a)**, 136.0 (s, C4a)**, 134.3 (d, C4'-H), 133.4 (d, C2'), 132.3 (d, C_{Ar}), 131.2 (d, C3), 130.0 (d, C8), 129.9 (d, C6'-H), 129.4 (s, C9a)*, 129.2 (s, C8a)*, 128.4 (d, C_{Ar}), 127.9 (d, C1), 126.5 (d, C_{Ar}) 126.3 (d, C_{Ar}), 126.1 (d, C_{Ar}), 84.0 (s, *C*CH$_3$), 24.9 (q, C*C*H$_3$).§

*/** Assignments are interconvertible.

§The signal of C3' was not detectable by ^{13}C NMR.

IR (ATR): ṽ (cm^{-1}) = 2977 (w, C-H), 1638 (s, C=O), 1592 (s, C=C), 1318 (s), 1143 (s), 742 (s), 706 (m).

HRMS (ESI): calc. for [M + H$^+$]: 415.1534; found: 415.1529.

(*R*)-2,2'-(2,2'-Dihydroxy-5,5',6,6',7,7',8,8'-octahydro-[1,1'-binaphthalene]-3,3'-diyl)bis(9*H*-thioxanthen-9-one) (S6)

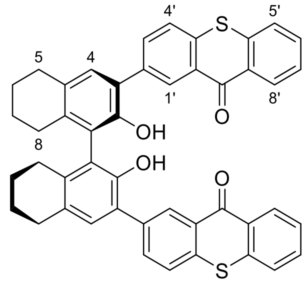

$C_{46}H_{34}O_4S_2$
714.89 g/mol

According to a literature procedure:[48] (*R*)-3,3'-Diiodo-5,5',6,6',7,7',8,8'-octahydro-[1,1'-binaphthalene]-2,2'-diol (200 mg, 366 µmol, 1.00 eq.) and borylated thioxanthone **S4** (310 mg, 915 µmol, 2.50 eq.) were dissolved in DME (3.7 mL), cataCXium® A (6.56 mg, 18.3 µmol, 5 mol%) and $Pd(OAc)_2$ (3.29 mg, 14.7 µmol, 4 mol%) were added. Subsequently, 1 M K_2CO_{3aq} (507 mg, 3.66 mL, 3.66 mmol, 10.0 eq.) was added and the reaction mixture was heated at 80 °C for 22 h. After the indicated reaction time CH_2Cl_2 was added and the phases were separated. The aqueous phase was extracted with CH_2Cl_2 (3 × 10 mL), the combined organic extracts were washed with saturated NH_4Cl_{aq} (1 × 20 mL) and water (1 × 20 mL) and were dried over Na_2SO_4. After filtration and removal of the solvent under reduced pressure, purification by column chromatography (silica, CH_2Cl_2 → CH_2Cl_2/Ac = 95/5) yielded the cross coupled product **S6** (177 mg, 248 µmol, 68%) as a yellow solid.

TLC: R_f = 0.62 (CH_2Cl_2) [UV/$KMnO_4$].

M.p.: 225 °C

^{1}H NMR (400 MHz, $CDCl_3$, 300 K): δ [ppm] = 8.84 (d, ^4J = 2.0 Hz, 2H, C1'-H), 8.64 (dd, ^3J = 8.1 Hz, ^4J = 1.4 Hz, 2H, C8'-H), 7.97 (dd, ^3J = 8.4 Hz, ^4J = 2.1 Hz, 2H, C3'-H), 7.56 – 7.65 (m, 6H, C4'-H, C5'-H, C6'-H), 7.48 (ddd ^3J = 6.4 Hz, ^3J = 8.1 Hz, ^4J = 1.9 Hz, 2H, C7'-H), 7.27 – 7.30 (m, 2H, C4-H), 5.04 (br s, 2H, OH), 2.72 – 2.89 (m, 4H, C5-H), 2.37 – 2.48 (m, 2H, C8-H^a), 2.19 – 2.33 (m, 2H, C8-H^b), 1.69 – 1.84 (m, 8H, C6-H, C7-H).

^{13}C NMR (101 MHz, $CDCl_3$, 300 K): δ [ppm] = 180.1 (s, 2 × C=O), 148.6 (s, 2 × C2), 137.5 (s, 2 × C2'), 137.4 (s, 2 × C4a), 136.6 (s, 2 × C-10a'), 135.9 (s, 2 × C4a'), 133.7 (d, 2 × C3'), 132.3 (d, 2 × C6'), 132.2 (d, 2 × C4), 131.0 (s, 2 × C8a), 130.1 (d, 2 × C1'), 130.1 (d, 2 × C8'), 129.4 (s,

2 × C9’a), 129.3 (s, 2 × C9’), 126.4 (d, 2 × C7’), 126.2 (d, 2 × C4’), 125.9 (d, 2 × C5’), 124.8 (s, 2 × C3), 119.8 (s, 2 × C1), 29.4 (t, 2 × C5), 27.4 (t, 2 × C8), 23.1 (t, 2 × C6), 23.1 (t, 2 × C7).

IR (ATR): ṽ (cm^{-1}) = 3357 (w, O-H), 2929 (m), 1635 (s, C=O), 1591 (s, C=C), 1457 (s), 1079 (m), 743 (s, sp^2-CH).

HRMS (ESI): calc. for [M + Na^+]: 737.1791; found: 737.1793.

Specific Rotation: $[\alpha]_D^{25} = -138$ (c = 1.0, $CHCl_3$).

2,2'-((11*bR*)-4-Hydroxy-4-oxido-8,9,10,11,12,13,14,15-octahydrodinaphtho[2,1-*d*:1',2'-*f*][1,3,2]dioxaphosphepine-2,6-diyl)bis(9*H*-thioxanthen-9-one) (8b)

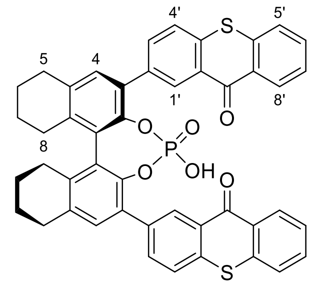

$C_{46}H_{33}O_6PS_2$
776.86 g/mol

According to a modified literature procedure:[49] Diol **S6** (131 mg, 183 µmol, 1.00 eq.) was dissolved in pyridine (4.3 mL) and $POCl_3$ (56.2 mg, 34.3 µL, 366 µmol, 2.00 eq.) was added dropwise at room temperature. The obtained yellow reaction solution was stirred at 60 °C for 19 h. Subsequently, water (4.3 mL) was added at room temperature and the reaction mixture was heated at 100 °C for 3 h. The reaction mixture was cooled back to room temperature and 6 N HCl_{aq} (8.3 mL) was added, followed by heating to 100 °C for 1 h. The reaction mixture was extracted with CH_2Cl_2 (3 × 15 mL) and the combined organic extracts were dried over Na_2SO_4, filtered and the solvent was removed under reduced pressure. Purification by Column chromatography (silica, CH_2Cl_2/MeOH = 95/5) yielded a yellow solid which was redissolved in CH_2Cl_2 (20 mL) and washed with 6 N HCl_{aq} (1 × 15 mL). Drying of the organic phase over Na_2SO_4, filtration and removal of all solvents under reduced pressure yielded phosphoric acid **8b** (115 mg, 148 µmol, 81%) as a yellow solid.

TLC: R_f = 0.19 (CH_2Cl_2/MeOH = 95/5) [UV].

M.p.: >230 °C

^{1}**H NMR** (400 MHz, DMSO-d_6, 300 K): δ [ppm] = 8.59 (d, ^{4}J = 2.0 Hz, 2H, C1'-H), 8.48 (dd, ^{3}J = 8.2 Hz, ^{4}J = 1.5 Hz, 2H, C8'-H), 8.14 (dd, ^{3}J = 8.5 Hz, ^{4}J = 2.1 Hz, 2H, C3'-H), 7.82 – 7.92 (m, 4H, C4'-H, C5'-H), 7.78 (ddd, ^{3}J = 8.2 Hz, ^{3}J = 7.0 Hz, ^{4}J = 1.5 Hz, 2H, C6'-H), 7.59 (ddd, ^{3}J = 8.2 Hz, ^{3}J = 7.0 Hz, ^{4}J = 1.3 Hz, 2H, C7'-H), 7.31 – 7.41 (m, 2H, C4-H), 2.81 – 2.99 (m, 4H, C5-H), 2.63 – 2.78 (m, 2H, C8-H^{a}), 2.18 – 2.37 (m, 2H, C8-H^{b}), 1.75 – 1.90 (m, 6H, C6-H, C7-H^{a}), 1.50 – 1.67 (m, 2H, C7-H^{b}).

^{13}**C NMR** (126 MHz, DMSO-d_6, 300 K): δ [ppm] = 178.8 (s, 2 × C=O), 143.6 (d, $^{2}J_{C\text{-}P}$ = 9.4 Hz, 2 × C2), 137.5 (s, 2 × C_{Ar}), 136.5 (s, 2 × C_{Ar}), 135.8 (s, 2 × C_{Ar}), 135.5 (s, 2 × C_{Ar}), 134.8 (s, 2 × C_{Ar}), 134.1 (d, 2 × C3'), 133.1 (d, 2 × C6'-H), 130.7 (d, 2 × C4), 129.6 (d, $^{3}J_{C\text{-}P}$ = 3.2 Hz, 2 × C3), 129.4 (d, 2 × C1'), 129.2 (d, 2 × C8'), 128.3 (s, 2 × C_{Ar}), 128.3 (s, 2 × C_{Ar}), 127.5 (s, 2 × C_{Ar}), 126.9 (d, 2 × C7'), 126.7 (d, 2 × C_{Ar}), 126.5 (d, 2 × C_{Ar}), 28.5 (t, 2 × C5), 27.5 (t, 2 × C8), 22.2 (t, 2 × C6), 22.1 (t, 2 × C7).

^{31}**P NMR** (162 MHz, DMSO-d_6, 300 K): δ [ppm] = −1.43.

IR (ATR): ṽ (cm^{-1}) = 1635 (m, C=O), 1593 (m, C=C), 1438 (m), 1215 (s), 1078 (m), 742 (s, sp^{2}-CH).

HRMS (ESI): calc. for [M + H^{+}]: 777.1529; found: 777.1533.

Specific Rotation: $[\alpha]_D^{25}$ = −204 (c = 1.0, $CHCl_3$).

(*R*)-2,2'-((2,2'-Dihydroxy-5,5',6,6',7,7',8,8'-octahydro-[1,1'-binaphthalene]-3,3'-diyl)bis(3,1-phenylene))bis(9*H*-thioxanthen-9-one) (S7)

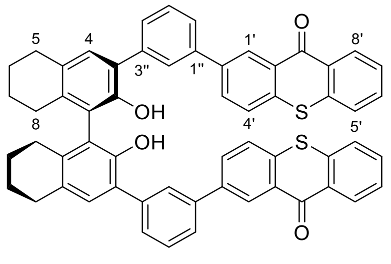

$C_{58}H_{42}O_4S_2$
867.09 g/mol

According to a literature procedure:[48] (*R*)-3,3'-Diiodo-5,5',6,6',7,7',8,8'-octahydro-[1,1'-binaphthalene]-2,2'-diol (400 mg, 732 µmol, 1.00 eq.) and borylated thioxanthone **S5** (759 mg, 1.83 mmol, 2.50 eq.) were dissolved in DME (7.3 mL), cataCXium® A (13.1 mg, 36.6 µmol, 5 mol%) and $Pd(OAc)_2$ (6.58 mg, 29.3 µmol, 4 mol%) were added. Subsequently, 1 M K_2CO_{3aq} (1.01 g, 7.32 mL, 7.32 mmol, 10.0 eq.) was added and the reaction mixture was heated at 80 °C for 18 h.

After the indicated reaction time CH_2Cl_2 was added and the phases were separated. The aqueous phase was extracted with CH_2Cl_2 (3 × 20 mL), the combined organic extracts were washed with saturated NH_4Cl_{aq} (1 × 40 mL) and water (1 × 40 mL) and were dried over Na_2SO_4. After filtration and removal of the solvent under reduced pressure, purification by column chromatography (silica, Hex/EtOAc 7/3 → CH_2Cl_2) yielded the cross coupled product **S7** (487 mg, 562 μmol, 77%) as a yellow solid.

TLC: R_f = 0.65 (CH_2Cl_2) [UV/$KMnO_4$].

M.p.: >230 °C

^{1}H NMR (400 MHz, $CDCl_3$, 300 K): δ [ppm] = 8.89 (d, ^4J = 2.1 Hz, 2H, C1'-H), 8.62 (d, ^3J = 8.2 Hz, 2H, C8'-H), 7.94 – 8.04 (m, 2H, C2''-H), 7.89 (dd, ^3J = 8.3 Hz, ^4J = 2.1 Hz, 2H, C3'-H), 7.39 – 7.68 (m, 14H, C4'-H, C5'-H, C6'-H, C7'-H, C4''-H, C5''-H, C6''-H), 7.21 – 7.33 (m, 2H, C4-H), 5.08 (br s, 2H, OH), 2.71 – 2.90 (m, 4H, C5-H), 2.43 – 2.54 (m, 2H, C8-H^a), 2.25 – 2.37 (m, 2H, C8-H^b), 1.65 – 1.85 (m, 8H, C6-H, C7-H).

^{13}C NMR (101 MHz, $CDCl_3$, 300 K): δ [ppm] = 180.1 (s, 2 × C=O), 148.4 (s, 2 × C2), 139.7 (s, 2 × C_{Ar}), 139.5 (s, 2 × C_{Ar}), 138.9 (s, 2 × C_{Ar}), 137.3 (s, 2 × C_{Ar}), 137.1 (s, 2 × C_{Ar}), 136.2 (s, 2 × C_{Ar}), 132.4 (d, 2 × C_{Ar}), 132.0 (d, 2 × C4), 131.3 (d, 2 × C3'), 130.6 (s, 2 × C_{Ar}), 130.0 (d, 2 × C8'), 129.5 (s, 2 × C_{Ar}), 129.3 (s, 2 × C_{Ar}), 129.1 (d, 2 × C_{Ar}), 128.9 (d, 2 × C_{Ar}), 128.3 (d, 2 × C2''), 128.0 (d, 2 × C1'), 126.6 (d, 2 × C_{Ar}), 126.4 (d, 2 × C_{Ar}), 126.1 (d, 2 × C_{Ar}), 125.9 (d, 2 × C_{Ar}), 120.2 (s, 2 × C1), 29.4 (t, 2 × C5), 27.4 (t, 2 × C8), 23.2 (t, 2 × C6), 23.2 (t, 2 × C7).[§]

[§]One of the aromatic carbon atoms was not detected by ^{13}C NMR, presumably due to signal overlap.

IR (ATR): ṽ (cm^{-1}) = 3367 (br, O-H), 2928 (w, C-H), 1725 (s, C=O), 1591 (s, C=C), 1438 (m), 1121 (w), 704 (w).

HRMS (ESI): calc. for [M + H^+]: 867.2598; found: 867.2607.

Specific Rotation: $[\alpha]_D^{25}$ = – 66 (c = 1.0, $CHCl_3$).

2,2'-(((11*bR*)-4-Hydroxy-4-oxido-8,9,10,11,12,13,14,15-octahydrodinaphtho[2,1-*d*:1',2'-*f*][1,3,2]dioxaphosphepine-2,6-diyl)bis(3,1-phenylene))bis(9*H*-thioxanthen-9-one) (8a)

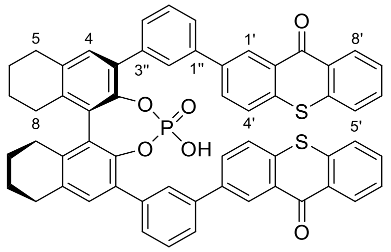

$C_{58}H_{41}O_6PS_2$
929.05 g/mol

According to a modified literature procedure:[49] Diol **S7** (422 mg, 487 µmol, 1.00 eq.) was dissolved in pyridine (11.5 mL) and $POCl_3$ (149 mg, 91.0 µL, 973 µmol, 2.00 eq.) was added dropwise at room temperature. The obtained yellow reaction solution was stirred at 60 °C for 17 h. Subsequently, water (11.5 mL) was added at room temperature and the reaction mixture was heated at 100 °C for 3 h. The reaction mixture was cooled back to room temperature and 6 N HCl_{aq} (22 mL) was added, followed by heating to 100 °C for 1 h. The reaction mixture was extracted with CH_2Cl_2 (3 × 30 mL) and the combined organic extracts were dried over Na_2SO_4, filtered and the solvent was removed under reduced pressure. Purification by column chromatography (silica, CH_2Cl_2/MeOH = 95/5) yielded a yellow solid which was redissolved in CH_2Cl_2 (40 mL) and washed with 6 N HCl_{aq} (1 × 30 mL). Drying of the organic phase over Na_2SO_4, filtration and removal of all solvents under reduced pressure yielded phosphoric acid **8a** (345 mg, 371 µmol, 76%) as a yellow solid.

TLC: R_f = 0.18 (CH_2Cl_2/MeOH = 95/5) [UV].

M.p.: >230 °C

^{1}H NMR (400 MHz, DMSO-d_6, 300 K): δ [ppm] = 8.73 (d, ^4J = 2.2 Hz, 2H, C1'-H), 8.47 (d, ^3J = 8.2 Hz, ^4J = 1.5 Hz, 2H, C8'-H), 8.18 (dd, ^3J = 8.5 Hz, ^4J = 2.2 Hz, 2H, C3'-H), 8.10 – 8.13 (m, 2H, C2''-H), 7.90 (d, ^3J = 8.5 Hz, 2H, C4'-H), 7.83 (d, ^3J = 7.9 Hz, 2H, C_{Ar}-H), 7.69 – 7.79 (m, 6H, C6'-H, C_{Ar}-H, C_{Ar}-H), 7.52 – 7.62 (m, 4H, C7'-H, C_{Ar}-H), 7.28 – 7.36 (m, 2H, C4-H), 2.79 – 2.96 (m, 4H, C5-H), 2.61 – 2.74 (m, 2H, C8-H^a), 2.18 – 2.33 (m, 2H, C8-H^b), 1.74 – 1.86 (m, 6H, C6-H, C7-H^a), 1.49 – 1.65 (m, 2H, C7-H^b).

^{13}C NMR (126 MHz, DMSO-d_6, 300 K): δ [ppm] = 179.2 (s, 2 × C=O), 144.2 (d, $^2J_{C\text{-}P}$ = 9.3 Hz, 2 × C2), 138.9 (s, 2 × C_{Ar}), 138.7 (s, 2 × C_{Ar}), 138.7 (s, 2 × C_{Ar}), 137.3 (s, 2 × C_{Ar}), 136.9 (s, 2 × C_{Ar}),

136.1 (s, 2 × C_{Ar}), 134.7 (s, C_{Ar}), 133.5 (d, C_{Ar}), 132.0 (d, 2 × C3'), 131.4 (d, 2 × C4), 131.2 (d, $^{3}J_{C-P}$ = 3.4 Hz, 2 × C3), 129.7 (d, 2 × C_{Ar}), 129.6 (d, 2 × C_{Ar}), 129.5 (d, 2 × C_{Ar}), 129.1 (s, 2 × C_{Ar}), 128.7 (s, 2 × C_{Ar}), 128.4 (d, 2 × C2''), 127.9 (d, 2 × C4'), 127.3 (d, 2 × C_{Ar}), 127.1 (d, 2 × C_{Ar}), 126.9 (d, 2 × C1'), 125.9 (d, 2 × C_{Ar}), 29.0 (t, 2 × C5), 27.9 (t, 2 × C8), 22.7 (t, 2 × C6), 22.7 (t, 2 × C7).§

§One of the aromatic carbon atoms was not detected by ^{13}C NMR, presumably due to signal overlap.

^{31}P NMR (162 MHz, DMSO-d_6, 300 K): δ [ppm] = −1.18.

IR (ATR): ṽ (cm^{-1}) = 2932 (w), 1638 (s, C=O), 1592 (s, C=C), 1439 (m), 1017 (m), 744 (s, sp^2-CH).

HRMS (ESI): calc. for [M + H^+]: 929.2155; found: 929.2163.

Specific Rotation: $[\alpha]_D^{25} = -168$ (c = 1.0, $CHCl_3$).

Non-symmetric H8-BINOL based catalyst 8c

3-(3-((11*cR*)-6-(3,5-Di-tert-butylphenyl)-4-hydroxy-4-oxido-8,9,10,11,12,13,14,15-octahydrodinaphtho[2,1-*d*:1',2'-*f*][1,3,2]dioxaphosphepin-2-yl)phenyl)-9*H*-thioxanthen-9-one (8c)

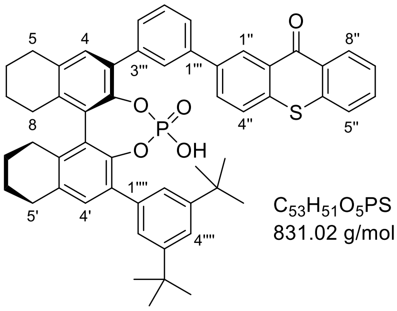

According to a modified literature procedure:[48,49] (*R*)-3,3'-Diiodo-5,5',6,6',7,7',8,8'-octahydro-[1,1'-binaphthalene]-2,2'-diol (1.00 g, 1.83 mmol, 1.00 eq.) and (3,5-di-*tert*-butylphenyl)boronic acid (429 mg, 1.83 mmol, 1.00 eq.) were dissolved in DME (18 mL), cataCXium® A (32.8 mg, 91.5 µmol, 5 mol%) and $Pd(OAc)_2$ (16.4 mg, 73.2 µmol, 4 mol%) were added. Subsequently, 1 M K_2CO_{3aq} (2.53 g, 18.3 mL, 18.3 mmol, 10.0 eq.) was added and the reaction mixture was heated at 80 °C for 19 h. After the indicated reaction time CH_2Cl_2 was added and the layers were separated. The aqueous phase was extracted with CH_2Cl_2 (3 × 30 mL), the combined organic extracts were washed with saturated NH_4Cl_{aq} (1 × 70 mL) and water (1 × 70 mL) and were dried over Na_2SO_4. After filtration and removal of the solvent under reduced pressure, purification by column chromatography (silica, Hex/EtOAc 99/1) yielded a mixture of the mono- and homo-di-cross coupled

product (56/44 mono/homo-di, 541 mg, equaling 275 mg, 452 µmol, 25% of mono) as a beige foam, which was directly used in the next step.

A part of the obtained product mixture (462 mg, 56/44 mono/homo-di, equaling 235 mg, 386 µmol, 1.00 eq. of mono) was reacted as previously described with borylated thioxanthone **S5** (160 mg, 386 µmol, 1.00 eq.), cataCXium® A (6.92 mg, 19.3 µmol, 5 mol%), $Pd(OAc)_2$ (3.46 mg, 15.4 µmol, 4 mol%) and 1 M K_2CO_{3aq} (533 mg, 3.86 mL, 3.86 mmol, 10.0 eq.) in DME (3.9 mL). Purification by column chromatography (silica, Hex → Hex/EtOAc 95/5) yielded the hetero-di-cross-coupled diol (201 mg, 261 µmol, 68%) as a yellow solid. The compound contained some aliphatic impurities and was therefore not completely characterized.

The hetero-di-cross-coupled diol (198 mg, 258 µmol, 1.00 eq.) was dissolved in pyridine (6.1 mL) and $POCl_3$ (79.0 mg, 48.2 µL, 515 µmol, 2.00 eq.) was added dropwise at room temperature. The obtained reaction solution was stirred at 60 °C for 20 h. Subsequently, water (6.1 mL) was added at room temperature and the reaction mixture was heated at 100 °C for 3 h. The reaction mixture was cooled back to room temperature and 6 N HCl_{aq} (11.7 mL) was added, followed by heating to 100 °C for 1 h. The reaction mixture was extracted with CH_2Cl_2 (3 × 20 mL) and the combined organic extracts were dried over Na_2SO_4, filtered and the solvent was removed under reduced pressure. Purification by column chromatography (silica, CH_2Cl_2/MeOH = 97/3) yielded a yellow solid which was redissolved in CH_2Cl_2 (25 mL) and washed with 6 N HCl_{aq} (1 × 15 mL). Drying of the organic phase over Na_2SO_4, filtration and removal of all solvents under reduced pressure yielded phosphoric acid **8c** (193 mg, 232 µmol, 90%) as a yellow solid.

TLC: R_f = 0.21 (CH_2Cl_2/MeOH = 95/5) [UV].

M.p.: > 230 °C

^{1}H NMR (500 MHz, DMSO-d_6, 300 K): δ [ppm] = 8.74 (d, ^4J = 2.2 Hz, 1H, C1"-H), 8.49 (dd, ^3J = 8.1 Hz, ^4J = 1.5 Hz, 1H, C8"-H), 8.17 (dd, ^3J = 8.5 Hz, ^4J = 2.2 Hz, 1H, C3"-H), 8.01 – 8.06 (m, 1H, C2'''-H), 7.95 (d, ^3J = 8.5 Hz, 1H, C4"-H), 7.85 – 7.89 (m, 1H, C_{Ar}-H), 7.76 – 7.81 (m, 2H, C6"-H, C_{Ar}-H), 7.66 – 7.74 (m, 1H, C_{Ar}-H), 7.56 – 7.62 (m, 2H, C7"-H, C_{Ar}-H), 7.53 (d, ^4J = 1.9 Hz, 2H, C2''''-H), 7.35 (*virt.* t, $^4J \approx {}^4J$ = 1.9 Hz, 1H, C4''''-H), 7.34 (s, 1H, C4'-H), 7.19 (s, 1H, C4-H), 2.76 – 2.93 (m, 4H, C5-H, C5'-H), 2.60 – 2.71 (m, 2H, C8-H^a, C8'-H^a), 2.11 – 2.34 (m, 2H, C8-H^b, C8'-H^b), 1.70 – 1.85 (m, 6H, C6-H, C6'-H, C7-H^a, C7'-H^a), 1.50 – 1.65 (m, 2H, C7-H^b, C7'-H^b), 1.30 (s, 18H, $C[CH_3]_3$).

^{13}C NMR (126 MHz, DMSO-d_6, 300 K): δ [ppm] = 178.8 (s, C=O), 149.8 (s, 2 × C3’’’’), 143.4 (d, $^2J_{C-P}$ = 9.5 Hz, C2*), 143.3 (d, $^2J_{C-P}$ = 9.5 Hz, C2’*), 138.5 (s, C_{Ar}), 138.4 (s, C_{Ar}), 138.1 (s, C_{Ar}), 137.0 (s, C_{Ar}), 136.5 (s, C_{Ar}), 136.3 (s, C_{Ar}), 135.8 (s, C_{Ar}), 134.5 (s, C_{Ar}), 134.2 (s, C_{Ar}), 133.1 (d, C_{Ar}), 131.8 (d, $^3J_{C-P}$ = 3.5 Hz, C3**), 131.5 (d, C3’’), 131.1 (d, C4’), 131.0 (d, C4), 130.7 ($^3J_{C-P}$ = 3.0 Hz, C3’**), 129.3 (d, C_{Ar}), 129.2 (d, C_{Ar}), 129.1 (d, C3’’), 128.7 (s, C_{Ar}), 128.3 (s, C_{Ar}), 127.9 (d, C2’’’), 127.5 (s, C_{Ar}), 127.3 (d, C4’’), 127.0 (s, C_{Ar}), 127.0 (s, C_{Ar}), 127.0 (d, C_{Ar}), 126.7 (d, C_{Ar}), 126.5 (d, C1’’), 125.6 (d, C_{Ar}), 123.9 (d, 2 × C2’’’’), 120.5 (d, C4’’’’), 34.7 (s, 2 × C[CH_3]$_3$), 31.4 (q, 2 × C[CH_3]$_3$), 28.6 (t, C5***), 28.5 (t, C5’***), 27.5 (t, C_{Alkyl}), 27.4 (t, C_{Alkyl}), 22.3 (t, C_{Alkyl}), 22.3 (t, C_{Alkyl}), 22.2 (t, C_{Alkyl}), 22.2 (t, C_{Alkyl}).

*/**/*** The assignments are interconvertible.

^{31}P NMR (162 MHz, DMSO-d_6, 300 K): δ [ppm] = −1.39.

IR (ATR): ṽ (cm^{-1}) = 2952 (s, C-H), 1641 (m, C=O), 1594 (s, C=O), 1439 (m), 1019 (s), 748 (s).

HRMS (ESI): calc. for [M + Na^+]: 853.3088; found: 853.3071.

Specific Rotation: $[\alpha]_D^{25}$ = − 188 (c = 1.0, $CHCl_3$).

SPINOL based Catalyst 9

2,2'-((7,7'-Bis(methoxymethoxy)-2,2',3,3'-tetrahydro-1,1'-spirobi[indene]-6,6'-diyl)bis(3,1-phenylene))bis(9*H*-thioxanthen-9-one) (S8)

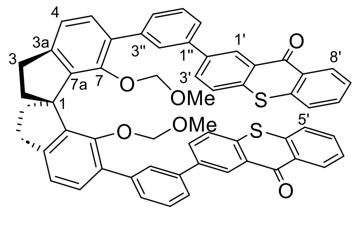

$C_{59}H_{44}O_6S_2$
913.12 g/mol

According to a modified literature procedure:[7] (*R*)-6,6'-Diiodo-7,7'-bis(methoxymethoxy)-2,2',3,3'-tetrahydro-1,1'-spirobi[indene] (240 mg, 405 µmol, 1.00 eq.) and borylated thioxanthone **S5** (420 mg, 1.01 mmol, 1.50 eq.) were dissolved in THF (12 mL) and MeOH (0.5 mL). [Pd(PPh_3)]$_4$ (70.3 mg, 60.8 µmol, 15 mol%) and 1 M K_2CO_{3aq} (336 mg, 1.22 mL, 2.43 mmol, 6.00 eq.) were added and the reaction mixture was heated at 75 °C for 16 h. After the indicated reaction time CH_2Cl_2 was added and the phases were separated. The organic phase was washed with saturated $NaCl_{aq}$ (1 × 20 mL) and dried over Na_2SO_4. After filtration and removal of the solvent under reduced

pressure, purification by column chromatography (silica, $CH_2Cl_2 \rightarrow CH_2Cl_2$/Ac = 90/10) yielded the catalyst precursor **S8** (132 mg, 145 µmol, 36%) as a yellow solid.

TLC: R_f = 0.41 (CH_2Cl_2) [UV/$KMnO_4$].

M.p.: 151 °C

^{1}H NMR (400 MHz, $CDCl_3$, 300 K): δ [ppm] = 8.85 (d, ^4J = 2.1 Hz, 2H, C1'-H), 8.52 (dd, ^3J = 8.1 Hz, ^4J = 1.5 Hz, 2H, C8'-H), 7.95 – 8.05 (m, 2H, C2''-H), 7.77 (dd, ^3J = 8.4 Hz, ^4J = 2.1 Hz, 2H, C3'-H), 7.61 – 7.67 (m, 2H, C6''-H), 7.36 – 7.58 (m, 12H, C1'-H, C5'-H, C6'-H, C7'-H, C4''-H, C5''-H), 7.28 (d, ^3J = 7.6 Hz, 2H, C5-H), 7.11 (d, ^3J = 7.6 Hz, 2H, C4-H), 4.43 (d, ^2J = 5.4 Hz, 2H, C*H*HOCH$_3$), 4.34 (d, ^2J = 5.4 Hz, 2H, CH*H*OCH$_3$), 3.03 – 3.24 (m, 4H, C3-H), 2.79 (s, 6H, CHHOC*H*$_3$), 2.45 – 2.64 (m, 2H, C2-H^a), 2.30 – 2.43 (m, 2H, C2-H^b).

^{13}C NMR (101 MHz, $CDCl_3$, 300 K): δ [ppm] = 179.8 (s, 2 × C=O), 151.9 (s, 2 × C7), 145.5 (s, 2 × C3a), 142.5 (s, 2 × C7a), 140.4 (s, 2 × C3''), 139.4 (s, 2 × C1''), 139.1 (s, 2 × C2'), 137.1 (s, 2 × C10'a), 136.0 (s, 2 × C4'a), 132.5 (s, 2 × C6), 132.1 (d, 2 × C_{Ar}), 130.8 (d, 2 × C3'), 130.3 (d, 2 × C5), 129.8 (d, 2 × C8'), 129.4 (s, 2 × C_{Ar}), 129.2 (s, 2 × C_{Ar}), 129.0 (d, 2 × C_{Ar}), 128.6 (d, 2 × C_{Ar}), 127.9 (d, 2 × C2''), 127.5 (d, 2 × C1'), 126.5 (d, 2 × C_{Ar}), 126.2 (d, 2 × C_{Ar}), 126.0 (d, 2 × C_{Ar}), 125.5 (d, 2 × C6''), 120.4 (d, 2 × C4), 98.7 (t, 2 × *C*HHOCH$_3$), 59.9 (s, C1), 56.5 (q, 2 × CHHO*C*H$_3$), 39.2 (t, 2 × C2), 31.2 (t, 2 × C3).

IR (ATR): ṽ (cm^{-1}) = 2937 (w), 1638 (s, C=O), 1592 (s, C=C), 1459 (m), 1439 (m), 1162 (m), 746 (s, sp^2-CH).

HRMS (ESI): calc. for [M + Na^+]: 935.2472; found: 935.2473.

Specific Rotation: $[\alpha]_D^{25}$ = 246 (c = 1.0, $CHCl_3$).

2,2'-((12-Hydroxy-12-oxido-4,5,6,7-tetrahydrodiindeno[7,1-*de*:1',7'-*fg*][1,3,2]dioxaphosphocine-1,10-diyl)bis(3,1-phenylene))bis(9*H*-thioxanthen-9-one) (9)

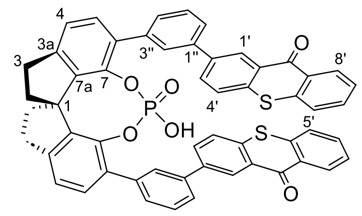

$C_{55}H_{35}O_6PS_2$
886.97 g/mol

According to a modified literature procedure:[49] Catalyst precursor **S8** (117 mg, 128 µmol, 1.00 eq.) was dissolved in 1,4-dioxane (2.5 mL) and HCl_{aq} (112 mg, 256 µL, 3.08 mmol, 24.0 eq.) was added. The reaction mixture was heated at 60 °C for 19 h, after which water was added (5 mL). The phases were separated, and the aqueous phase was extracted with CH_2Cl_2 (3 × 10 mL). The combined organic extracts were dried over Na_2SO_4, filtered and the solvent was removed under reduced pressure. The obtained crude diol was used without further purification in the next step.

The crude diol (105 mg, 127 µmol, 1.00 eq.) was dissolved in pyridine (3.0 mL) and $POCl_3$ (39.0 mg, 23.8 µL, 255 µmol, 2.00 eq.) was added dropwise at room temperature. The obtained reaction solution was stirred at 60 °C for 15 h. Subsequently, water (3.0 mL) was added at room temperature and the reaction mixture was heated at 100 °C for 3 h. The reaction mixture was cooled back to room temperature and 6 N HCl_{aq} (5.8 mL) was added, followed by heating to 100 °C for 1 h. The reaction mixture was extracted with CH_2Cl_2 (3 × 10 mL), and the combined organic extracts were dried over Na_2SO_4, filtered and the solvent was removed under reduced pressure. Purification by column chromatography (silica, CH_2Cl_2/MeOH = 97/3) yielded a yellow solid which was redissolved in CH_2Cl_2 (20 mL) and washed with 6 N HCl_{aq} (1 × 15 mL). Drying of the organic phase over Na_2SO_4, filtration and removal of all solvents under reduced pressure yielded phosphoric acid **9** (71.3 mg, 80.4 µmol, 63%) as a yellow solid.

TLC: R_f = 0.37 (CH_2Cl_2/MeOH = 95/5) [UV/$KMnO_4$].

M.p.: >230 °C

^{1}H NMR (400 MHz, DMSO-d_6, 300 K): δ [ppm] = 8.63 (s, 2H, C1'-H), 8.44 (d, ^3J = 8.2 Hz, 2H, C8'-H), 8.02 (s, 2H, C2''-H), 7.70 – 7.90 (m, 8H, C_{Ar}-H), 7.45 – 7.67 (m, 8H, C_{Ar}-H), 7.38 (d,

^3J = 7.6 Hz, 2H, C5-H), 7.24 (d, ^3J = 7.6 Hz, 2H, C4-H), 3.10 – 3.29 (m, 2H, C3-H^a), 2.67 – 3.02 (m, 2H, C3-H^b), 2.25 – 2.46 (m, 2H, C2-H^a), 1.89 – 2.20 (m, 2H, C2-H^b).

^{13}C NMR (126 MHz, DMSO-d_6, 300 K): δ [ppm] = 178.7 (s, 2 × C=O), 145.4 (s, 2 × C_{Ar}), 143.1 (d, $^2J_{C\text{-}P}$ = 8.2 Hz, 2 × C7), 140.9 (s, 2 × C_{Ar}), 139.1 (s, 2 × C_{Ar}), 138.7 (s, 2 × C_{Ar}), 138.2 (s, 2 × C_{Ar}), 136.5 (s, 2 × C_{Ar}), 135.5 (s, 2 × C_{Ar}), 133.8 (d, $^3J_{C\text{-}P}$ = 8.2 Hz, 2 × C6), 133.0 (d, 2 × C_{Ar}), 131.5 (d, 2 × C2''), 129.8 (d, 2 × C5), 129.1 (d, 2 × C_{Ar}), 128.9 (d, 2 × C_{Ar}), 128.6 (s, 2 × C_{Ar}), 128.3 (d, 2 × C_{Ar}), 127.9 (d, 2 × C_{Ar}), 127.4 (d, 2 × C_{Ar}), 126.8 (d, 2 × C_{Ar}), 126.6 (d, 2 × C_{Ar}), 126.4 (d, 2 × C1'), 125.1 (d, 2 × C_{Ar}), 122.0 (d, 2 × C4), 59.4 (s, C1), 38.7 (t, 2 × C2), 29.8 (t, 2 × C3).[§]

[§]One of the aromatic carbon atoms was not detected by ^{13}C NMR, presumably due to signal overlap.

^{31}P NMR (203 MHz, DMSO-d_6, 300 K): δ [ppm] = −13.09.
IR (ATR): ṽ (cm^{-1}) = 1636 (m, C=O), 1593 (m, C=C), 1326 (w), 1215 (s), 1078 (w), 742 (s).

HRMS (ESI): calc. for [M + H^+]: 887.1686; found: 887.1691.

Specific Rotation: $[\alpha]_D^{25}$ = 242 (c = 1.0, $CHCl_3$)

7. Racemic Photoinduced *Diels-Alder Reaction*

General Procedure 1 (GP 1): Racemic photoinduced Diels-Alder Reaction

In a Duran phototube carboxylic acid **1** (1.00 eq.) was dissolved in CH_2Cl_2 to obtain a 40 mM solution. The diene was added, and the solution was diluted with the equal amount of CH_2Cl_2 to obtain a 20 mM solution. The solution was irradiated for 3.5 h with fluorescent light tubes (λ_{max} = 366 nm). The solvent was removed under reduced pressure and the crude product was purified by column chromatography.

5-Hydroxy-1,4,4a,5,6,7,8,9-octahydro-5,9a-(epoxymethano)-1,4-methanobenzo[7]annulen-10-one (*rac*-3a)

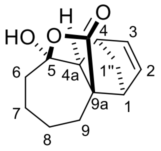

$C_{13}H_{16}O_3$
220.27 g/mol

Following **GP1** carboxylic acid **1** (77.1 mg, 500 µmol, 1.00 eq.) was reacted with cyclopentadiene (1.65 g, 2.07 mL, 25 mmol, 50.0 eq.) in 25 mL CH_2Cl_2. After column chromatography (silica, Hex/EtOAc = 60/40) the product was obtained in two fractions. The first fraction contained a diastereomeric mixture of the product *rac*-**3a**/*rac*-**3a'** (*d.r.* = 76/24, 38.5 mg, 175 µmol, 35%) and was obtained as a white solid. The second fraction contained only the major product isomer *rac*-**3a** (60.3 mg, 274 µmol, 55%) and was obtained as a white solid (combined yield: *d.r.* = 91/9, 98.8 mg, 90%).

*Analytical data of the major diastereomer rac-***3a***:*

TLC: R_f = 0.38 (Hex/EtOAc = 50/50) [$KMnO_4$].

Mp.: 152 °C.

^{1}H NMR (500 MHz, CD_2Cl_2, 300 K): δ [ppm] = 6.09 – 6.27 (m, 2H, C2-H, C3-H), 3.31 (br s, 1H, OH), 3.01 – 3.18 (m, 1H, C4-H), 2.74 – 2.83 (m, 1H, C1-H), 2.70 (d, ^3J = 3.6 Hz, 1H, C4a-H), 2.06 – 2.20 (m, 1H, C9-H^a), 1.93 – 2.03 (m, 1H, C6-H^a), 1.77 – 1.89 (m, 2H, C6-H^b, C8-H^a), 1.62 – 1.77 (m, 4H, C7-H^a, C8-H^b, C9-H^b, C1”-H^a), 1.44 – 1.56 (m, 1H, C1”-H^b), 1.29 – 1.41 (m, 1H, C7-H^b).

^{13}C NMR (101 MHz, CD_2Cl_2, 300 K): δ [ppm] = 179.7 (s, C=O), 138.2 (d, C2), 134.0 (d, C3), 107.2 (s, C5), 60.8 (s, C9a), 52.3 (d, C4a), 51.2 (t, C1”), 50.2 (d, C1), 44.4 (d, C4), 43.2 (t, C6), 32.2 (t, C9), 25.3 (t, C8), 22.5 (t, C7).

IR (ATR): ṽ (cm^{-1}) = 3281 (br, O-H), 2937 (w, C-H), 1716 (s, C=O), 1242 (m), 1223 (m), 909 (m), 714 (s, C=C).

HRMS (ESI): calc. for [M + H^+]: 221.1178; found: 221.1180.

Crystals suitable for X-ray analysis were obtained by preparing a concentrated solution of *rac*-**3a** in EtOAc and allowing the solvent to slowly evaporate at room temperature.

5'-Hydroxy-1',4',4a',5',6',7',8',9'-octahydrospiro[cyclopropane-1,12'-[5,9a](epoxymethano)[1,4]methanobenzo[7]annulen]-10'-one (*rac*-3b/*rac*-3b')

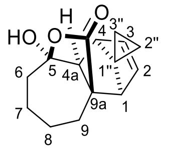

$C_{15}H_{18}O_3$
246.31 g/mol

Following **GP1** carboxylic acid **1** (15.4 mg, 100 µmol, 1.00 eq.) was reacted with spiro[2.4]hepta-4,6-diene (138 mg, 150 µL, 1.5 mmol, 15.0 eq.) in 5 mL CH_2Cl_2. After column chromatography (silica, Hex/EtOAc = 60/40) the product was obtained as a white solid containing two diastereoisomers (*rac*-**3b**/*rac*-**3b'**) (*d.r.* = 94/6, 12.2 mg, 49.5 µmol, 50%).

*NMR data of the major diastereomer rac-**3b**:*

TLC: R_f = 0.47 (Hex/EtOAc = 50/50) [$KMnO_4$].

^{1}H NMR (500 MHz, CD_2Cl_2, 300 K): δ [ppm] = 6.23 – 6.36 (m, 2H, C2-H, C3-H), 3.29 (br s, 1H, OH), 2.94 (d, ^3J = 3.8 Hz, 1H, C4a-H), 2.44 (dd, ^3J = 3.8 Hz, ^3J = 1.9 Hz, 1H, C4-H), 2.19 – 2.30 (m, 1H, C1-H), 2.03 – 2.10 (m, 1H, C9-H^a), 1.96 – 2.02 (m, 1H, C6-H^a), 1.80 – 1.91 (m, 3H, C8-H^a, C6-H^b, C9-H^b), 1.67 – 1.80 (m, 2H, C7-H^a, C8-H^b), 1.32 – 1.46 (m, 1H, C7-H^b), 0.66 (dt, ^2J = 9.6 Hz, ^3J = 5.2 Hz, 1H, C2”-H^a), 0.50 (dt, ^2J = 9.6 Hz, ^3J = 5.2 Hz, 1H, C2”-H^b), 0.45 (dt, ^2J = 10.1 Hz, ^3J = 5.2 Hz, 1H, C3”-H^a), 0.38 (dt, ^2J = 10.1 Hz, ^3J = 5.2 Hz, 1H, C3”-H^b).

^{13}C NMR (101 MHz, CD_2Cl_2, 300 K): δ [ppm] = 176.4 (s, C=O), 135.3 (d, C2), 131.4 (d, C3), 104.5 (s, C5), 59.3 (s, C9a), 51.6 (d, C1), 49.9 (d, C4a), 47.1 (d, C4), 45.1 (s, C1”), 40.1 (t, C6), 28.3 (t, C9), 22.4 (t, C8), 19.8 (t, C7), 6.2 (t, C2”), 0.9 (t, C3”).

IR (ATR): ṽ (cm^{-1}) = 3368 (br, O-H), 2932 (w, C-H), 1731 (s, C=O), 1153 (m), 1213 (m), 908 (m), 734 (m, C=C).

HRMS (ESI): calc. for [M + H^+]: 247.1334; found: 247.1344.

5-Hydroxy-1-propyl-1,4,4a,5,6,7,8,9-octahydro-5,9a-(epoxymethano)benzo[7]annulen-10-one (*rac*-3c/*rac*-3c')

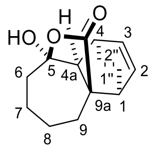

$C_{14}H_{18}O_3$
250.34 g/mol

Following **GP1** carboxylic acid **1** (30.8 mg, 200 µmol, 1.00 eq.) was reacted with cyclohexadiene (801 mg, 953 µL, 10.0 mmol, 50.0 eq.) in 10 mL CH_2Cl_2. After column chromatography (silica, Hex/EtOAc = 70/30) the product was obtained as a white solid containing two diastereoisomers (*rac*-**3c**/*rac*-**3c'**) (*d.r.* = 88/12, 26.9 mg, 115 µmol, 57%).

*NMR data of the major diastereomer rac-**3c**:*

TLC: R_f = 0.16 (Hex/EtOAc = 70/30) [$KMnO_4$].

^{1}H NMR (500 MHz, CD_2Cl_2, 300 K): δ [ppm] = 6.19 – 6.26 (m, 2H, C2-H, C3-H), 3.57 (br s, 1H, OH), 2.82 – 2.90 (m, 1H, C4-H), 2.54 – 2.68 (m, 1H, C1-H), 2.12 – 2.31 (m, 1H, C4a-H), 1.93 – 2.04 (m, 1H, C6-H^a), 1.60 – 1.88 (m, 7H, C6-H^b, C7-H^a, C8-H^a, C9-H, C2''-H), 1.46 – 1.55 (m, 1H, C1''-H^a), 1.35 – 1.45 (m, 1H, C8-H^b), 1.20 – 1.28 (m, 2H, C7-H^b, C1''-H^b).

^{13}C NMR (101 MHz, CD_2Cl_2, 300 K): δ [ppm] = 180.8 (s, C=O), 135.4 (d, C2), 133.0 (d, C3), 108.9 (s, C5), 56.8 (s, C9a), 50.3 (d, C4a), 42.3 (t, C6), 37.0 (d, C1), 31.6 (t, C9), 30.0 (d, C4), 27.3 (t, C1''), 24.9 (t, C2''), 23.0 (t, C8), 18.5 (t, C7).

IR (ATR): ṽ (cm^{-1}) = 3370 (br, O-H), 2937 (w, C-H), 1724 (s, C=O), 1227 (m), 1053 (m), 912 (m), 743 (w, C=C).

HRMS (ESI): calc. for [M + H^+]: 235.1334; found: 235.1331.

5-Hydroxy-1,4,4a,5,6,7,8,9-octahydro-1,4-epoxy-5,9*a*-(epoxymethano)benzo[7]annulen-10-one (*rac*-3d/*rac*-3d')

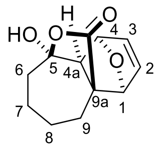

$C_{12}H_{14}O_4$
222.24 g/mol

Following **GP1** carboxylic acid **1** (30.8 mg, 200 µmol, 1.00 eq.) was reacted with furan (681 mg, 727 µL, 10.0 mmol, 50.0 eq.) in 10 mL CH_2Cl_2. After column chromatography (silica, CH_2Cl_2/MeOH = 95/5) the product was isolated as a colorless solid containing two diastereoisomers (*rac*-**3d**/*rac*-**3d'**) (*d.r.* = 71/29, 44.2 mg, 190 µmol, 95%).

*NMR data of the major diastereomer rac-**3d**:*

TLC: R_f = 0.27 (CH_2Cl_2/MeOH= 95/5) [$KMnO_4$].

^{1}H NMR (500 MHz, CD_2Cl_2, 300 K): δ [ppm] = 6.46 – 6.52 (m, 2H, C2-H, C3-H), 5.01 – 5.06 (m, 1H, C1-H), 4.65 – 4.74 (m, 1H, C4-H), 4.24 (br s, OH), 2.68 – 2.98 (m, 1H, C4a-H), 2.18 – 2.26 (m, 1H, C9-H^a), 1.95 – 2.13 (m, 2H, C6-H), 1.67 – 1.89 (m, 4H, C7-H^a, C8-H, C9-H^b), 1.29 – 1.42 (m, 1H, C7-H^b).

^{13}C NMR (126 MHz, CD_2Cl_2, 300 K): δ [ppm] = 178.6 (s, C=O), 137.8 (d, C2), 134.5 (d, C3), 106.3 (s, C5), 84.2 (d, C4), 80.7 (d, C1), 62.0 (s, C9a), 51.5 (d, C4a), 43.6 (t, C6), 31.0 (t, C9), 25.2 (t, C8), 22.9 (t, C7).

IR (ATR): ṽ (cm^{-1}) = 3366 (br, O-H), 2935 (m, C-H), 1736 (s, C=O), 1242 (m), 897 (m), 716 (w).

HRMS (ESI): calc. for [M + H^+]: 223.0970; found: 223.0964.

5-Hydroxy-1,4-dimethyl-1,4,4a,5,6,7,8,9-octahydro-1,4-epoxy-5,9a-(epoxymethano)benzo[7]annulen-10-one (*rac*-3e)

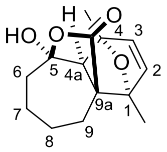

$C_{14}H_{18}O_4$
250.29 g/mol

Following **GP1** carboxylic acid **1** (154 mg, 1.00 mmol, 1.00 eq.) was reacted with 2,5-dimethylfurane (4.81 g, 5.32 mL, 50.0 mmol, 50.0 eq.) in 50 mL CH_2Cl_2. After column

chromatography (silica, Hex/EtOAc = 70/30 + 0.1% AcOH) the product *rac*-**3e** was isolated as a colorless solid (233 mg, 931 µmol, 93%).

TLC: R_f = 0.45 (Hex/EtOAc = 50/50 + 0.1% AcOH) [$KMnO_4$].

Mp.: 205 °C.

^{1}H NMR (400 MHz, CD_2Cl_2, 300 K): δ [ppm] = 6.30 (d, ^3J = 5.6 Hz, 1H, C3-H), 6.25 (d, ^3J = 5.6 Hz, 1H, C2-H), 3.55 (br s, 1H, OH), 2.56 (s, 1H, C4a-H), 1.66 – 2.05 (m, 7H, C6-H, C7-H^a, C8-H. C9-H), 1.60 (s, 3H, C4-CH_3), 1.51 (s, 3H, C1-CH_3), 1.33 – 1.46 (m, 1H, C7-H^b).

^{13}C NMR (126 MHz, CD_2Cl_2, 300 K): δ [ppm] = 178.5 (s, C=O), 141.5 (d, C2), 138.4 (d, C3), 105.9 (s, C5), 88.5 (s, C1), 87.6 (s, C4), 65.2 (s, C9a), 58.1 (d, C4a-H), 43.9 (t, C6), 28.4 (t, C6), 25.1 (t, C8), 22.6 (t, C7), 19.6 (q, C4-*C*H_3), 14.7 (q, C1-*C*H_3).

IR (ATR): ṽ (cm^{-1}) = 3368 (br, O-H), 2934 (m, C-H), 1727 (s, C=O), 1194 (m), 902 (s), 864 (s).

HRMS (ESI): calc. for [M + H^+]: 251.1283; found: 251.1288.

Crystals suitable for X-ray analysis were obtained by preparing a concentrated solution of *rac*-**3e** in EtOAc and allowing the solvent to slowly evaporate at room temperature.

1,4-Diethyl-5-hydroxy-1,4,4a,5,6,7,8,9-octahydro-1,4-epoxy-5,9a-(epoxymethano)benzo[7]annulen-10-one (*rac*-3f)

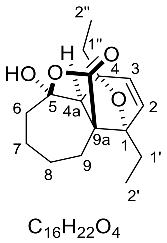

$C_{16}H_{22}O_4$
278.35 g/mol

Following **GP1** carboxylic acid **1** (30.8 mg, 200 µmol, 1.00 eq.) was reacted with 2,5-diethylfurane (621 mg, 701 µL, 5.00 mmol, 25.0 eq.) in 10 mL CH_2Cl_2. After column chromatography (silica, Hex/EtOAc = 70/30 + 0.1% AcOH) the product *rac*-**3f** was isolated as a colorless solid (52.8 mg, 190 µmol, 95%).

TLC: R_f = 0.32 (Hex/EtOAc = 70/30 + 0.1% AcOH) [$KMnO_4$].

Mp.: 118 °C.

^{1}H NMR (400 MHz, CD_2Cl_2, 300 K): δ [ppm] = 6.25 – 6.36 (m, 2H, C2-H, C3-H), 3.60 (br s, 1H, OH), 2.60 (1H, C4a-H), 2.13 – 2.25 (m, 1H, C1"-H^a), 1.62 – 2.04 (m, 10H, C6-H, C7-H^a, C8-H, C9-H, C1'-H, C1"-H^b), 1.33 – 1.46 (m, 1H, C7-H^b), 1.03 (t, ^3J = 7.5 Hz, 3H, C2'-H), 0.98 (t, ^3J = 7.5 Hz, 3H, C2"-H).

^{13}C NMR (101 MHz, CD_2Cl_2, 300 K): δ [ppm] = 178.4 (s, C=O), 139.7 (d, C2), 137.3 (s, C3), 92.5 (s, C4), 91.3 (s, C1), 65.4 (s, C9a), 57.0 (d, C4a), 43.8 (t, C6), 28.4 (t, C9), 26.3 (t, C1"), 25.0 (t, C8), 22.5 (t, C1'), 21.7 (t, C7), 9.5 (q, C2'), 9.0 (q, C2").[§]

[§]Carbon atom C5 was not detectable by ^{13}C NMR.

IR (ATR): ṽ (cm^{-1}) = 3334 (br, O-H), 2939 (w, C-H), 1756 (s, C=O), 1233 (m), 1182 (s), 926 (s), 907 (s).

HRMS (ESI): calc. for [M + H^+]: 279.1596; found: 279.1591.

5-Hydroxy-1,4-dipropyl-1,4,4a,5,6,7,8,9-octahydro-1,4-epoxy-5,9a-(epoxymethano)benzo[7]annulen-10-one (*rac*-3g)

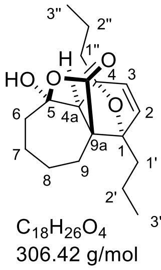

Following **GP1** carboxylic acid **1** (30.8 mg, 200 μmol, 1.00 eq.) was reacted with 2,5-dipropylfuran (761 mg, 864 μL, 5.00 mmol, 25.0 eq.) in 10 mL CH_2Cl_2. After column chromatography (silica, Hex/EtOAc = 80/20 + 0.1% AcOH) the product *rac*-**3g** was isolated as a colorless oil (58.8 mg, 192 μmol, 96%).

TLC: R_f = 0.19 (Hex/EtOAc = 80/20 + 0.1% AcOH) [$KMnO_4$].

^{1}H NMR (500 MHz, CD_2Cl_2, 300 K): δ [ppm] = 6.31 (d, ^3J = 5.7 Hz, 1H, C3-H), 6.29 (d, ^3J = 5.7 Hz, 1H, C2-H), 3.49 (br s, 1H, OH), 2.57 (s, 1H, C4a-H), 2.09 – 2.24 (m, 1H, C1"-H^a), 1.92 – 2.02 (m, 2H, C6-H^a, C9-H^a), 1.77 – 1.92 (m, 4H C6-H^b, C8-H^a, C9-H^b, C1'-H^a), 1.64 – 1.77 (m, 4H, C7-H^a, C8-H^b, C1'-H^b, C1"-H^b), 1.36 – 1.57 (m, 5H, C7-H^b, C2'-H, C2"-H), 0.91 – 0.98 (m, 6H, C3'-H, C3"-H).

^{13}**C NMR** (126 MHz, CD_2Cl_2, 300 K): δ [ppm] = 178.5 (s, C=O), 139.9 (d, C2), 137.4 (d, C3), 92.2 (s, C4), 91.0 (s, C1), 65.5 (s, C9a), 57.3 (d, C4a), 44.0 (t, C6), 35.8 (t, C1''), 31.0 (t, C1'), 28.5 (t, C9), 25.2 (t, C8), 22.7 (t, C7), 19.1 (t, C2'), 18.6 (C2''), 14.9 (q, C3'), 14.9 (C3'').[§]

[§]Carbon atom C5 was not detectable by ^{13}C NMR.

IR (ATR): ṽ (cm^{-1}) = 3365 (br, O-H), 2933 (m, C-H), 1732 (s, C=O), 1235 (s), 1189 (s), 950 (s), 911 (s).

HRMS (ESI): calc. for [M + H^+]: 307.1909; found: 307.1909.

1,4-Dibutyl-5-hydroxy-1,4,4a,5,6,7,8,9-octahydro-1,4-epoxy-5,9a-(epoxymethano)benzo[7]annulen-10-one (*rac*-3h)

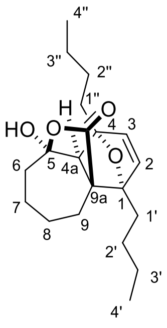

$C_{20}H_{30}O_4$
334.46 g/mol

Following **GP1** carboxylic acid **1** (30.8 mg, 200 µmol, 1.00 eq.) was reacted with 2,5-dibutylfuran (901 mg, 1.04 mL, 5.00 mmol, 25.0 eq.) in 10 mL CH_2Cl_2. After column chromatography (silica, Hex/EtOAc = 90/10 → 80/20 + 0.1% AcOH) the product *rac*-**3h** was isolated as a colorless oil (64.2 mg, 176 µmol, 88%).

TLC: R_f = 0.45 (Hex/EtOAc = 60/40) [$KMnO_4$].

^{1}**H NMR** (400 MHz, CD_2Cl_2, 300 K): δ [ppm] = 6.31 (d, ^3J = 5.7 Hz, 1H, C3-H), 6.27 (^3J = 5.7 Hz, 1H, C2-H), 4.27 (br s, 1H, OH), 2.47 – 2.65 (m, 1H, C4a-H), 2.11 – 2.24 (m, 1H, C1''-H^a), 1.67 – 2.05 (m, 11H, C1''-H^b, C6-H, C7-H, C8-H, C9-H, C1'-H), 1.27 – 1.50 (m, 8H, C2'-H, C3'-H, C2''-H, C3''-H), 0.85 – 0.96 (m, 6H, C4'-H, C4''-H).

^{13}**C NMR** (101 MHz, CD_2Cl_2, 300 K): δ [ppm] = 179.0 (s, C=O), 139.7 (d, C2), 137.5 (d, C3), 92.2 (s, C4), 91.1 (s, C1), 65.5 (s, C9a), 57.7 (d, C4a), 43.8 (t, C6), 33.4 (t, C1''), 28.7 (t, C9), 28.6 (t, C1'), 27.9 (t, C2''), 27.4 (t, C2'), 25.3 (t, C8), 23.7 (t, C3''), 23.7 (t, C3'), 22.8 (t, C7), 14.4 (q, C4'), 14.4 (q, C4'').

[§]Carbon atom C5 was not detectable by ^{13}C NMR.

IR (ATR): ṽ (cm^{-1}) = 3351 (br, O-H), 2933 (m, C-H), 1732 (s, C=O), 1237 (s), 1189 (s).

HRMS (ESI): calc. for [M + H^+]: 335.2222; found: 335.2223.

5-Hydroxy-1,2-dimethyl-1,4,4a,5,6,7,8,9-octahydro-1,4-epoxy-5,9a-(epoxymethano)benzo[7]annulen-10-one (*rac*-3i/*rac*-3i'/*rac*-3i")

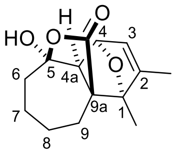

$C_{14}H_{18}O_4$
250.29 g/mol

Following **GP1** carboxylic acid **1** (30.8 mg, 200 µmol, 1.00 eq.) was reacted with 2,3-dimethylfuran (961 mg, 1.06 mL, 10.0 mmol, 50.0 eq.) in 10 mL CH_2Cl_2. After column chromatography (silica, Hex/EtOAc = 50/50 + 0.1% AcOH) the product was obtained as a white solid containing a mixture of two regioisomers (*rac*-**3i**/*rac*-**3i'**), of which the minor isomer *rac*-**3i'** was present as two diastereoisomers (*rac*-**3i'**/*rac*-**3i"**) (*r.r.* = 84/16, *d.r.* [minor regioisomer] = 73/27, 49.1 mg, 196 µmol, 98%).

*NMR data of the major regioisomer rac-***3i**:

TLC: R_f = 0.34 (Hex/EtOAc 50/50) [$KMnO_4$].

^{1}H NMR (400 MHz, CD_2Cl_2, 300 K): δ [ppm] = 6.07 – 6.12 (m, 1H, C3-H), 4.78 (dd, ^3J = 4.6 Hz, ^3J = 1.9 Hz, 1H, C4-H), 4.57 (br s, 1H, OH), 2.98 (d, ^3J = 4.6 Hz, 1H, C4a-H), 1.95 – 2.06 (m, 2H, C6-H^a, C9-H^a), 1.66 – 1.93 (m, 8H, C6-H^b, C7-H^a, C8-H, C9-H^b, C2-CH_3), 1.37 – 1.49 (m, 4H, C7-H^b, C1-CH_3).

^{13}C NMR (101 MHz, CD_2Cl_2, 300 K): δ [ppm] = 177.6 (s, C=O), 150.2 (s, C2), 129.5 (d, C3), 105.7 (s, C5), 91.1 (s, C1), 78.4 (d, C4-H), 62.1 (s, C9a), 54.4 (d, C4a), 43.3 (t, C6), 28.6 (t, C9), 25.0 (t, C8), 22.5 (t, C7), 13.7 (q, C2-CH_3), 13.1 (q, C1-CH_3).

IR (ATR): ṽ (cm^{-1}) = 3346 (br, O-H), 2937 (m, C-H), 1734 (s, C=O), 1240 (s), 1078 (m), 937 (s), 621 (m).

HRMS (ESI): calc. for [M + H^+]: 251.1283; found: 251.1288.

3-Bromo-5-hydroxy-1,4,4a,5,6,7,8,9-octahydro-1,4-epoxy-5,9a-(epoxymethano)benzo[7]annulen-10-one (*rac*-3j') and 2-Bromo-5-hydroxy-1,4,4a,5,6,7,8,9-octahydro-1,4-epoxy-5,9a-(epoxymethano)benzo[7]annulen-10-one (*rac*-3j)

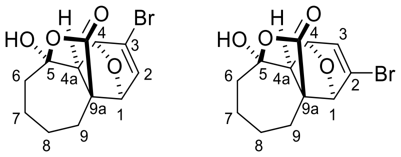

$C_{12}H_{13}BrO_4$
301.14 g/mol

Following **GP1** carboxylic acid **1** (30.8 mg, 200 µmol, 1.00 eq.) was reacted with 3-bromofuran (1.47 g, 896 µL, 10.0 mmol, 50.0 eq.) in 10 mL CH_2Cl_2. After column chromatography (silica, Hex/EtOAc = 80/20 + 0.1% AcOH) two regioisomeric products were isolated. The first eluting minor isomer *rac*-**3j'** (19.2 mg, 63.8 µmol, 32%) was isolated as an off-white solid. The major isomer *rac*-**3j** (32.9 mg, 109 µmol, 55%) was also isolated as an off-white solid (combined yield: *r.r.* = 63/37, 52.1 mg, 87%).

*Analytical data of the minor regioisomer rac-**3j'**:*

TLC: R_f = 0.53 (Hex/EtOAc = 70/30) [$KMnO_4$].

Mp.: 187 °C.

^{1}H NMR (400 MHz, CD_2Cl_2, 300 K): δ [ppm] = 6.53 (d, ^3J = 1.9 Hz, 1H, C2-H), 5.14 (s, 1H, C4-H), 4.94 (d, ^3J = 1.9 Hz, 1H, C1-H), 4.14 (br s, 1H, OH), 2.15 (s, 1H, C4a-H), 2.00 – 2.14 (m, 2H, C6-H^a, C9-H^a), 1.73 – 1.91 (m, 3H, C6-H^b, C7-H^a, C8-H^a), 1.31 – 1.54 (m, 2H, C7-H^b, C8-H^b), 1.14 – 1.28 (m, 1H, C9-H^b).

^{13}C NMR (101 MHz, CD_2Cl_2, 300 K): δ [ppm] = 177.6 (s, C=O), 132.6 (d, C2), 130.5 (s, C3), 106.8 (s, C5), 85.3 (d, C1), 83.4 (d, C4), 62.1 (s, C9a), 51.4 (d, C4a), 42.4 (t, C6), 28.4 (t, C9), 25.1 (t, C8), 22.1 (t, C7).

IR (ATR): ṽ (cm^{-1}) = 3325 (br, O-H), 2928 (m, C-H), 1727 (s, C=O), 1319 (m), 1173 (s), 938 (s).

HRMS (ESI): calc. for [M + H^+]: 301.0075; found: 301.0081.

*Analytical data of the major regioisomer rac-**3j**:*

TLC: R_f = 0.23 (Hex/EtOAc = 70/30) [$KMnO_4$].

Mp.: 168 °C.

^{1}H NMR (400 MHz, CD_2Cl_2, 300 K): δ [ppm] = 6.17 – 6.91 (m, 1H, C3-H), 4.91 – 5.09 (m, 1H, C4-H), 4.59 (s, 1H, C1-H), 3.96 (br s, 1H, OH), 2.73 – 3.22 (m, 1H, C4a-H), 2.23 – 2.41 (m, 1H, C9-H^a), 2.04 – 2.13 (m, 1H, C6-H^a), 1.68 – 2.01 (m, 5H, C6-H^b, C8-H, C9-H^b, C7-H^a), 1.37 – 1.54 (m, 1H, C7-H^b).

^{13}C NMR (101 MHz, CD_2Cl_2, 300 K): δ [ppm] = 176.0 (s, C=O), 135.1 (d, C3), 127.0 (s, C2), 100.5 (s, C5), 88.3 (d, C1), 82.6 (d, C4), 62.4 (s, C9a), 43.8 (t, C6), 31.3 (t, C9), 25.3 (t, C8), 22.7 (t, C7).[§]

[§]Carbon atom C4a was not detectable by ^{13}C NMR.

IR (ATR): ṽ (cm^{-1}) = 3304 (br, O-H), 2936 (m, C-H), 1719 (s, C=O), 1239 (m), 898 (s), 872 (s).

HRMS (ESI): calc. for [M + H^+]: 301.0075; found: 301.0081.

5-Hydroxy-10-oxo-4a,5,6,7,8,9-hexahydro-1,4-epoxy-5,9a-(epoxymethano)benzo[7]annulen-1(4H)-yl)methyl acetate (*rac*-3k)

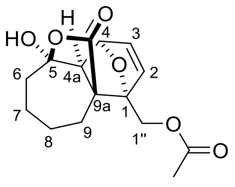

$C_{15}H_{18}O_6$
294.30 g/mol

Following **GP1** carboxylic acid **1** (30.8 mg, 200 µmol, 1.00 eq.) was reacted with furan-2-ylmethyl acetate (1.40 g, 1.25 mL, 10.0 mmol, 50.0 eq.) in 10 mL CH_2Cl_2. After column chromatography (silica, Hex/EtOAc = 70/30) product *rac*-**3k** was isolated as a colorless oil (14.3 mg, 48.6 µmol, 24%).

TLC: R_f = 0.21 (Hex/EtOAc = 70/30) [$KMnO_4$].

^{1}H NMR (400 MHz, CD_2Cl_2, 300 K): δ [ppm] = 6.66 (ddd, ^3J = 5.8 Hz, ^3J = 1.9 Hz, ^4J = 0.9 Hz, 1H, C3-H), 6.30 (d, ^3J = 5.8 Hz, 1H, C2-H, 5.28 (d, 1H, ^3J = 1.9 Hz, C4-H), 4.80 (d, ^2J = 12.9 Hz, 1H, C1''-H^a), 4.56 (d, ^2J = 12.9 Hz, 1H, C1''-H^b), 4.19 (br s, 1H, OH), 2.17 (d, ^4J = 0.9 Hz, 1H, C4a-H), 2.01 – 2.14 (m, 5H, C6-H^a, C9-H^a, CH_3), 1.83 – 1.91 (m, 1H, C8-H^a, 1.71 – 1.81 (m, 2H, C6-H^b, C7-H^a), 1.33 – 1.45 (m, 2H, C7-H^b, C8-H^b), 1.19 – 1.30 (m, 1H, C9-H^b).

^{13}C NMR 101 MHz, CD_2Cl_2, 300 K): δ [ppm] = 177.2 (s,O=*C*-C9a), 171.2 (s, O=*C*CH_3), 141.4 (d, C3), 133.9 (d, C2), 106.5 (s, C5), 91.8 (s, C1), 78.6 (d, C4), 61.7 (t, C1''), 61.5 (s, C9a), 55.1 (d, C4a), 43.0 (t, C6), 29.6 (t, C9), 25.8 (t, C8), 22.5 (t, C7), 21.1 (q, CH_3).

IR (ATR): ṽ (cm^{-1}) = 3404 (br, O-H), 2937 (m, C-H), 1736 (s, C=O), 1234 (s), 948 (m), 911 (s).

HRMS (ESI): calc. for [M + H^+]: 295.1182; found: 295.1181.

5-Hydroxy-1-methyl-1,4,4*a*,5,6,7,8,9-octahydro-1,4-epoxy-5,9*a*-(epoxymethano)benzo[7]*a*nnulen-10-one (*rac*-3l/*rac*-3l')

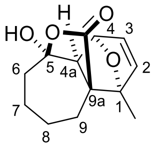

$C_{13}H_{16}O_4$
236.27 g/mol

Following **GP1** carboxylic acid **1** (46.3 mg, 300 µmol, 1.00 eq.) was reacted with 2-methylfuran (1.23 g, 1.33 mL, 15.0 mmol, 50.0 eq.) in 15 mL CH_2Cl_2. After column chromatography (silica, Hex/EtOAc = 50/50 + 0.1% AcOH) the product was obtained as a white solid containing a mixture of two regioisomers (*rac*-**3l**/*rac*-**3l'**) (*r.r.* = 64/36, 46.0 mg, 195 µmol, 65%).

NMR data of the major regioisomer:

TLC: R_f = 0.21 (Hex/EtOAc = 50/50 + 0.1% AcOH) [$KMnO_4$].

^{1}H NMR (500 MHz, CD_2Cl_2, 300 K): δ [ppm] = 6.47 (dd, ^3J = 5.7 Hz, ^3J = 1.8 Hz, 1H, C3-H), 6.28 (d, ^3J = 5.7 Hz, 1H, C2-H), 4.91 (dd, ^3J = 4.7 Hz, ^3J = 1.8 Hz, 1H, C4-H), 4.18 (br s, 1H, OH), 2.94 (br s, 1H, C4a-H), 1.96 – 2.10 (m, 2H, C6-H^a, C9-H^a), 1.81 – 1.90 (m, 1H, C6-H^b), 1.68 – 1.81 (m, 4H, C7-H^a, C8-H, C9-H^b), 1.55 (s, 3H, CH_3), 1.33 – 1.44 (m, 1H, C7-H^b).

^{13}C NMR (101 MHz, CD_2Cl_2, 300 K): δ [ppm] = 178.8 (s, C=O), 141.2 (d, C2), 135.3 (d, C3), 106.0 (s, C5), 89.8 (s, C1), 79.6 (d, C4), 62.2 (s, C9a), 43.4 (t, C6), 28.2 (t, C9), 25.0 (t, C8), 22.5 (t, C7), 14.7 (q, CH_3).§

§Carbon atom C4a was not detectable by ^{13}C NMR, the respective signal overlaps with the residual solvent signal of CD_2Cl_2.

IR (ATR): ṽ (cm^{-1}) = 3367 (br, O-H), 2930 (m, C-H), 1733 (s, C=O), 1241 (s), 905 (m), 720 (m).

HRMS (ESI): calc. for [M + H^+]: 237.1126; found: 237.1127.

8. Benzylation of Primary Photoproducts and Catalytic Photoinduced *Diels-Alder* Reaction

*General Procedure 2 (GP 2): Benzylation of primary photoproducts **3**[5]*

Photoproduct **3** (1.00 eq.) was dissolved in DMF (25 mM), K_2CO_3 (1.50 eq.) and BnBr (2.00 eq.) were added, and the obtained suspension was stirred for 1 h (unless otherwise stated). Water (15 mL) was added, the phases were separated, and the aqueous phase was extracted with Et_2O (3 × 10 mL). The combined organic extracts were washed with $NaCl_{aq}$ (1 × 20 mL), dried over Na_2SO_4, filtered and the solvent was removed under reduced pressure. The obtained crude product was purified by column chromatography.

General Procedure 3 (GP 3): Catalytic photoinduced Diels-Alder Reaction

A solution of carboxylic acid **1** (1.00 eq.), catalyst (10 mol%) and the respective diene (50.0 eq.) in dichloromethane (c = 10 mM) was cooled to −20 °C and irradiated (λ = 459 nm) for the indicated time. The solvent and excess diene were removed under reduced pressure and the obtained crude product was benzylated according to **GP 2**.

Benzyl (1*R*,4*S*,4a*R*,9a*S*)-9-oxo-1,4,5,6,7,8,9,9a-octahydro-4a*H*-1,4-methanobenzo[7]annulene-4a-carboxylate (4a/4a')

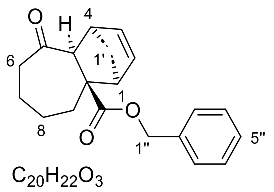

$C_{20}H_{22}O_3$
310.39 g/mol

Benzylation of the primary photoproduct:

According to **GP2** a diastereomeric mixture of the photoproducts *rac*-**3a**/*rac*-**3a'** (*d.r.* = 76/24, 16.6 mg, 75.4 µmol, 1.00 eq.) was reacted with K_2CO_3 (15.6 mg, 113 µmol, 1.50 eq.) and BnBr (25.8 mg, 17.9 µL, 151 µmol, 2.00 eq.) in DMF (3.0 mL). After column chromatography (silica, Hex/EtOAc = 80/20) the product *rac*-**4a**/*rac*-**4a'** was obtained as a colorless oil (*d.r.* = 76/24, 21.2 mg, 68.3 µmol, 91%).

Catalytic photoinduced Diels-Alder Reaction:

According to **GP 3** carboxylic acid **1** (15.4 mg, 100 µmol, 1.00 eq.) was reacted with catalyst **8a** (9.29 mg, 10.0 µmol, 10 mol%) and cyclopentadiene (331 mg, 412 µL, 5.00 mmol, 50.0 eq.) in 10 mL CH_2Cl_2. After benzylation and column chromatography (silica, P/Et_2O = 90/10 → 70/30) a

diastereomeric mixture of the benzylated products **4a**/**4a’** [*d.r.* = 62/38, 14.0 mg, 45.2 µmol, 45%, 38% *ee* (major), 11% *ee* (minor)] was obtained as a colorless oil.

*NMR data of the major diastereoisomer **4a**:*

TLC: R_f = 0.19 (Hex/EtOAc = 80/20) [$KMnO_4$].

^{1}H NMR (400 MHz, CD_2Cl_2, 300 K): δ [ppm] = 7.24 – 7.44 (m, 5H, C3”-H, C4”-H, C5”-H), 6.25 (dd, ^3J = 5.6 Hz, ^3J = 3.0 Hz, 1H, C2-H), 5.84 (dd, ^3J = 5.6 Hz, ^3J = 2.9 Hz, 1H, C3-H), 5.03 (d, ^2J = 12.3 Hz, 1H, C1”-H^a), 4.99 (d, ^2J = 12.3 Hz, C1”-H^b, 1H, C1”-H^b), 3.04 – 3.05 (m, 1H, C4a-H), 2.97 – 3.02 (m, 1H, C1-H), 2.83 – 2.89 (m, 1H, C4-H), 2.37 – 2.42 (m, 1H, C9-H^a), 2.32 – 2.36 (m, 2H, C6), 2.00 – 2.09 (m, 1H, C8-H^a), 1.72 – 1.86 (m, 3H, C9-H^b, C7-H), 1.50 -1.56 (m, 1H, C1’-H^a), 1.44 – 1.49 (m, 1H, C1’-H^b), 1.25 – 1.34 (m, 1H, C8-H^b).

^{13}C NMR (126 MHz, CD_2Cl_2, 300 K): δ [ppm] = 211.1 (s, C=O), 174.4 (s, COO), 136.8 (d, C2), 136.6 (s, C2”), 136.0 (d, C3), 129.1 (d, C_{Ar}), 129.0 (d, C_{Ar}), 129.0 (d, C_{Ar}), 66.8 (t, C1”), 62.4 (d, C4a), 59.9 (s, C9a), 52.0 (d, C4), 47.7 (d, C1’), 44.8 (d, C1), 43.7 (t, C6), 38.8 (t, C9), 28.3 (t, C8), 25.4 (t, C7).

IR (ATR): ṽ (cm^{-1}) = 2930 (w, C-H), 1735 (s, C=O), 1707 (s, C=O), 1139 (s), 721 (w), 699 (w).

HRMS (ESI): calc. for [M + H^+]: 311.1642; found: 311.1642.

Chiral HPLC: t_{R1} = 8.6 min, t_{R2} = 9.1 min, t_{R3} = 9.6 min, t_{R4} = 12.2 min, (*Daicel* Chiralpak AD-H, 250×4.6 mm, n-Hep/iso-PrOH = 90/10, 1 mL/min, λ = 210 nm).

Benzyl (1*R*,4*S*,4a*S*,9a*R*)-9-oxo-1,4,5,6,7,8,9,9a-octahydro-4a*H*-1,4-epoxybenzo[7]annulene-4a-carboxylate (4d) and Benzyl (1*S*,4*R*,4a*S*,9a*R*)-9-oxo-1,4,5,6,7,8,9,9a-octahydro-4a*H*-1,4-epoxy benzo[7]annulene-4a-carboxylate (4d')

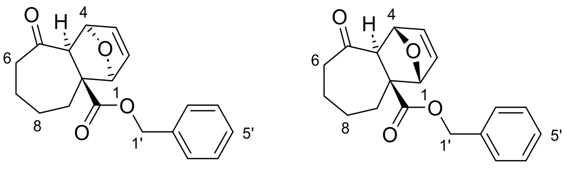

$C_{19}H_{20}O_4$
310.39 g/mol

Benzylation of the primary photoproduct:

According to **GP2** a diastereomeric mixture of the photoproducts *rac*-**3d**/*rac*-**3d'** (*d.r.* = 71/29, 33.3 mg, 150 µmol, 1.00 eq.) was reacted with K_2CO_3 (31.1 mg, 225 µmol, 1.50 eq.) and BnBr (51.3 mg, 35.6 µL, 300 µmol, 2.00 eq.) in DMF (6.0 mL). After column chromatography (silica, P/Et_2O = 80/20 → 50/50) the two diastereomeric products were isolated as colorless oils. The minor diastereomer *rac*-**3d'** eluted first (4.0 mg, 12.8 µmol, 9%) followed by the major diastereomer *rac*-**3d** (24.0 mg, 76.8 µmol, 51%).

Catalytic photoinduced Diels-Alder Reaction:

According to **GP 3** carboxylic acid **1** (15.4 mg, 100 µmol, 1.00 eq.) was reacted with catalyst **8a** (9.29 mg, 10.0 µmol, 10 mol%) and furane (340 mg, 364 µL, 5.00 mmol, 50.0 eq.) in 10 mL CH_2Cl_2. After benzylation and column chromatography (silica, P/Et_2O = 80/20 → 50/50) the two diastereomeric products were isolated as colorless oils. The minor diastereomer **3d'** eluted first (1.3 mg, 4.17 µmol, 4%, 27% *ee*), followed by the major diastereomer **3d** (14.5 mg, 46.5 µmol, 47%, 19% *ee*).

Analytical data of the minor diastereomer:

TLC: R_f = 0.37 (P/Et_2O = 50/50) [$KMnO_4$].

^{1}H NMR (500 MHz, CD_2Cl_2, 300 K): δ [ppm] = 7.13 – 7.43 (m, 5H, C3'-H, C4'-H, C5'-H), 6.45 (dd, ^3J = 5.8 Hz, ^3J = 1.7 Hz, 1H, C3-H), 6.19 (dd, ^3J = 5.8 Hz, ^3J = 1.6 Hz, 1H, C2-H), 5.26 – 5.30 (m, 1H, C4-H), 5.12 – 5.15 (m, 2H, C1'-H), 4.28 – 4.70 (m, 1H, C1-H), 3.16 (s, 1H, C4a-H), 2.59 (*virt.* dt, ^2J = 11.9 Hz, $^3J \approx {}^3J$ = 3.5 Hz, 1H, C6-H^a), 2.46 (ddd, ^2J = 14.6 Hz, ^3J = 6.0 Hz, ^3J = 2.4 Hz, 1H, C9-H^a), 2.35 – 2.43 (m, 1H, C6-H^b), 1.81 – 1.91 (m, 1H, C7-H^a), 1.67 – 1.79 (m, 1H, C8-H^a), 1.47 – 1.51 (m, 1H, C9-H^b), 1.36 – 1.46 (m, 1H, C7-H^b), 1.23 – 1.33 (m, 1H, C8-H^b).

^{13}**C NMR** (126 MHz, CD_2Cl_2, 300 K): δ [ppm] = 210.0 (s, C=O), 173.0 (s, COO), 137.6 (d, C3), 135.8 (s, C2'), 135.2 (d, C2), 128.6 (d, C4'), 128.4 (d, C5'), 128.4 (d, C3'), 86.0 (d, C1), 79.4 (d, C4), 67.0 (t, C1'), 57.6 (d, C4a), 55.8 (s, C8a), 43.3 (t, C6), 35.8 (t, C9), 25.1 (t, C8), 24.9 (t, C7).

IR (ATR): ṽ (cm^{-1}) = 2934 (m, C-H), 1713 (s, C=O), 1455 (w), 1203 (w), 1166 (s), 905 (w), 700 (w).

HRMS (ESI): calc. for [M + H^+]: 313.1435; found: 313.1436.

Chiral HPLC: t_{R1} = 11.5 min t_{R2} = 16.5 min (*Daicel* Chiralpak AD-H, 250×4.6 mm, n-Hep/iso-PrOH = 90/10, 1 mL/min, λ = 210 nm).

Analytical data of the major diastereomer:

TLC: R_f = 0.23 (P/Et_2O = 50/50) [$KMnO_4$].

^{1}**H NMR** (400 MHz, CD_2Cl_2, 300 K): δ [ppm] = 7.29 – 7.42 (m, 5H, C3'-H, C4'-H, C5'-H), 6.50 (dd, ^3J = 5.8 Hz, ^3J = 1.8 Hz, 1H, C3-H), 6.12 (dd, ^3J = 5.8 Hz, ^3J = 1.6 Hz, 1H, C2-H), 5.05 (d, ^2J = 12.1 Hz, 1H, C1'-H^a), 5.01 (^2J = 12.1 Hz, 1H, C1'-H^b), 4.89 – 4.96 (m, 1H, C4-H), 4.72 – 4.76 (m, 1H, C1-H), 3.24 (d, ^3J = 3.5 Hz, 1H, C4a-H), 2.46 (*virt.* dt, ^2J = 13.0 Hz, $^3J \approx {}^3J$ = 3.7 Hz, 1H, C9-H^a), 2.34 – 2.40 (m, 2H, C6-H), 2.01 – 2.12 (m, 1H, C8-H^a), 1.78 – 1.95 (m, 3H, C7-H, C9-H^b), 1.24 – 1.39 (m, 1H, C8-H^b).

^{13}**C NMR** (101 MHz, CD_2Cl_2, 300 K): δ [ppm] = 210.0 (s, C=O), 172.9 (s, COO), 136.6 (d, C1), 136.1 (s, C2'), 135.5 (d, C4), 129.2 (d, C4'), 129.1 (d, C5'), 129.0 (d, C3'), 84.5 (d, C1), 80.8 (d, C1), 67.2 (t, C1'), 61.8 (d, C4a-H), 60.1 (s, C8a), 43.3 (t, C6), 37.5 (t, C9), 27.9 (t, C8), 25.2 (t, C7).

IR (ATR): ṽ (cm^{-1}) = 2933 (w, C-H), 1740 (s, C=O), 1709 (s, C=O), 1140 (s), 1020 (w), 891 (m).

HRMS (ESI): calc. for [M + H^+]: 313.1435; found: 313.1438.

Chiral HPLC: t_{R1} = 12.0 min t_{R2} = 15.0 min (*Daicel* Chiralpak AD-H, 250×4.6 mm, n-Hep/iso-PrOH = 90/10, 1 mL/min, λ = 210 nm).

Benzyl (1*R*,4*S*,4a*S*,9a*R*)-1,4-dimethyl-9-oxo-1,4,5,6,7,8,9,9a-octahydro-4a*H*-1,4-epoxybenzo[7]annulene-4a-carboxylate (4e)

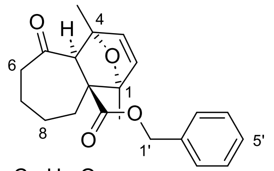

$C_{21}H_{24}O_4$
340.42 g/mol

Benzylation of the primary photoproduct:

According to **GP2** the photoproduct **3e** (12.5 mg, 50.0 µmol, 1.00 eq.) was reacted with K_2CO_3 (10.4 mg, 75.0 µmol, 1.50 eq.) and BnBr (17.3 mg, 11.9 µL, 100 µmol, 2.00 eq.) in DMF (2.0 mL). After column chromatography (silica, Hex/EtOAc = 70/30) the product was obtained as a colorless solid (16.4 mg, 48.2 µmol, 96%).

Catalytic photoinduced Diels-Alder Reaction:

According to **GP 3** carboxylic acid **1** (15.4 mg, 100 µmol, 1.00 eq.) was reacted with catalyst **8a** (9.29 mg, 10.0 µmol, 10 mol%) and 2,5-dimethylfuran (481 mg, 532 µL, 5.00 mmol, 50.0 eq.) in 10 mL CH_2Cl_2. After benzylation and column chromatography (silica, Hex/EtOAc = 95/5 → 90/10) the benzylated product **4e** (28.1 mg, 82.6 µmol, 83%, 20% *ee*) was isolated as a colorless solid.

TLC: R_f = 0.39 (Hex/EtOAc = 70/30) [$KMnO_4$].

M.p.: 104 °C

^{1}H NMR (500 MHz, CD_2Cl_2, 300 K): δ [ppm] = 7.05 – 7.47 (m, 5H, C3'-H, C4'-H, C5'-H), 6.27 (d, ^3J = 5.5 Hz, 1H, C3-H), 5.86 (d, ^3J = 5.5 Hz, 1H, C2-H), 5.01 (s, 2H, C1'-H), 2.90 (s, 1H, C4a-H), 2.25 – 2.38 (m, 3H, C6-H, C9-H^a), 2.03 – 2.14 (m, 1H, C8-H^a), 1.78 – 1.86 (m, 2H, C7-H), 1.68 – 1.78 (m, 1H, C9-H^b), 1.53 (s, 3H, C1-CH_3), 1.51 (s, 3H, C4-CH_3), 1.16 – 1.31 (m, 1H, C8-H^b).

^{13}C NMR (126 MHz, CD_2Cl_2, 300 K): δ [ppm] = 209.9 (s, C=O), 172.9 (s, COO), 139.7 (d, C2), 138.9 (d, C3), 135.9 (s, C2'), 129.0 (d, C4'), 128.9 (d, C3'), 128.8 (d, C5'), 88.6 (s, C4), 86.8 (s, C1), 67.4 (d, C4a), 67.0 (t, C1'), 64.3 (s, C8a), 43.4 (t, C6), 34.9 (t, C9), 27.6 (t, C8), 24.8 (t, C7), 18.1 (q, C4-*C*H_3), 14.9 (q, C1-*C*H_3).

IR (ATR): ṽ (cm^{-1}) = 2937 (w, C-H), 1738 (s, C=O), 1703 (s, C=O), 1151 (s), 1067 (s), 903 (m), 864 (s).

HRMS (ESI): calc. for [M + H^+]: 341.1748; found: 341.1750.

Chiral HPLC: t_{R1} = 7.0 min t_{R2} = 9.6 min (*Daicel* Chiralpak AD-H, 250×4.6 mm, n-Hep/iso-PrOH = 90/10, 1 mL/min, λ = 210 nm).

Crystals suitable for X-ray analysis were obtained by preparing a concentrated solution of *rac*-**4e** in MeCN and allowing the solvent to slowly evaporate at room temperature.

Benzyl (1*R*,4*S*,4a*S*,9a*R*)-1,4-dibutyl-9-oxo-1,4,5,6,7,8,9,9a-octahydro-4a*H*-1,4-epoxybenzo[7]annulene-4a-carboxylate (4h)

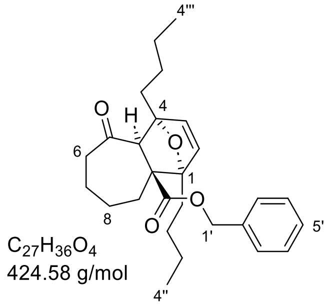

Benzylation of the primary photoproduct:

According to **GP2** the photoproduct *rac*-**3h** (19.5 mg, 58.3 µmol, 1.00 eq.) was reacted with K_2CO_3 (12.1 mg, 87.5 µmol, 1.50 eq.) and BnBr (19.9 mg, 13.9 µL, 117 µmol, 2.00 eq.) in DMF (2.4 mL). After column chromatography (silica, P/Et_2O = 80/20) the product *rac*-**4h** was obtained as a colorless oil (24.7 mg, 58.2 µmol, *quant.*).

Catalytic photoinduced Diels-Alder Reaction:

According to **GP 3** carboxylic acid **1** (15.4 mg, 100 µmol, 1.00 eq.) was reacted with catalyst **8a** (9.29 mg, 10.0 µmol, 10 mol%) and 2,5-dibutylfuran (901 mg, 1.04 mL, 5.00 mmol, 50.0 eq.) in 10 mL CH_2Cl_2. After benzylation and column chromatography (silica, Hex/EtOAc = 90/10 → 80/20) the benzylated product **4h** (13.9 mg, 32.7 µmol, 33%, 16% *ee*) was isolated as a colorless oil.

TLC: R_f = 0.18 (P/Et_2O = 80/20) [$KMnO_4$].

^{1}H NMR (400 MHz, CD_2Cl_2, 300 K): δ [ppm] = 7.25 – 7.44 (m, 5H, C3'-H, C4'-H, C5'-H), 6.14 – 6.32 (m, 1H, C3-H), 5.82 (d, ^3J = 5.6 Hz, 1H, C2-H), 5.04 (d, ^2J = 12.2 Hz, 1H, C1'-H^a), 5.00 (d, ^2J = 12.2 Hz, 1H, C1'-H^b), 2.93 (s, 1H, C4a-H), 2.27 – 2.40 (m, 3H, C6-H, C9-H^a), 2.00 – 2.19 (m, 3H, C1'''-H^a, C1''-H^a, C8-H^a), 1.60 – 1.87 (m, 5H, C9-H^b, C1'''-H^b, C1''-H^b, C7-H), 1.17 – 1.42 (m, 9H, C8-H^b, C3''-H, C4''-H, C3'''-H, C4'''-H), 0.82 – 0.93 (m, 6H, C4''-H, C4'''-H).

^{13}C NMR (101 MHz, CD_2Cl_2, 300 K): δ [ppm] = 210.1 (s, C=O), 173.3 (s, COO), 138.3 (d, C2), 138.2 (d, C3), 136.2 (s, C2'), 129.2 (d, C4'), 129.1 (d, C3'), 128.9 (d, C5'), 92.4 (s, C4), 90.3 (s, C1),

67.1 (t, C1'), 66.6 (d, C4a), 64.8 (s, C8a), 43.6 (t, C6), 35.2 (t, C9), 32.0 (t, C1'''), 28.5 (t, C1''), 28.1 (t, C2'''), 27.9 (t, C8), 27.2 (t, C2''), 25.0 (t, C7), 23.7 (t, C3'''), 23.7 (t, C3''), 14.4 (q, C4'''), 14.3 (q, C4'').

IR (ATR): ṽ (cm^{-1}) = 2928 (m, C-H), 1736 (s, C=O), 1709 (s, C=O), 1456 (w), 1146 (s), 971 (m), 698 (m).

HRMS (ESI): calc. for [M + H^+]: 425.2687; found: 425.2688.

Chiral HPLC: t_{R1} = 19.8 min t_{R2} = 22.0 min (*Daicel* Chiralcel OJ-RH, 150×4.6 mm, MeCN/H_2O = 20/80 → 100/0, 1 mL/min, λ = 215 nm).

Benzyl (4a*R*,9a*S*)-2,3-dimethyl-9-oxo-1,4,5,6,7,8,9,9a-octahydro-4a*H*-benzo[7]annulene-4a-carboxylate (4m)

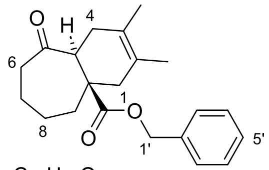

*Synthesis of rac-**4m**:*

Following **GP1** carboxylic acid **1** (15.4 mg, 100 µmol, 1.00 eq.) was reacted with 2,3-dimethylbutadiene (411 mg, 563 µL, 5.00 mmol, 50.0 eq.) in 5 mL CH_2Cl_2. After column chromatography (silica, Hex/EtOAc = 70/30 → EtOAc) the product *rac*-**3m** was obtained as a colorless oil (10.3 mg, 43.6 µmol, 44%).

*Compound rac-**3m** showed strongly broadened signals in the respective ^1H and ^{13}C NMR spectra. This is attributed to an equilibrium between the opened and the closed lactol/ketone form of the molecule. The compound was therefore not completely characterized.*

According to **GP2** the photoproduct *rac*-**3m** (15.7 mg, 66.4 µmol, 1.00 eq.) was reacted with K_2CO_3 (13.8 mg, 100 µmol, 1.50 eq.) and BnBr (22.7 mg, 15.8 µL, 133 µmol, 2.00 eq.) in DMF (2.7 mL). After column chromatography (silica, Hex/EtOAc = 90/10 → 80/20) the product *rac*-**4m** was obtained as a colorless oil (20.6 mg, 63.1 µmol, 95%).

Catalytic photoinduced Diels-Alder Reaction:

According to **GP 3** carboxylic acid **1** (15.4 mg, 100 µmol, 1.00 eq.) was reacted with catalyst **8a** (9.29 mg, 10.0 µmol, 10 mol%) and 2,3-dimethylbutadiene (411 mg, 566 µL, 5.00 mmol, 50.0 eq.) in 10 mL CH_2Cl_2. After benzylation and column chromatography (silica, P/Et_2O = 90/10 → 85/15) the benzylated product **4m** (7.8 mg, 23.9 µmol, 24%, 25% *ee*) was obtained as a colorless oil.

TLC: R_f = 0.23 (Hex/EtOAc = 80/20) [$KMnO_4$].

^{1}H NMR (400 MHz, CD_2Cl_2, 300 K): δ [ppm] = 7.30 – 7.38 (m, 3H, C4'-H, C5'-H), 7.23 – 7.29 (m, 2H, C3'-H), 5.15 (d, ^2J = 12.4 Hz, 1H, C1'-H^a), 5.02 (d, ^2J = 12.4 Hz, 1H, C1'-H^b), 2.33 – 2.64 (m, 5H, C1-H^a, C4-H^a, C6-H, C4a-H), 2.07 – 2.28 (m, 3H, C1-H^b, C4-H^b, C9-H^a), 1.72 – 1.88 (m, 2H, C7-H^a, C8-H^a), 1.61 – 1.70 (m, 1H, C9-H^b), 1.52 – 1.58 (m, 7H, 2 × CH_3, C7-H^b), 1.37 – 1.49 (m, 1H, C8-H^b).

^{13}C NMR (101 MHz, CD_2Cl_2, 300 K): δ [ppm] = 213.0 (s, C=O), 175.1 (s, COO), 136.8 (s, C2'), 129.0 (d, C4'), 128.6 (d, C3'), 128.5 (d, C5'), 125.8 (s, C2), 123.5 (s, C3), 66.7 (t, C1'), 53.9 (d, C4a), 48.6 (s, C8a), 45.3 (t, C1), 44.3 (t, C6), 41.2 (t, C9), 33.5 (t, C4), 25.4 (t, C8), 23.7 (t, C7), 18.8 (q, C3-*C*H_3), 18.6 (q, C2-*C*H_3).

IR (ATR): ṽ (cm^{-1}) = 2924 (m, C-H), 1725 (s, C=O), 1682 (s), 1454 (m), (s, 1170), 697 (s).

HRMS (ESI): calc. for [M + H^+]: 327.1955; found: 327.1956.

Chiral HPLC: t_{R1} = 6.2 min, t_{R2} = 6.9 min (*Daicel* Chiralpak AD-H, 250×4.6 mm, n-Hep/iso-PrOH = 90/10, 1 mL/min, λ = 210 nm).

Benzyl 10-oxo-1,2,3,4,5,6,7,8,9,10,10a,11-dodecahydro-5a*H*-cyclohepta[*b*]naphthalene-5a-carboxylate (*rac*-4n)

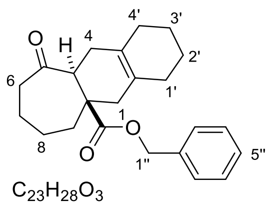

$C_{23}H_{28}O_3$
352.47 g/mol

*Synthesis of rac-**3n**:*

Following **GP1** carboxylic acid **1** (15.4 mg, 100 µmol, 1.00 eq.) was reacted with 1,2-dimethylenecyclohexane (216 mg, 2.00 mmol, 20.0 eq.) in 5 mL CH_2Cl_2. After column

chromatography (silica, Hex/EtOAc = 70/30 → EtOAc + 0.1% AcOH) the product *rac*-**3n** was obtained as a colorless solid (22.9 mg, 87.3 µmol, 87%).

*Compound rac-**3n** showed strongly broadened signals in the respective ^{1}H and ^{13}C NMR spectra. This is attributed to an equilibrium between the opened and the closed lactol/ketone form of the molecule. The compound was therefore not completely characterized.*

According to **GP2** the photoproduct *rac*-**3n** (22.9 mg, 87.3 µmol, 1.00 eq.) was reacted with K_2CO_3 (18.1 mg, 131 µmol, 1.50 eq.) and BnBr (29.9 mg, 20.8 µL, 175 µmol, 2.00 eq.) in DMF (3.5 mL) for 0.5 h. After column chromatography (silica, Hex/EtOAc = 90/10) the product *rac*-**4n** was obtained as a colorless oil (12.1 mg, 34.3 µmol, 39%).

TLC: R_f = 0.17 (Hex/EtOAc = 90/10) [$KMnO_4$].

^{1}H NMR (400 MHz, CD_2Cl_2, 300 K): δ [ppm] = 7.18 – 7.40 (m, 5H, C3”-H, C4”-H, C5”-H), 5.18 (d, ^{2}J = 12.4 Hz, 1H, C1”-H^a), 5.01 (d, ^{2}J = 12.4 Hz, 1H, C1”-H^b), 2.46 – 2.67 (m, 3H, C6-H , C4a-H), 2.21 – 2.40 (m, 3H, C1-H^a, C4-H^a, C9-H^a), 2.01 – 2.16 (m, 2H, C1-H^b, C4-H^b), 1.73 – 1.89 (m, 6H, C7-H^a, C8-H^a, C1’-H, C4’-H), 1.53 – 1.67 (m, 4H, C7-H^b, C9-H^b, C2’-H), 1.36 – 1.50 (m, 3H, C8-H^b, C3’-H).

^{13}C NMR (126 MHz, CD_2Cl_2, 300 K): δ [ppm] = 213.1 8 (s, C=O), 175.1 (s, COO), 136.9 (s, C2”), 128.9 (d, C4”-H), 128.6 (d, C5”-H), 128.5 (d, C3”), 128.0 (s, C2), 125.8 (s, C3), 66.6 (t, C1”), 53.9 (d, C4a), 48.5 (s, C8a), 44.2 (t, C6), 44.1 (t, C1), 41.1 (t, C9), 32.3 (t, C4), 30.1 (t, C1’), 29.9 (t, C4’), 25.3 (t, C8), 23.7 (t, C7), 23.6 (t, C2’), 23.5 (t, C3’).

IR (ATR): ṽ (cm^{-1}) = 2927 (m, C-H), 1724 (s, C=O), 1683 (s), 1453 (m), 1152 (s), 698 (s).

HRMS (ESI): calc. for [M + H^+]: 353.2117; found: 353.2119.

9. Iodolactonization of Compound *rac*-3e

11-Iodo-3a,5-dimethyloctahydro-1*H*,6*H*-3,5-methanocyclohepta[*c*]furo[3,4-*b*]furan-1,6-dione (*rac*-6)

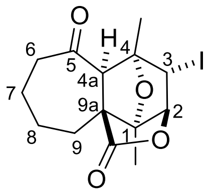

$C_{14}H_{17}IO_4$
376.19 g/mol

According to a modified literature procedure:[50] Photoproduct *rac*-**3e** (20.0 mg, 79.9 µmol, 1.00 eq.) was dissolved in 760 µL MeCN and 30 µL water and iodine (99.4 mg, 392 µmol, 4.90 eq.) were added. The obtained solution was stirred for two hours, and the reaction was stopped by addition of sat. $Na_2S_2O_{3aq}$ (2 mL). The phases were separated, and the aqueous phase was extracted with Et_2O (3 × 3 mL), the combined organic phases were dried over Na_2SO_4, filtered and the solvent was removed under reduced pressure. The crude product was purified by column chromatography (silica, Hex/EtOAc = 90/10) and lactone *rac*-**6** (25.2 mg, 67.0 µmol, 84%) was obtained as a colorless solid.

TLC: R_f = 0.69 (Hex/EtOAc = 50/50) [$KMnO_4$].

M.p.: 144 °C

^{1}H NMR (400 MHz, CD_2Cl_2, 300 K): δ [ppm] = 4.88 (s, 1H, C2-H), 4.73 (s, 1H, C3-H), 3.51 (s, 1H, C4a-H), 2.48 – 2.64 (m, 2H, C6-H^a, C7-H^a), 2.41 (ddd, ^2J = 19.8 Hz, ^3J = 12.1 Hz, ^3J = 2.8 Hz, 1H, C6-H^b), 1.92 – 2.04 (m, 3H, C9-H^a, C8-H), 1.75 – 1.90 (m, 1H, C7-H^b), 1.57 – 1.69 (m, 1H, C9-H^b), 1.51 (s, 6H, C1-CH_3, C4-CH_3).

^{13}C NMR (101 MHz, CD_2Cl_2, 300 K): δ [ppm] = 209.3 (s, C5), 176.7 (s, COO), 93.4 (d, C2), 93.4 (s, C1), 86.3 (s, C4), 67.5 (d, C4a), 53.6 (s, C9a), 43.2 (t, C6), 33.6 (d, C3), 31.2 (t, C9), 24.4 (q, C1-*C*H_3), 24.0 (t, C7), 23.8 (t, C8), 14.5 (q, C4-*C*H_3).

IR (ATR): ṽ (cm^{-1}) = 2926 (w, C-H), 1772 (s, C=O), 1698 (s, C=O), 1345 (m), 1033 (s), 841 (m).

HRMS (ESI): calc. for [M + H$^+$]: 377.0250; found: 377.0255.

10. Determination of the Absolute Configuration

4-Bromobenzyl (1*R*,4*S*,4a*S*,9a*R*)-1,4-dimethyl-9-oxo-1,4,5,6,7,8,9,9a-octahydro-4a*H*-1,4-epoxybenzo[7]annulene-4a-carboxylate (5)

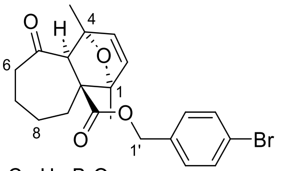

$C_{21}H_{23}BrO_4$
419.32 g/mol

Racemic Synthesis:

Following **GP1** carboxylic acid **1** (30.8 mg, 200 µmol, 1.00 eq.) was reacted with 2,5-dimethylfuran (961 mg, 1.06 mL, 50.0 mmol, 50.0 eq.) in 10 mL CH_2Cl_2. The solvent was removed under reduced pressure and the crude product was subjected to analogous conditions as described in **GP2** using 4-bromobenzyl bromide for the benzylation. Column chromatography (silica, Hex/EtOAc = 80/20) yielded *rac*-**5** (69.3 mg, 165 µmol, 83%) as a colorless solid.

Catalytic photoinduced Diels-Alder Reaction:

According to **GP 3** carboxylic acid **1** (15.4 mg, 100 µmol, 1.00 eq.) was reacted with catalyst **8a** (9.29 mg, 10.0 µmol, 10 mol%) and 2,5-dimethylfuran (481 mg, 532 µL, 5.00 mmol, 50.0 eq.) in 10 mL CH_2Cl_2. After benzylation (using 4-bromobenzyl bromide) and column chromatography (silica, P/Et_2O = 80/20 → 70/30) the benzylated product **5** (28.8 mg, 68.7 µmol, 69%, 18% *ee*) was isolated as a colorless solid.

TLC: R_f = 0.40 (P/Et_2O = 50/50) [$KMnO_4$].

M.p.: 129 °C

^{1}H NMR (400 MHz, CD_2Cl_2, 300 K): δ [ppm] = 7.46 – 7.54 (m, 2H, C4'-H), 7.18 – 7.29 (m, 2H, C3'-H), 6.28 (d, ^3J = 5.6 Hz, 1H, C3-H), 5.86 (d, ^3J = 5.6 Hz, 1H, C2-H), 4.93 – 5.02 (m, 2H, C1'-H), 2.91 (s, 1H, C4a-H), 2.27 – 2.37 (m, 3H, C6-H, C9-H^a), 2.00 – 2.15 (m, 1H, C8-H^a), 1.67 – 1.88 (m, 3H, C7-H, C9-H^b), 1.53 (s, 3H, C4-CH_3), 1.51 (s, 3H, C1-CH_3), 1.12 – 1.30 (m, 1H, C8-H^b).

^{13}C NMR (126 MHz, CD_2Cl_2, 300 K): δ [ppm] = 209.7 (s, C5), 172.9 (s, COO), 139.7 (d, C2), 139.1 (d, C3), 135.0 (s, C2'), 132.1 (d, C4'), 130.9 (d, C3'), 122.8 (s, C5'), 88.7 (s, C1), 86.8 (s, C4), 67.5 (d, C4a), 66.2 (t, C1'), 64.3 (s, C8a), 43.4 (t, C6), 34.9 (t, C9), 27.6 (t, C8), 24.8 (t, C7), 18.2 (q, C1-*C*H_3), 15.0 (q, C4-*C*H_3).

IR (ATR): ṽ (cm^{-1}) = 2924 (w, C-H), 1737 (s, C=O), 1699 (s, C=O), 1449 (m), 1145 (s), 968 (m), 798 (s).

HRMS (ESI): calc. for [M + H^+]: 419.0853; found: 419.0860.
Chiral HPLC: t_{R1} = 8.5 min t_{R2} = 13.8 min (*Daicel* Chiralpak AD-H, 250×4.6 mm, n-Hep/iso-PrOH = 90/10, 1 mL/min, λ = 210 nm).

Determination of the Absolute Configuration:

The enantiomers of the racemate were separated by semi-preparative HPLC on a chiral stationary phase (Daicel Chiralpak AD-H, 250×20 mm, n-Hep/iso-PrOH = 90/10) to obtain enantiomerically pure material for crystallization.

Crystals suitable for X-ray analysis of *ent*-**5** were obtained by preparing a concentrated solution of the enantiomerically pure material in CH_2Cl_2 and allowing the solvent to slowly evaporate at room temperature.

*SC-XRD Structure Report of ent-**5***:

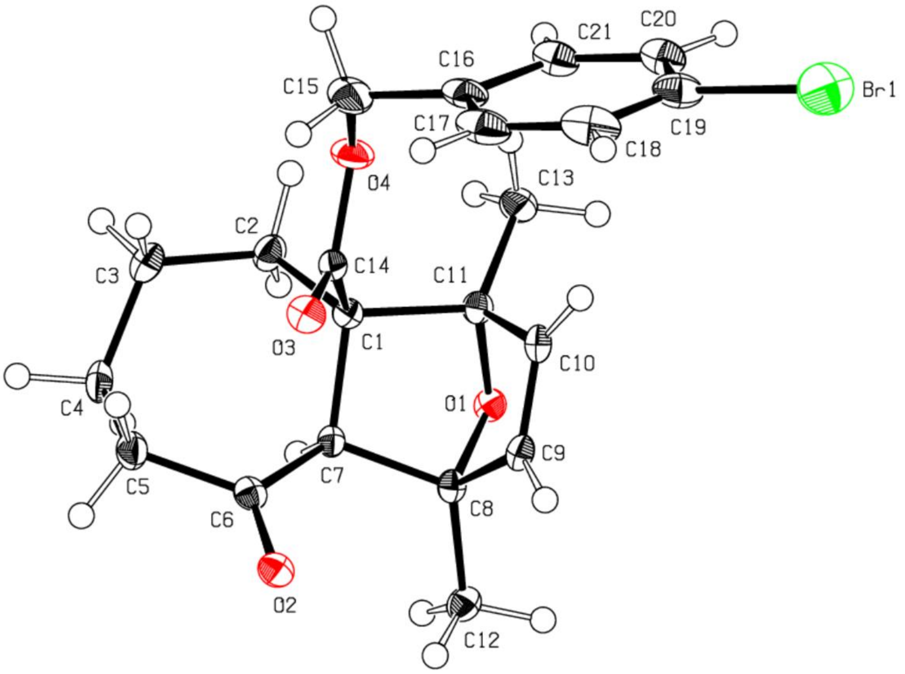

A colorless, block-shaped crystal of $C_{21}H_{23}BrO_4$ coated with perfluorinated ether and fixed on top of a *Kapton* micro sampler was used for X-ray crystallographic analysis. The X-ray intensity data were collected at 100(2) K on a Bruker D8 VENTURE Duo three-angle diffractometer with an IMS microsource with MoK_α radiation (λ=0.71073 Å) using APEX4.[51] The diffractometer was equipped with a Helios optic monochromator, a Bruker PHOTON II detector, and an Oxford Cryostream low temperature device.

A matrix scan was used to determine the initial lattice parameters. All data were integrated with the Bruker SAINT V8.40B software package using a narrow-frame algorithm and the reflections were corrected for Lorentz and polarization effects, scan speed, and background.[52] The integration of the data using a monoclinic unit cell yielded a total of 72707 reflections within a 2θ range [°] of 5.18 to 55.76 (0.76 Å), of which 4497 were independent. Data were corrected for absorption effects including odd and even ordered spherical harmonics by the multi-scan method (SADABS 2016/2).[53] Space group assignment was based upon systematic absences, E statistics, and successful refinement of the structure.

The structure was solved by direct methods using SHELXT and refined by full-matrix least-squares methods against F^2 by minimizing $\Sigma w(F_o^2 - F_c^2)^2$ using SHELXL in conjunction with SHELXLE.[54–56] All non-hydrogen atoms were refined with anisotropic displacement parameters. Hydrogen atoms were refined isotropically on calculated positions using a riding model with their U_{iso} values constrained to 1.5 times the U_{eq} of their pivot atoms for terminal sp^3 carbon atoms and a C–H distance of 0.98 Å. Non-methyl hydrogen atoms were refined using a riding model with methylene, aromatic, and other C–H distances of 0.99 Å, 0.95 Å, and 1.00 Å, respectively, and U_{iso} values constrained to 1.2 times the U_{eq} of their pivot atoms.

Neutral atom scattering factors for all atoms and anomalous dispersion corrections for the non-hydrogen atoms were taken from International Tables for Crystallography.[57] Supplementary crystallographic data reported in this paper have been deposited with the Cambridge Crystallographic Data Centre (CCDC 2409857) and can be obtained free of charge from The Cambridge Crystallographic Data Centre via www.ccdc.cam.ac.uk/structures.[58] This report and the CIF file were generated using FinalCif.[59]

Table S10.1. Crystal data and structure refinement for *ent*-**5**.

CCDC number	CCDC 2409857
Empirical formula	$C_{21}H_{23}BrO_4$
Formula weight	419.30
Temperature [K]	100(2)

Crystal system	monoclinic
Space group (number)	$P2_1$ (4)
a [Å]	8.1668(7)
b [Å]	10.1528(7)
c [Å]	11.8358(10)
α [°]	90
β [°]	105.845(3)
γ [°]	90
Volume [$Å^3$]	944.09(13)
Z	2
ρ_{calc} [gcm^{-3}]	1.475
μ [mm^{-1}]	2.200
F(000)	432
Crystal size [mm^3]	0.210×0.461×0.846
Crystal colour	colourless
Crystal shape	block
Radiation	MoK_α (λ=0.71073 Å)
2θ range [°]	5.18 to 55.76 (0.76 Å)
Index ranges	$-10 \leq h \leq 10$<br>$-13 \leq k \leq 13$<br>$-15 \leq l \leq 15$
Reflections collected	72707
Independent reflections	4497<br>R_{int} = 0.0452<br>R_{sigma} = 0.0242
Completeness to θ = 25.242°	99.9 %
Data / Restraints / Parameters	4497 / 1 / 237
Goodness-of-fit on F^2	1.072
Final *R* indexes [$I \geq 2\sigma(I)$]	R_1 = 0.0266<br>wR_2 = 0.0557
Final *R* indexes [all data]	R_1 = 0.0326<br>wR_2 = 0.0596
Largest peak/hole [$eÅ^{-3}$]	0.41/−0.58
Flack parameter	0.034(3)

For crystallization, the later-eluting enantiomer E2 was used, corresponding to the minor enantiomer of the photochemical reaction. For comparison the HPLC trace of the racemate (*rac*-**5**) (top left), the trace of the photochemical reaction product (**5**) (top right) as well as the traces of the enantiomerically pure material of E1 (**5**) (bottom left) and E2 (*ent*-**5**, used for crystallization, bottom right) are shown.

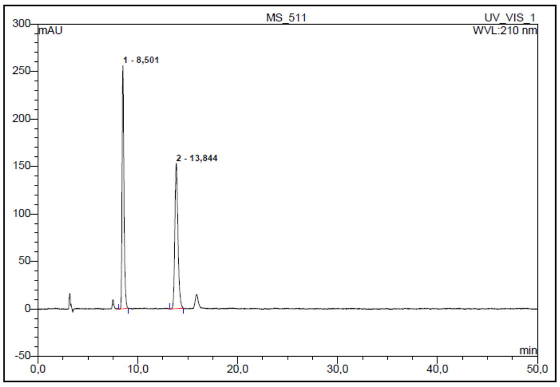

No.	Ret.Time min	Peak Name	Height mAU	Area mAU*min	Rel.Area %	Amount	Type
1	8,50	n.a.	256,033	55,626	50,32	n.a.	BMB*
2	13,84	n.a.	152,921	54,922	49,68	n.a.	BMB*
Total:			408,954	110,548	100,00	0,000	

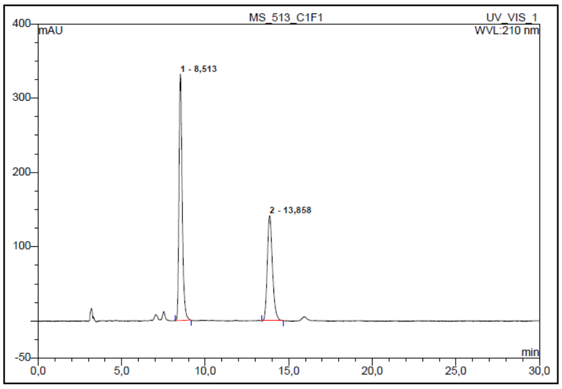

No.	Ret.Time min	Peak Name	Height mAU	Area mAU*min	Rel.Area %	Amount	Type
1	8,51	n.a.	331,906	73,219	59,19	n.a.	BMB*
2	13,86	n.a.	141,402	50,487	40,81	n.a.	BMB*
Total:			473,308	123,706	100,00	0,000	

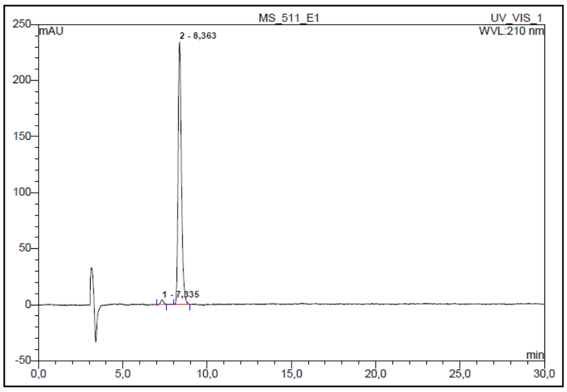

No.	Ret.Time min	Peak Name	Height mAU	Area mAU*min	Rel.Area %	Amount	Type
1	7,33	n.a.	4,311	0,849	1,56	n.a.	BMB*
2	8,36	n.a.	234,182	53,573	98,44	n.a.	BMB*
Total:			238,494	54,422	100,00	0,000	

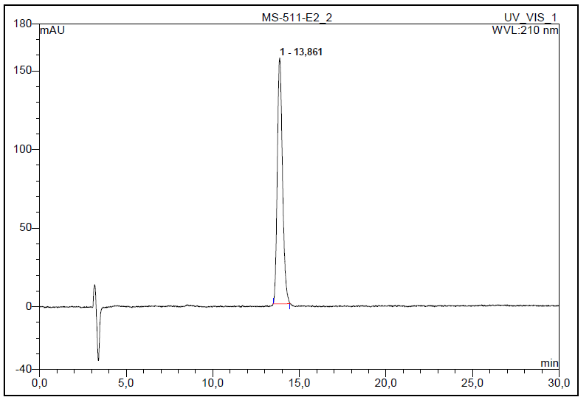

No.	Ret.Time min	Peak Name	Height mAU	Area mAU*min	Rel.Area %	Amount	Type
1	13,86	n.a.	156,512	54,373	100,00	n.a.	BMB
Total:			156,512	54,373	100,00	0,000	

11. Additional Experiments

Reaction of S3 with 2,5-Dimethylfuran under Direct Irradiation

In analogy to **GP1** benzylated carboxylic acid **S3** (12.2 mg, 50.0 µmol, 1.00 eq.) was reacted with 2,5-dimethylfuran (240 mg, 266 µL, 2.50 mmol, 50 eq.) in 2.5 mL CH_2Cl_2. After column chromatography (silica, Hex/EtOAc 80/20) the product *rac*-**4e** was obtained as a colorless solid (15.5 mg, 45.5 µmol, 91%). NMR data matched the previously reported data.

Reaction of S3 with 2,5-Dimethylfuran under Sensitizing Conditions Employing Catalyst 7

In analogy to **GP 3** benzylated carboxylic acid **S3** (12.2 mg, 50.0 µmol, 1.00 eq.) was reacted with catalyst **7** (4.60 mg, 5.00 µmol, 10 mol%) and 2,5-dimethylfuran (240 mg, 266 µL, 2.50 mmol, 50.0 eq.) in 5 mL CH_2Cl_2. After 22 h of irradiation the solvent was removed under reduced pressure and the crude product was analyzed by ^{1}H NMR, revealing no characteristic signals of the expected *Diels-Alder* product.

Reaction of 1 with 2,5-Dimethylfuran under Direct Irradiation using 50 mol% (*R*)-TRIP

In analogy to **GP1** carboxylic acid **1** (7.71 mg, 50.0 µmol, 1.00 eq.) was reacted with 2,5-dimethylfuran (240 mg, 266 µL, 2.50 mmol, 50 eq.) and (*R*)-TRIP (18.8 mg, 25.0 µmol, 0.50 eq.) in 2.5 mL CH_2Cl_2. According to **GP2** the crude product was reacted with K_2CO_3 (10.4 mg, 75.0 µmol, 1.50 eq.) and BnBr (17.1 mg, 11.9 µL, 100 µmol, 2.00 eq.) in DMF (1.25 mL) for 1 h. After column chromatography (silica, Hex/EtOAc = 80/20) *rac*-**4e** was obtained as a colorless solid (14.1 mg, 41.4 µmol, 83%). Chiral HPLC analysis revealed a racemic sample.

12. Additional Crystal Structures

*SC-XRD Structure Report of rac-**3a***:

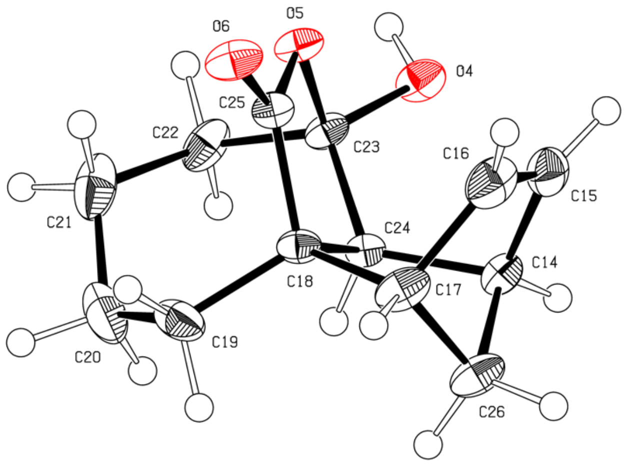

A colorless, triangle-shaped crystal of $C_{13}H_{16}O_3$ coated with perfluorinated ether and fixed on top of a Kapton micro sampler was used for X-ray crystallographic analysis. The X-ray intensity data were collected at 100(2) K on a Bruker D8 VENTURE three-angle diffractometer with a TXS rotating anode with MoK_{α} radiation (λ=0.71073 Å) using APEX4.[51] The diffractometer was equipped with a Helios optic monochromator, a Bruker PHOTON III detector, and an Oxford Cryostream low temperature device.

A matrix scan was used to determine the initial lattice parameters. All data were integrated with the Bruker SAINT (Bruker, software package using a narrow-frame algorithm and the reflections were corrected for Lorentz and polarisation effects, scan speed, and background.[52] The integration of the data using a monoclinic unit cell yielded a total of 50849 reflections within a 2θ range [°] of 4.17 to 52.81 (0.80 Å), of which 4643 were independent. Data were corrected for absorption effects including odd and even ordered spherical harmonics by the multi-scan method (SADABS 2016/2).[53] Space group assignment was based upon systematic absences, E statistics, and successful refinement of the structure.

The structure was solved by direct methods using SHELXT and refined by full-matrix least-squares methods against F^2 by minimizing $\Sigma w(F_o^2 - F_c^2)^2$ using SHELXL in conjunction with SHELXLE.[54–56] All non-hydrogen atoms were refined with anisotropic displacement parameters. Hydrogen atoms were refined isotropically on calculated positions using a riding model with their U_{iso} values constrained to 1.5 times the U_{eq} of their pivot atoms for terminal sp^3 carbon atoms and a C–H distance of 0.98 Å. Non-methyl hydrogen atoms were refined using

a riding model with methylene, aromatic, and other C–H distances of 0.99 Å, 0.95 Å, and 1.00 Å, respectively, and U_{iso} values constrained to 1.2 times the U_{eq} of their pivot atoms.

Neutral atom scattering factors for all atoms and anomalous dispersion corrections for the non-hydrogen atoms were taken from International Tables for Crystallography.[57] Supplementary crystallographic data reported in this paper have been deposited with the Cambridge Crystallographic Data Centre (CCDC 2409855) and can be obtained free of charge from The Cambridge Crystallographic Data Centre via www.ccdc.cam.ac.uk/structures.[58] This report and the CIF file were generated using FinalCif.[59]

Table S12.1. Crystal data and structure refinement for *rac*-**3a**.

CCDC number	CCDC 2409855
Empirical formula	$C_{13}H_{16}O_3$
Formula weight	220.26
Temperature [K]	100(2)
Crystal system	monoclinic
Space group (number)	$P2_1/c$ (14)
a [Å]	14.7781(10)
b [Å]	13.1621(8)
c [Å]	11.8310(8)
α [°]	90
β [°]	99.468(3)
γ [°]	90
Volume [Å^3]	2269.9(3)
Z	8
ρ_{calc} [gcm^{-3}]	1.289
μ [mm^{-1}]	0.091
F(000)	944
Crystal size [mm^3]	0.250×0.751×0.898
Crystal colour	colourless
Crystal shape	triangle
Radiation	MoK_α (λ=0.71073 Å)
2θ range [°]	4.17 to 52.81 (0.80 Å)
Index ranges	−18 ≤ h ≤ 18<br>−16 ≤ k ≤ 16<br>−14 ≤ l ≤ 14
Reflections collected	50849
Independent reflections	4643<br>R_{int} = 0.0503<br>R_{sigma} = 0.0237
Completeness to θ = 25.242°	99.9
Data / Restraints / Parameters	4643 / 45 / 314
Goodness-of-fit on F^2	1.102

Final *R* indexes [$I \geq 2\sigma(I)$]	R_1 = 0.0447<br>wR_2 = 0.1143
Final *R* indexes [all data]	R_1 = 0.0473<br>wR_2 = 0.1160
Largest peak/hole [eÅ^{-3}]	0.36/−0.23

*SC-XRD Structure Report of rac-**3e**:*

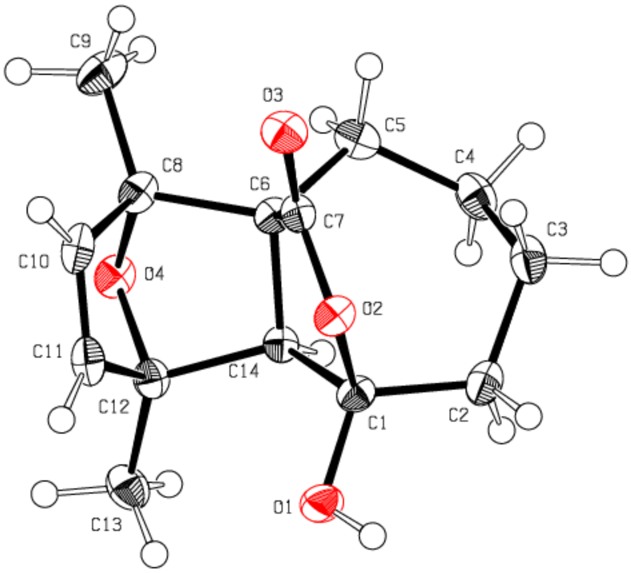

A colorless, block-shaped crystal of $C_{14}H_{18}O_4$ coated with perfluorinated ether and fixed on top of a Kapton micro sampler was used for X-ray crystallographic analysis. The X-ray intensity data were collected at 100(2) K on a Bruker D8 VENTURE three-angle diffractometer with a TXS rotating anode with MoK_α radiation (λ=0.71073 Å) using APEX4.[51] The diffractometer was equipped with a Helios optic monochromator, a Bruker PHOTON III detector, and an Oxford Cryostream low temperature device.

A matrix scan was used to determine the initial lattice parameters. All data were integrated with the Bruker SAINT V8.40B software package using a narrow-frame algorithm and the reflections were corrected for Lorentz and polarisation effects, scan speed, and background.[52] The integration of the data using a orthorhombic unit cell yielded a total of 90040 reflections within a 2θ range [°] of 4.17 to 52.80 (0.80 Å), of which 2567 were independent. Data were corrected for absorption effects including odd and even ordered spherical harmonics by the

multi-scan method SADABS.[53] Space group assignment was based upon systematic absences, E statistics, and successful refinement of the structure.

The structure was solved by direct methods using SHELXT and refined by full-matrix least-squares methods against F^2 by minimizing $\Sigma w(F_o^2 - F_c^2)^2$ using SHELXL in conjunction with SHELXLE.[54–56] All non-hydrogen atoms were refined with anisotropic displacement parameters. Hydrogen atoms were refined isotropically on calculated positions using a riding model with their U_{iso} values constrained to 1.5 times the U_{eq} of their pivot atoms for terminal sp^3 carbon atoms and a C–H distance of 0.98 Å. Non-methyl hydrogen atoms were refined using a riding model with methylene, aromatic, and other C–H distances of 0.99 Å, 0.95 Å, and 1.00 Å, respectively, and U_{iso} values constrained to 1.2 times the U_{eq} of their pivot atoms.

Neutral atom scattering factors for all atoms and anomalous dispersion corrections for the non-hydrogen atoms were taken from International Tables for Crystallography.[57] Supplementary crystallographic data reported in this paper have been deposited with the Cambridge Crystallographic Data Centre (CCDC 2409858) and can be obtained free of charge from The Cambridge Crystallographic Data Centre via www.ccdc.cam.ac.uk/structures.[58] This report and the CIF file were generated using FinalCif.[59]

Table S12.2. Crystal data and structure refinement for *rac*-**3e**.

CCDC number	CCDC 2409858
Empirical formula	$C_{14}H_{18}O_4$
Formula weight	250.28
Temperature [K]	100(2)
Crystal system	orthorhombic
Space group (number)	$Pbca$ (61)
a [Å]	8.5857(9)
b [Å]	14.8387(16)
c [Å]	19.523(2)
α [°]	90
β [°]	90
γ [°]	90
Volume [Å^3]	2487.2(5)
Z	8
ρ_{calc} [gcm^{-3}]	1.337
μ [mm^{-1}]	0.097
F(000)	1072
Crystal size [mm^3]	0.064×0.430×0.590
Crystal colour	colourless
Crystal shape	block
Radiation	MoK_α (λ=0.71073 Å)
2θ range [°]	4.17 to 52.80 (0.80 Å)

Index ranges	$-10 \leq h \leq 10$<br>$-18 \leq k \leq 18$<br>$-24 \leq l \leq 24$
Reflections collected	90040
Independent reflections	2567<br>R_{int} = 0.0754<br>R_{sigma} = 0.0165
Completeness to θ = 25.242°	100.0 %
Data / Restraints / Parameters	2567 / 0 / 168
Goodness-of-fit on F^2	1.139
Final R indexes [$I \geq 2\sigma(I)$]	R_1 = 0.0384<br>wR_2 = 0.0911
Final R indexes [all data]	R_1 = 0.0449<br>wR_2 = 0.0939
Largest peak/hole [eÅ^{-3}]	0.29/−0.23

*SC-XRD Structure Report of rac-**4e**:*

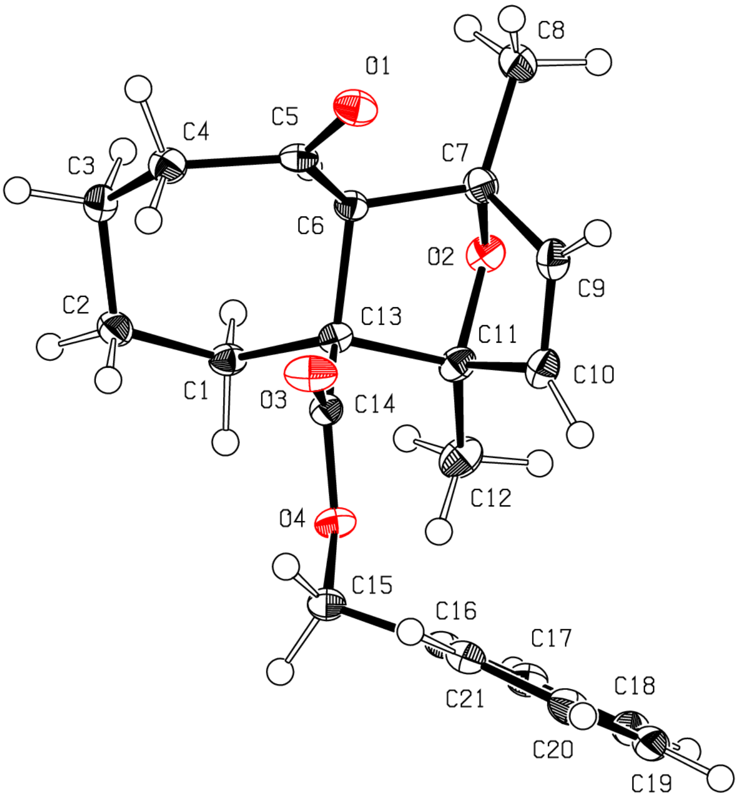

A colorless, block-shaped crystal of $C_{21}H_{24}O_4$ coated with perfluorinated ether and fixed on top of a Kapton micro sampler was used for X-ray crystallographic analysis. The X-ray intensity

data were collected at 100(2) K on a Bruker D8 VENTURE three-angle diffractometer with a TXS rotating anode with MoK_α radiation (λ=0.71073 Å) using APEX4.[51] The diffractometer was equipped with a Helios optic monochromator, a Bruker PHOTON III detector, and an Oxford Cryostream low temperature device.

A matrix scan was used to determine the initial lattice parameters. All data were integrated with the Bruker SAINT V8.40B software package using a narrow-frame algorithm and the reflections were corrected for Lorentz and polarisation effects, scan speed, and background.[52] The integration of the data using a monoclinic unit cell yielded a total of 86516 reflections within a 2θ range [°] of 4.74 to 52.81 (0.80 Å), of which 3498 were independent. Data were corrected for absorption effects including odd and even ordered spherical harmonics by the multi-scan method (SADABS 2016/2).[53] Space group assignment was based upon systematic absences, E statistics, and successful refinement of the structure.

The structure was solved by direct methods using SHELXT and refined by full-matrix least-squares methods against F^2 by minimizing $\Sigma w(F_o^2 - F_c^2)^2$ using SHELXL in conjunction with SHELXLE.[54–56] All non-hydrogen atoms were refined with anisotropic displacement parameters. Hydrogen atoms were refined isotropically on calculated positions using a riding model with their U_{iso} values constrained to 1.5 times the U_{eq} of their pivot atoms for terminal sp^3 carbon atoms and a C–H distance of 0.98 Å. Non-methyl hydrogen atoms were refined using a riding model with methylene, aromatic, and other C–H distances of 0.99 Å, 0.95 Å, and 1.00 Å, respectively, and U_{iso} values constrained to 1.2 times the U_{eq} of their pivot atoms.

Neutral atom scattering factors for all atoms and anomalous dispersion corrections for the non-hydrogen atoms were taken from International Tables for Crystallography.[57] Supplementary crystallographic data reported in this paper have been deposited with the Cambridge Crystallographic Data Centre (CCDC **2409856**) and can be obtained free of charge from The Cambridge Crystallographic Data Centre via www.ccdc.cam.ac.uk/structures.[58] This report and the CIF file were generated using FinalCif.[59]

Table S12.3. Crystal data and structure refinement for *rac*-**4e**.

CCDC number	CCDC 2409856
Empirical formula	$C_{21}H_{24}O_4$
Formula weight	340.40
Temperature [K]	100(2)
Crystal system	monoclinic
Space group (number)	$P2_1/c$ (14)
a [Å]	13.5720(7)
b [Å]	7.3479(4)
c [Å]	18.0819(7)

α [°]	90
β [°]	108.275(2)
γ [°]	90
Volume [Å^3]	1712.28(15)
Z	4
ρ_{calc} [gcm^{-3}]	1.320
μ [mm^{-1}]	0.090
F(000)	728
Crystal size [mm^3]	0.088×0.224×0.331
Crystal colour	colourless
Crystal shape	block
Radiation	MoK_α (λ=0.71073 Å)
2θ range [°]	4.74 to 52.81 (0.80 Å)
Index ranges	−16 ≤ h ≤ 16<br>−9 ≤ k ≤ 9<br>−22 ≤ l ≤ 22
Reflections collected	86516
Independent reflections	3498<br>R_{int} = 0.0445<br>R_{sigma} = 0.0168
Completeness to θ = 25.242°	99.7 %
Data / Restraints / Parameters	3498 / 0 / 228
Goodness-of-fit on F^2	1.055
Final R indexes [I≥2σ(I)]	R_1 = 0.0383<br>wR_2 = 0.0978
Final R indexes [all data]	R_1 = 0.0404<br>wR_2 = 0.0998
Largest peak/hole [eÅ^{-3}]	0.29/−0.24

13. Emission Spectra and Triplet Energy Measurements

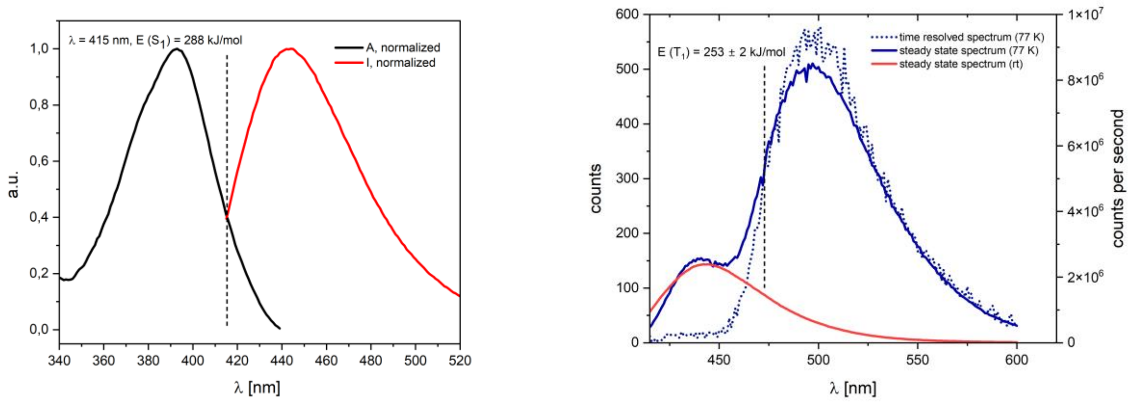

Figure S13.1. *Left:* UV/VIS spectrum of **8a** in dichloromethane (c = 50 μM) normalized to the absorption maximum at 393 nm; luminescence of **8a** in dichloromethane (c = 50 μM) at room temperature, normalized to the emission maximum at 444 nm. *Right:* Steady state spectra of **8a** in dichloromethane (c = 50 μM) at room temperature and 77 K given in counts per second (solid lines), time resolved spectrum of **8a** in dichloromethane (c = 50 μM) at 77 K after 100 μs delay in counts (dashed line).

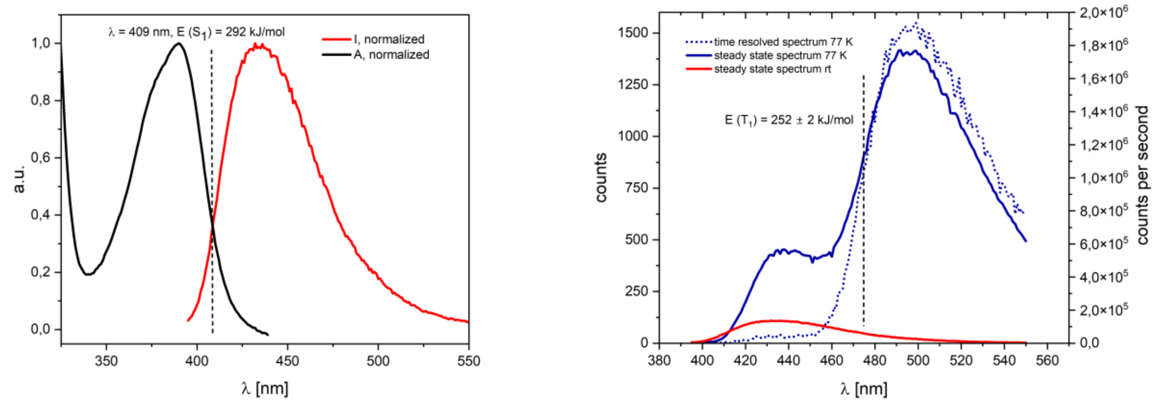

Figure S13.2. *Left:* UV/VIS spectrum of **8b** in dichloromethane (c = 50 μM) normalized to the absorption maximum at 390 nm; luminescence of **8b** in dichloromethane (c = 50 μM) at room temperature, normalized to the emission maximum at 432 nm. *Right:* Steady state spectra of **8b** in dichloromethane (c = 50 μM) at room temperature and 77 K given in counts per second (solid lines), time resolved spectrum of **8b** in dichloromethane (c = 50 μM) at 77K after 100 μs delay in counts (dashed line).

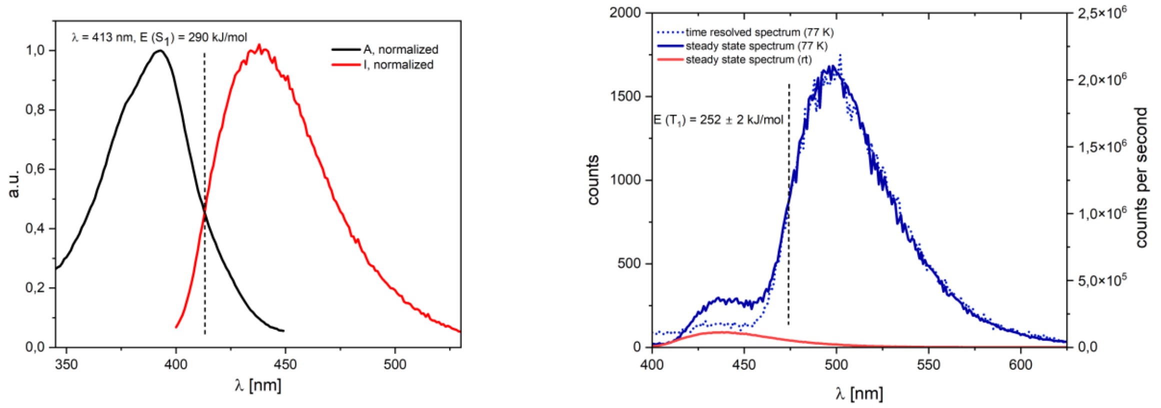

Figure S13.3. *Left:* UV/VIS spectrum of **9** in dichloromethane (c = 50 µM) normalized to the absorption maximum at 393 nm; luminescence of **9** in dichloromethane (c = 50 µM) at room temperature, normalized to the emission maximum at 438 nm. *Right:* Steady state spectra of **9** in dichloromethane (c = 50 µM) at room temperature and 77 K given in counts per second (solid lines), time resolved spectrum of **9** in dichloromethane (c = 50 µM) at 77 K after 50 µs delay in counts (dashed line).

14. Chiral HPLC Traces

Benzyl (1*R*,4*S*,4a*R*,9a*S*)-9-oxo-1,4,5,6,7,8,9,9a-octahydro-4a*H*-1,4-methanobenzo[7]annulene-4a-carboxylate (4a) and Benzyl (1*S*,4*R*,4a*R*,9a*S*)-9-oxo-1,4,5,6,7,8,9,9a-octahydro-4a*H*-1,4-methanobenzo[7]annulene-4a-carboxylate (4a')

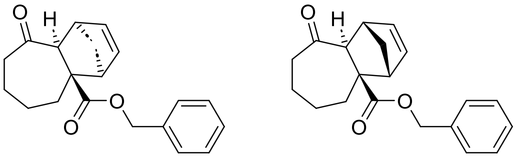

*Racemic Products rac-**4a** and rac-**4a'***

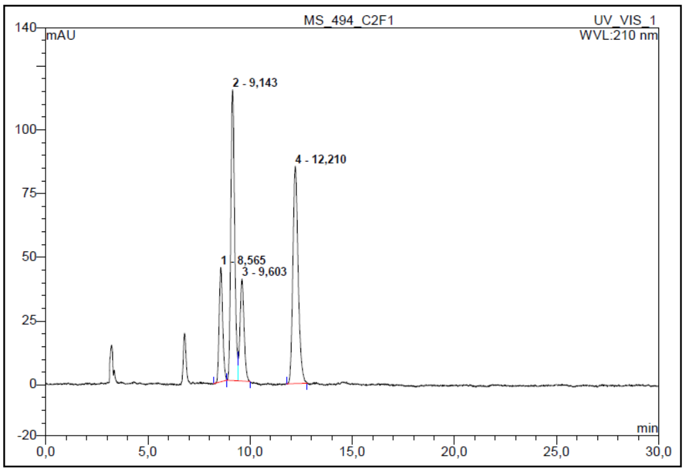

No.	Ret.Time min	Peak Name	Height mAU	Area mAU*min	Rel.Area %	Amount	Type
1	8,56	n.a.	44,793	8,856	12,89	n.a.	BMb*
2	9,14	n.a.	114,019	24,903	36,24	n.a.	bM *
3	9,60	n.a.	40,075	9,374	13,64	n.a.	MB*
4	12,21	n.a.	85,282	25,590	37,24	n.a.	BMB*
Total:			284,169	68,723	100,00	0,000	

*Enantioenriched Products **4a** and **4a'***

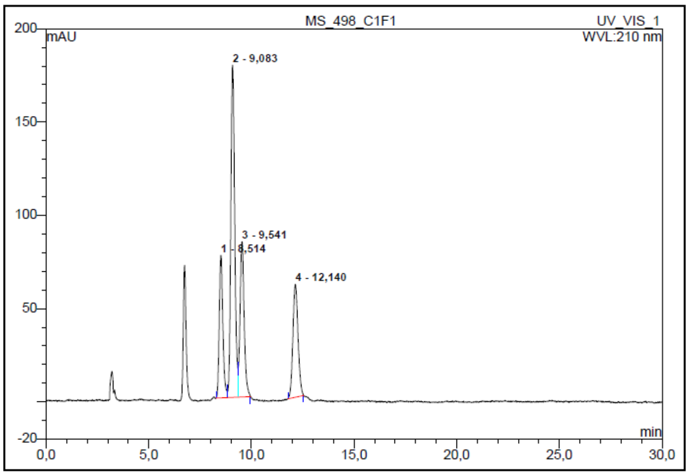

No.	Ret.Time min	Peak Name	Height mAU	Area mAU*min	Rel.Area %	Amount	Type
1	8,51	n.a.	76,393	15,535	16,95	n.a.	BM
2	9,08	n.a.	178,125	39,195	42,78	n.a.	M
3	9,54	n.a.	83,248	19,414	21,19	n.a.	MB
4	12,14	n.a.	60,449	17,486	19,08	n.a.	BMB
Total:			398,214	91,629	100,00	0,000	

Benzyl (1*R*,4*S*,4a*S*,9a*R*)-9-oxo-1,4,5,6,7,8,9,9a-octahydro-4a*H*-1,4-epoxybenzo[7]annulene-4a-carboxylate (4d) and Benzyl (1*S*,4*R*,4a*S*,9a*R*)-9-oxo-1,4,5,6,7,8,9,9a-octahydro-4a*H*-1,4-epoxybenzo[7]annulene-4a-carboxylate (4d')

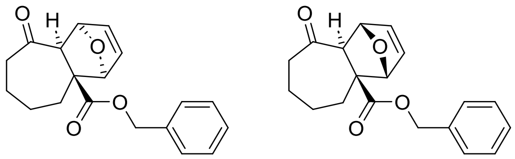

Racemic Product rac-**4d**

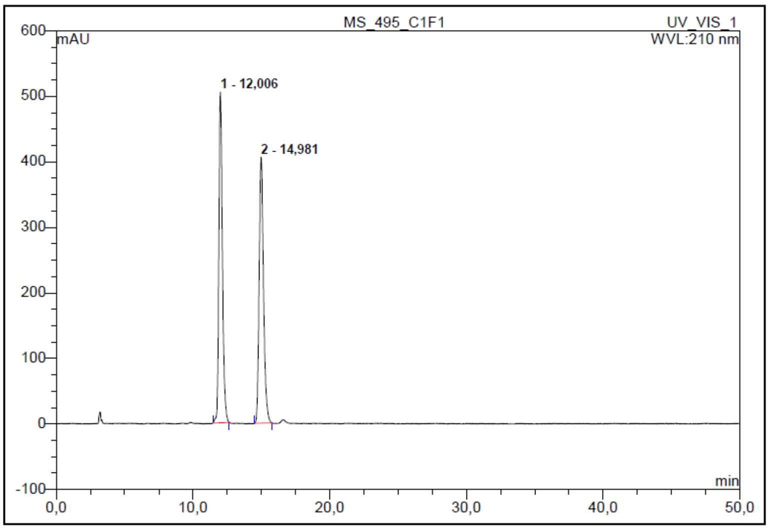

No.	Ret.Time min	Peak Name	Height mAU	Area mAU*min	Rel.Area %	Amount	Type
1	12,01	n.a.	504,644	152,464	50,27	n.a.	BMB
2	14,98	n.a.	405,595	150,801	49,73	n.a.	BMB
Total:			910,239	303,265	100,00	0,000	

Racemic Product rac-**4d'**

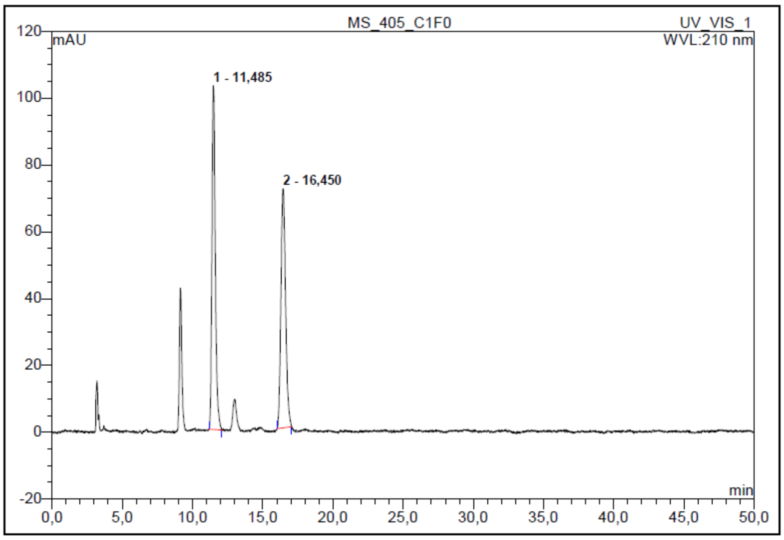

No.	Ret.Time min	Peak Name	Height mAU	Area mAU*min	Rel.Area %	Amount	Type
1	11,49	n.a.	102,903	28,459	50,95	n.a.	BMB
2	16,45	n.a.	71,577	27,403	49,05	n.a.	BMB*
Total:			174,480	55,862	100,00	0,000	

Enantioenriched Product **4d**

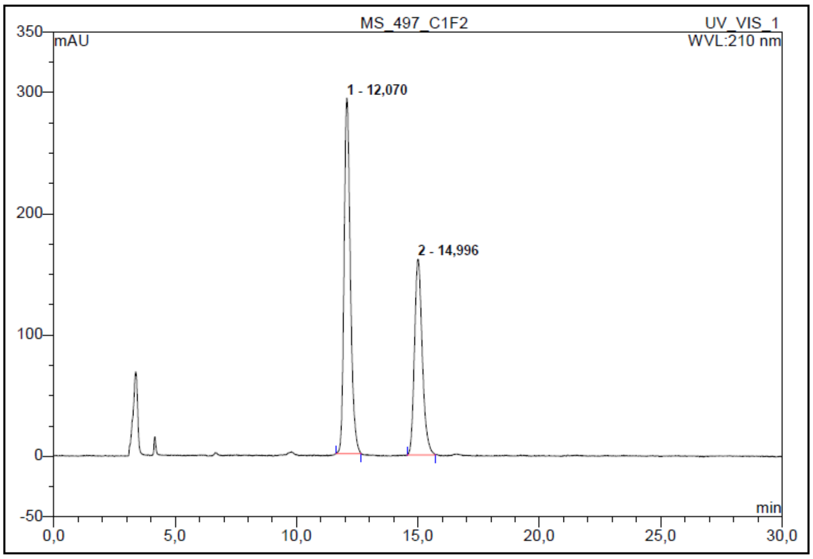

No.	Ret.Time min	Peak Name	Height mAU	Area mAU*min	Rel.Area %	Amount	Type
1	12,07	n.a.	293,402	87,704	59,41	n.a.	BMB
2	15,00	n.a.	161,433	59,911	40,59	n.a.	BMB
Total:			454,834	147,615	100,00	0,000	

Enantioenriched Product **4d'**

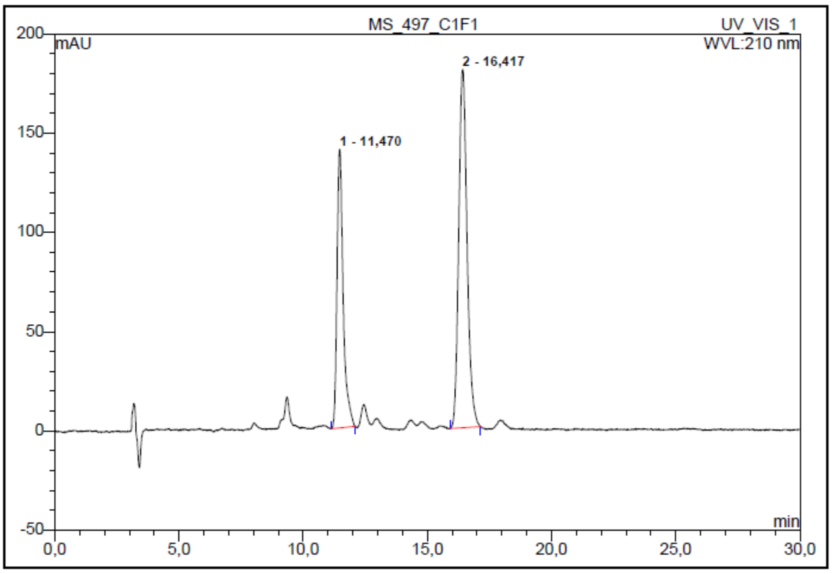

No.	Ret.Time min	Peak Name	Height mAU	Area mAU*min	Rel.Area %	Amount	Type
1	11,47	n.a.	140,337	40,298	36,46	n.a.	BMB
2	16,42	n.a.	180,454	70,227	63,54	n.a.	BMB
Total:			320,791	110,525	100,00	0,000	

Benzyl (1*R*,4*S*,4a*S*,9a*R*)-1,4-dimethyl-9-oxo-1,4,5,6,7,8,9,9a-octahydro-4a*H*-1,4-epoxybenzo[7]annulene-4a-carboxylate (4e)

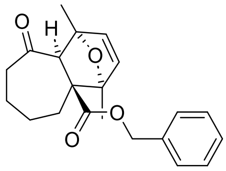

Racemic Product rac-**4e**

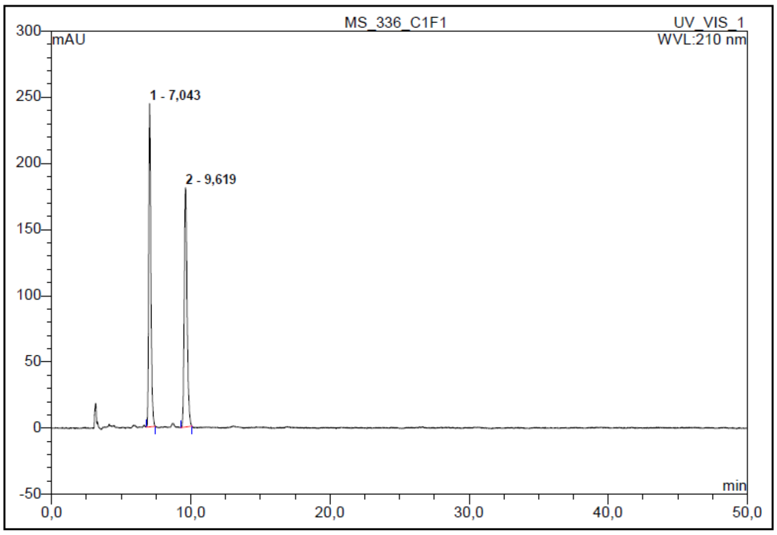

No.	Ret.Time min	Peak Name	Height mAU	Area mAU*min	Rel.Area %	Amount	Type
1	7,04	n.a.	244,231	43,548	50,15	n.a.	BMB
2	9,62	n.a.	180,825	43,287	49,85	n.a.	BMB
Total:			425,056	86,835	100,00	0,000	

Enantioenriched Product **4e** (Catalyst **7**)

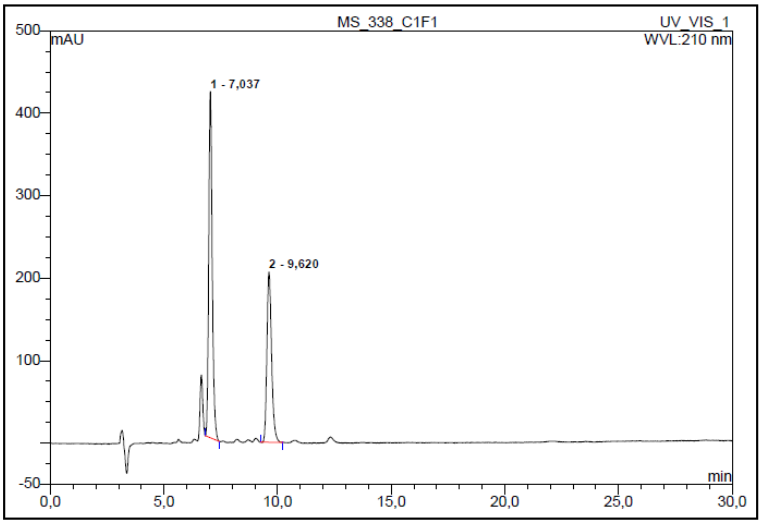

No.	Ret.Time min	Peak Name	Height mAU	Area mAU*min	Rel.Area %	Amount	Type
1	7,04	n.a.	419,933	75,945	60,61	n.a.	BMB*
2	9,62	n.a.	206,513	49,353	39,39	n.a.	BMB*
Total:			626,446	125,299	100,00	0,000	

Enantioenriched Product **4e** (Catalyst **8a**)

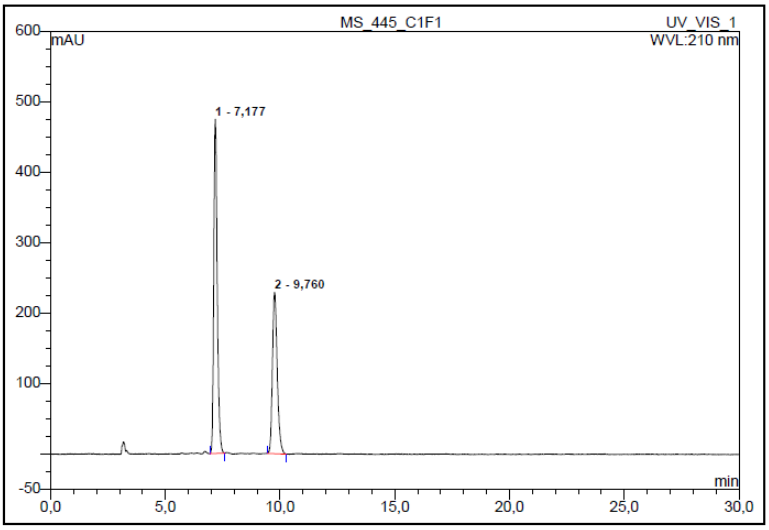

No.	Ret.Time min	Peak Name	Height mAU	Area mAU*min	Rel.Area %	Amount	Type
1	7,18	n.a.	474,062	83,113	60,05	n.a.	BMB
2	9,76	n.a.	228,734	55,293	39,95	n.a.	BMB
Total:			702,796	138,407	100,00	0,000	

Enantioenriched Product **4e** (Catalyst **8b**)

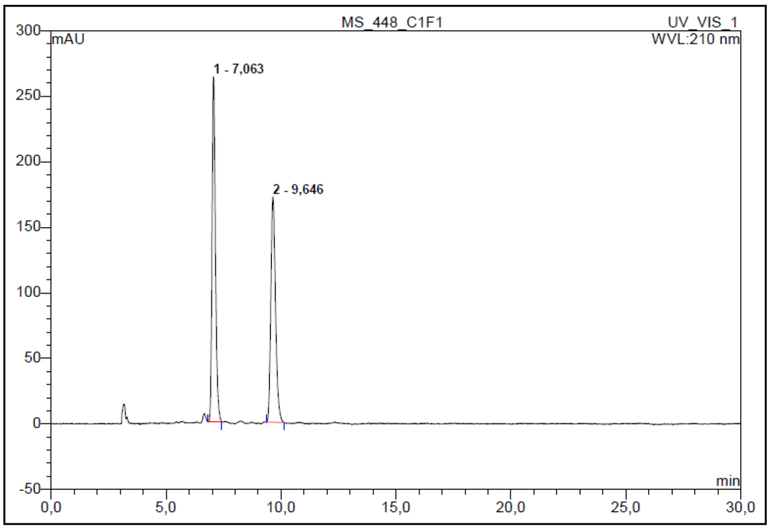

No.	Ret.Time min	Peak Name	Height mAU	Area mAU*min	Rel.Area %	Amount	Type
1	7,06	n.a.	263,342	45,067	52,60	n.a.	BMB
2	9,65	n.a.	171,962	40,616	47,40	n.a.	BMB
Total:			435,304	85,683	100,00	0,000	

Enantioenriched product **4e** (Catalyst **8c**)

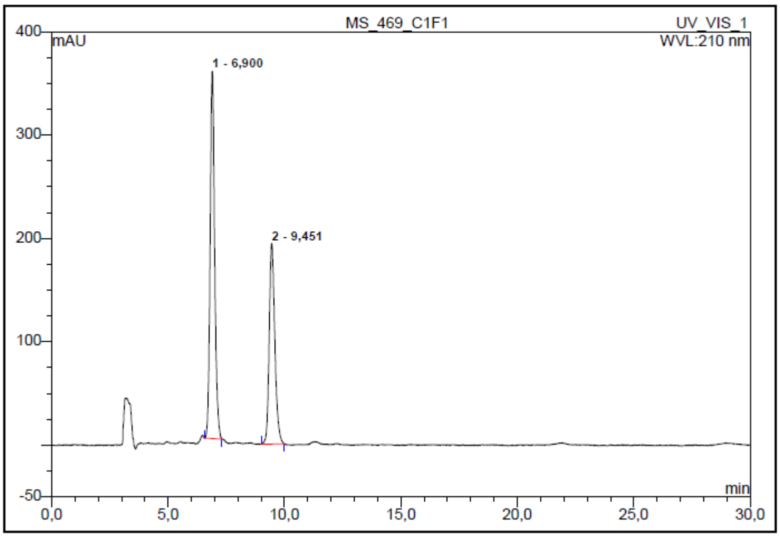

No.	Ret.Time min	Peak Name	Height mAU	Area mAU*min	Rel.Area %	Amount	Type
1	6,90	n.a.	355,599	79,748	59,50	n.a.	BMB
2	9,45	n.a.	194,179	54,282	40,50	n.a.	BMB
Total:			549,779	134,030	100,00	0,000	

Enantioenriched product **4e** (Catalyst **9**)

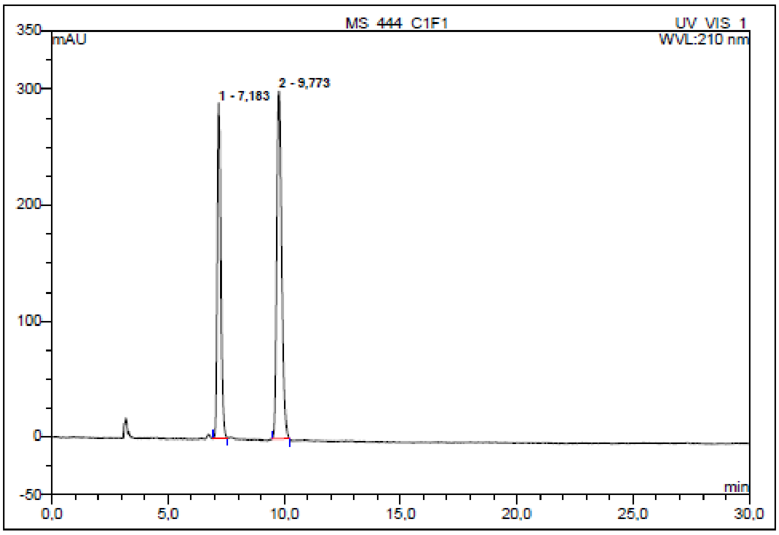

No.	Ret.Time min	Peak Name	Height mAU	Area mAU*min	Rel.Area %	Amount	Type
1	7,18	n.a.	288,810	50,267	41,05	n.a.	BMB
2	9,77	n.a.	299,543	72,194	58,95	n.a.	BMB
Total:			588,353	122,461	100,00	0,000	

Benzyl (1*R*,4*S*,4a*S*,9a*R*)-1,4-dibutyl-9-oxo-1,4,5,6,7,8,9,9a-octahydro-4a*H*-1,4-epoxybenzo[7]annulene-4a-carboxylate (4h)

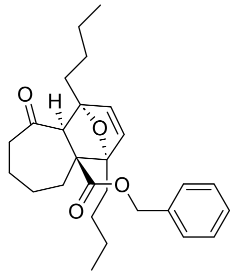

Racemic product rac-**4h**

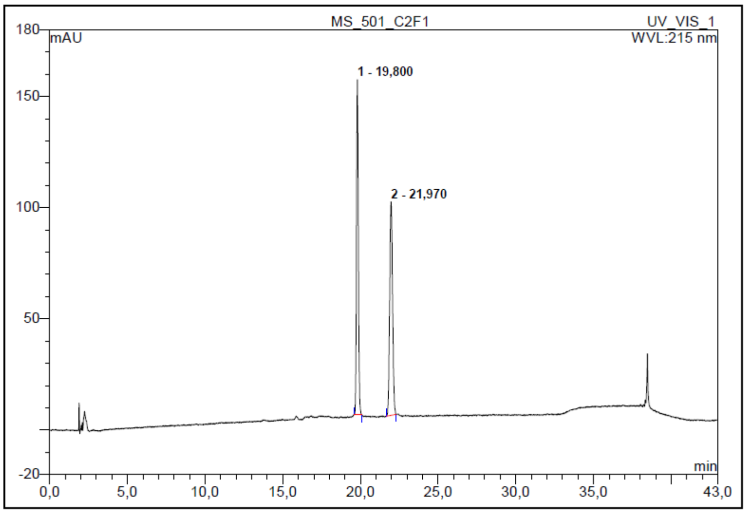

No.	Ret.Time min	Peak Name	Height mAU	Area mAU*min	Rel.Area %	Amount	Type
1	19,80	n.a.	150,713	20,863	50,01	n.a.	BMB
2	21,97	n.a.	96,082	20,858	49,99	n.a.	BMB
Total:			246,795	41,721	100,00	0,000	

Enantioenriched product **4h'**

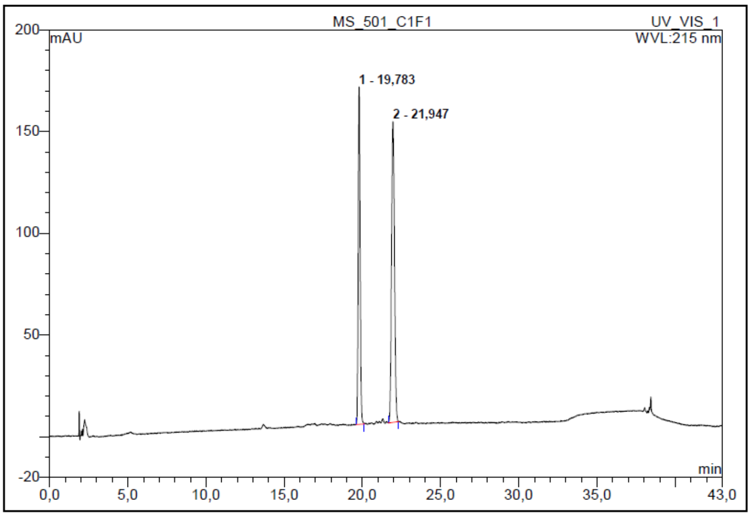

No.	Ret.Time min	Peak Name	Height mAU	Area mAU*min	Rel.Area %	Amount	Type
1	19,78	n.a.	165,888	23,158	41,77	n.a.	BMB
2	21,95	n.a.	147,906	32,281	58,23	n.a.	BMB
Total:			313,794	55,439	100,00	0,000	

Benzyl (4a*R*,9a*S*)-2,3-dimethyl-9-oxo-1,4,5,6,7,8,9,9a-octahydro-4a*H*-benzo[7]annulene-4a-carboxylate (4m)

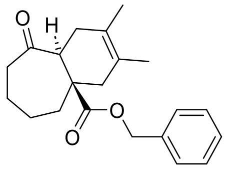

Racemic product rac-**4m**

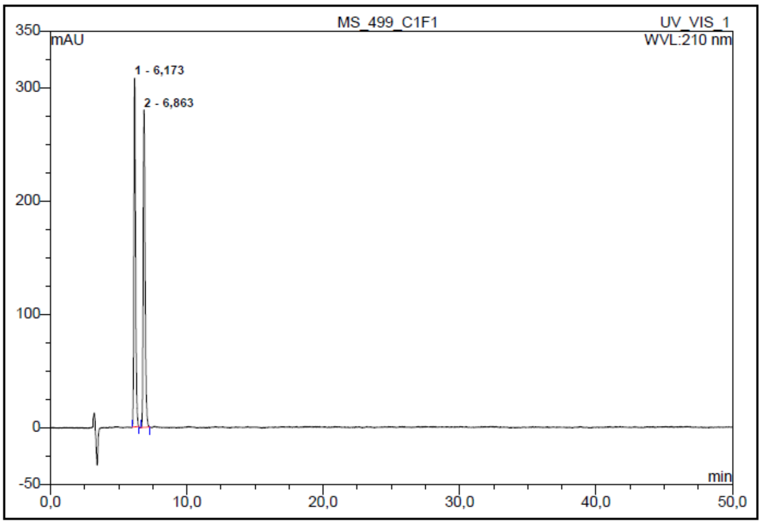

No.	Ret.Time min	Peak Name	Height mAU	Area mAU*min	Rel.Area %	Amount	Type
1	6,17	n.a.	307,543	44,065	49,56	n.a.	BMB
2	6,86	n.a.	279,940	44,851	50,44	n.a.	BMB
Total:			587,482	88,916	100,00	0,000	

Enantioenriched product **4m**

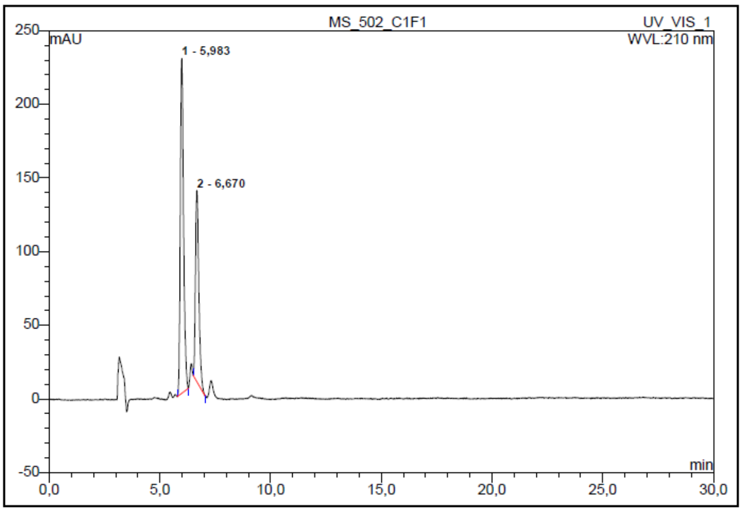

No.	Ret.Time min	Peak Name	Height mAU	Area mAU*min	Rel.Area %	Amount	Type
1	5,98	n.a.	227,221	38,744	62,72	n.a.	BMB
2	6,67	n.a.	129,803	23,033	37,28	n.a.	BMB
Total:			357,024	61,777	100,00	0,000	

4-Bromobenzyl (1*R*,4*S*,4a*S*,9a*R*)-1,4-dimethyl-9-oxo-1,4,5,6,7,8,9,9a-octahydro-4a*H*-1,4-epoxybenzo[7]annulene-4a-carboxylate (5)

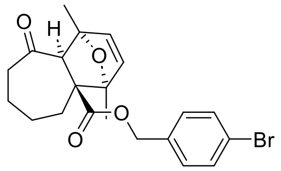

Racemic product rac-**5**

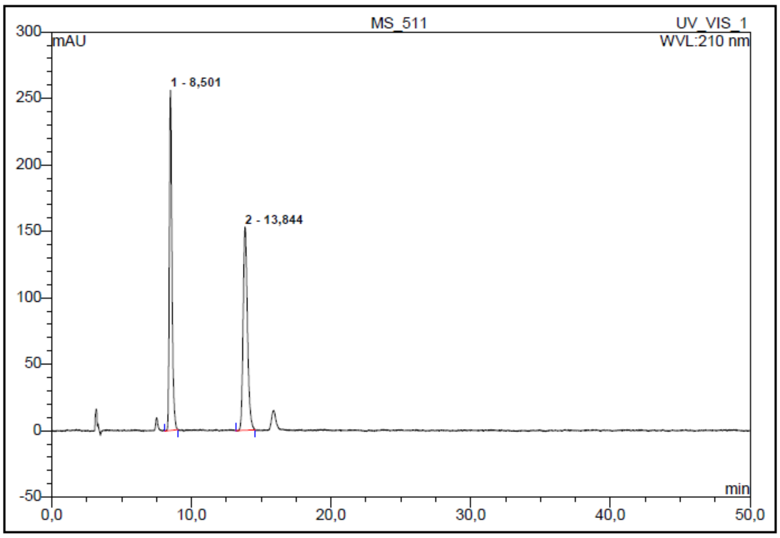

No.	Ret.Time min	Peak Name	Height mAU	Area mAU*min	Rel.Area %	Amount	Type
1	8,50	n.a.	256,033	55,626	50,32	n.a.	BMB*
2	13,84	n.a.	152,921	54,922	49,68	n.a.	BMB*
Total:			408,954	110,548	100,00	0,000	

Enantioenriched product **5**

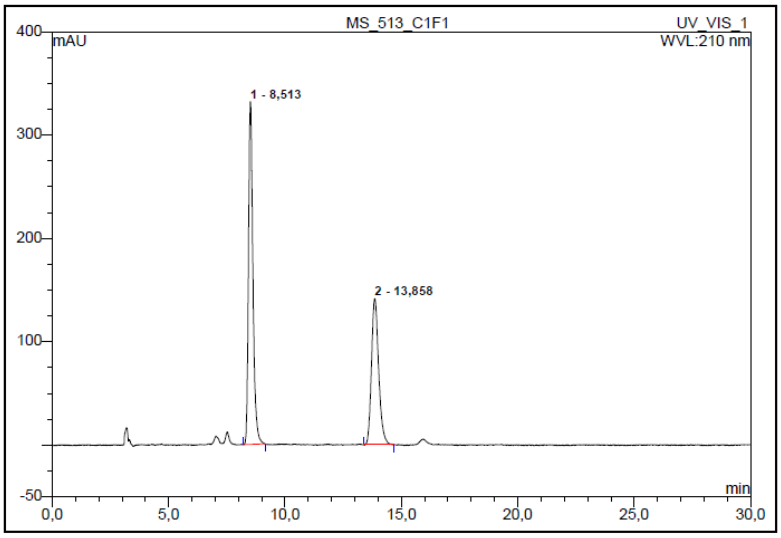

No.	Ret.Time min	Peak Name	Height mAU	Area mAU*min	Rel.Area %	Amount	Type
1	8,51	n.a.	331,906	73,219	59,19	n.a.	BMB*
2	13,86	n.a.	141,402	50,487	40,81	n.a.	BMB*
Total:			473,308	123,706	100,00	0,000	

15. NMR Spectra of New Compounds

15.1 NMR Spectra of Substrates

Methyl 3-oxocyclohept-1-ene-1-carboxylate (S2)

^{1}H NMR (500 MHz, $CDCl_3$):

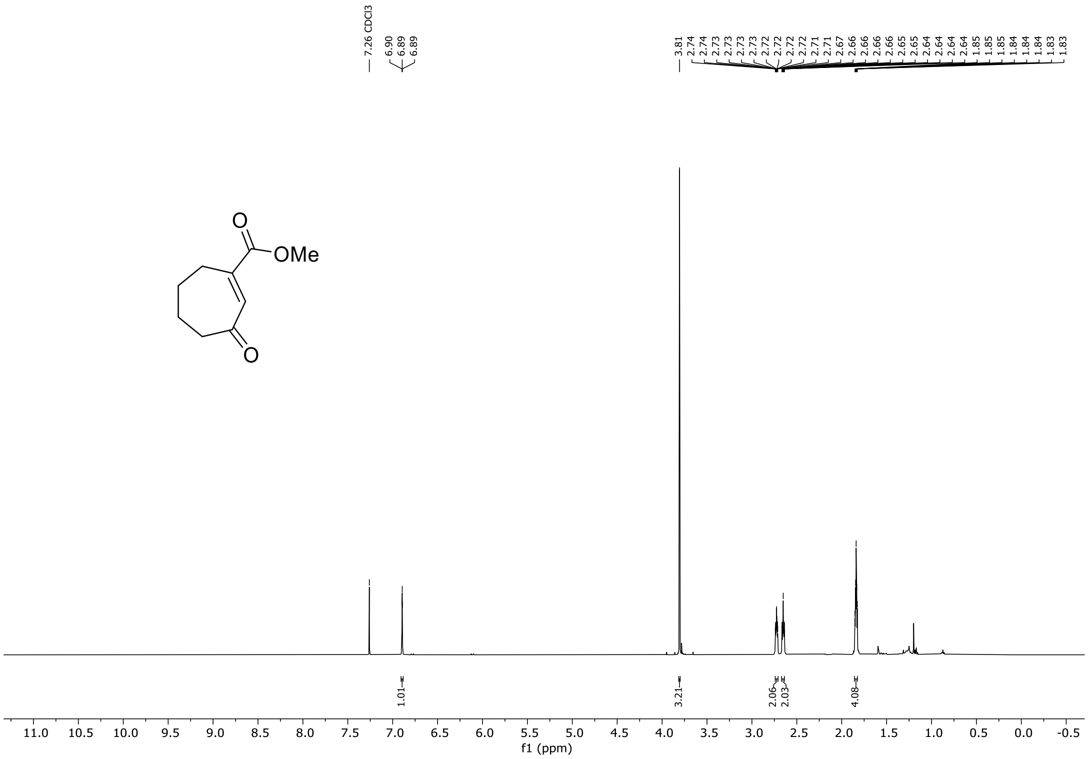

^{13}C NMR (126 MHz, $CDCl_3$):

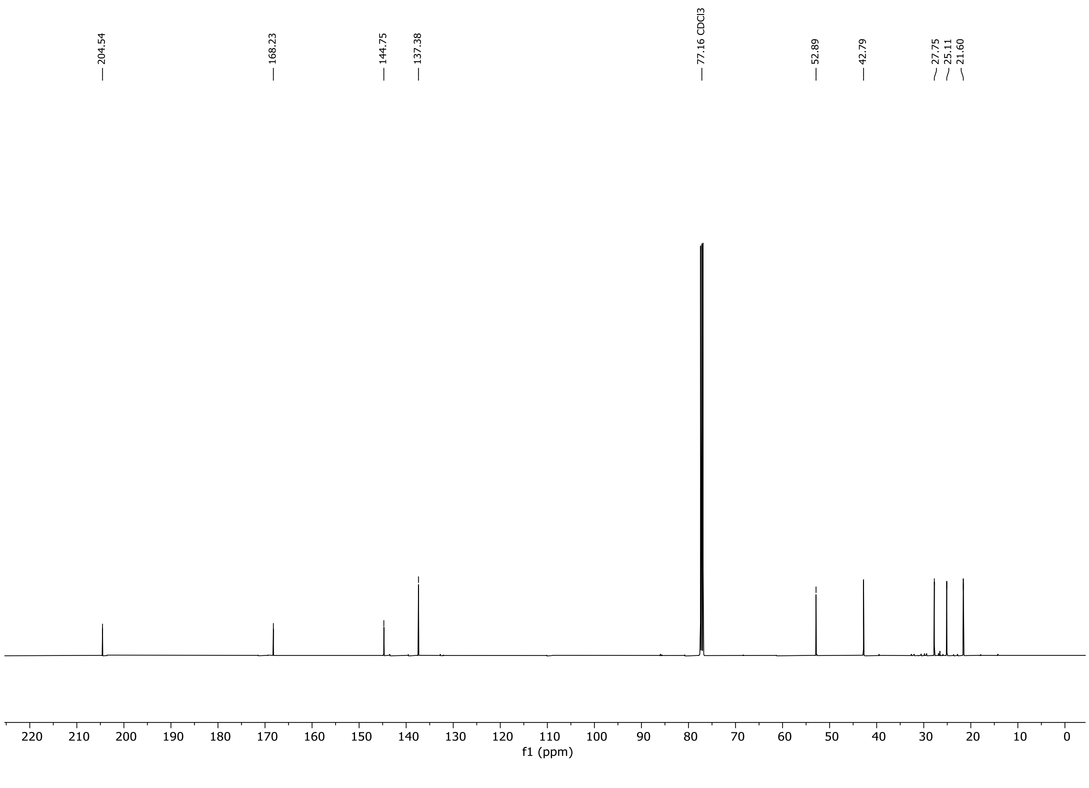

3-Oxocyclohept-1-ene-1-carboxylic acid (1)

¹H NMR (500 MHz, $CDCl_3$):

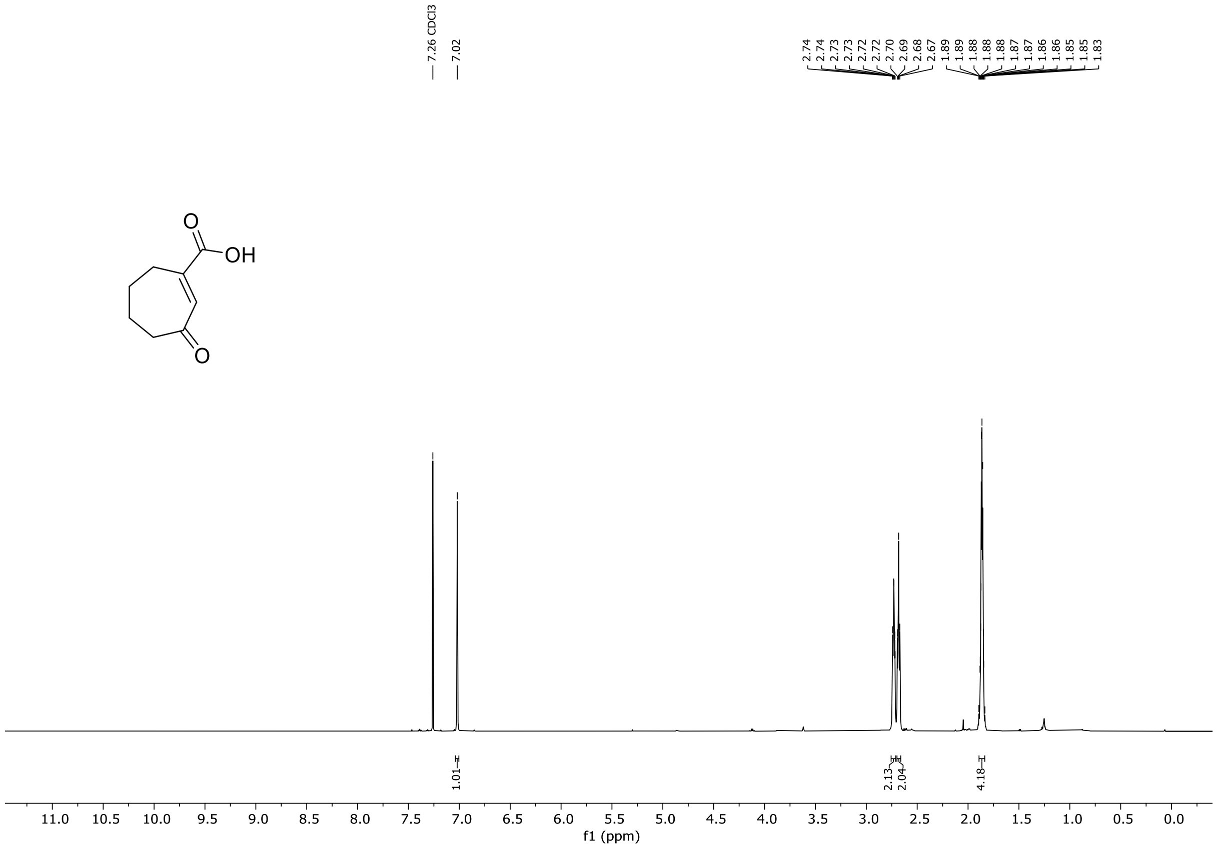

¹³C NMR (126 MHz, $CDCl_3$):

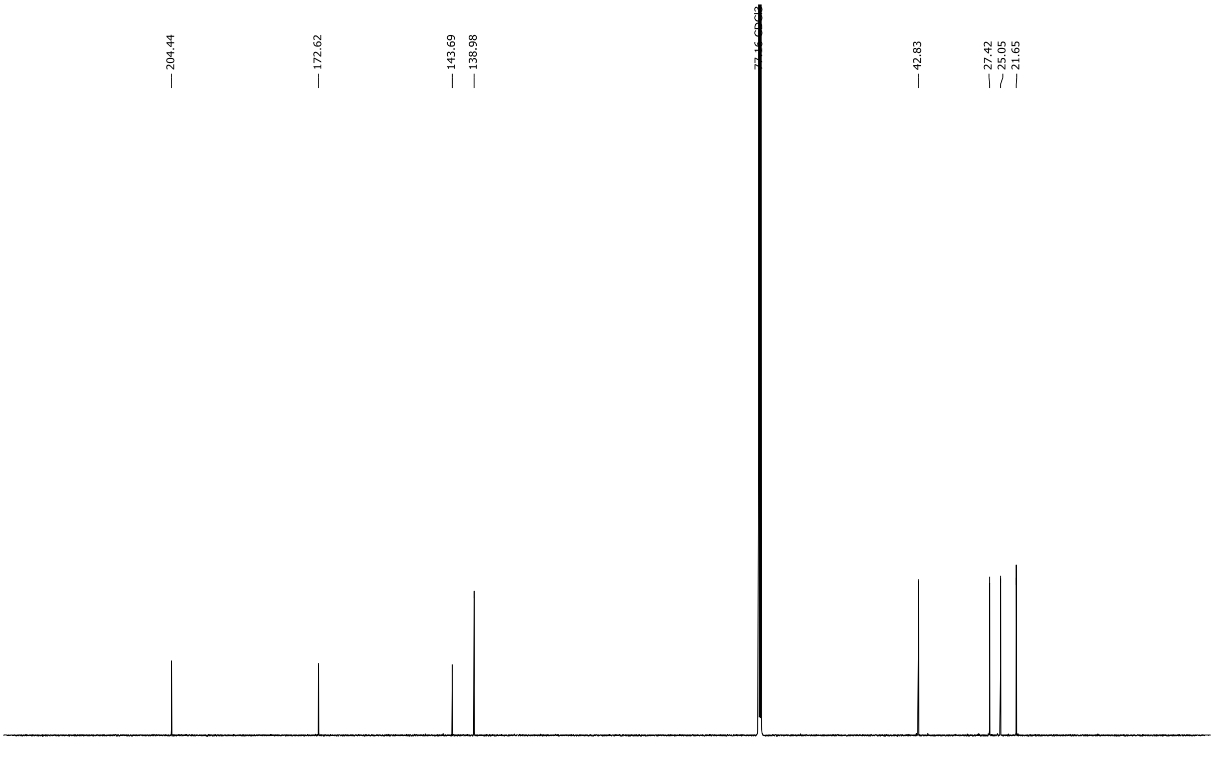

Benzyl 3-oxocyclohept-1-ene-1-carboxylate (S3)

^{1}H NMR (300 MHz, $CDCl_3$):

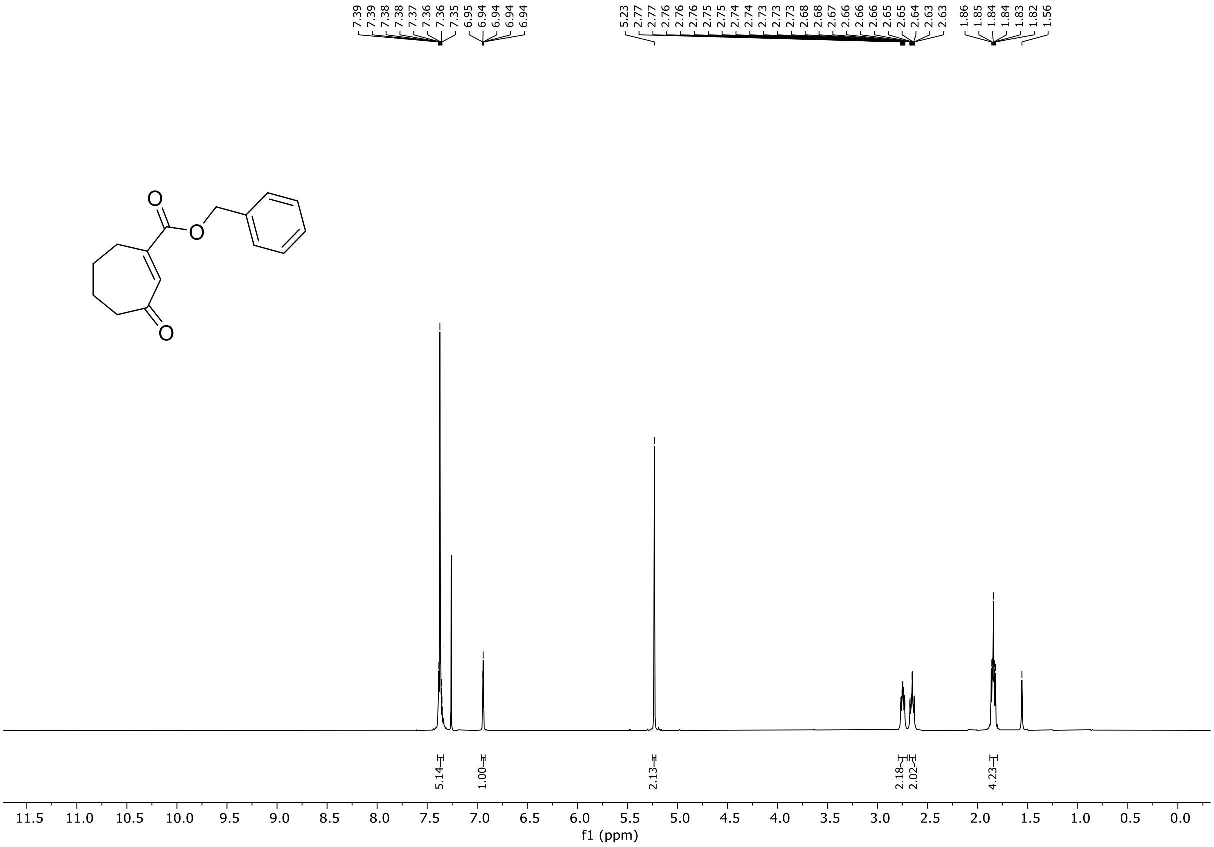

^{13}C NMR (101 MHz, $CDCl_3$):

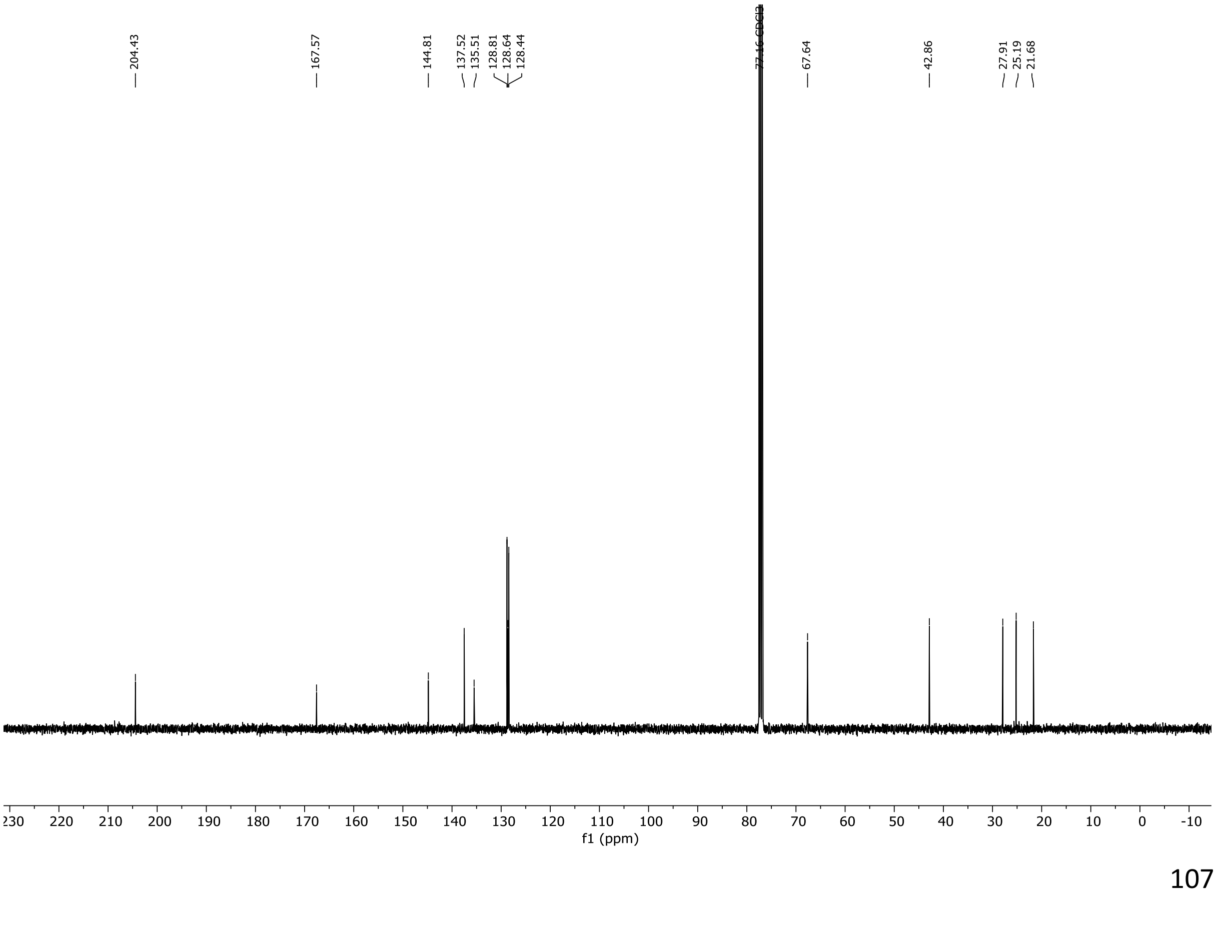

15.2 Synthesis of Phosphoric Acid Catalysts

2-(3-(4,4,5,5-Tetramethyl-1,3,2-dioxaborolan-2-yl)phenyl)-9*H*-thioxanthen-9-one (S5)

^{1}H NMR (400 MHz, $CDCl_3$):

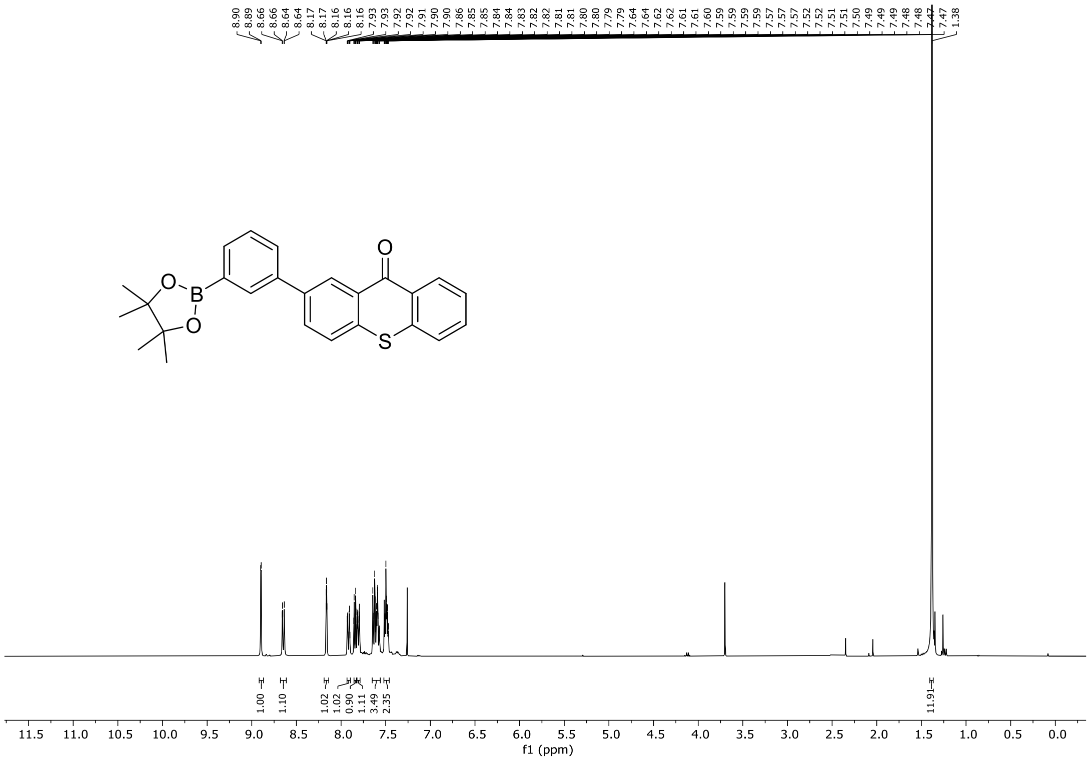

^{13}C NMR (101 MHz, $CDCl_3$):

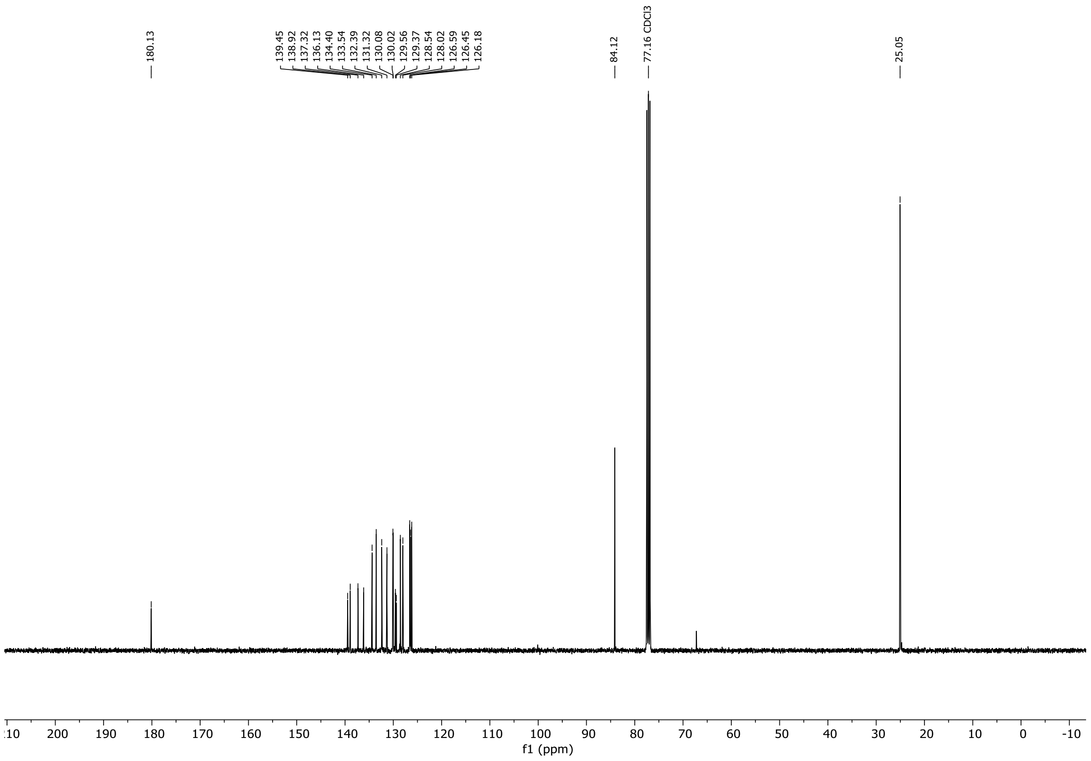

(*R*)-2,2'-(2,2'-Dihydroxy-5,5',6,6',7,7',8,8'-octahydro-[1,1'-binaphthalene]-3,3'-diyl)bis(9*H*-thioxanthen-9-one) (S6)

^{1}H NMR (400 MHz, $CDCl_3$):

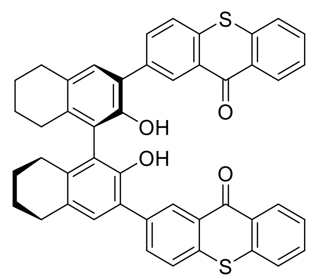

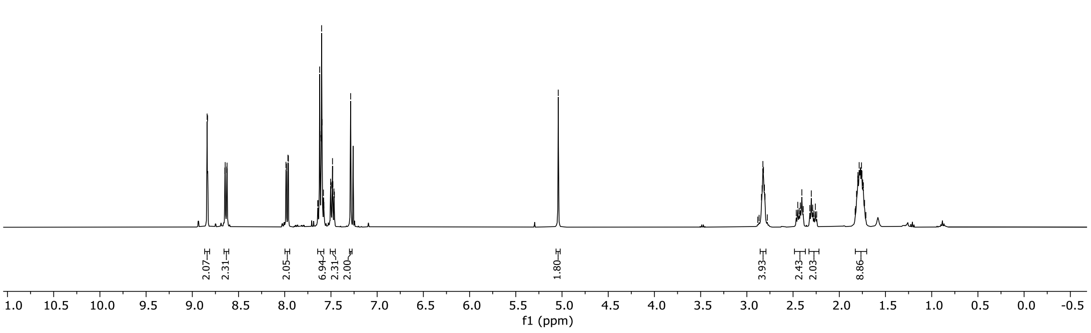

^{13}C NMR (101 MHz, $CDCl_3$):

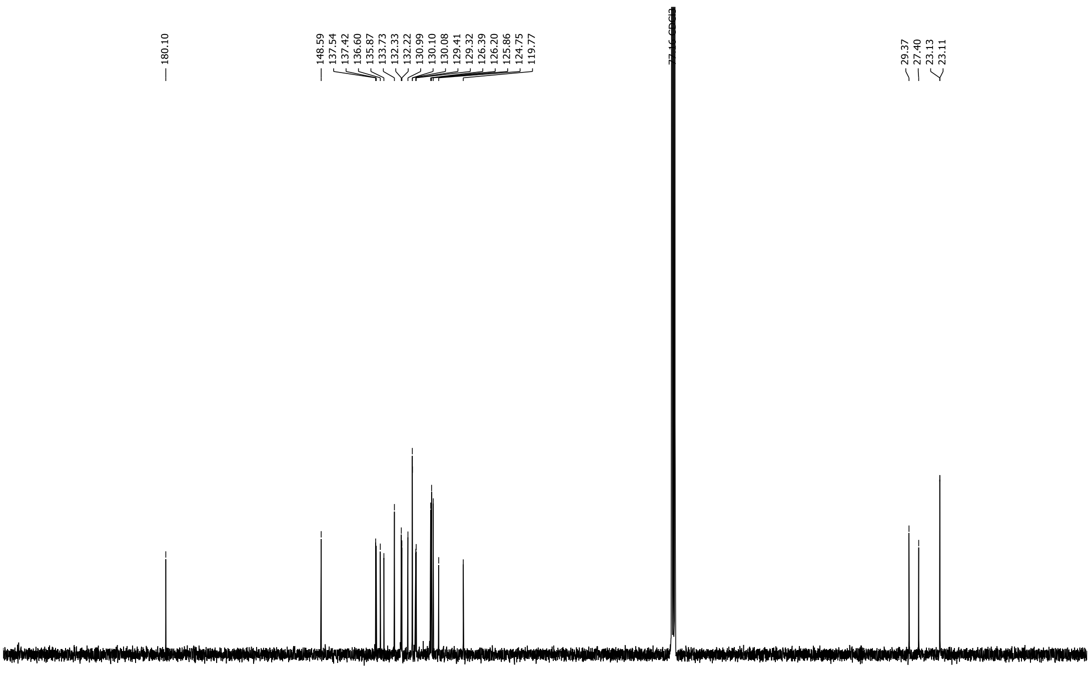

2,2'-((11*bR*)-4-Hydroxy-4-oxido-8,9,10,11,12,13,14,15-octahydrodinaphtho[2,1-*d*:1',2'-*f*][1,3,2]dioxaphosphepine-2,6-diyl)bis(9*H*-thioxanthen-9-one) (8b)

^{1}H NMR (400 MHz, DMSO-d_6):

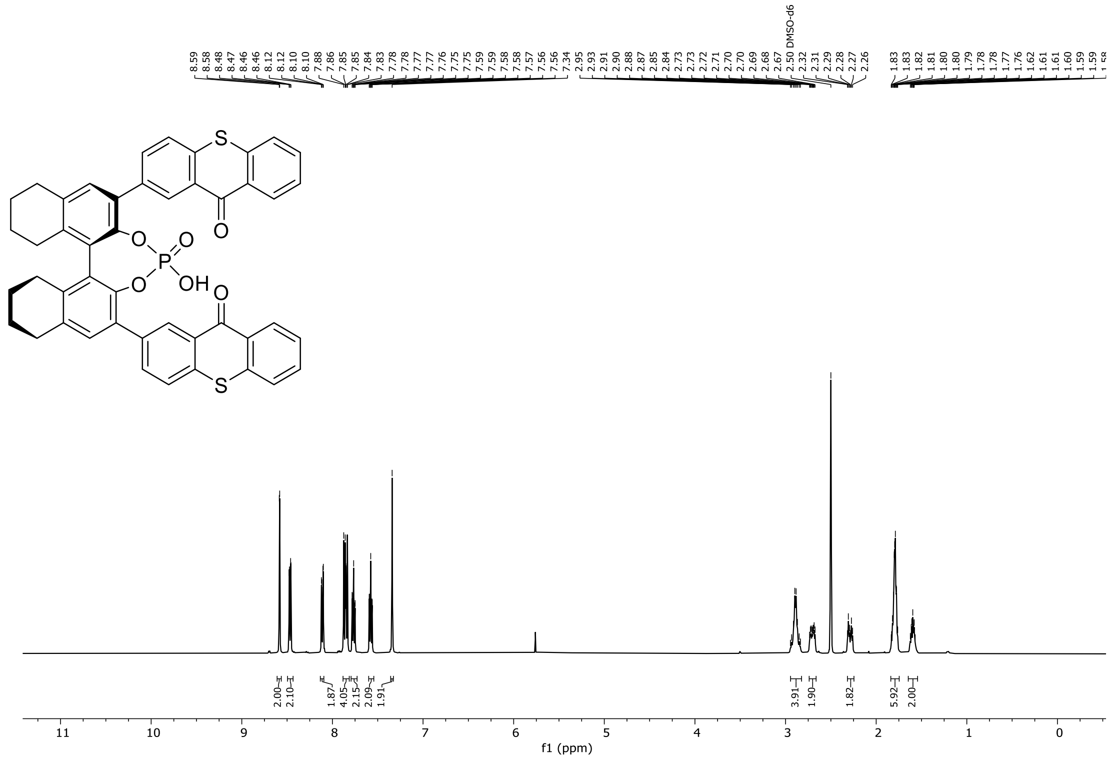

^{13}C NMR (126 MHz, DMSO-d_6):

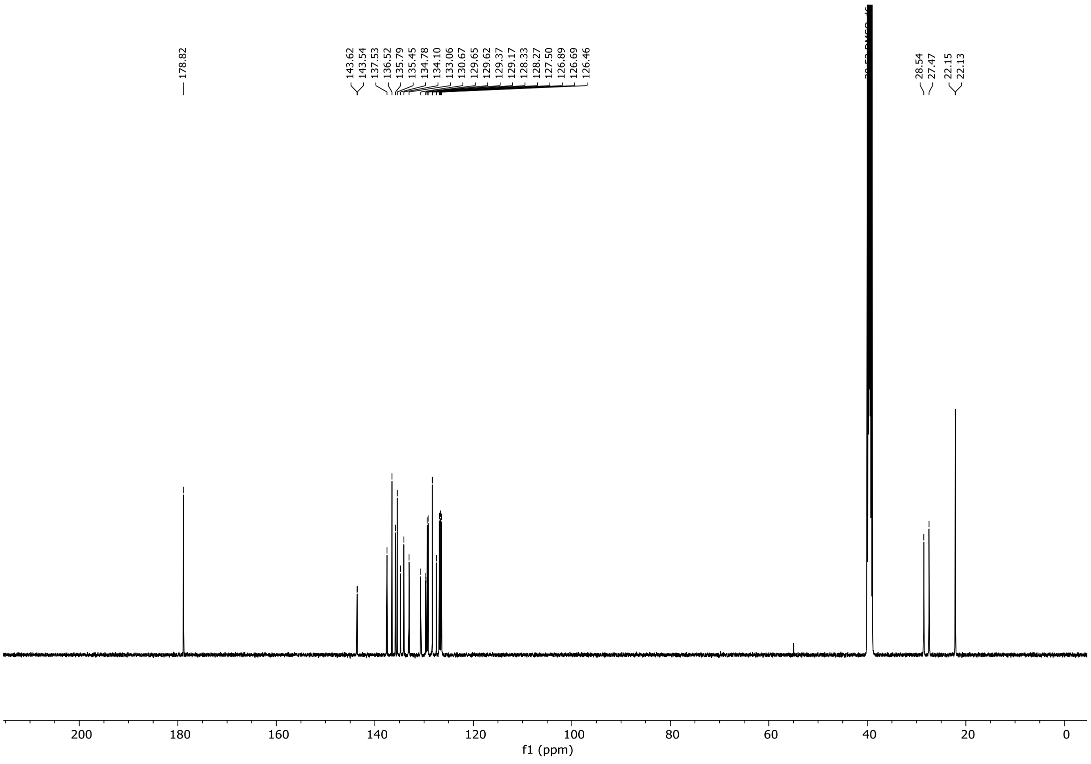

^{31}**P NMR** (162 MHz, DMSO-d_6):

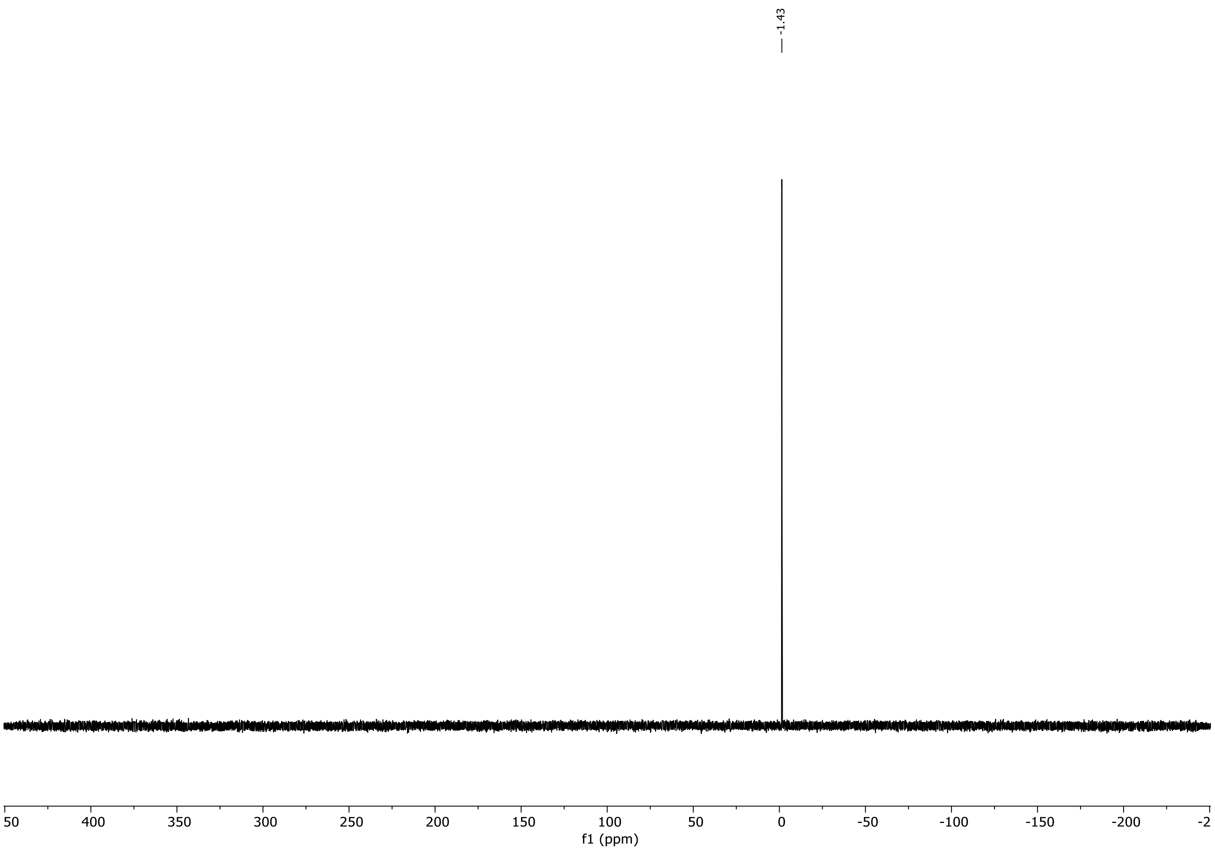

(*R*)-2,2'-((2,2'-Dihydroxy-5,5',6,6',7,7',8,8'-octahydro-[1,1'-binaphthalene]-3,3'-diyl)bis(3,1-phenylene))bis(9*H*-thioxanthen-9-one) (S7)

^{1}**H NMR** (400 MHz, CDCl$_3$):

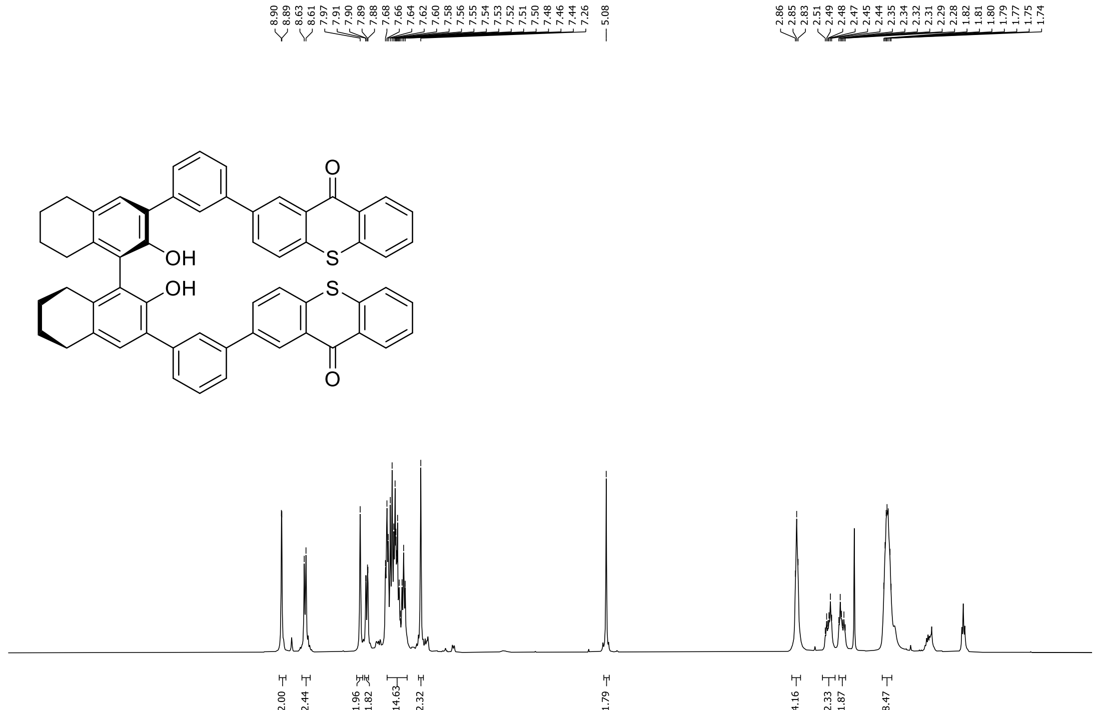

^{13}C NMR (101MHz, $CDCl_3$):

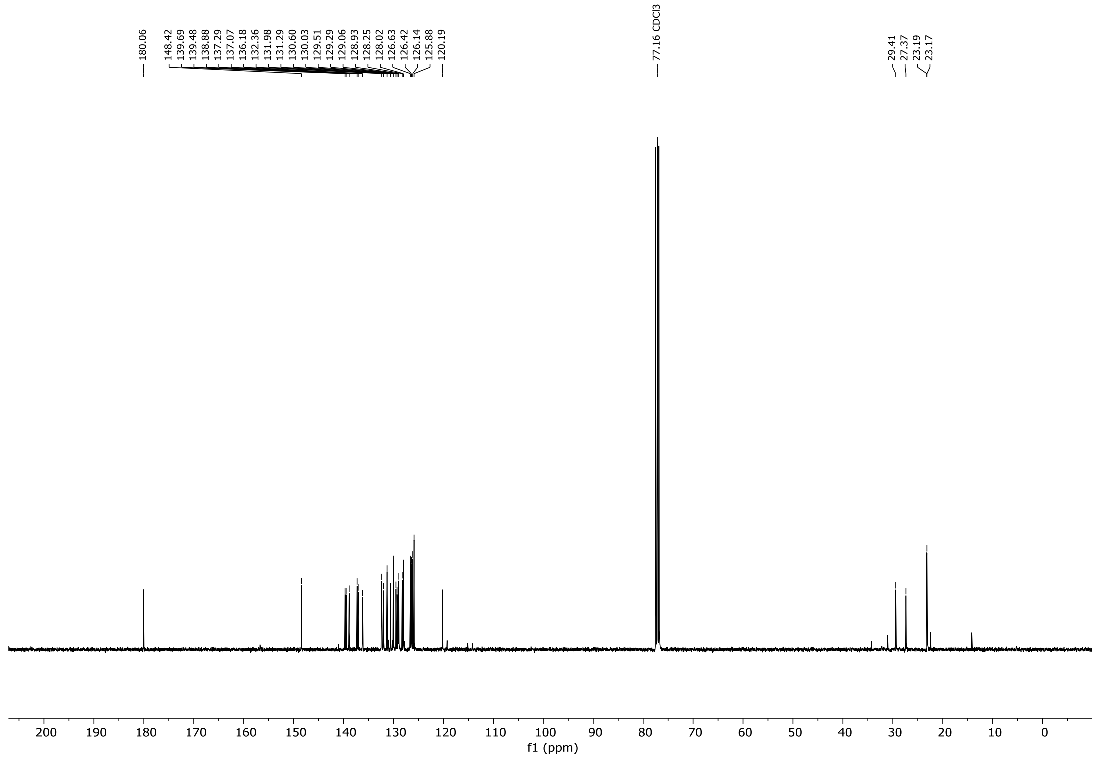

2,2'-(((11*bR*)-4-Hydroxy-4-oxido-8,9,10,11,12,13,14,15-octahydrodinaphtho[2,1-*d*:1',2'-*f*][1,3,2]dioxaphosphepine-2,6-diyl)bis(3,1-phenylene))bis(9*H*-thioxanthen-9-one) (8a)

^{1}H NMR (400 MHz, DMSO-d_6):

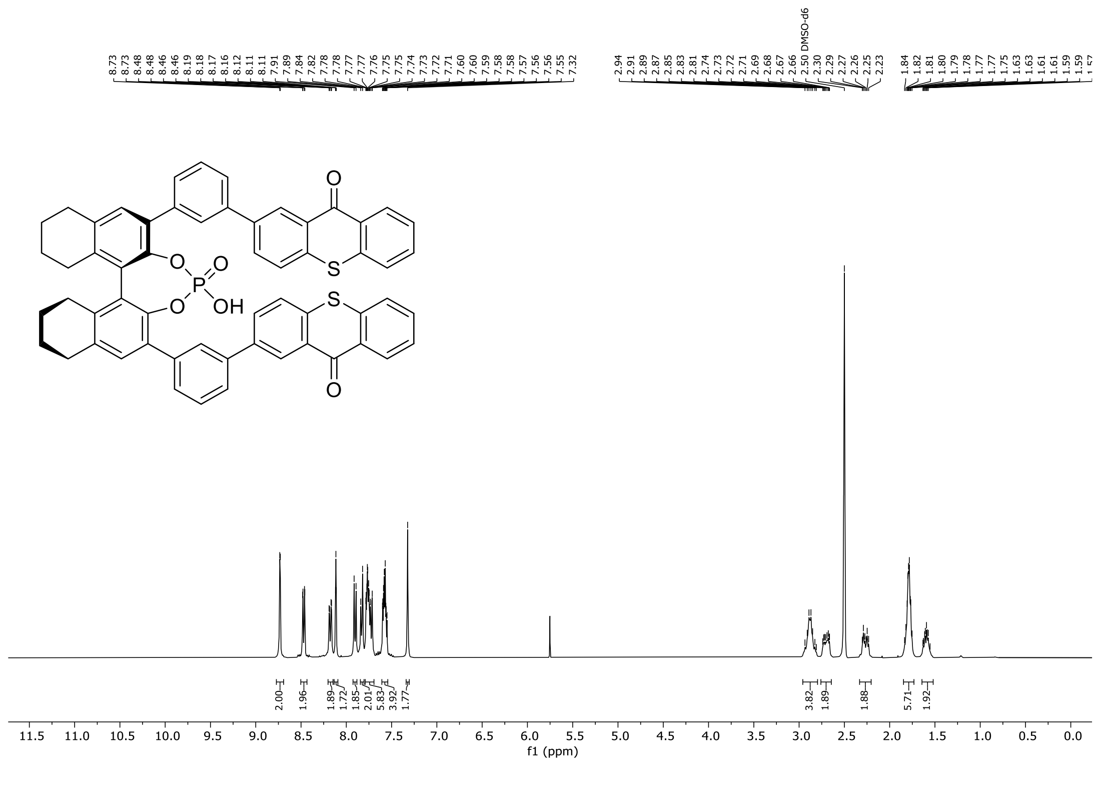

^{13}**C NMR** (126 MHz, DMSO-d_6):

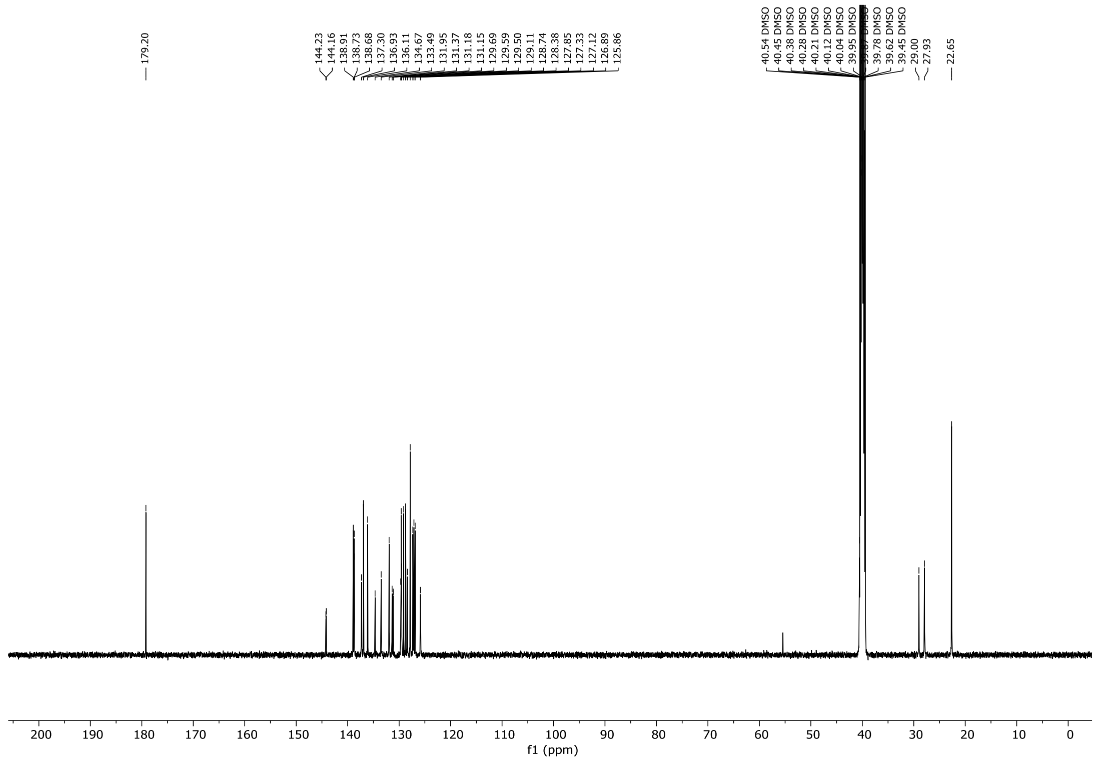

^{31}**P NMR** (162 MHz, DMSO-d_6):

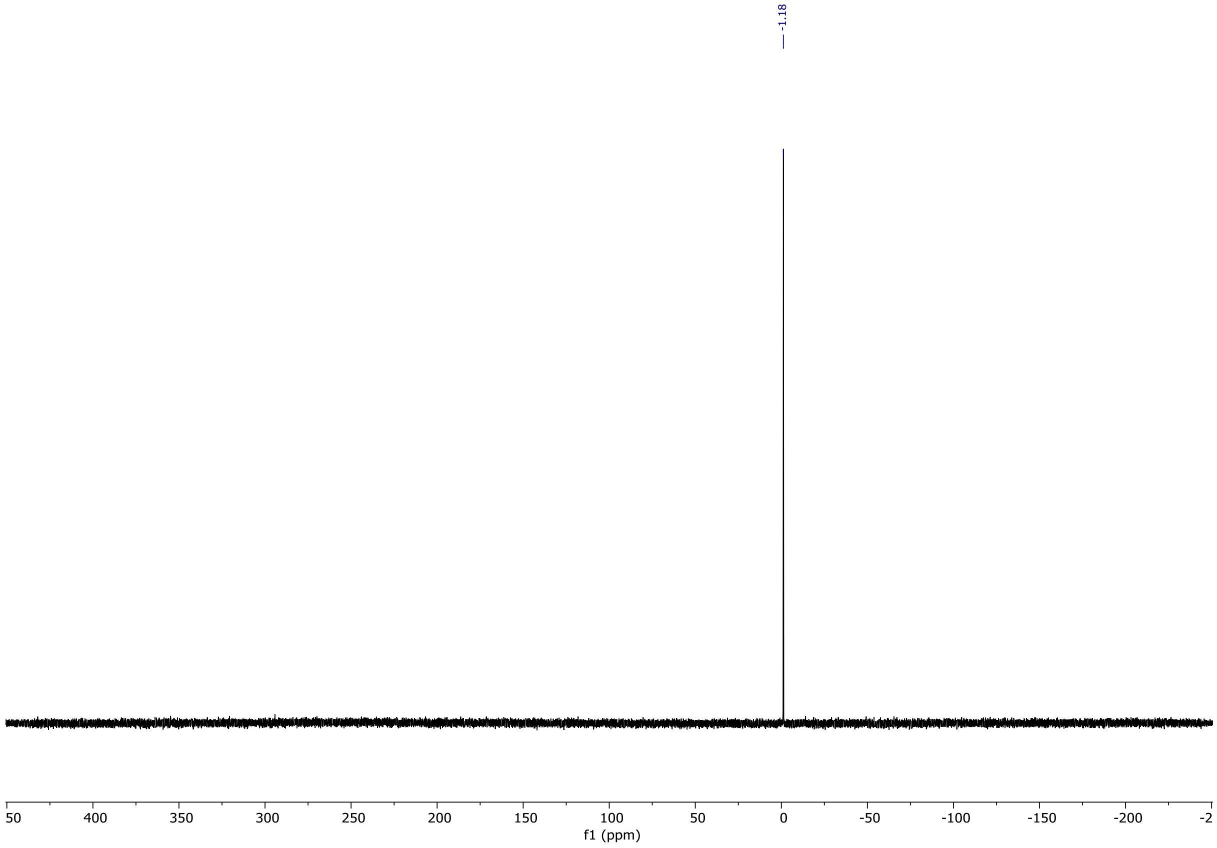

3-(3-((11*cR*)-6-(3,5-Di-tert-butylphenyl)-4-hydroxy-4-oxido-8,9,10,11,12,13,14,15-octahydrodinaphtho[2,1-*d*:1',2'-*f*][1,3,2]dioxaphosphepin-2-yl)phenyl)-9*H*-thioxanthen-9-one (8c)

^{1}H NMR (500 MHz, DMSO-d_6):

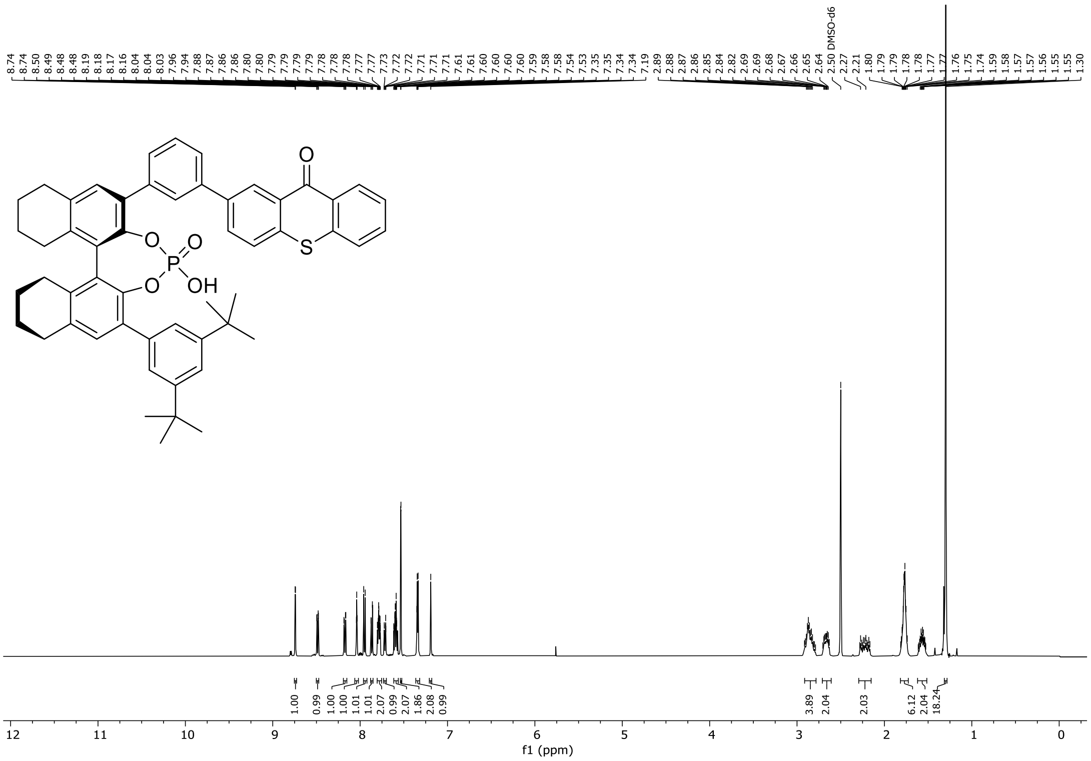

^{13}C NMR (126 MHz, DMSO-d_6):

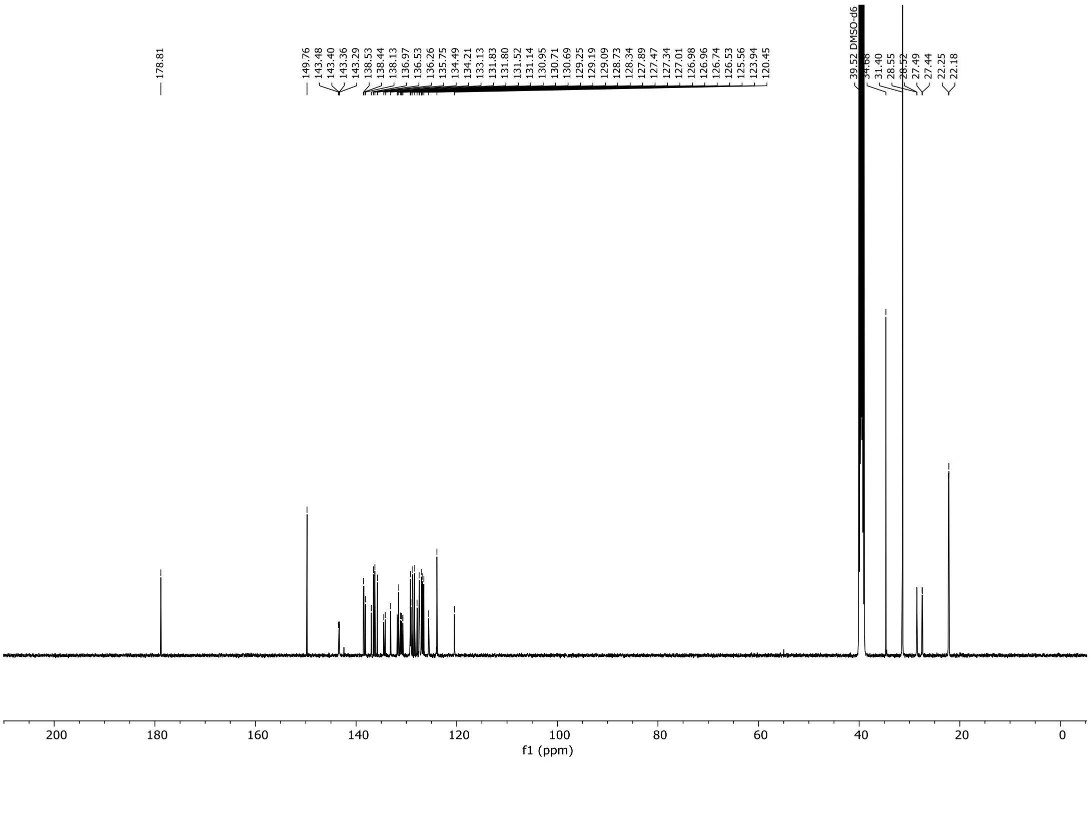

^{31}**P NMR** (162 MHz, DMSO-*d*$_6$):

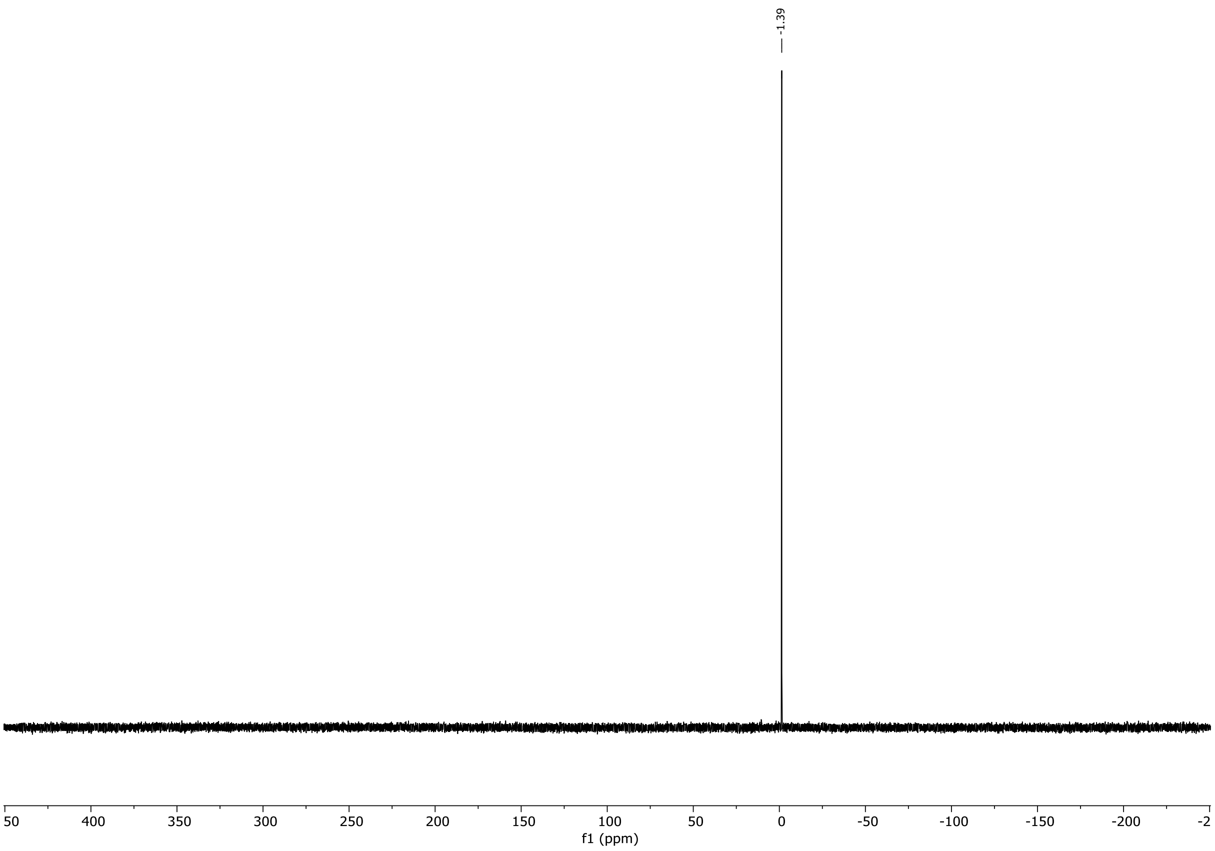

2,2'-((7,7'-Bis(methoxymethoxy)-2,2',3,3'-tetrahydro-1,1'-spirobi[indene]-6,6'-diyl)bis(3,1-phenylene))bis(9*H*-thioxanthen-9-one) (S8)

^{1}**H NMR** (400 MHz, CDCl$_3$):

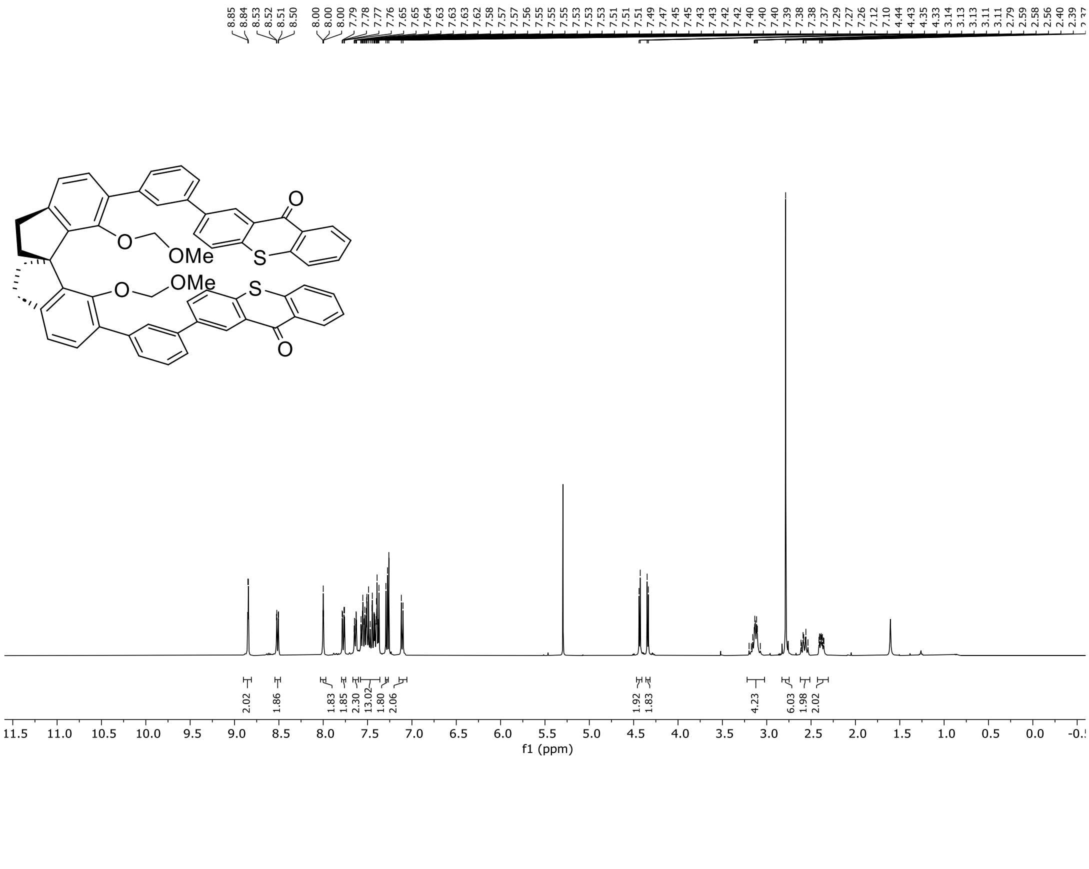

^{13}C NMR (101 MHz, $CDCl_3$):

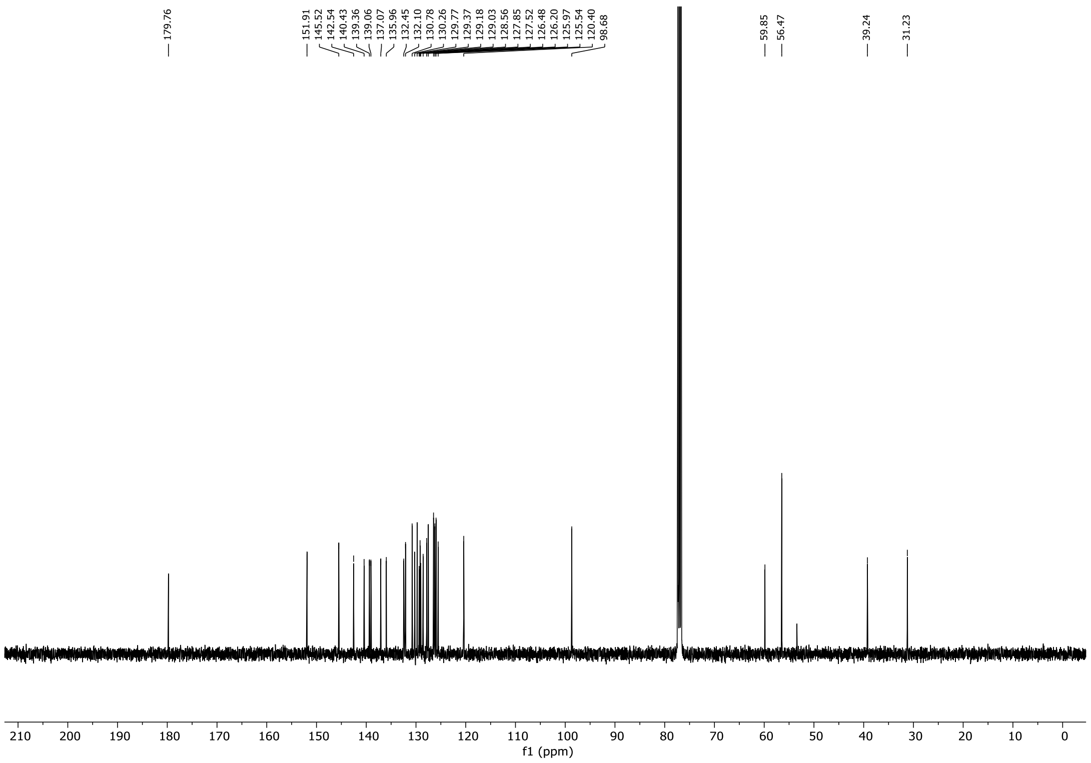

2,2'-((12-Hydroxy-12-oxido-4,5,6,7-tetrahydrodiindeno[7,1-*de*:1',7'-*fg*][1,3,2]dioxaphosphocine-1,10-diyl)bis(3,1-phenylene))bis(9*H*-thioxanthen-9-one) (9)

^{1}H NMR (400 MHz, DMSO-d_6):

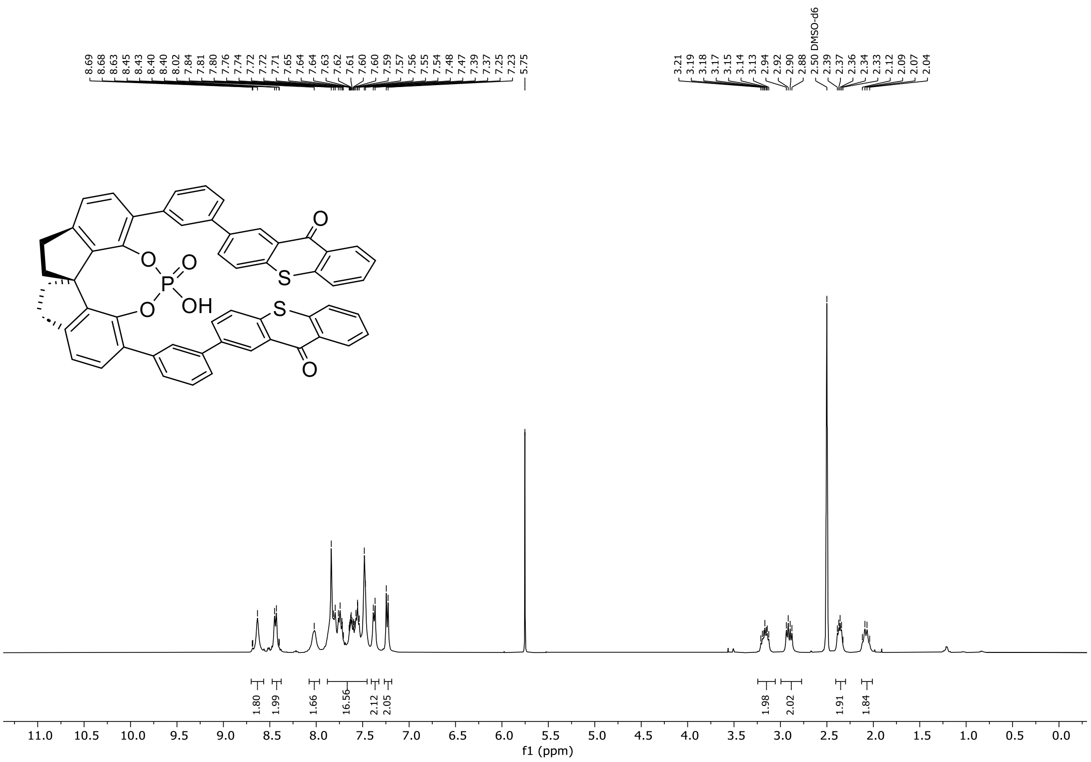

^{13}C NMR (126 MHz, DMSO-d_6):

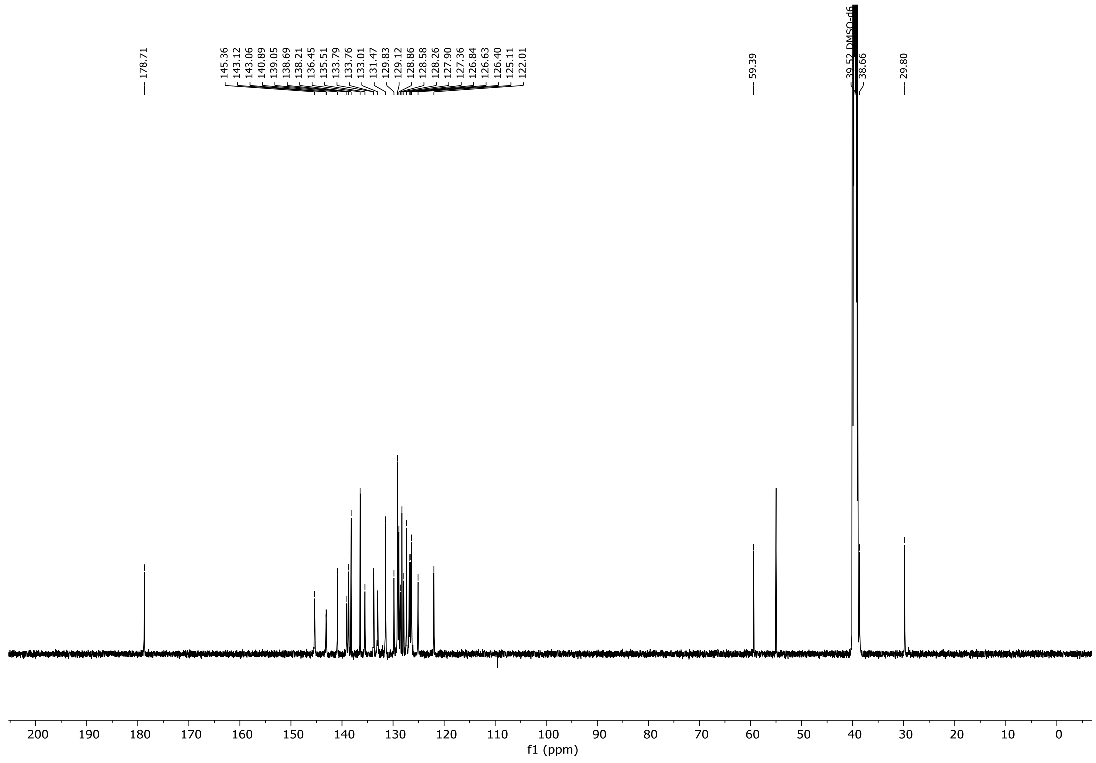

^{31}P NMR (203 MHz, DMSO-d_6):

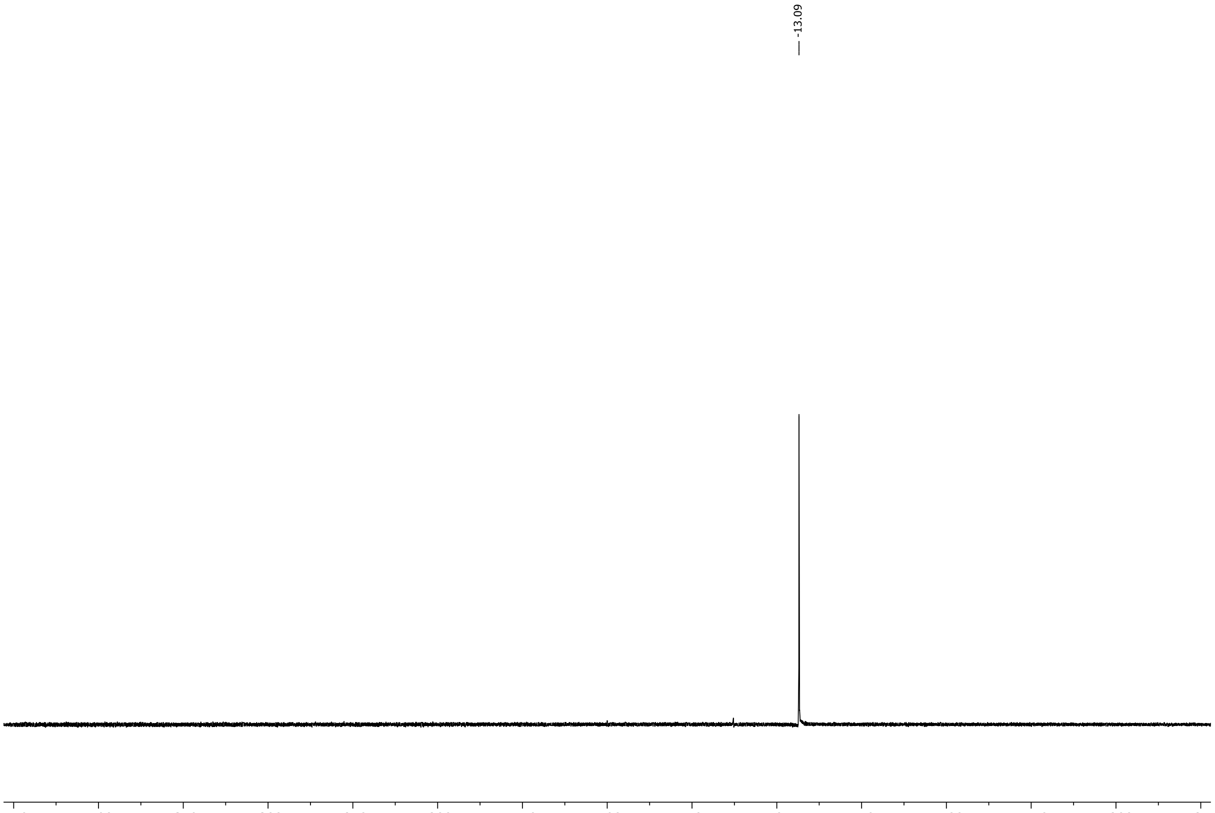

15.3 Primary Photoproducts

5-Hydroxy-1,4,4a,5,6,7,8,9-octahydro-5,9a-(epoxymethano)-1,4-methanobenzo[7]annulen-10-one (*rac*-3a)

^{1}H NMR (500 MHz, CD_2Cl_2):

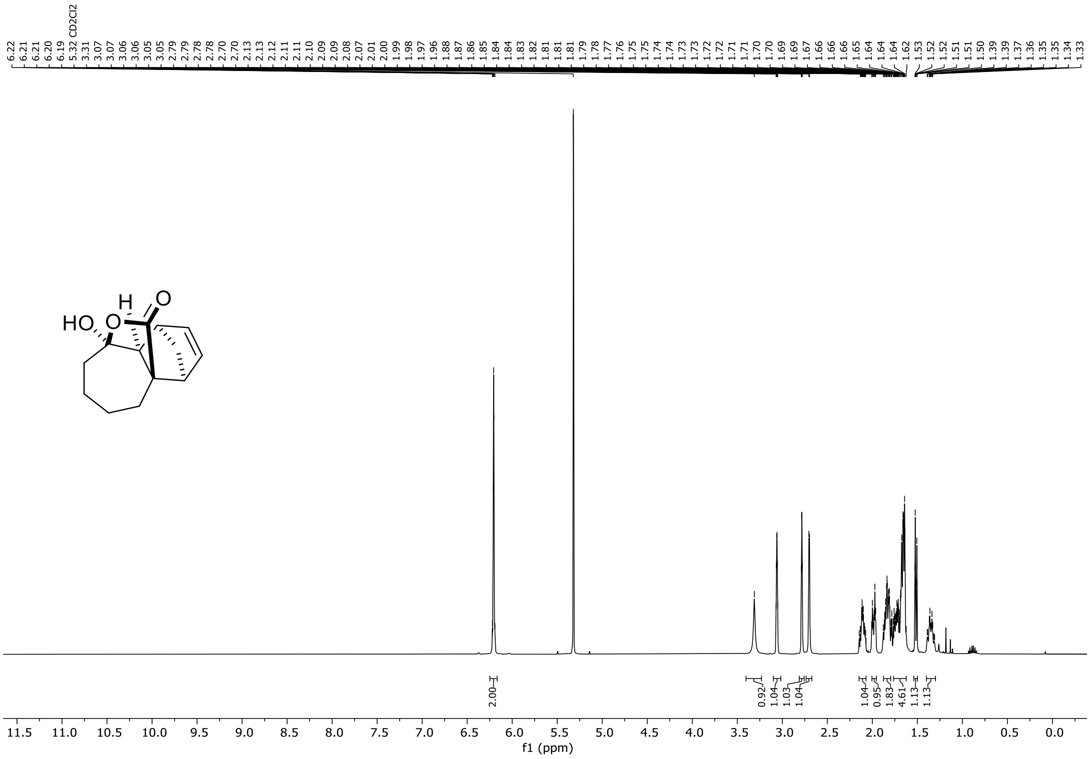

^{13}C NMR (101 MHz, CD_2Cl_2):

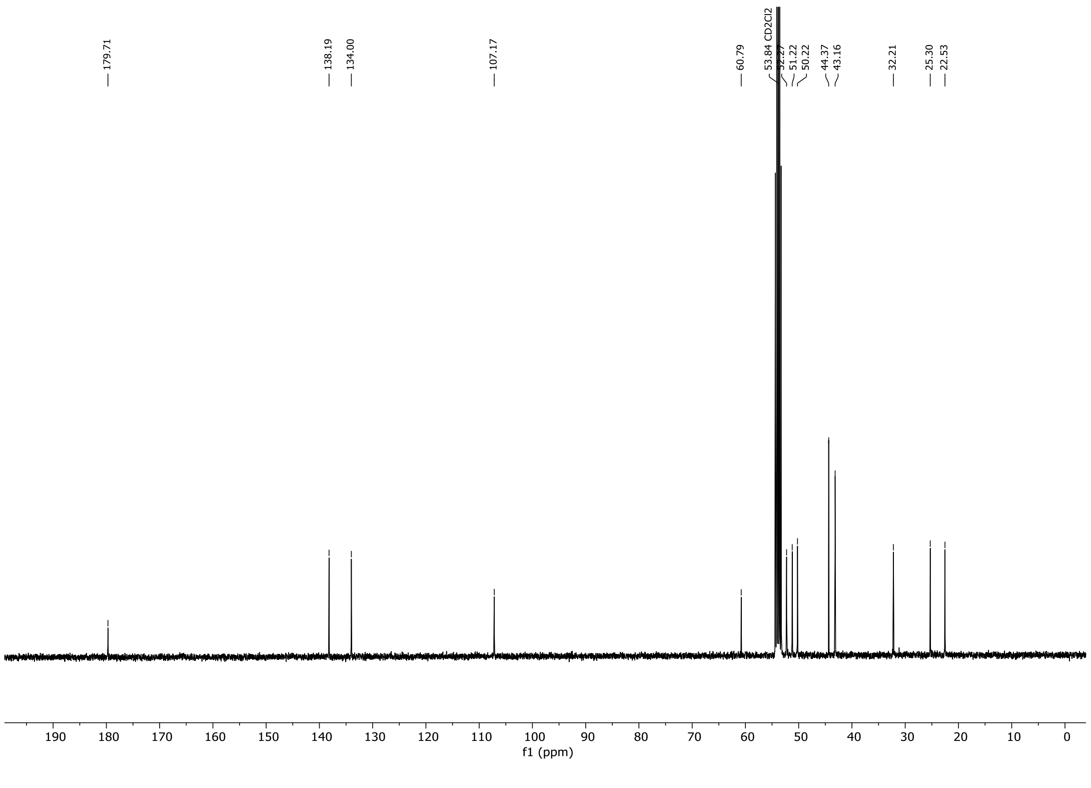

5'-Hydroxy-1',4',4a',5',6',7',8',9'-octahydrospiro[cyclopropane-1,12'-[5,9a](epoxymethano)[1,4]methanobenzo[7]annulen]-10'-one (*rac*-3b/*rac*-3b')

^{1}H NMR (500 MHz, CD_2Cl_2):

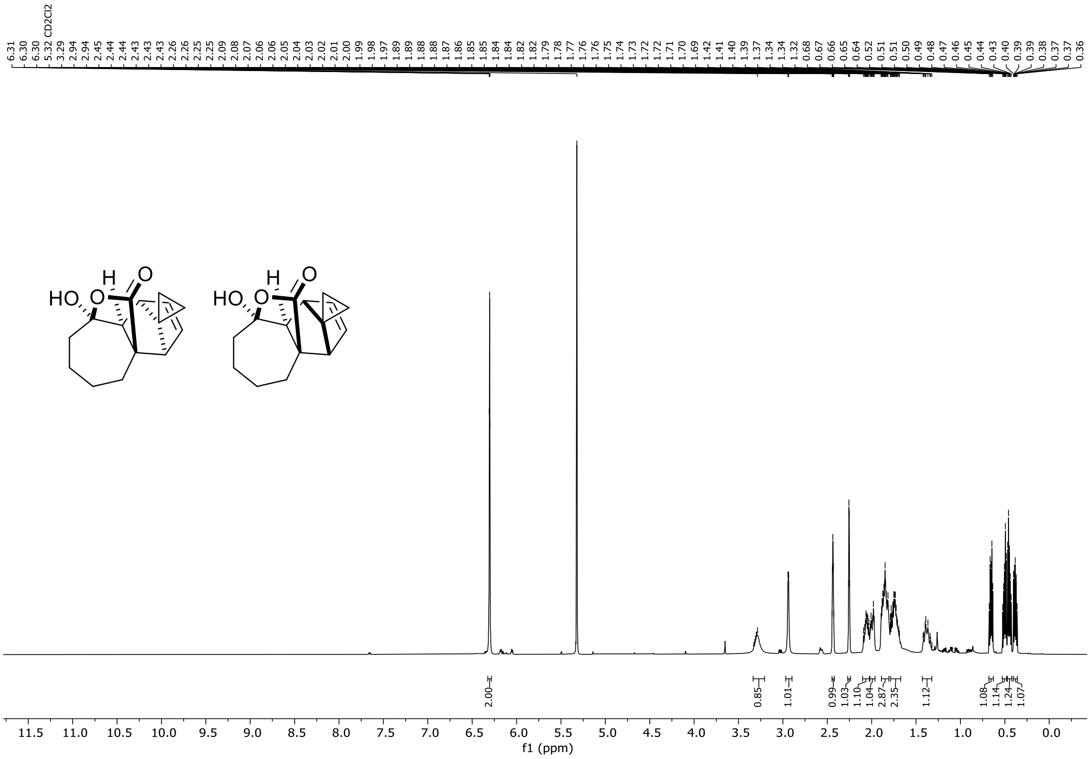

^{13}C NMR (101 MHz, CD_2Cl_2):

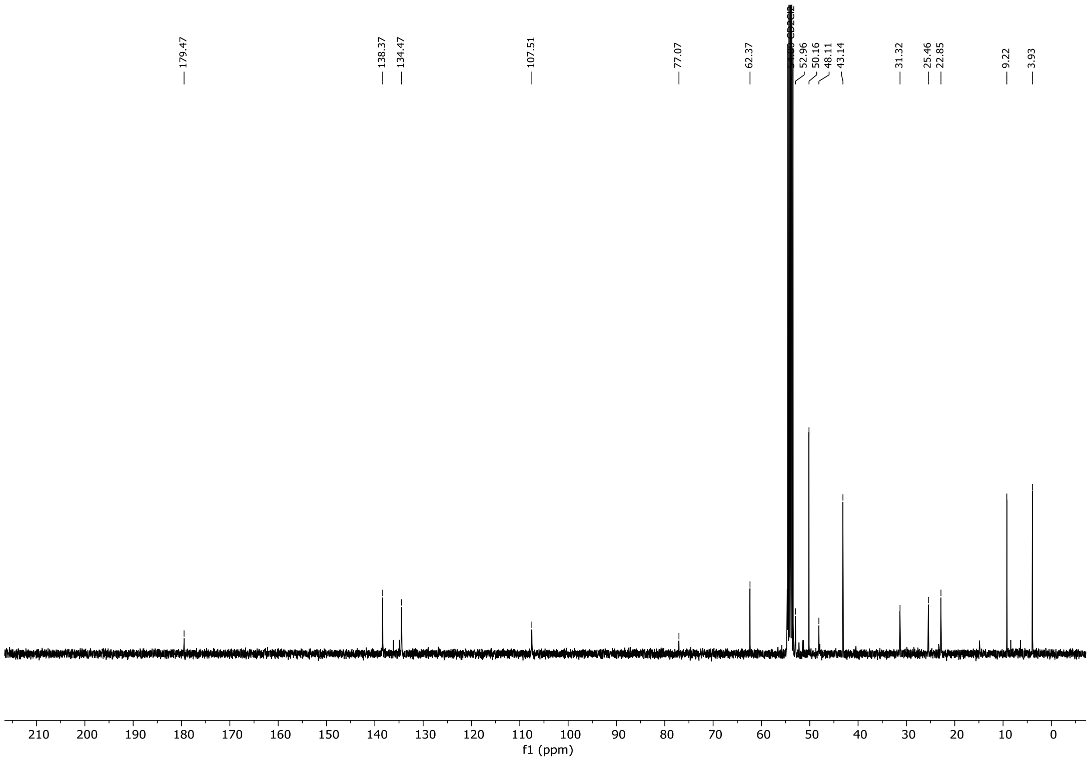

5-Hydroxy-1-propyl-1,4,4a,5,6,7,8,9-octahydro-5,9a-(epoxymethano)benzo[7]annulen-10-one (*rac*-3c/*rac*-3c’)

^{1}H NMR (500 MHz, CD_2Cl_2):

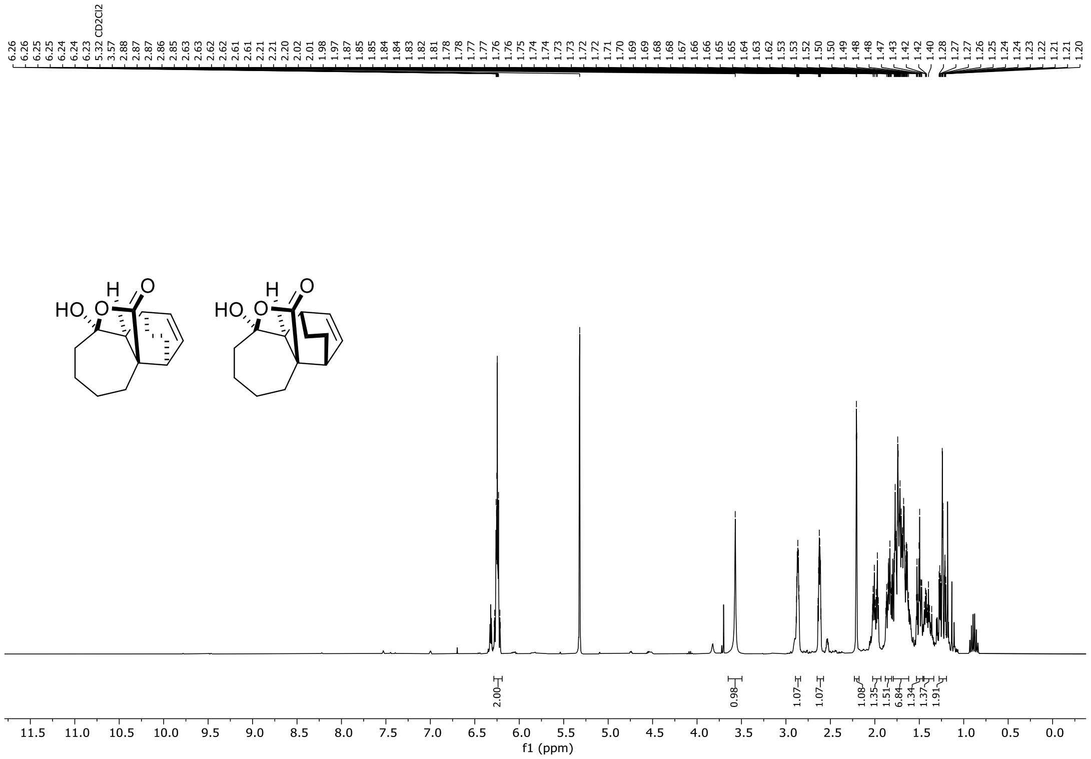

^{13}C NMR (101 MHz, CD_2Cl_2):

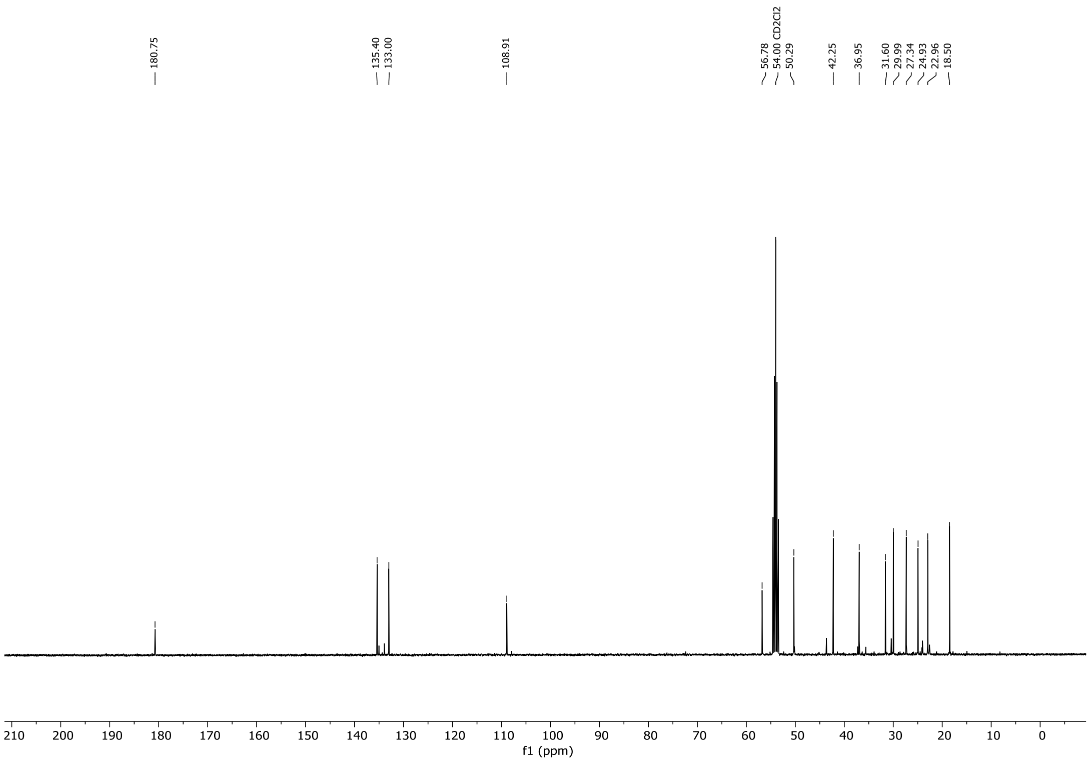

5-Hydroxy-1,4,4a,5,6,7,8,9-octahydro-1,4-epoxy-5,9*a*-(epoxymethano)benzo[7]annulen-10-one (*rac*-3d/*rac*-3d')

^{1}H NMR (400 MHz, CD_2Cl_2):

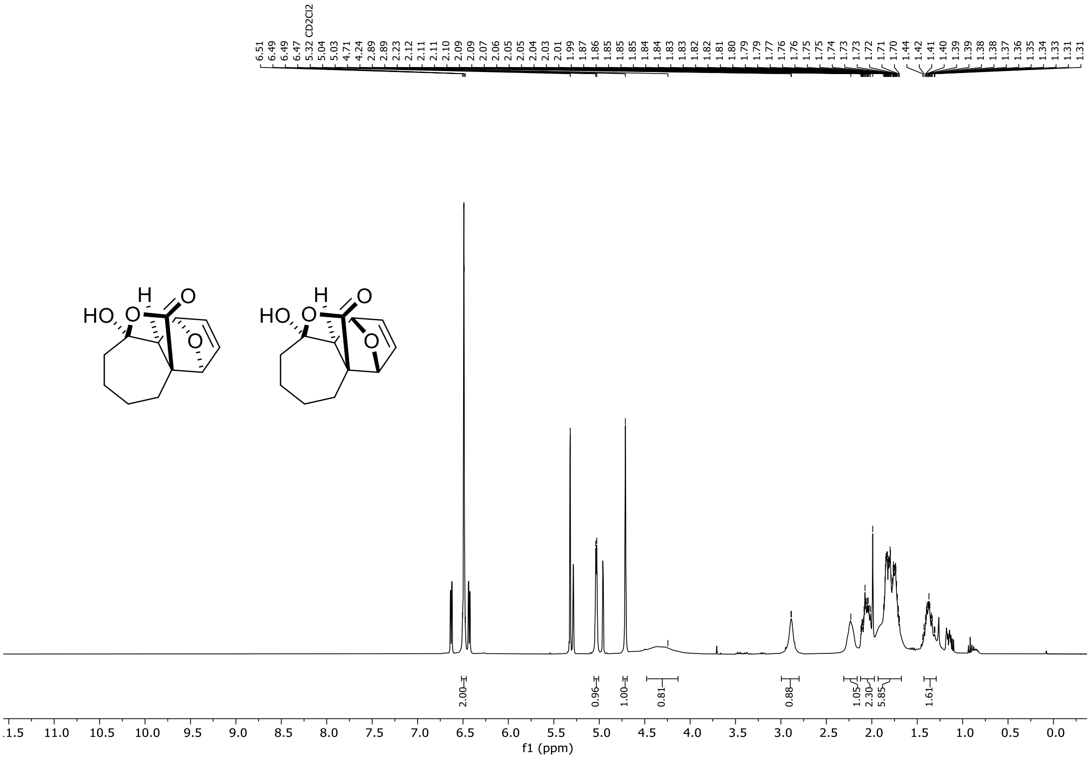

^{13}C NMR (101 MHz, CD_2Cl_2):

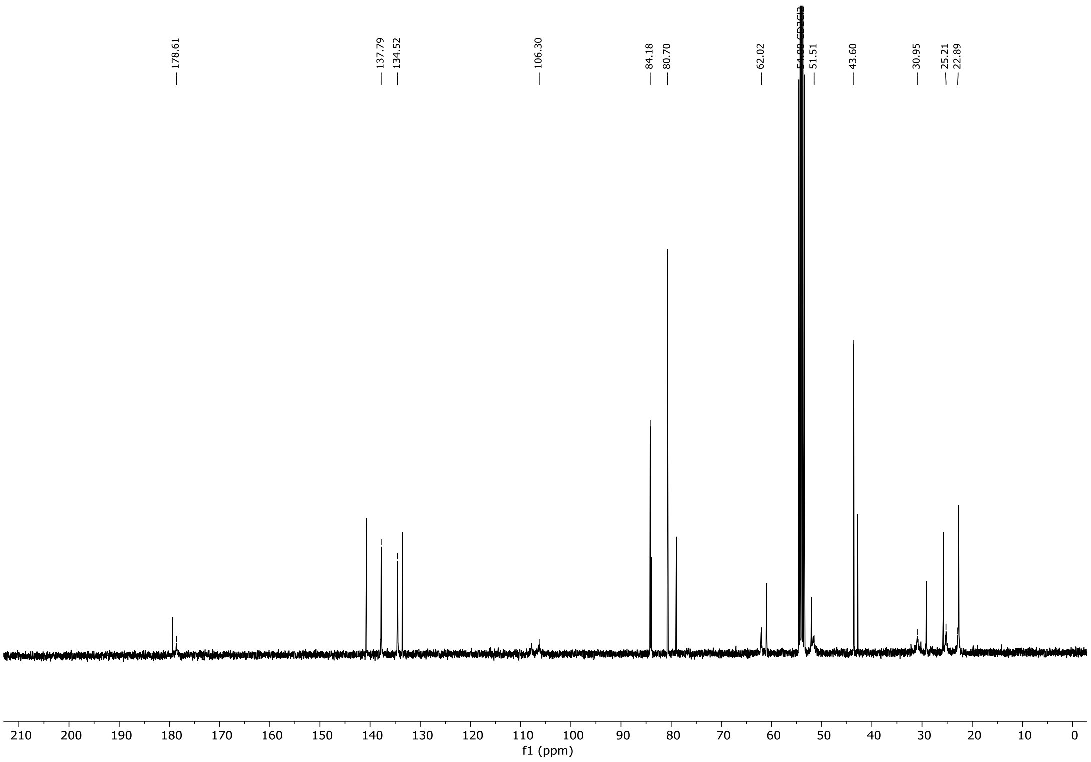

5-Hydroxy-1,4-dimethyl-1,4,4a,5,6,7,8,9-octahydro-1,4-epoxy-5,9a-(epoxymethano)benzo [7]annulen-10-one (*rac*-3e)

^{1}H NMR (400 MHz, CD_2Cl_2):

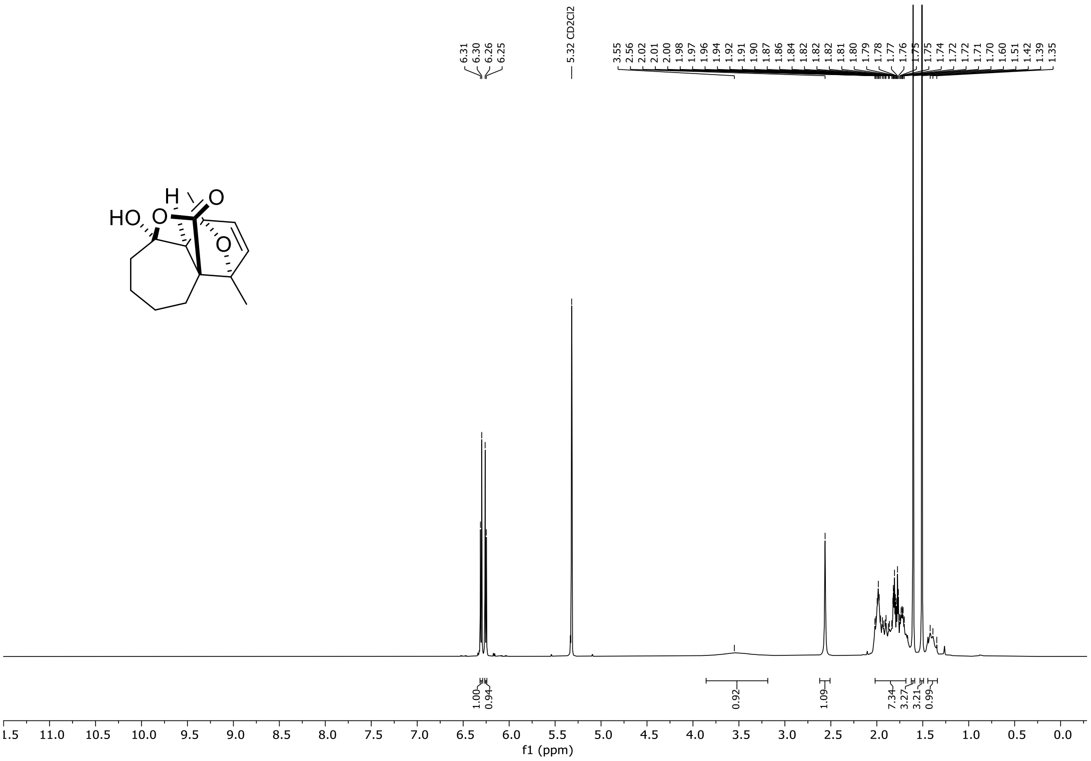

^{13}C NMR (126 MHz, CD_2Cl_2):

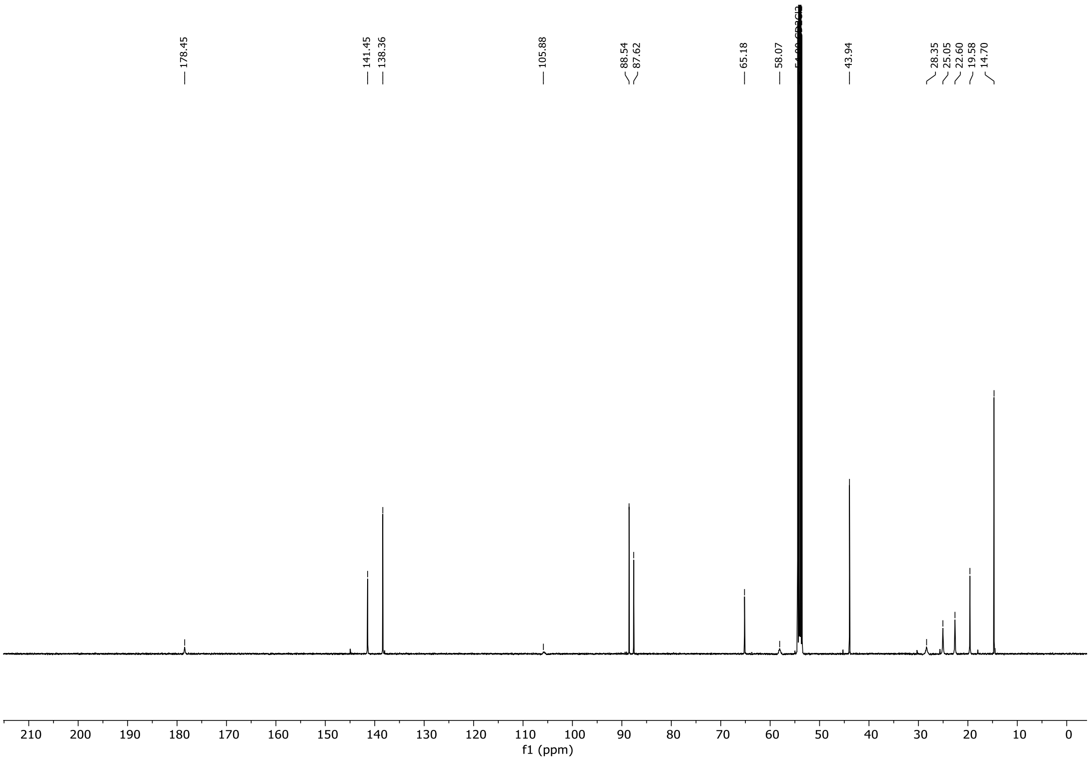

1,4-Diethyl-5-hydroxy-1,4,4a,5,6,7,8,9-octahydro-1,4-epoxy-5,9a-(epoxymethano)benzo[7]annulen-10-one (*rac*-3f)

^{1}H NMR (400 MHz, CD_2Cl_2):

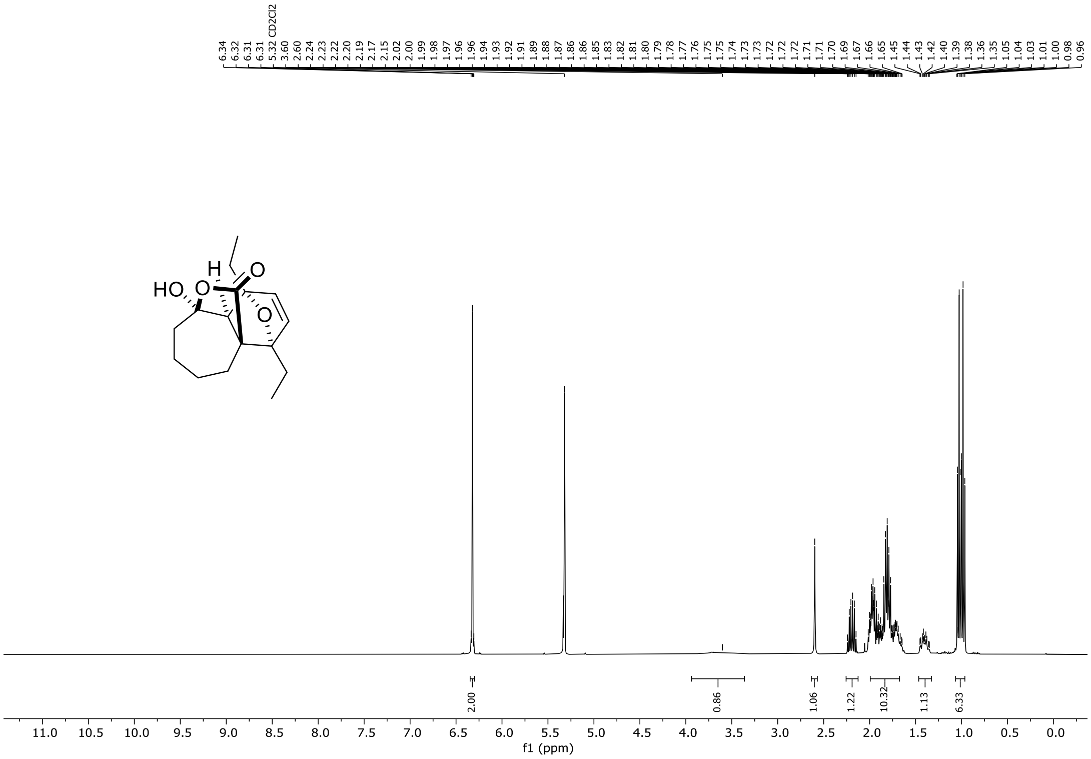

^{13}C NMR (101 MHz, CD_2Cl_2):

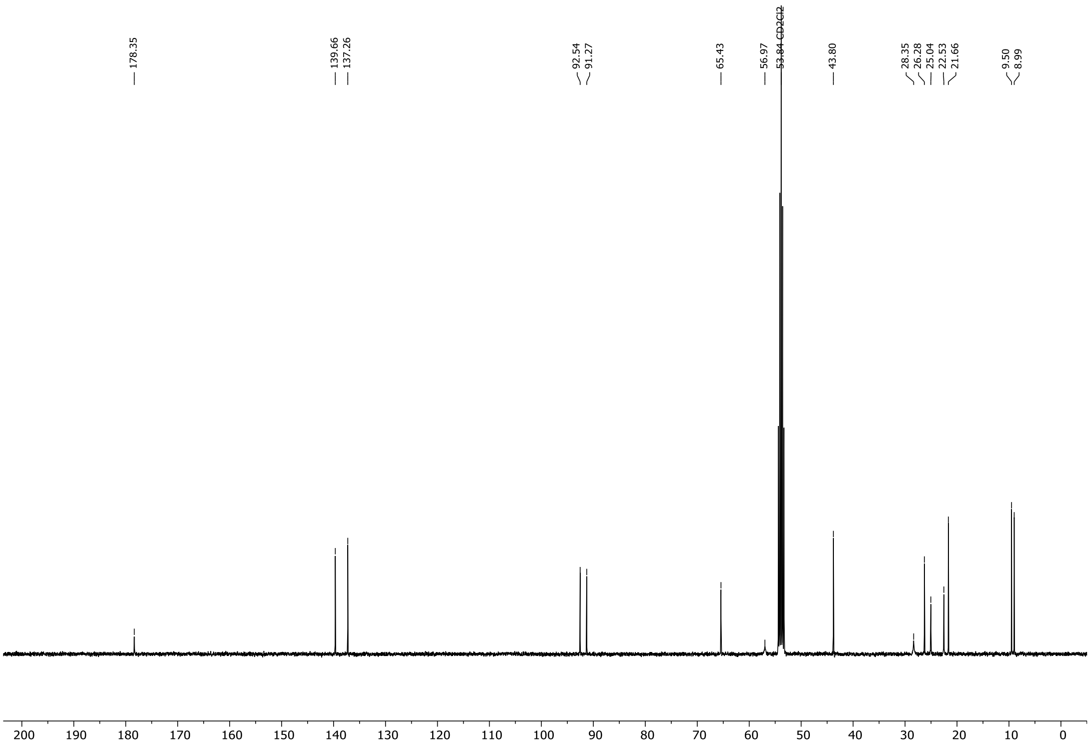

5-Hydroxy-1,4-dipropyl-1,4,4a,5,6,7,8,9-octahydro-1,4-epoxy-5,9a-(epoxymethano)benzo [7]annulen-10-one (*rac*-3g)

^{1}H NMR (400 MHz, CD_2Cl_2):

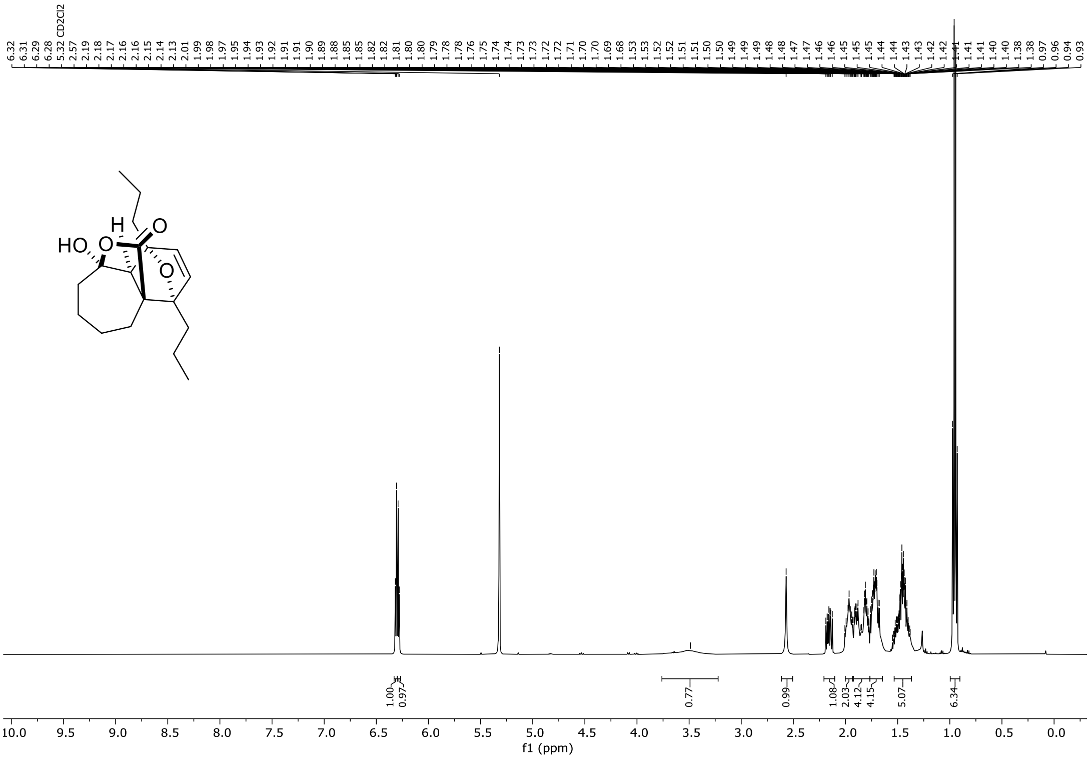

^{13}C NMR (101 MHz, CD_2Cl_2):

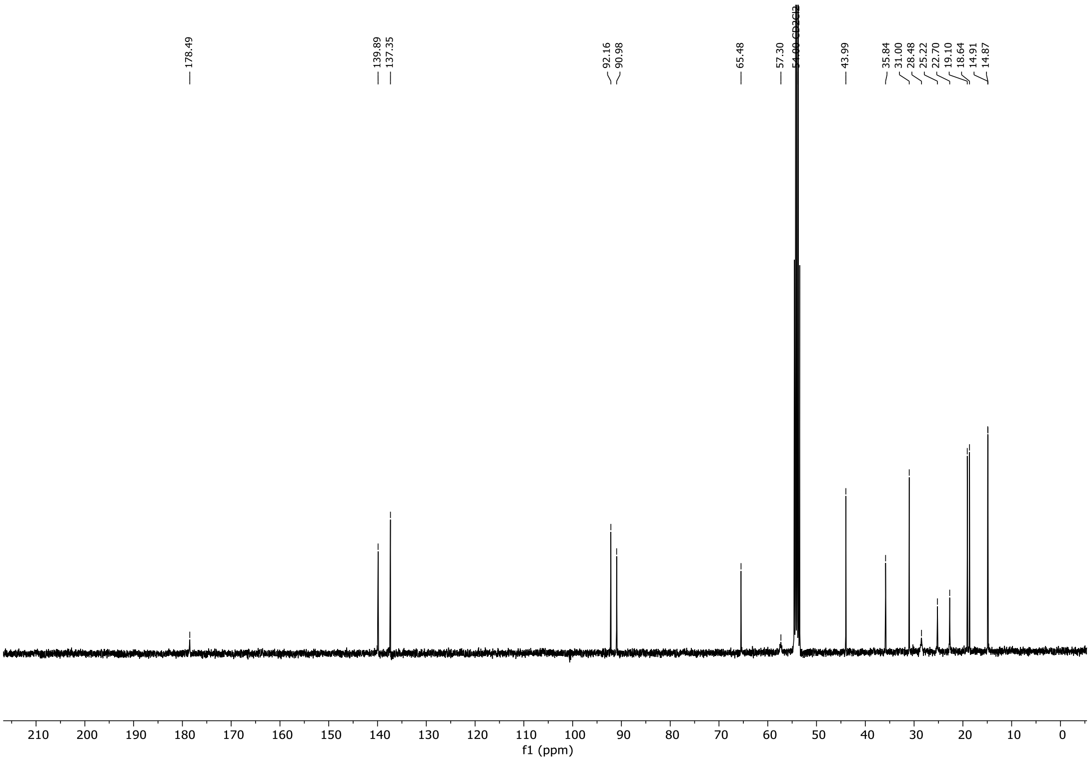

1,4-Dibutyl-5-hydroxy-1,4,4a,5,6,7,8,9-octahydro-1,4-epoxy-5,9a-(epoxymethano)benzo[7]annulen-10-one (*rac*-3h)

^{1}H NMR (400 MHz, CD_2Cl_2):

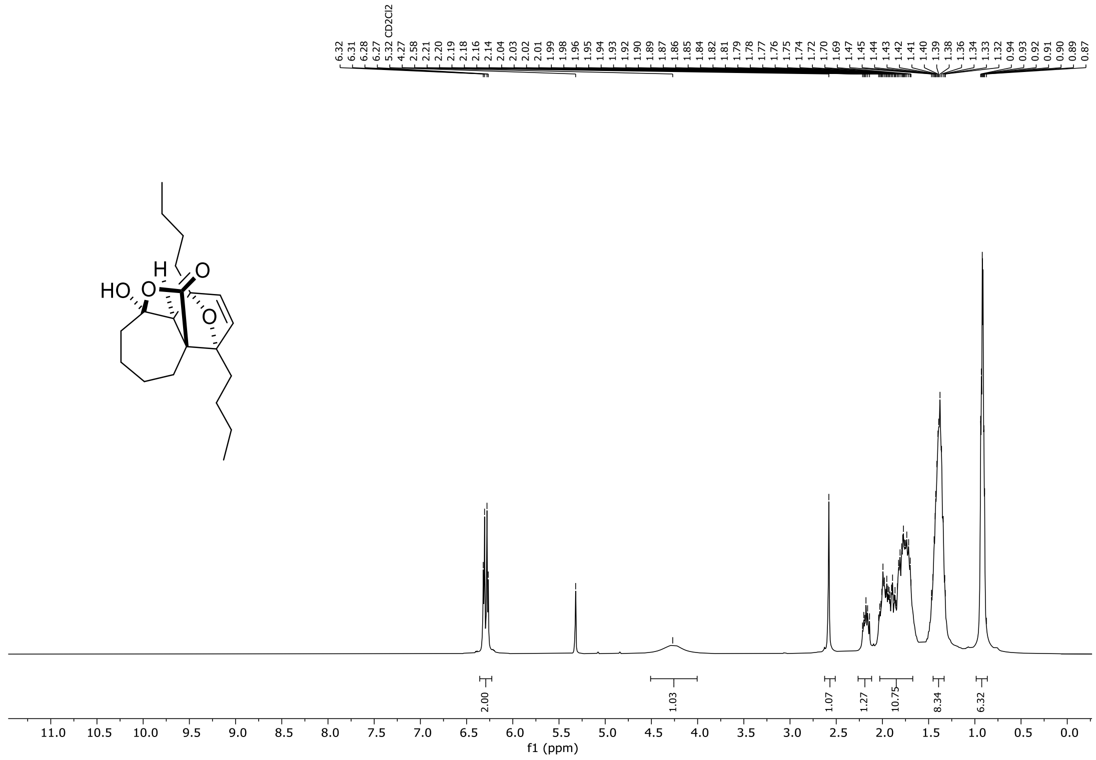

^{13}C NMR (101 MHz, CD_2Cl_2):

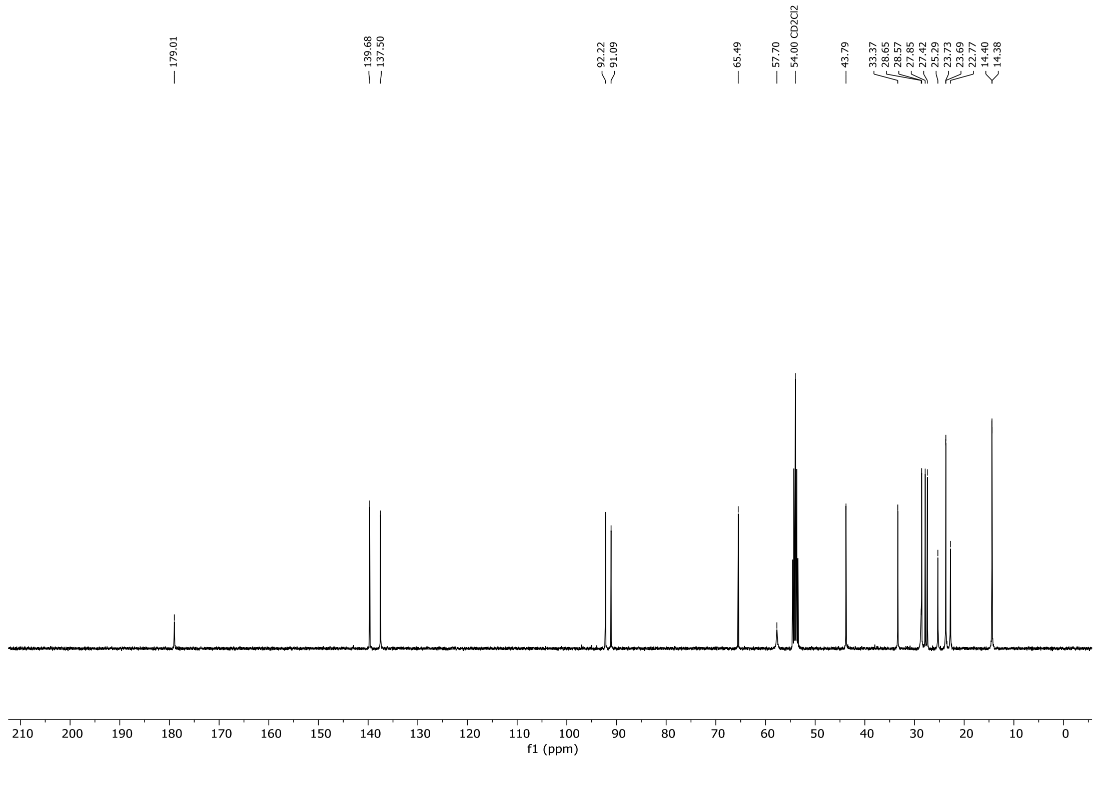

5-Hydroxy-1,2-dimethyl-1,4,4a,5,6,7,8,9-octahydro-1,4-epoxy-5,9a-(epoxymethano)benzo [7]annulen-10-one (*rac*-3i/*rac*-3i'/*rac*-3i")

^{1}**H NMR** (400 MHz, CD_2Cl_2):

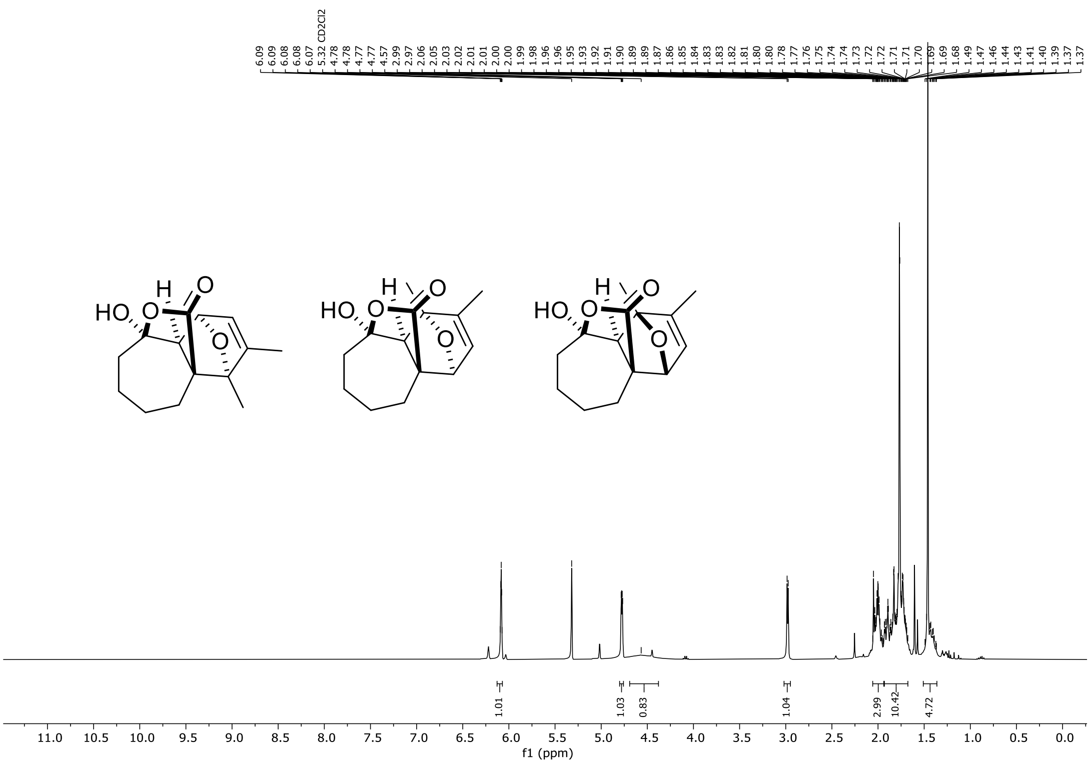

^{13}**C NMR** (101 MHz, CD_2Cl_2):

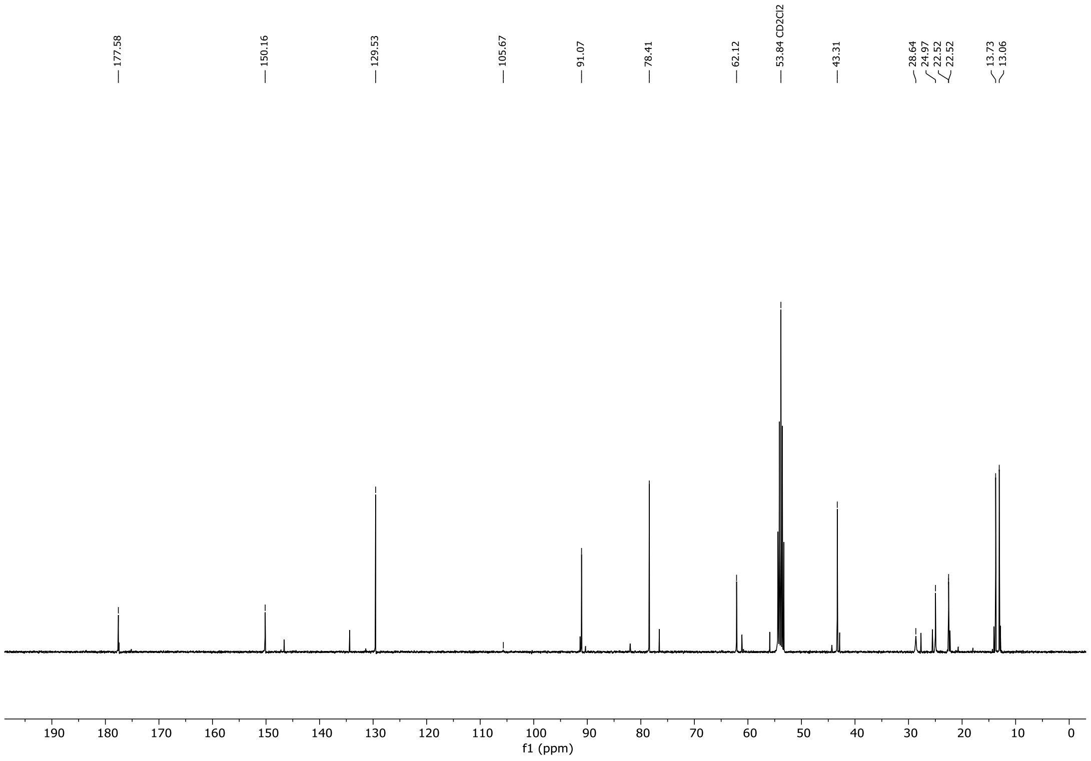

3-Bromo-5-hydroxy-1,4,4a,5,6,7,8,9-octahydro-1,4-epoxy-5,9a-(epoxymethano)benzo[7]annulen-10-one (*rac*-3j')

^{1}H NMR (400 MHz, CD_2Cl_2):

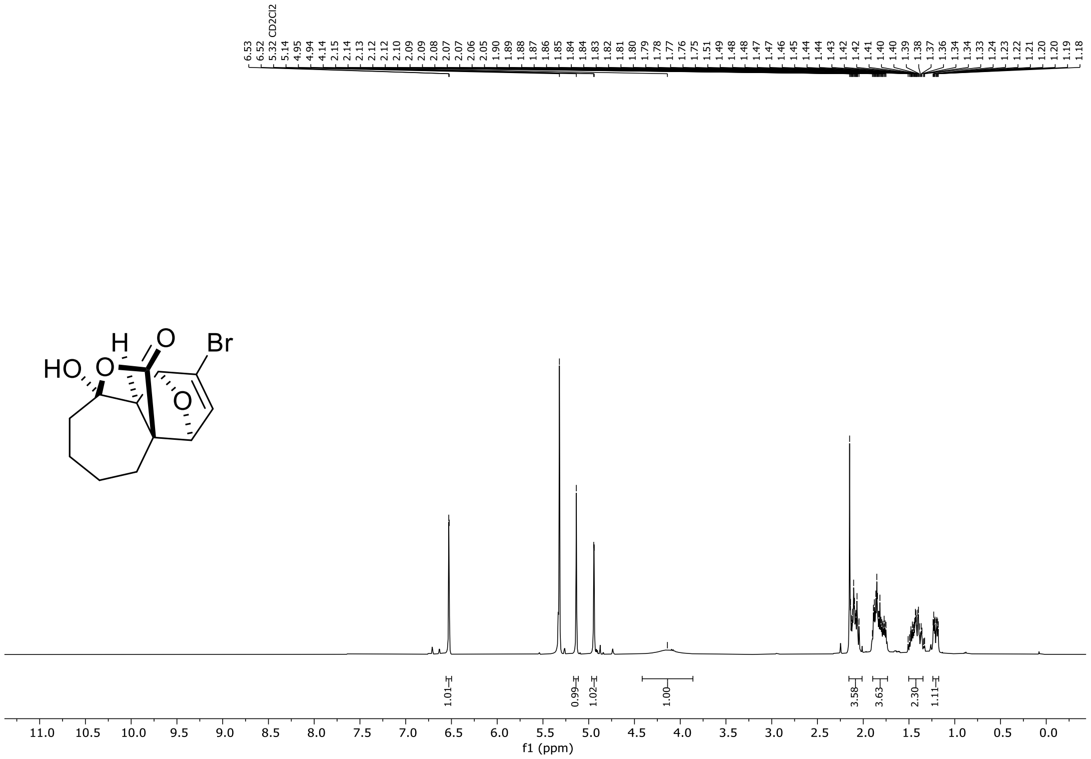

^{13}C NMR (101 MHz, CD_2Cl_2):

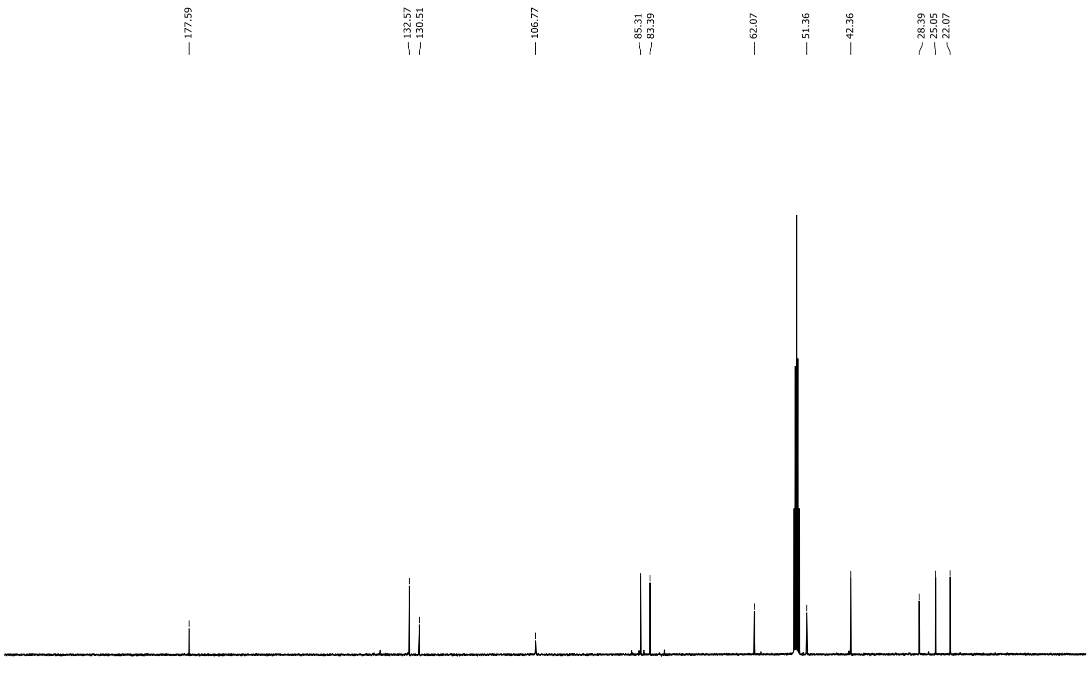

2-Bromo-5-hydroxy-1,4,4a,5,6,7,8,9-octahydro-1,4-epoxy-5,9a-(epoxymethano)benzo[7]annulen-10-one (*rac*-3j)

^{1}H NMR (400 MHz, CD_2Cl_2):

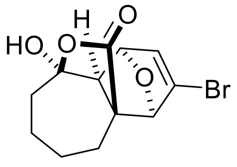

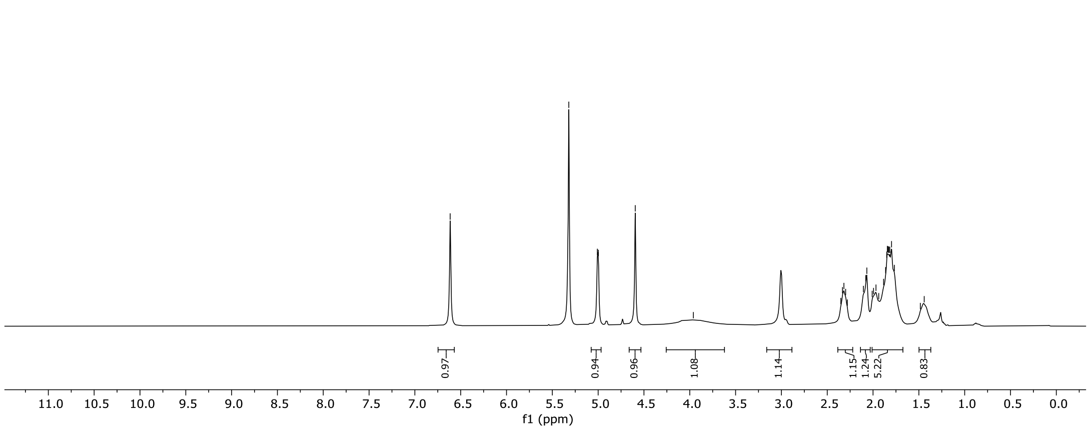

^{13}C NMR (101 MHz, CD_2Cl_2):

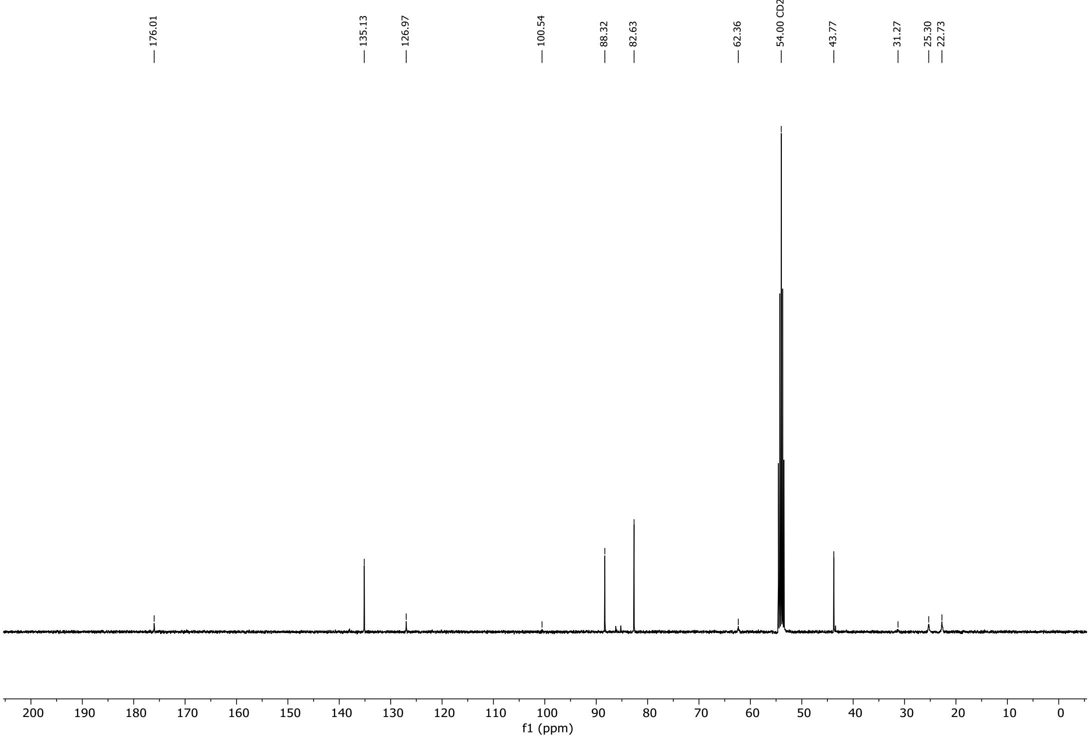

5-Hydroxy-10-oxo-4a,5,6,7,8,9-hexahydro-1,4-epoxy-5,9a-(epoxymethano)benzo[7]annulen-1(4H)-yl)methyl acetate (*rac*-3k)

^{1}H NMR (400 MHz, CD_2Cl_2):

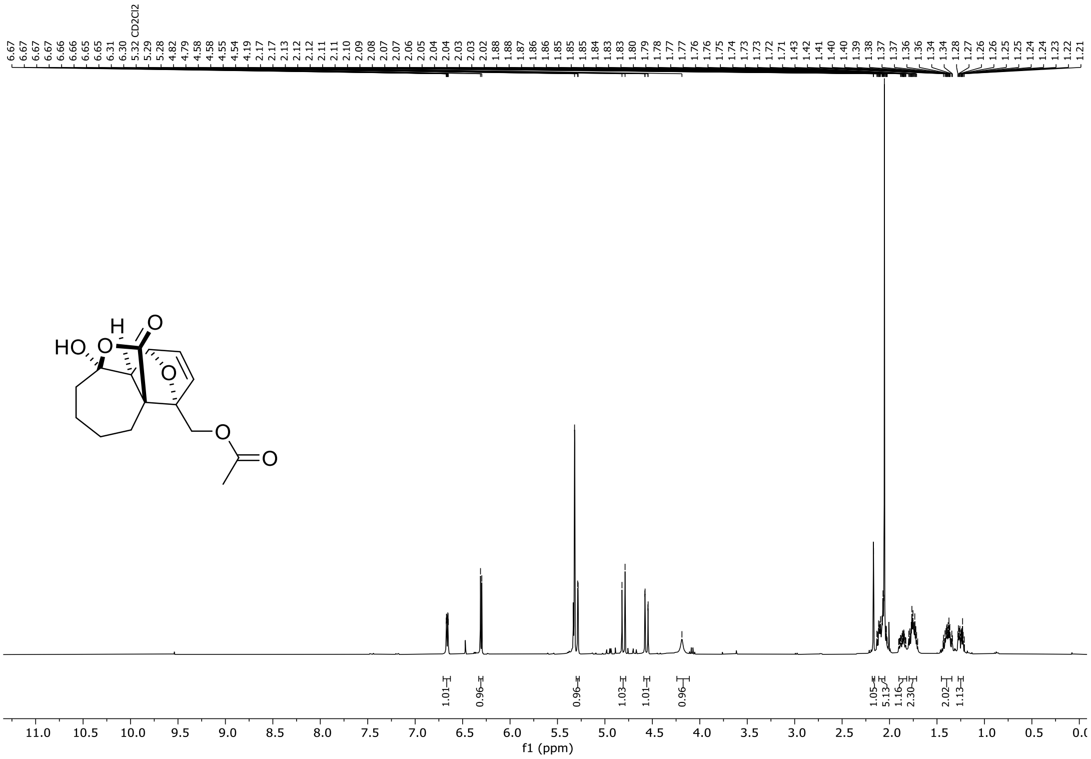

^{13}C NMR (101 MHz, CD_2Cl_2):

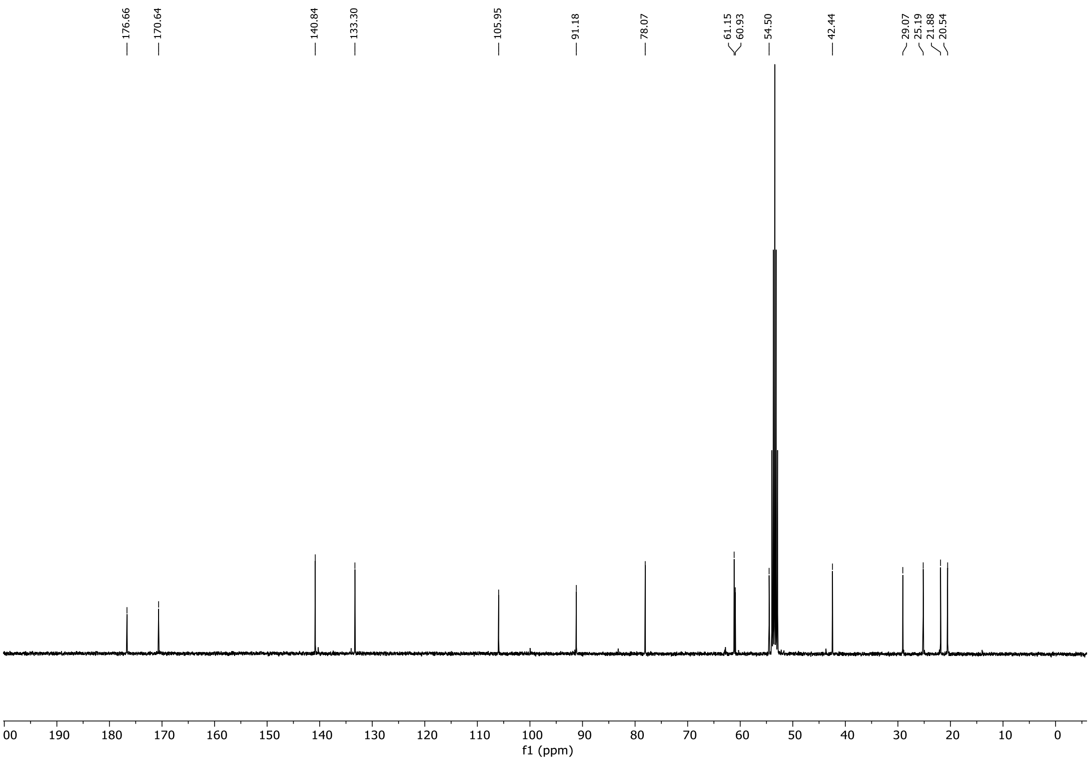

5-Hydroxy-1-methyl-1,4,4*a*,5,6,7,8,9-octahydro-1,4-epoxy-5,9*a*-(epoxymethano)benzo[7] *a*nnulen-10-one (*rac*-3l/*rac*-3l')

^{1}H NMR (500 MHz, CD_2Cl_2):

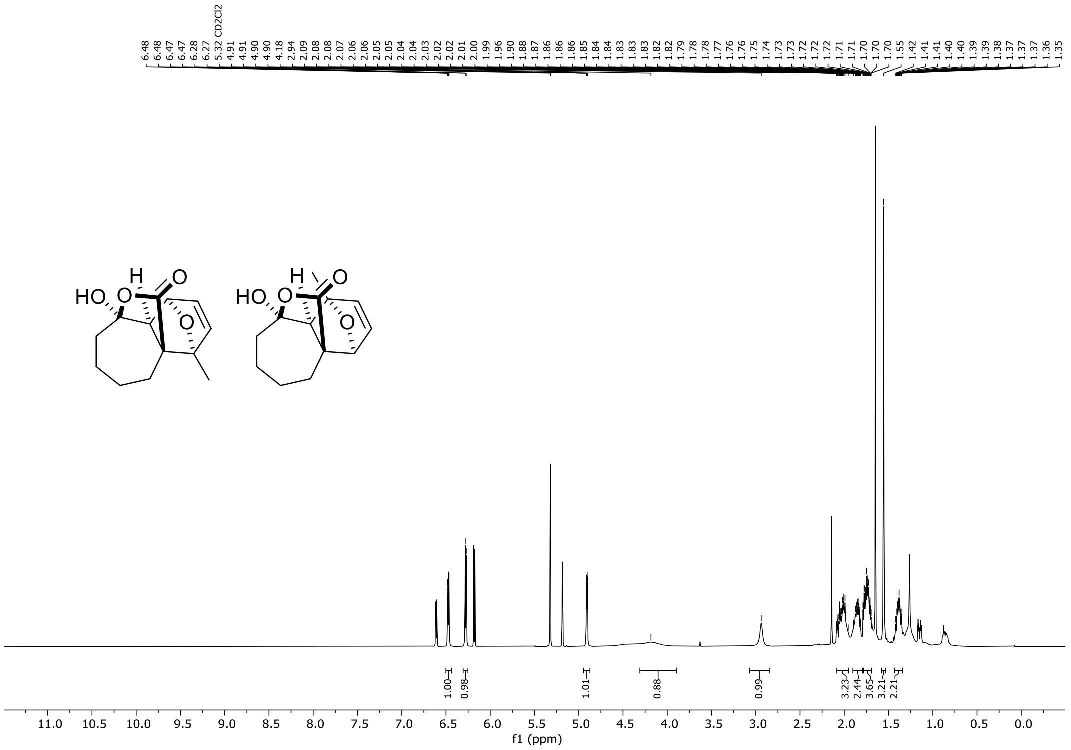

^{13}C NMR (101 MHz, CD_2Cl_2):

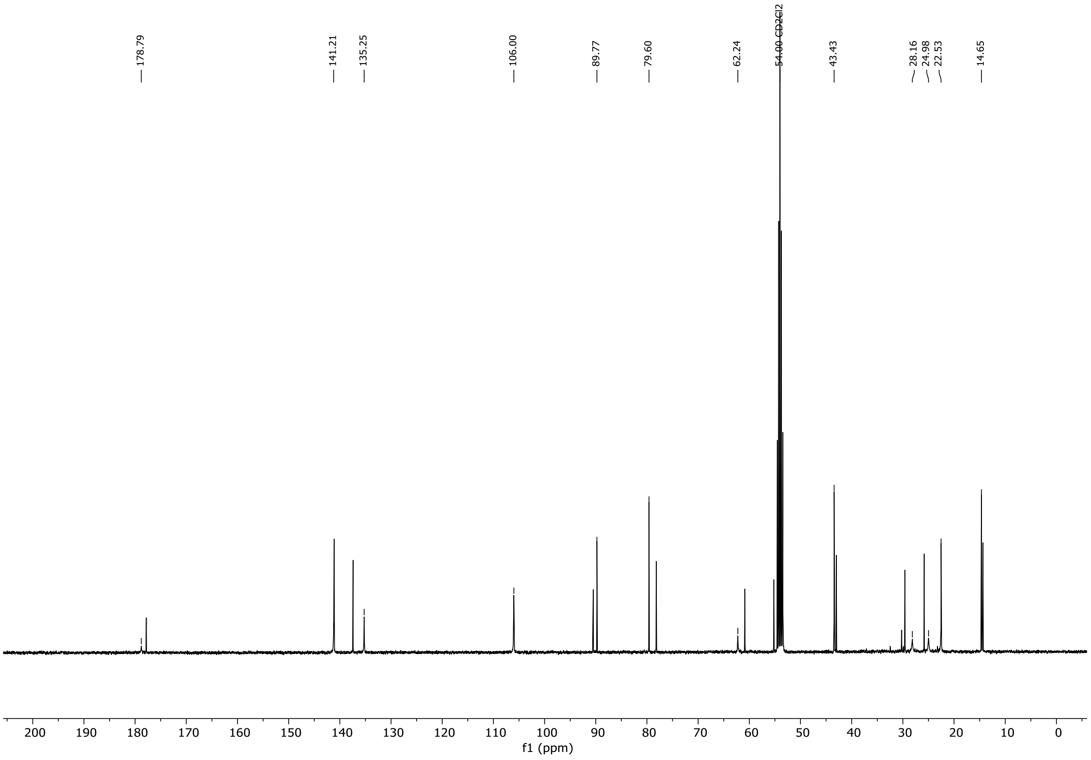

15.4 Derivatized Photoproducts

Benzyl 9-oxo-1,4,5,6,7,8,9,9a-octahydro-4a*H*-1,4-methanobenzo[7]annulene-4a-carboxylate (*rac*-4a/*rac*-4a')

^{1}H NMR (400 MHz, CD_2Cl_2):

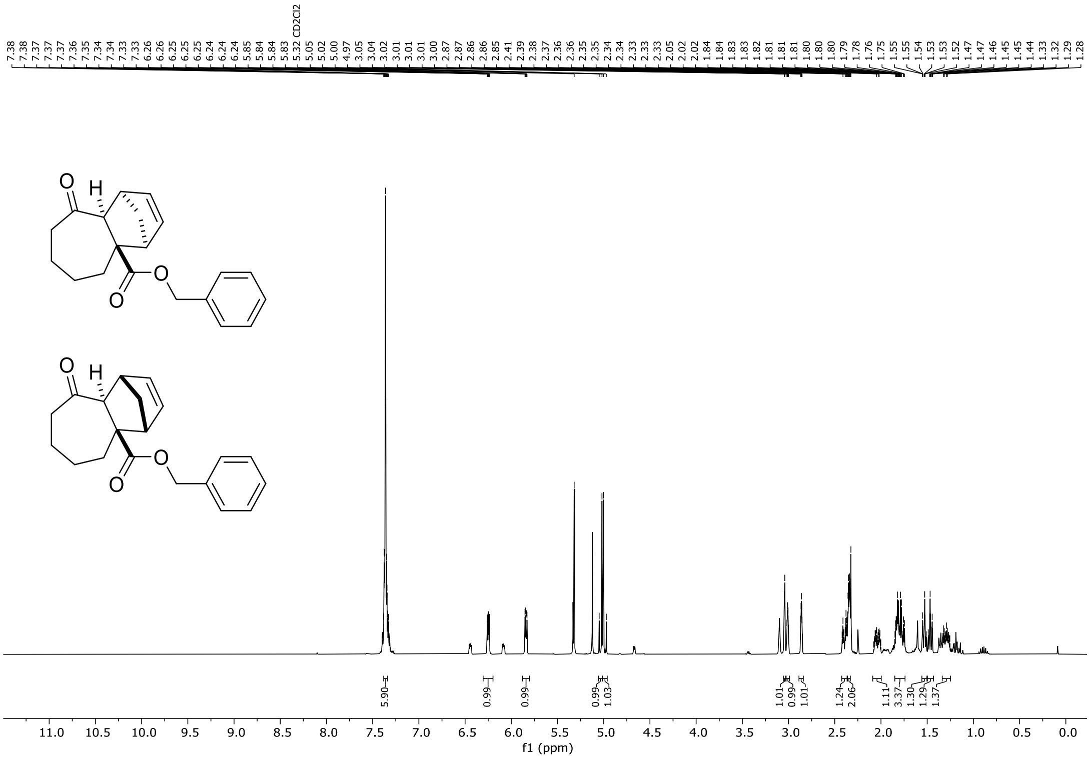

^{13}C NMR (101 MHz, CD_2Cl_2):

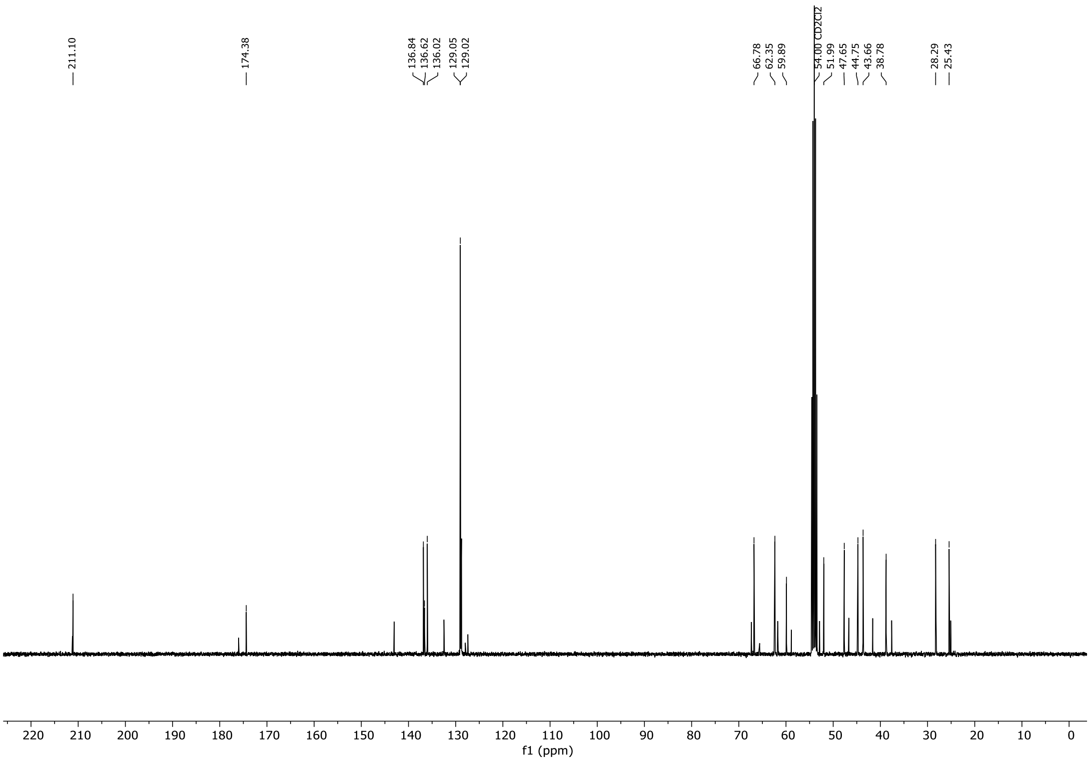

Benzyl 9-oxo-1,4,5,6,7,8,9,9a-octahydro-4a*H*-1,4-epoxybenzo[7]annulene-4a-carboxylate (*rac*-4d)

^{1}H NMR (500 MHz, CD_2Cl_2):

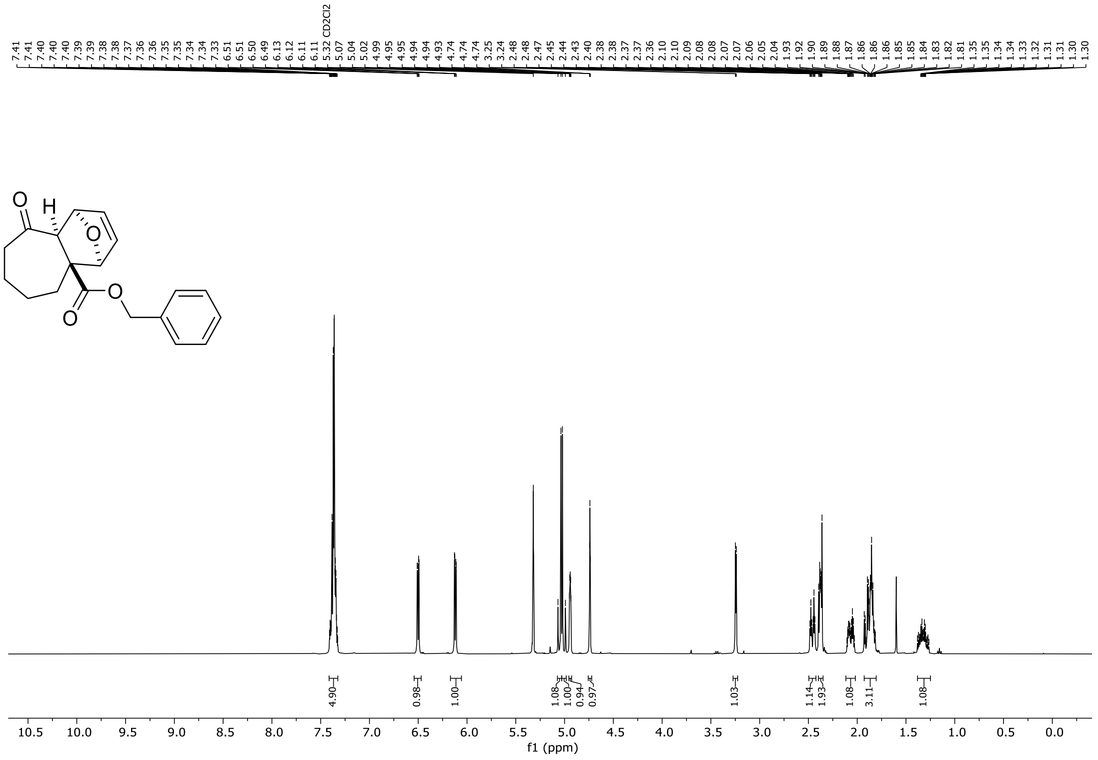

^{13}C NMR (126 MHz, CD_2Cl_2):

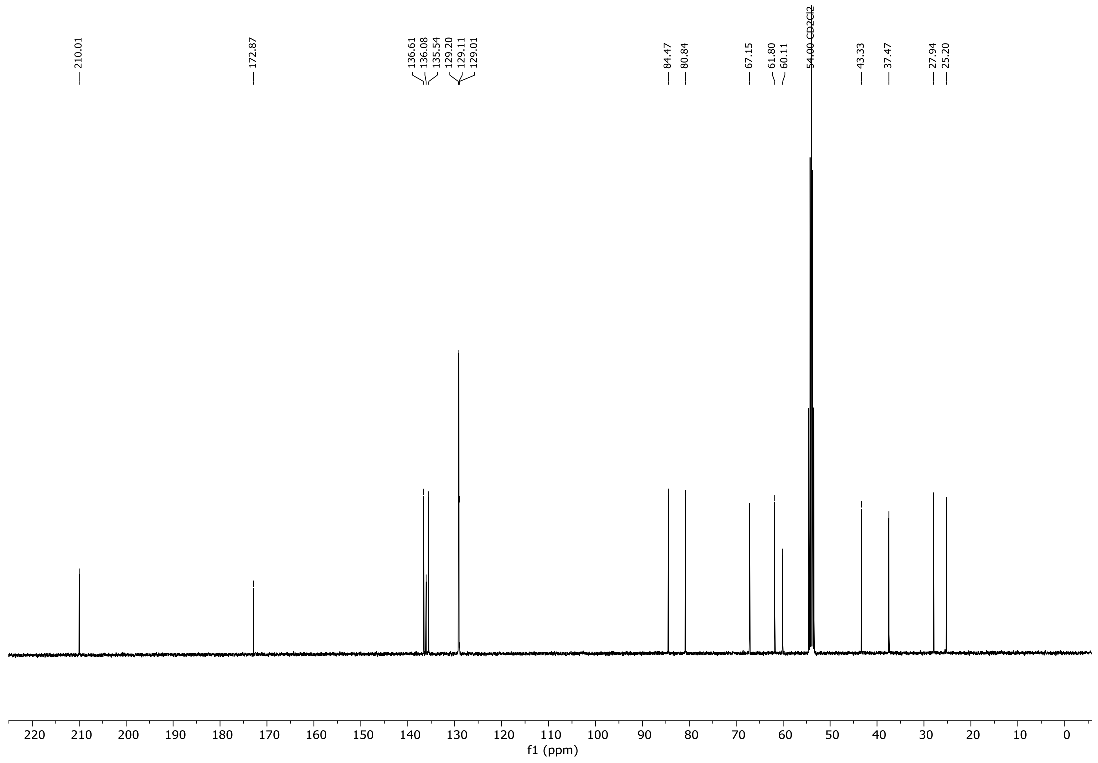

Benzyl 9-oxo-1,4,5,6,7,8,9,9a-octahydro-4a*H*-1,4-epoxybenzo[7]annulene-4a-carboxylate (*rac*-4d')

^{1}H NMR (500 MHz, CD_2Cl_2):

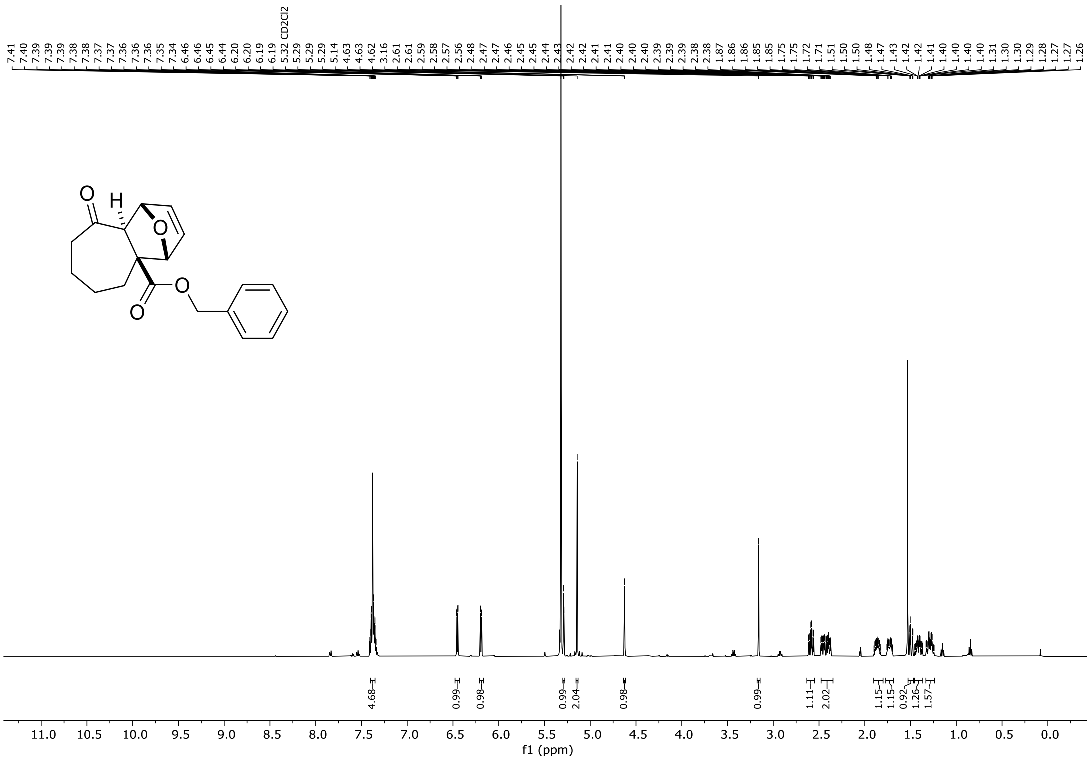

^{13}C NMR (126 MHz, CD_2Cl_2):

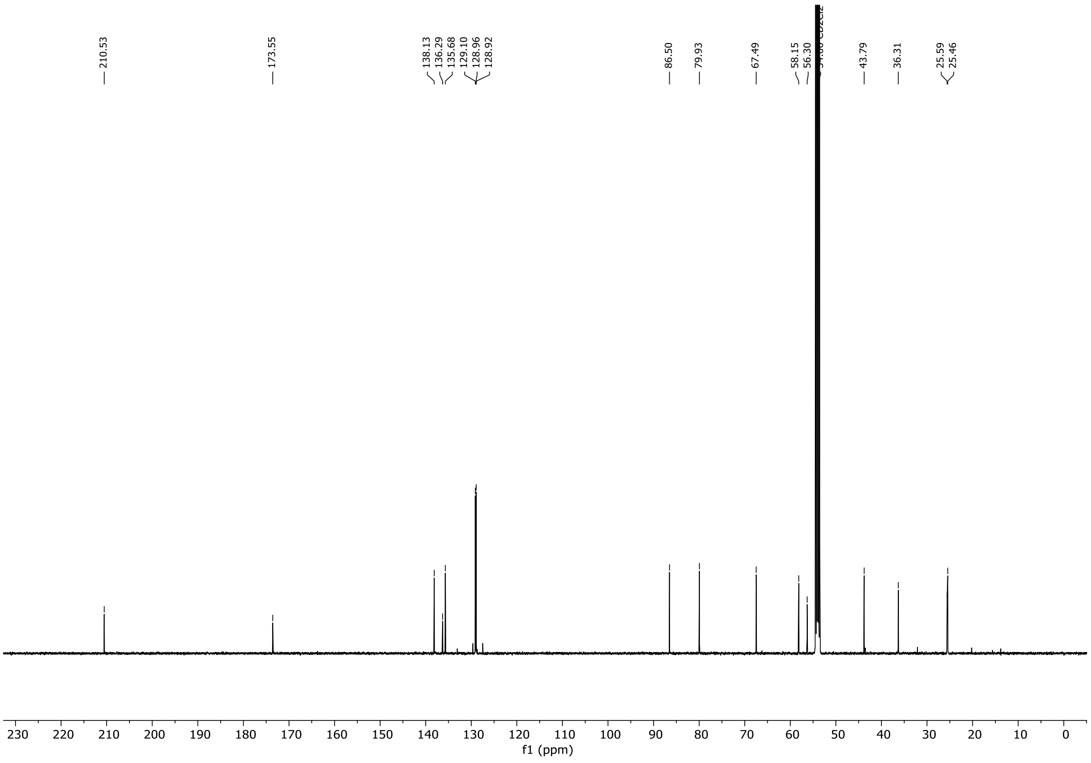

Benzyl 1,4-dimethyl-9-oxo-1,4,5,6,7,8,9,9a-octahydro-4a*H*-1,4-epoxybenzo[7]annulene-4a-carboxylate (*rac*-4e)

^{1}H NMR (500 MHz, CD_2Cl_2):

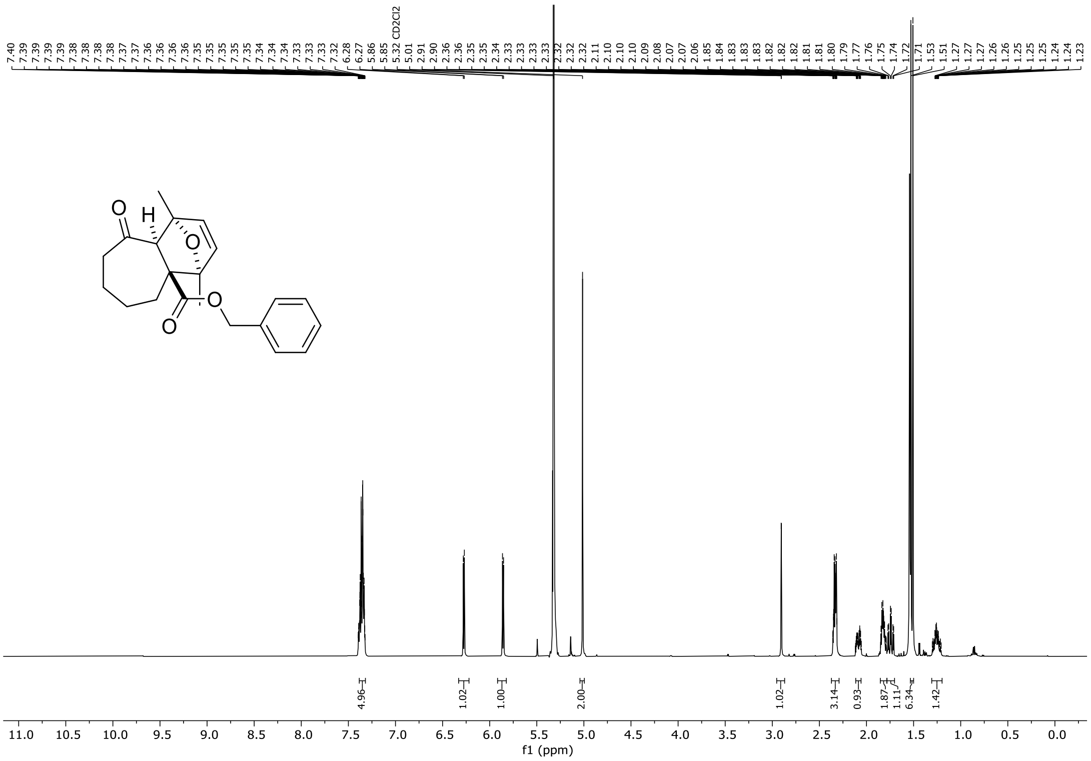

^{13}C NMR (126 MHz, CD_2Cl_2):

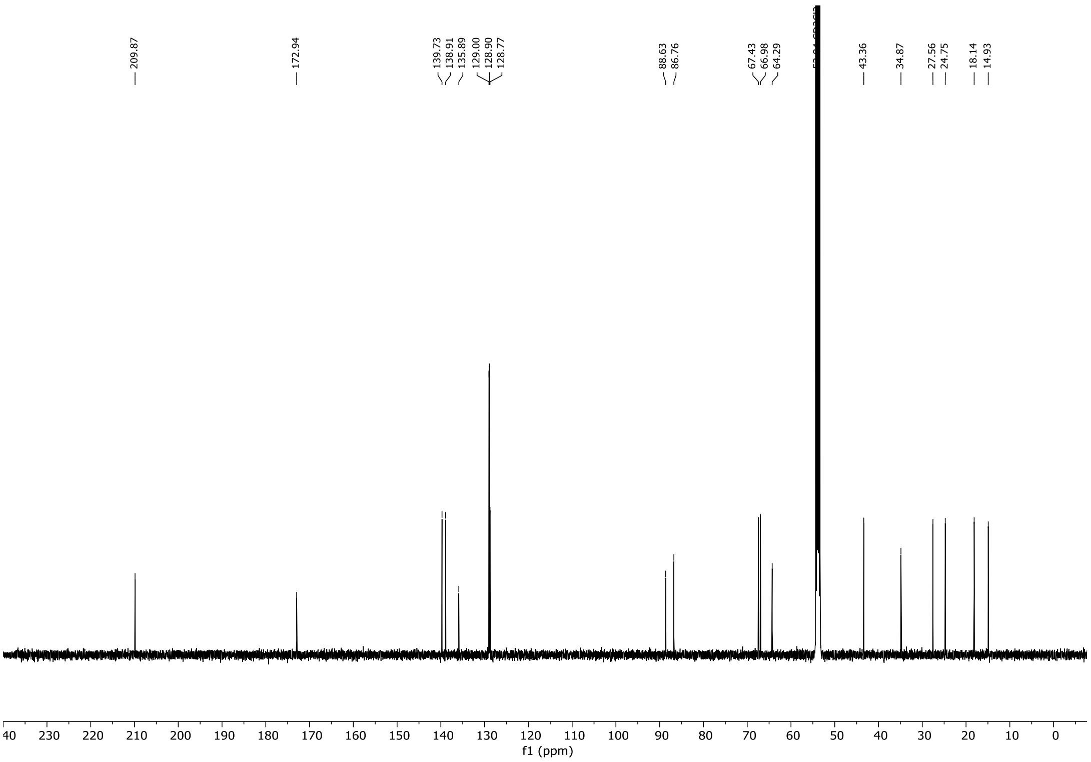

Benzyl 1,4-dibutyl-9-oxo-1,4,5,6,7,8,9,9a-octahydro-4a*H*-1,4-epoxybenzo[7]annulene-4a-carboxylate (*rac*-4h)

^{1}H NMR (400 MHz, CD_2Cl_2):

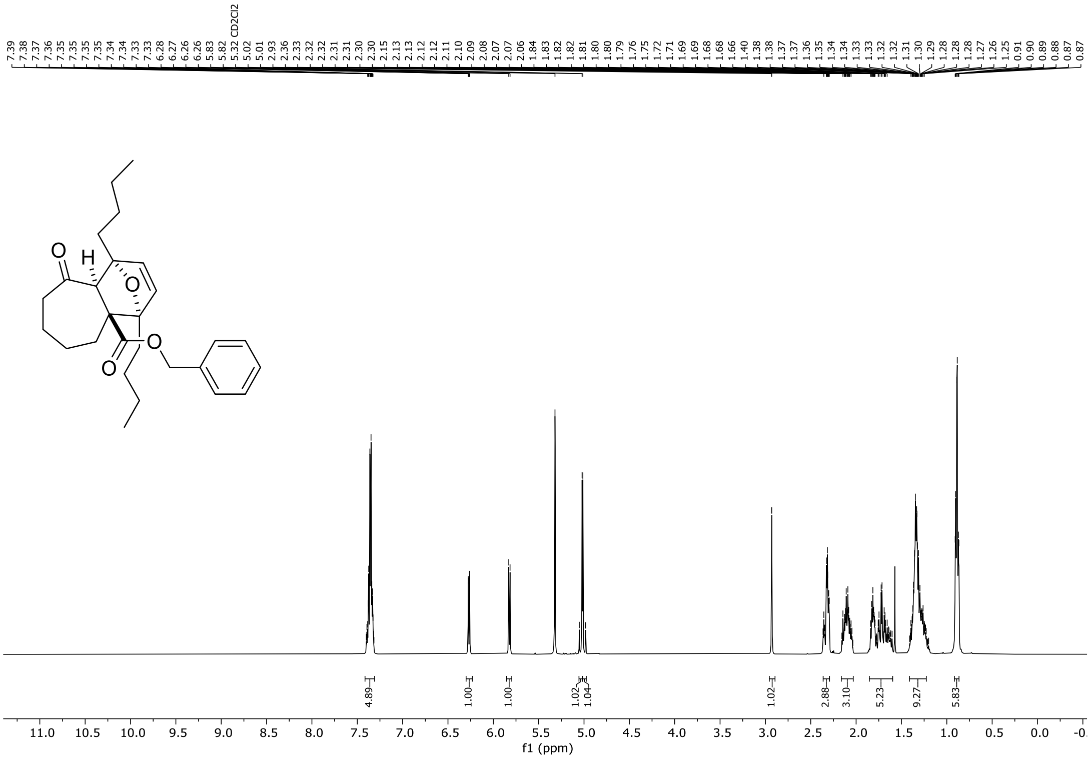

^{13}C NMR (101MHz, CD_2Cl_2):

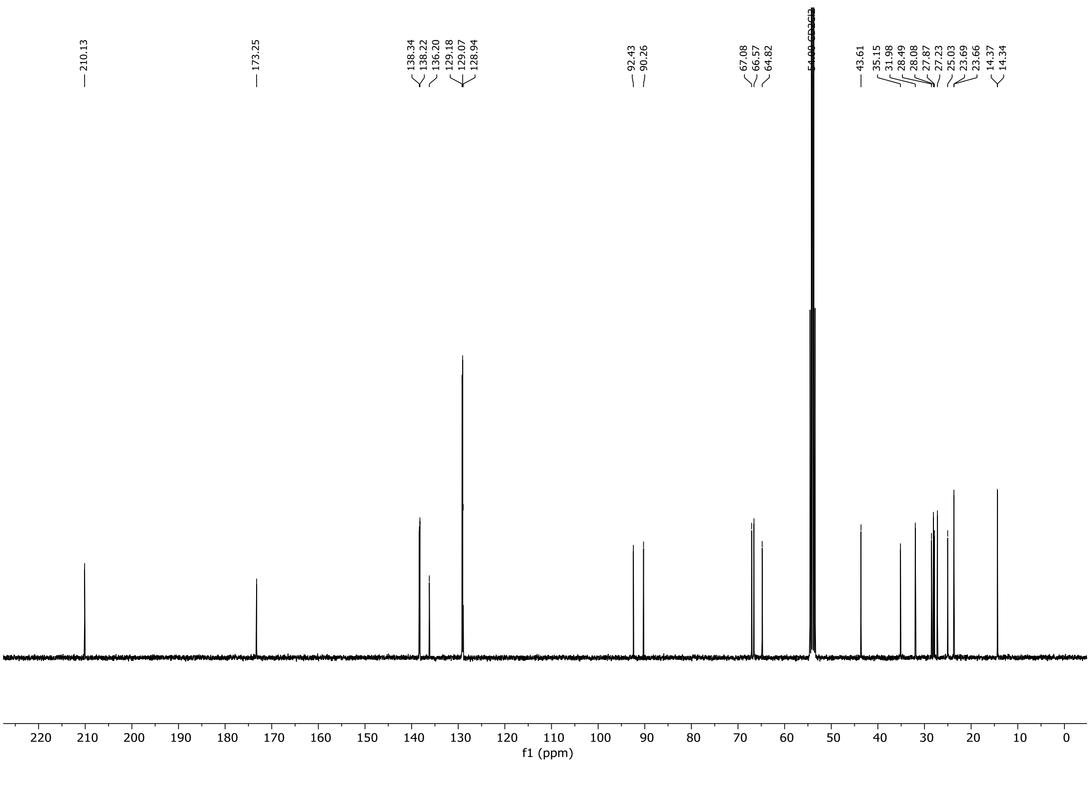

Benzyl 2,3-dimethyl-9-oxo-1,4,5,6,7,8,9,9a-octahydro-4a*H*-benzo[7]annulene-4a-carboxylate (*rac*-4m)

^{1}H NMR (400 MHz, CD_2Cl_2):

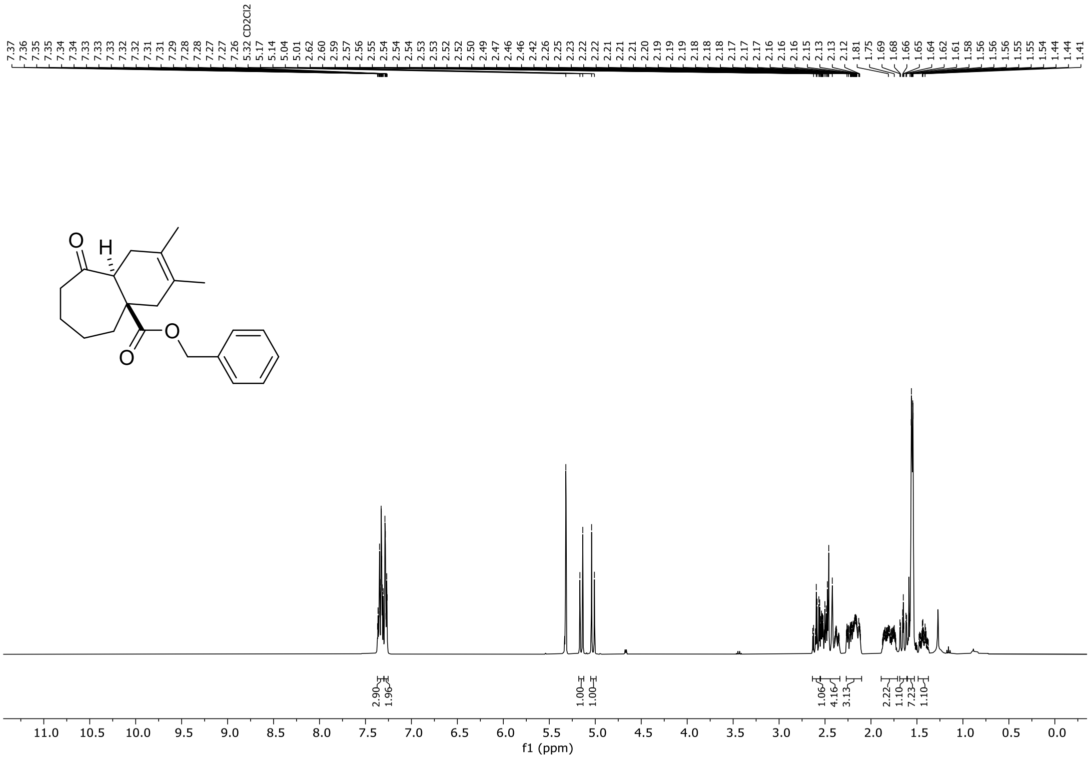

^{13}C NMR (101 MHz, CD_2Cl_2):

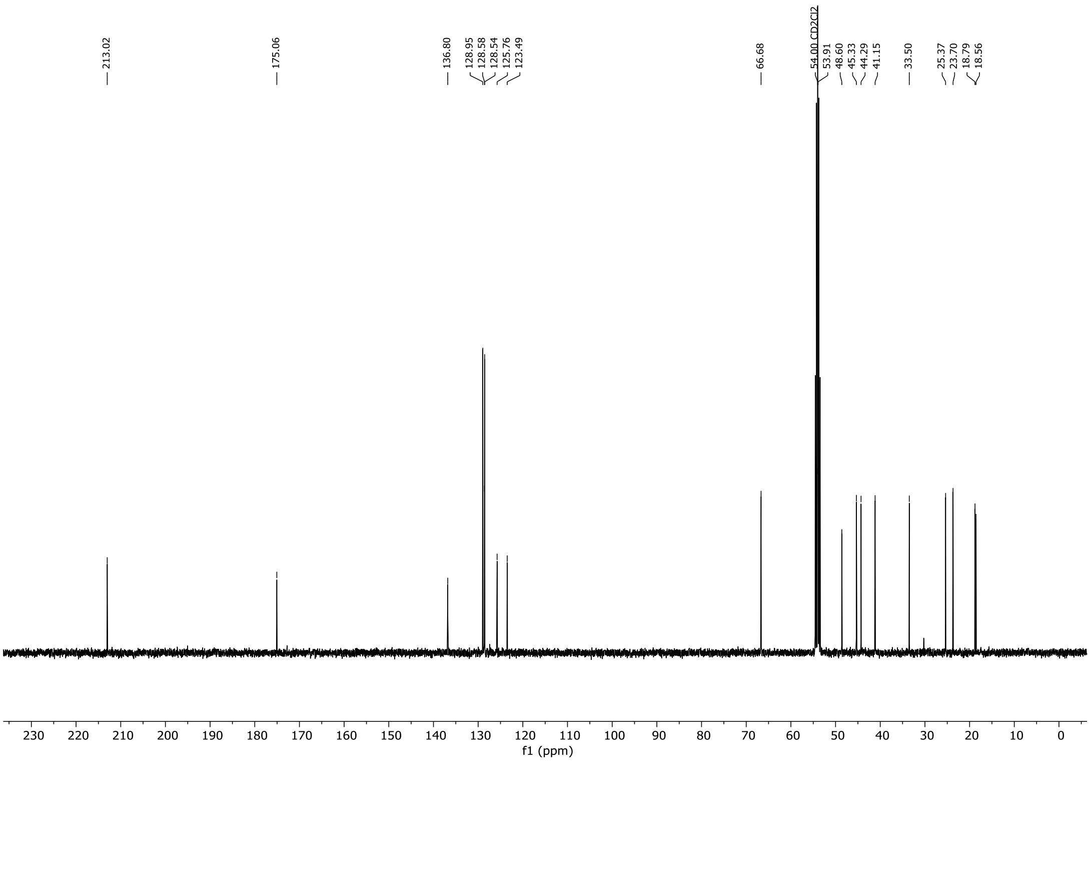

Benzyl 10-oxo-1,2,3,4,5,6,7,8,9,10,10a,11-dodecahydro-5a*H*-cyclohepta[b]naphthalene-5a-carboxylate (*rac*-4n)

^{1}H NMR (400 MHz, CD_2Cl_2):

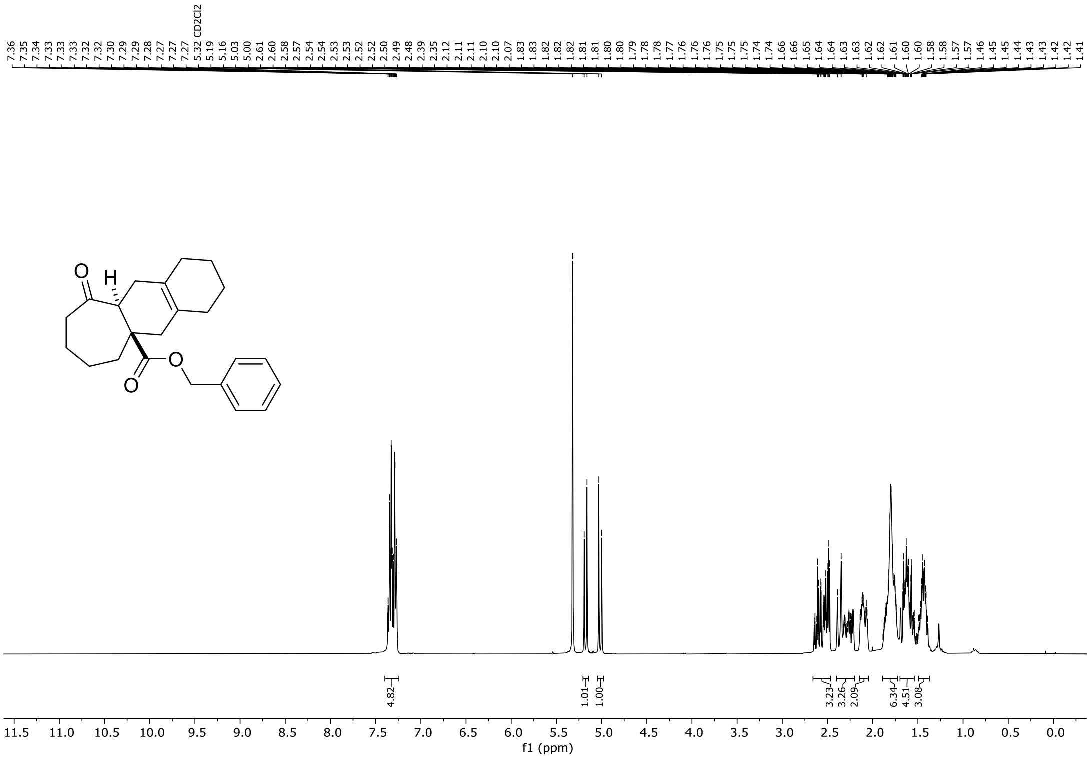

^{13}C NMR (126 MHz, CD_2Cl_2):

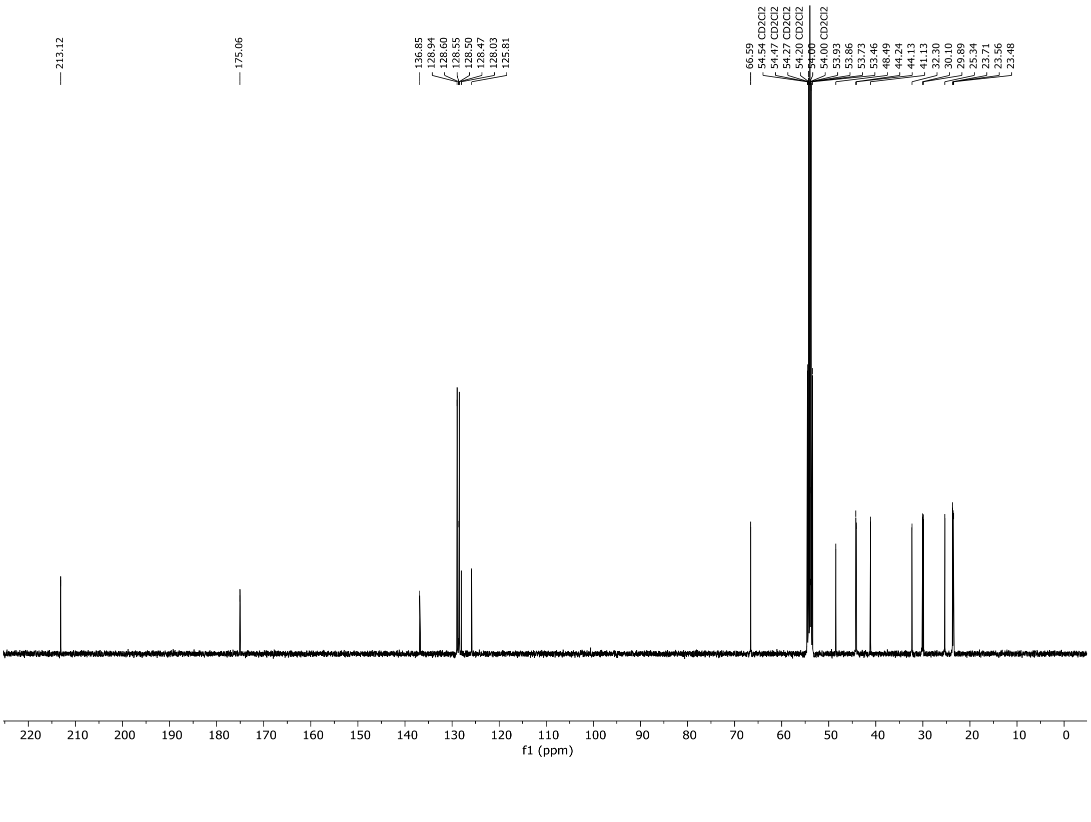

4-Bromobenzyl-1,4-dimethyl-9-oxo-1,4,5,6,7,8,9,9a-octahydro-4a*H*-1,4-epoxybenzo[7]annulene-4a-carboxylate (*rac*-5)

^{1}H NMR (400 MHz, CD_2Cl_2):

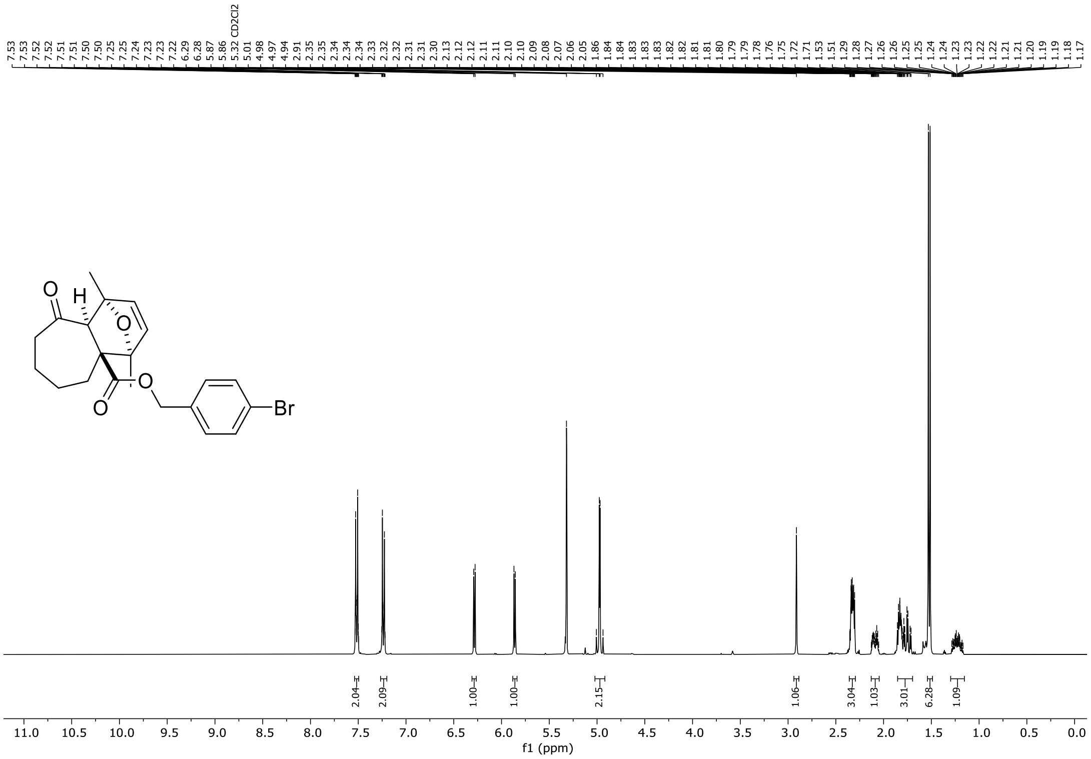

^{13}C NMR (126 MHz, CD_2Cl_2):

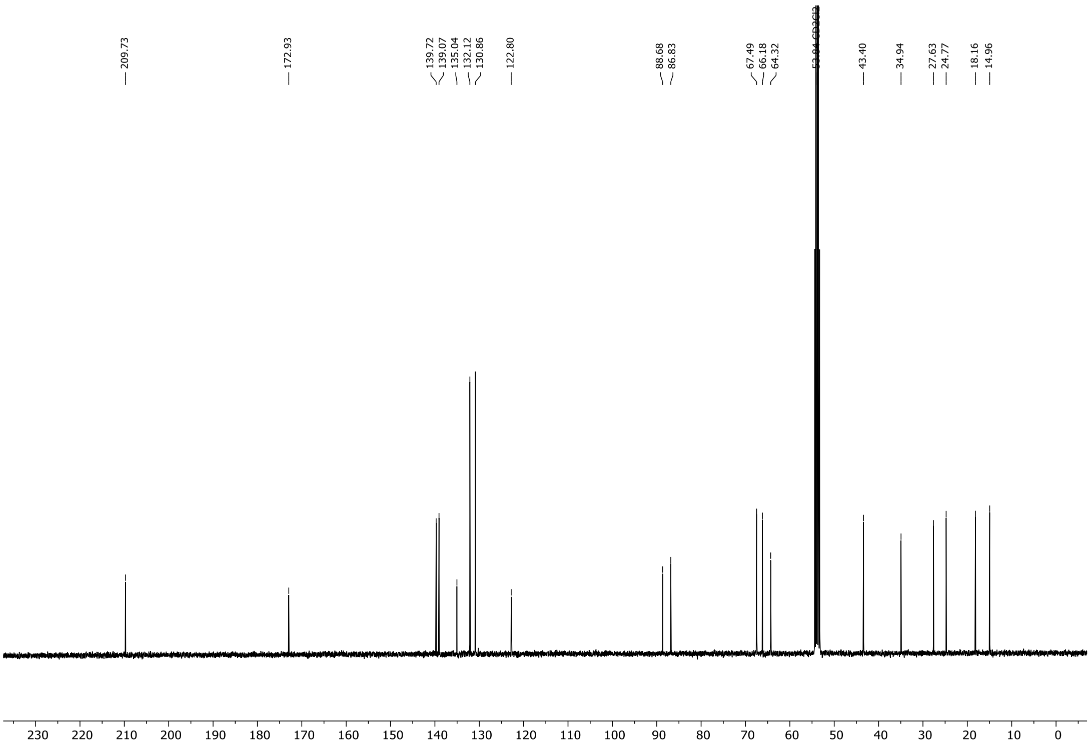

11-Iodo-3a,5-dimethyloctahydro-1*H*,6*H*-3,5-methanocyclohepta[*c*]furo[3,4-*b*]furan-1,6-dione (*rac*-6)

^{1}H NMR (400 MHz, CD_2Cl_2):

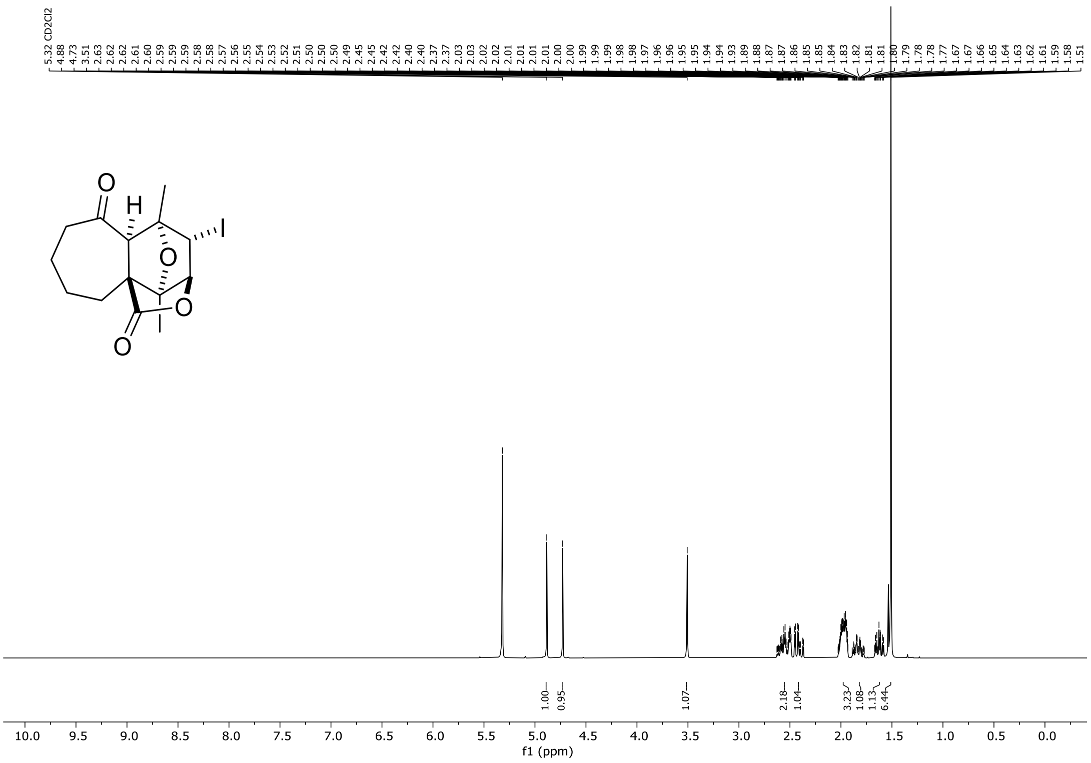

^{13}C NMR (101 MHz, CD_2Cl_2):

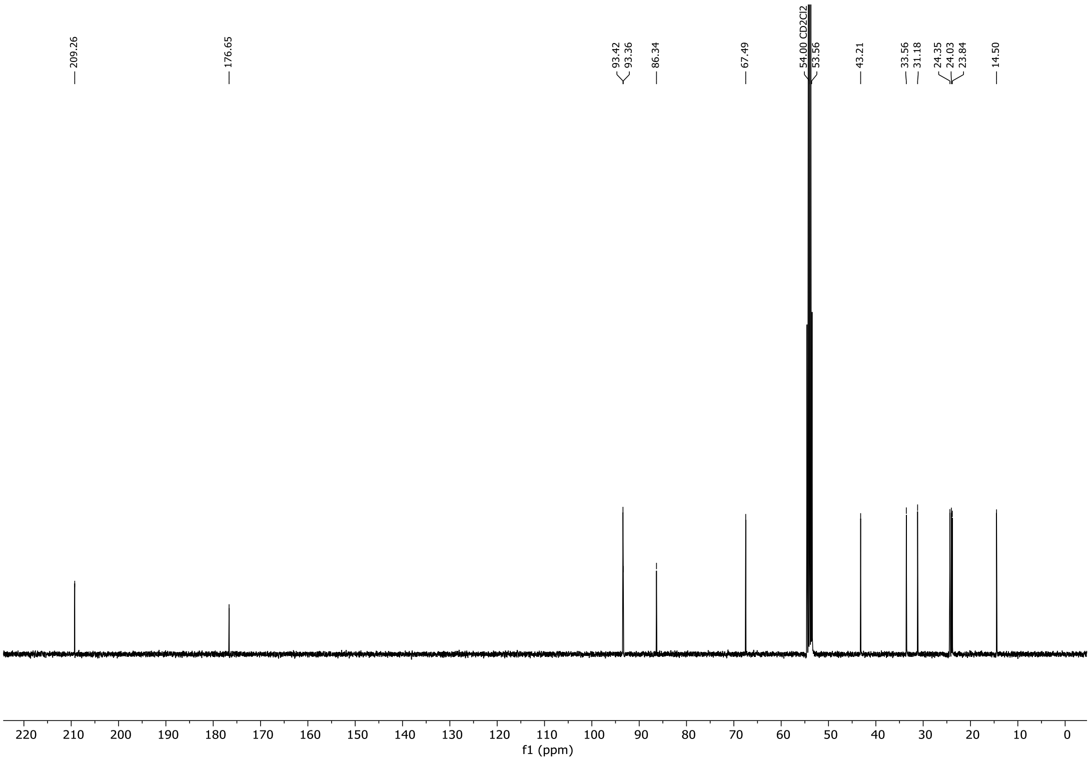

16. Data Sheets of Light Sources

366 nm reactor

Lehrstuhl OC 1 - TUM

200 nm 250 nm 300 nm 350 nm 400 nm 450 nm 500 nm 550 nm 600 nm 650 nm

Datasheet FLT015 RPR-Set1-UV-A

Basic Information

Type	Fluorescent light tube
Description	Set1 (UV-A)
Manufacturer / Supplier	n/a / Rayonet
Order number / Date of purch.	n/a / n/a
Internal lot / serial number	Set1 / FLT015

Specification Manufacturer

Type / size	T5 tube, G5 socket
Mechanical specification	16 mm diameter, 288 mm length
Electrical specification	8 W
Wavelength (range, typ.)	350 nm
Spectral width (FWHM)	~ 30 nm
Datasheet	

Characterization

Description of measurement	Measured with Ocean-optics USB4000 spectrometer using a calibrated setup (cosine corrector/fibre). The cosine corrector was placed at 20 mm distance from a single fluorescent tube at half height.	
Measured dominant wavelength / Int.	365 nm	104 µW/mm²nm
Measured spectral width (FWHM)	18 nm	
Integral Reference intensity / range	2194 µW/cm²	300-450 nm

Spectrum

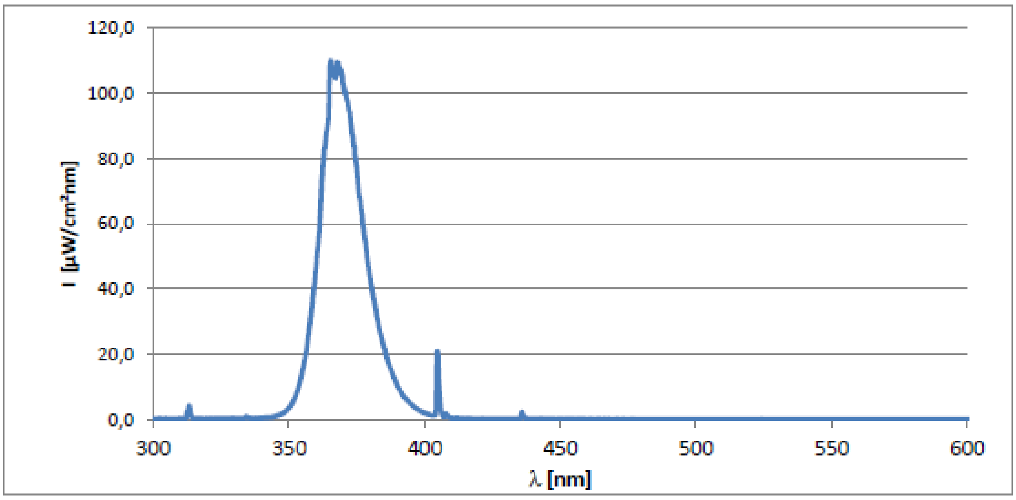

459 nm LED

Lehrstuhl OC 1 - TUM

200 nm 250 nm 300 nm 350 nm 400 nm 450 nm 500 nm 550 nm 600 nm 650 nm

Datasheet LED055 Av-455-3W

Basic Information

Type	High-Power-LED
Description	Avonec 455-460 nm / 3 W
Manufacturer / Supplier	n/a / Avonec
Order number / Date of purch.	n/a / 07/2017
Internal lot / serial number	2017-07 / LED055

Specification Manufacturer

Type / size	single emitter / ca. 1 x 1 mm
Mechanical specification	
Electrical specification	700 mA, UF 3.7 V
Wavelength (range, typ.)	455-460 nm, typ. n/a
Spectral width (FWHM)	n/a
Datasheet	n/a

Characterization

Description of measurement	Measured with Ocean-optics USB4000 spectrometer using a calibrated setup (cosine corrector/fibre). The distance between the emitting surface and the surface of the cosine corrector was 20 mm. The LED was operated at 700 mA on a passive heat-sink at approx. 20 °C	
Measured dominant wavelength / Int.	459 nm	2028 µW/mm²nm
Measured spectral width (FWHM)	20 nm	
Integral Reference intensity / range	49315 µW/cm²	350-500 nm

Spectrum

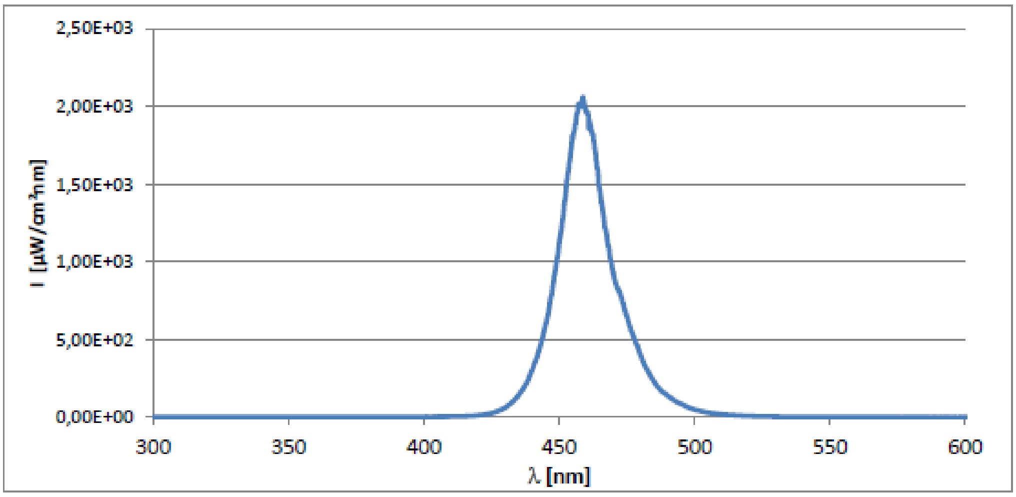

17. References

[1] S. Poplata, T. Bach, *J. Am. Chem. Soc.* **2018**, *140*, 3228.

[2] D. Lenhart, A. Bauer, A. Pöthig, T. Bach, *Chem. Eur. J.* **2016**, *22*, 6519.

[3] C. Asta, J. Conrad, S. Mika, Beifuss,†, Uwe, *Green Chem.* **2011**, *13*, 3066.

[4] D. P. Schwinger, M. T. Peschel, C. Jaschke, C. Jandl, R. de Vivie-Riedle, T. Bach, *J. Org. Chem.* **2022**, *87*, 4838.

[5] Franziska Pecho, You-Quan Zou, Johannes Gramüller, Tadashi Mori, Stefan M. Huber, Andreas Bauer, Ruth M. Gschwind, Thorsten Bach, *Chem. Eur. J.* **2020**, *26*, 5190.

[6] a) A. Korostylev, V. I. Tararov, C. Fischer, A. Monsees, A. Börner, *J. Org. Chem.* **2004**, *69*, 3220; b) Liang Li, Yidong Liu, Yang Peng, Lei Yu, Xiaoyan Wu, Hailong Yan, *Angew. Chem. Int. Ed.* **2016**, *55*, 331.

[7] Y. Yang, S.-F. Zhu, H.-F. Duan, C.-Y. Zhou, L.-X. Wang, Q.-L. Zhou, *J. Am. Chem. Soc.* **2007**, *129*, 2248.

[8] Understanding FT-IR Data Processing, Dr. Werner Herres and Dr. Joern Gronholz, Bruker Analytische Messtechnik GmbH https://mmrc.caltech.edu/FTIR/Literature/General/Understanding%20FTIR.pdf (date of call: 14.01.2025).

[9] R. Bonneau, P. Fornier de Violet, J. Joussot-Dubien, *Nouv. J. Chim.* **1977**, *1*, 31.

[10] C. Müller, T. Pascher, A. Eriksson, P. Chabera, J. Uhlig, *J. Phys. Chem. A* **2022**, *126*, 4087.

[11] a) A. A. Granovsky, *J. Chem. Phys.* **2011**, *134*, 214113; b) T. Shiozaki, W. Győrffy, P. Celani, H.-J. Werner, *J. Chem. Phys.* **2011**, *135,* 081106; c) BAGEL, Brilliantly Advanced General Electronic-structure Library. http://www.nubakery.org under the GNU General Public License; d) T. Shiozaki, *WIREs Comput. Mol. Sci.* **2018**, *8*, e1331.

[12] D. E. Woon, T. H. Dunning, *J. Chem. Phys.* **1994**, *100*, 2975.

[13] J. Patrick Zobel, Juan J. Nogueira, Leticia González, *Chem. Sci.* **2017**, *8*, 1482.

[14] S. Chattopadhyay, R. K. Chaudhuri, U. S. Mahapatra, A. Ghosh, S. S. Ray, *WIREs Comput. Mol. Sci.* **2016**, *6*, 266.

[15] Y. Nishimoto, S. Battaglia, R. Lindh, *J. Chem. Theory.* **2022**, *18*, 4269.

[16] a) J.-D. Chai, M. Head-Gordon, *J. Chem. Phys.* **2008**, *128*, 084106; b) S. Grimme, J. Antony, S. Ehrlich, H. Krieg, *J. Chem. Phys.* **2010**, *132*, 154104; c) F. Neese, *WIREs Comput. Mol. Sci.* **2018**, *8*, e1327.

[17] a) F. Weigend, R. Ahlrichs, *Phys. Chem. Chem. Phys.* **2005**, *7*, 3297; b) F. Weigend, *Phys. Chem. Chem. Phys.* **2006**, *8*, 1057.

[18] M. Casanova-Páez, L. Goerigk, *J. Chem. Theory. Comput.* **2021**, *17*, 5165.

[19] A. Hellweg, C. Hättig, S. Höfener, W. Klopper, *Theor. Chem. Acc.* **2007**, *117*, 587.

[20] A. V. Marenich, C. J. Cramer, D. G. Truhlar, *J. Phys. Chem. B* **2009**, *113*, 6378.

[21] D. Mester, M. Kállay, *J. Chem. Theory. Comput.* **2022**, *18*, 1646.

[22] J. P. Dahl, M. Springborg, *J. Chem. Phys.* **1988**, *88*, 4535.

[23] a) I. Fdez Galván, M. Vacher, A. Alavi, C. Angeli, F. Aquilante, J. Autschbach, J. J. Bao, S. I. Bokarev, N. A. Bogdanov, R. K. Carlson, L. F. Chibotaru, J. Creutzberg, N. Dattani, M. G. Delcey, S. S. Dong, A. Dreuw, L. Freitag, L. M. Frutos, L. Gagliardi, F. Gendron, A. Guissani, L. González, G. Grell, M. Guo, C. E. Hoyer, M. Johansson, S. Keller, S. Knecht, G. Kovačević, E. Källman, G. Li Manni, M. Lundberg, Y. Ma, S. Mai, J. P. Malahdo, P. Å. Malmquist, P. Marquetand, S. A. Mewes, J. Norell, M. Olivucci, M. Oppel, Q. M. Phung, K. Pierloot, F. Plasser, M. Reiher, A. M. Sand, I. Schapiro, P. Sharma, C. J. Stein, L. K. Sørensen, D. G. Truhlar, M. Ugandi, L. Ungur, A. Valentini, S. Vancoillie, V. Veryazov, O. Weser, O.; T. A. Wesołowski, P.-O. Widmark, S. Wouters, A. Zech, J. P. Zobel, R. Lindh, *J. Chem. Theory. Comput.* **2019**, *15*, 5925; b) F. Aquilante, J. Autschbach, A. Baiardi, S. Battaglia, V. A. Borin, L. F. Chibotaru, I. Conti, L. de Vico, M. Delcey, I. Fdez Galván et al., *J. Chem. Phys.* **2020**, *152*, 214117.

[24] F. Plasser, G. Granucci, J. Pittner, M. Barbatti, M. Persico, H. Lischka, *J. Chem. Phys.* **2012**, *137*, 22A514.

[25] A. Jain, E. Alguire, J. E. Subotnik, *J. Chem. Theory. Comput.* **2016**, *12*, 5256.

[26] F. Plasser, S. Mai, M. Fumanal, E. Gindensperger, C. Daniel, L. González, *J. Chem. Theory. Comput.* **2019**, *15*, 5031.

[27] F. Weigend, A. Köhn, C. Hättig, *J. Chem. Phys.* **2002**, *116*, 3175.

[28] a) C. Bannwarth, S. Ehlert, S. Grimme, *J. Chem. Theory. Comput.* **2019**, *15*, 1652; b) Philipp Pracht, Fabian Bohle, Stefan Grimme, *Phys. Chem. Chem. Phys.* **2020**, *22*, 7169; c) P. Pracht, C. Bannwarth, *J. Chem. Theory. Comput.* **2022**, *18*, 6370.

[29] Gaussian 16, Revision A.03, M. J. Frisch, G. W. Trucks, H. B. Schlegel, G. E. Scuseria, M. A. Robb, J. R. Cheeseman, G. Scalmani, V. Barone, G. A. Petersson, H. Nakatsuji, X. Li, M. Caricato, A. V. Marenich, J. Bloino, B. G. Janesko, R. Gomperts, B. Mennucci, H. P. Hratchian, J. V. Ortiz, A. F. Izmaylov, J. L. Sonnenberg, D. Williams-Young, F. Ding, F. Lipparini, F. Egidi, J. Goings, B. Peng, A. Petrone, T. Henderson, D. Ranasinghe, V. G. Zakrzewski, J. Gao, N. Rega, G. Zheng, W. Liang, M. Hada, M. Ehara, K. Toyota, R. Fukuda, J. Hasegawa, M. Ishida, T. Nakajima, Y. Honda, O. Kitao, H. Nakai, T. Vreven, K. Throssell, J. A. Montgomery, Jr., J. E. Peralta, F. Ogliaro, M. J. Bearpark, J. J. Heyd, E. N. Brothers, K. N. Kudin, V. N. Staroverov, T. A. Keith, R. Kobayashi, J. Normand, K. Raghavachari, A. P. Rendell, J. C. Burant, S. S. Iyengar, J. Tomasi, M. Cossi, J. M. Millam, M. Klene, C. Adamo, R. Cammi, J. W. Ochterski, R. L. Martin, K. Morokuma, O. Farkas, J. B. Foresman, and D. J. Fox, Gaussian, Inc., Wallingford CT, **2016**.

[30] a) R. A. Kendall, T. H. Dunning, R. J. Harrison, *J. Chem. Phys.* **1992**, *96*, 6796; b) G. Scalmani, M. J. Frisch, *J. Chem. Phys.* **2010**, *132*, 114110.

[31] a) H. Kruse, S. Grimme, *J. Chem. Phys.* **2012**, *136*, 154101; b) E. Caldeweyher, C. Bannwarth, S. Grimme, *J. Chem. Phys.* **2017**, *147*, 034112; c) E. Caldeweyher, S. Ehlert, A. Hansen, H. Neugebauer, S. Spicher, C. Bannwarth, S. Grimme, *J. Chem. Phys.* **2019**, *150*, 154122; d) S. Grimme, A. Hansen, S. Ehlert, J.-M. Mewes, *J. Chem. Phys.* **2021**, *154*, 064103.

[32] V. Barone, M. Cossi, *J. Phys. Chem. A* **1998**, *102*, 1995.

[33] T. Gasevic, J. B. Stückrath, S. Grimme, M. Bursch, *J. Phys. Chem. A* **2022**, *126*, 3826.

[34] Y. Guo, C. Riplinger, U. Becker, D. G. Liakos, Y. Minenkov, L. Cavallo, F. Neese, *J. Chem. Phys.* **2018**, *148*, 11101.

[35] D. G. Liakos, F. Neese, *J. Phys. Chem. A* **2012**, *116*, 4801.

[36] D. Yepes, F. Neese, B. List, G. Bistoni, *J. Am. Chem. Soc.* **2020**, *142*, 3613.

[37] S. Grimme, *J. Chem. Theory. Comput.* **2019**, *15*, 2847.

[38] S. Grimme, F. Bohle, A. Hansen, P. Pracht, S. Spicher, M. Stahn, *J. Phys. Chem.. A* **2021**, *125*, 4039.

[39] a) F. Plasser, H. Lischka, *J. Chem. Theory. Comput.* **2012**, *8*, 2777; b) F. Plasser, *J. Chem. Phys.* **2020**, *152*, 84108.

[40] M. T. Peschel, P. Kabaciński, D. P. Schwinger, E. Thyrhaug, G. Cerullo, T. Bach, J. Hauer, R. de Vivie-Riedle, *Angew. Chem. Int. Ed.* **2021**, *133*, 10243.

[41] A. Nenov, R. de Vivie-Riedle, *J. Chem. Phys.* **2011**, *135*.

[42] S. Smidstrup, A. Pedersen, K. Stokbro, H. Jónsson, *J. Chem. Phys.* **2014**, *140*, 214106.

[43] F. Matthias Bickelhaupt, Kendall N. Houk, *Angew. Chem. Int. Ed.* **2017**, *129*, 10204.

[44] O. H. Wheeler, I. Lerner, *J. Am. Chem. Soc.* **1956**, *78*, 63.

[45] J. L. Pawlak, G. A. Berchtold, *J. Org. Chem.* **1988**, *53*, 4063.

[46] J.-Q. Yu, E. J. Corey, *J. Am. Chem. Soc.* **2003**, *125*, 3232.

[47] R. Takagi, T. Tanimoto, *Org. Biomol. Chem.* **2022**, *20*, 3940.

[48] W.-J. Huang, Y.-Y. Ma, L.-X. Liu, B. Wu, G.-F. Jiang, Y.-G. Zhou, *Org. Lett.* **2021**, *23*, 2393.

[49] J. Lyu, M. Leone, A. Claraz, C. Allain, L. Neuville, G. Masson, *RSC Adv.* **2021**, *11*, 36663.

[50] Freya M. Harvey, Alexandra H. Heidecker, Christian Merten, Thorsten Bach, *Org. Biomol. Chem.* **2023**, *21*, 4422.

[51] *APEX4 Suite of Crystallographic Software,* Version 2021-10.0, Bruker AXS Inc., Madison, Wisconsin, USA, **2021**.

[52] Bruker, SAINT, V8.40B, Bruker AXS Inc., Madison, Wisconsin, USA.

[53] L. Krause, R. Herbst-Irmer, G. M. Sheldrick, D. Stalke, *J. Appl. Cryst.* **2015**, *48*, 3.

[54] G. M. Sheldrick, *Acta Cryst. A* **2015**, *71*, 3.

[55] G. M. Sheldrick, *Acta Cryst. C* **2015**, *71*, 3.

[56] C. B. Hübschle, G. M. Sheldrick, B. Dittrich, *J. Appl. Cryst.* **2011**, *44*, 1281.

[57] Ed. E. Prince, *International Tables for Crystallography Volume C, Mathematical, Physical and Chemical Tables*, International Union of Crystallography, Chester, England, **2006**, 500–502; 219–222; 193–199.

[58] C. R. Groom, I. J. Bruno, M. P. Lightfoot, S. C. Ward, *Acta Cryst. B* **2016**, *72*, 171.

[59] D. Kratzert, FinalCif, V125, https://dkratzert.de/finalcif.html.
